# TMSBr-mediated solvent- and work-up-free synthesis of α-2-deoxyglycosides from glycals

**DOI:** 10.3762/bjoc.12.164

**Published:** 2016-08-04

**Authors:** Mei-Yuan Hsu, Yi-Pei Liu, Sarah Lam, Su-Ching Lin, Cheng-Chung Wang

**Affiliations:** 1 Institute of Chemistry, Academia Sinica, Taipei 115, Taiwan; 2 Chemical Biology and Molecular Biophysics Program, Taiwan International Graduate Program, Academia Sinica, Taipei 115, Taiwan; 3 Department of Chemistry, National Taiwan University, Taipei 106, Taiwan; 4 Department of Chemistry, National Central University, Jhongli 320, Taiwan

**Keywords:** 2-deoxyglycosides, glycals, trimethylsilyl bromide (TMSBr), triphenylphosphine oxide (TPPO)

## Abstract

The thio-additions of glycals were efficiently promoted by a stoichiometric amount of trimethylsilyl bromide (TMSBr) to produce *S*-2-deoxyglycosides under solvent-free conditions in good to excellent yields. In addition, with triphenylphosphine oxide as an additive, the TMSBr-mediated direct glycosylations of glycals with a large range of alcohols were highly α-selective.

## Introduction

Deoxyglycosides are essential moieties of numerous bioactive natural products, and are prevalent subunits in antitumor and antibiotic agents [1–3]. Furthermore, 2-deoxy- and 2,6-dideoxyglycosides are crucial components for the pharmacology and bioactivity of many biologically active compounds [4], and were recently observed to inhibit cancer growth [5]. Because of the relevance of 2-deoxyglycosides, great efforts have been made in researching the assembly of oligosaccharides containing these sugars [6–7]. However, the absence of a neighbouring group at C2 causes poor stereoselectivity and high susceptibility to hydrolysis, which are the main obstacles to constructing glycosidic linkages stereoselectively [8]. Some approaches, such as the AgPF_6_-DTBMS [9] and preactivation approach [10], can directly yield stereoselective glycosylations. Indirect methods that utilize auxiliary groups at C2, including halogen atoms [11–18], thio [19–21], and seleno groups [22–25], 1,2-migratory glycosylations that involve sulfur [26–33], oxygen [34], or nitrogen [35–37] atoms as directing groups and long-range directing functionalities at C6 [10,38–42] have also been developed to improve the stereoselectivity. However, additional required steps involving the introduction and removal of directing groups are reducing the efficiency.

Thioglycosides are some of the most commonly used donors for glycosylation reactions because of their high stability and reactivity [43]. Numerous stereoselective synthetic methods that use 2-deoxythioglycosides have been reported [9–10,44–49]. We recently developed a glycosyl chloride-mediated synthesis of highly α-selective 2-deoxyglucosides by using 2-deoxythioglucosides [50]. In the literature, to synthesize 2-deoxythioglycosides, a highly toxic tin hydride reagent was used to produce *S*-2-deoxysugars from glycosyl bromide through an anomeric glycosyl radical and acetate rearrangement, followed by subsequent thioglycosylation to afford 2-deoxythioglycosides as anomeric mixtures [10]. Glycals have been considered as alternative precursors for producing 2-deoxythioglycosides as well as oligosaccharides. Several methods based on the use of glycals in the presence of Lewis acids for *S*- or *O*-2-deoxyglycoside preparations have been developed [51–63]. However, based on the hard and soft (Lewis) acids and bases (HSAB) theory, hard acids would coordinate to the harder O3 in glycals in preference to the softer alkene to initiate an undesired Ferrier rearrangement, leading to the formation of a considerable amount of 2,3-unsatuated glycosides. This constitutes the major competitive reaction pathway in acid-catalysed 2-deoxyglycosylation of glycals [52,57,59]. Besides, unfavourable conditions involving the use of expensive or toxic metal complexes, high temperatures, and long reaction times are usually required in most of the aforementioned methods.

Furthermore, organic solvents in laboratories are associated with numerous health hazards [64], and most of them are consumed during chemical reactions, work-up and purification procedures. Especially, dichloromethane, one of the most general solvents for glycosylation reactions, is acknowledged as an acute inhalation hazard and carcinogen [65–66]. To date, only a few studies of glycosylation under neat conditions have been published. In these methods either the need of heating [67–69] or the use of ball milling [70–72] was demanded. Moreover, the selectivity was manipulated by the neighbouring group effect on C2 [67,69–71], which is absent in 2-deoxyglycosides. Mild, work-up- and solvent-free reaction conditions for highly stereoselective 2-deoxyglycosylation is therefore desirable. Here, we present a solvent- and work-up-free approach to prepare *S*- and α-selective *O-*2-deoxyglycosides from glycals.

## Results and Discussion

In our preliminary study, 77% yield of 2-deoxythiolglucoside **2** was produced exclusively when glucal **1** in the presence of *p*-thiocresol was promoted by a stoichiometric amount of TMSBr under neat conditions at room temperature under ambient atmosphere (Table 1, α:β = 2:1, entry 1). Without work-up and washing, *S*-2-deoxyglycoside **2** could be directly isolated and purified by flash column chromatography. We further extended the scope of the reaction to other glycals by using TMSBr as the promoter under neat conditions (Table 1, entries 2–6). For per-*O*-benzylated glucal (**3**) and per-*O*-acetylated rhamnal (**5**), the corresponding thiol-2-deoxyglycosides **7** (61%, α:β = 2:1) and **10** (76%, α:β = 2:1) were produced in good yields with moderate stereoselectivity (Table 1, entry 2 and 5). Interestingly, as shown in Table 1, entries 1 and 2, the inductive effect of the substituents played critical roles in their reactivity. Electron-withdrawing groups, but not electron-donating groups, in glycals were likely to enhance the reactivity. The effect of the donor conformation on the stereoselectivity of the glycosylation was probed by galactal **4** and fucal **6**. *S*-2-Deoxygalactoside **8** (87%, α:β = 3:1, Table 1, entry 3) and *S*-2-deoxyfucoside **11** (89%, α:β = 3:1, Table 1, entry 6) were produced in high yields with slightly superior selectivity. As shown in Table 1, entry 4, a prolonged reaction time led to further reaction of **8** to give 22% of a dithiol acetal side product **9**.

**Table 1:** TMSBr-mediated thio-addition of glycals.

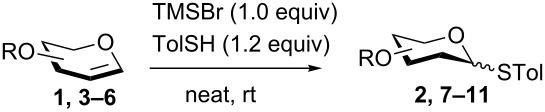			

Entry	Glycal	Time (h)	Yield (α:β)

1	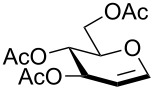 **1**	3	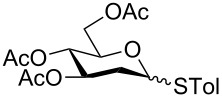 **2**, 77% (2:1)
2	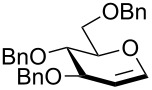 **3**	4	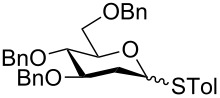 **7**, 61% (2:1)
3	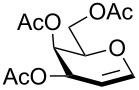 **4**	3	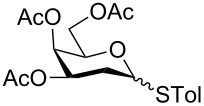 **8**, 87% (3:1)
4	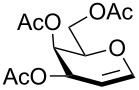 **4**	5	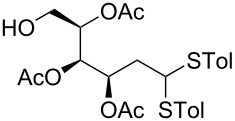 **8**, 63% (3:1); **9**, 22%
5	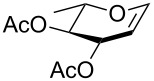 **5**	3	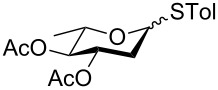 **10**, 76% (2:1)
6	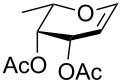 **6**	3	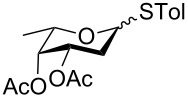 **11**, 89% (3:1)

Inspired by the results obtained in the synthesis of *S*-2-deoxyglycosides, we explored the use of numerous alcohols as acceptors in order to directly synthesize *O*-2-deoxyglycosides from glycals. In Table 2 the reaction of glucal **1** and benzyl alcohol (**12**) under similar reaction conditions was tested and *O*-benzyl 2-deoxyglucoside **25** was produced in 59% yield in the presence of TMSBr (α:β = 3:1, Table 2, entry 1). To further improve the α-selectivity and the yield, various additives were screened (Table 2, entries 2–11) [73–77]. Several participating solvents, dimethylformamide (DMF) (64%, Table 2, entry 2) [76], acetonitrile (ACN) [74] (44%, Table 2, entry 3), tetrahydrofuran (THF) (56%, Table 2, entry 4), and dioxane [75] (50%, Table 2, entry 5) were tested as glycosylation modulators and similar yields of **25** were obtained, but their α-selectivities dramatically improved to α:β = 10:1. In addition, **25** was afforded in 67% with excellent α-selectivity (α:β = 10:1) with the addition of dimethyl sulfide (DMS) (Table 2, entry 6). However, the basic additive 2,4,6-tri-*tert*-butylpyridine (TTBP) produced **25** with poor selectivity (52%, α:β = 2:1, Table 2, entry 7). Furthermore, various phosphine and phosphine oxide reagents were added in *O*-2-deoxyglycosylation reactions (Table 2, entries 8–11); surprisingly, the desired product **25** exhibited a high yield with excellent α-selectivity (78%, α:β = 10:1, Table 2, entry 11) with TPPO [77].

**Table 2:** Additives in TMSBr-mediated 2-deoxyglycosylation of glucal **1**.

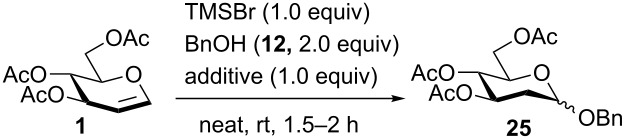			

Entry	Additive	Yield	Ratio (α:β)

1	None	59%	3:1
2	DMF	64%	10:1
3	ACN	44%	10:1
4	THF	56%	10:1
5	dioxane	50%	10:1
6	DMS	67%	10:1
7	2,4,6-tri-*tert*-butylpyridine (TTBP)	52%	2:1
8	triphenylphosphine (TPP)	73%	7:1
9^a^	diphenyl phosphate (DPP)	25%	5:1
10	trimethyl phosphine oxide (TMPO)	50%	2:1
11	triphenyl phosphine oxide (TPPO)	78%	10:1

^a^**1** was recovered in 46%.

Encouraged by these results, we attempted to extend the scope of the glycosylation of 3,4,6-*O*-acetyl- and *O*-benzylglucal (**1** and **3**) with other acceptors (Table 3). Under the optimized conditions, glucal **1** reacted with numerous primary, secondary, and tertiary alcohols, including methanol (**13**), allyl alcohol (**14**), isopropanol (**15**), *tert*-butanol (**16**), 5-azidopentanol (**17**), cyclohexanol (**18**) and 2-adamantanol (**19**), to give *O*-2-deoxyglucosides in high yields (74–90%) and α-selectivities (α:β = 7–10:1, Table 3, entries 2–8). Regarding the glycosylation with amino acid derivatives, L-serine **20** and threonine derivative **21**, increased ratio of β*-*glucosides were formed in their corresponding products **33** (71%, 5:1, Table 3, entry 9) and **34** (79%, 4:1, Table 3, entry 10). For the use of monosaccharides as acceptors, primary monosaccharides **22** and **23** gave disaccharides **35** (80%, Table 3, entry 11) and **36** (78%, Table 3, entry 12) respectively in high yields with moderate α-selectivity (α:β = 4:1). Surprisingly, in the glycosylation with secondary monosaccharide acceptor **24***,* α-disaccharide **37** (56%, Table 3, entry 13) was isolated as the sole product. For per-*O*-benzylated glucal **3**, a higher yield of **38** (97%, Table 3, entry 14) was produced with good selectivity (α:β = 5:1) in the presence of TPPO when compared to the additive-free conditions (79%, Table 3, entry 15). For aliphatic alcohols (**13**–**19**), glycosylation products (**39**–**45**) were always obtained in excellent yields (75–95%) and moderate selectivities (α:β = 3–5:1, Table 3, entries 16–22). The reaction with amino acid residues **20** and **21** (Table 3, entries 23 and 24) produced aminosugars **46** (68%, α:β = 5:1) and **47** (74%, α:β = 4:1) in good yields with moderate α-selectivity. Disaccharides **48** (90%, α:β = 4:1, Table 3, entry 25) and **49** (80%, α:β = 4:1, Table 3, entry 26) were formed in high yields with moderate selectivity similar to the examples of the products of primary monosaccharide acceptors **22** and **23**. Finally, the secondary monosaccharide acceptor **24** (Table 3, entry 27) also underwent complete α*-*selective glycosylation, producing α-disaccharide **50** (67%) as the only product. According to our study, the glycosylation of per-*O*-acetylated glucal **1** with aliphatic alcohols **12**–**19** showed better α-selectivities as compared to the per-*O*-benzylated glucal **3**. However, with amino acid derivatives **20** and **21** and monosaccharides **22**–**24** as acceptors, similar α-selectivities were attained with both glucals **1** and **3**.

**Table 3:** TMSBr-mediated 2-deoxyglycosylation of glucals **1** and **3**.

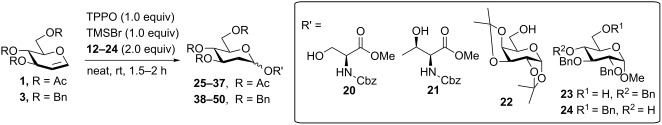				

Entry	Donor	Acceptor	Product	Yield (α:β)

1	**1**	benzyl alcohol (**12**)	**25**	78% (10:1)
2	**1**	methanol (**13**)	**26**	82% (10:1)
3	**1**	3-propenol (**14**)	**27**	78% (7:1)
4	**1**	isopropanol (**15**)	**28**	74% (9:1)
5	**1**	*tert*-butanol (**16**)	**29**	76% (8:1)
6	**1**	5-azidopentanol (**17**)	**30**	90% (10:1)
7	**1**	cyclohexanol (**18**)	**31**	89% (10:1)
8	**1**	2-adamantanol (**19**)	**32**	86% (10:1)
9^a^	**1**	**20**	**33**	71% (5:1)
10^a^	**1**	**21**	**34**	79% (4:1)
11	**1**	**22**	**35**	80% (4:1)
12^a^	**1**	**23**	**36**	78% (4:1)
13^a^	**1**	**24**	**37**	56% α only
14	**3**	**12**	**38**	97% (5:1)
15^b^	**3**	**12**	**38**	79% (6:1)
16	**3**	**13**	**39**	95% (4:1)
17	**3**	**14**	**40**	91% (5:1)
18	**3**	**15**	**41**	93% (5:1)
19	**3**	**16**	**42**	85% (3:1)
20	**3**	**17**	**43**	89% (5:1)
21	**3**	**18**	**44**	81% (5:1)
22	**3**	**19**	**45**	75% (5:1)
23^a^	**3**	**20**	**46**	68% (5:1)
24^a^	**3**	**21**	**47**	74% (4:1)
25	**3**	**22**	**48**	90% (4:1)
26^a^	**3**	**23**	**49**	80% (4:1)
27^a^	**3**	**24**	**50**	67% α only

^a^A minimum amount of CH_2_Cl_2_ was added for solubility. ^b^Without the addition of TPPO.

The results using acetylated galactal **4** were summarized in Table 4. The reactions using several aliphatic alcohols (**12**, **14**–**18**) as acceptors yielded the desired compounds (**51**, **53**–**57**) in excellent yields (90–99%) with high α-selectivities (α:β = 7–9:1, Table 4, entries 1, 3–7). Glycosylation with MeOH (**13**); however, produced **52** in an excellent yield but lower selectivity (95%, α:β = 4:1, Table 4, entry 2). The bulky acceptor 2-adamantanol (**19**) produced compound **58** in a decreased yield but with excellent selectivity because of its low solubility (43%, α:β = 13:1, Table 4, entry 8). L-Serine and threonine derivatives **20** and **21** reacted with galactal **4** to give the glycosylated amino acids **59** and **60** (Table 4, entries 9 and 10) in excellent selectivities (**59**, α only*; ***60**, α:β = 9:1) but in different yields (**59**, 50%*; ***60**, 97%). In Table 4, entry 11, disaccharide **61** was acquired in the presence of **22** with a moderate yield and selectivity (50%, α:β = 4:1). When primary monosaccharide **23** was used as the acceptor, disaccharide **62** was provided in an excellent yield and selectivity (94%, α:β = 10:1, Table 4, entry 12). Additionally, a 60% yield of the α-only product **63** was observed exclusively when the secondary hydroxyl glucoside **24** was used (Table 4, entry 13). Notably, the disubstituted side product was not observed in this reaction.

**Table 4:** TMSBr-mediated 2-deoxyglycosylation of *O*-acetyl galactal **4**.

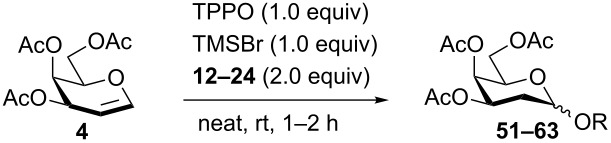			

Entry	Acceptor	Product	Yield (α/β)

1	**12**	**51**	quant. (9:1)
2	**13**	**52**	95% (4:1)
3	**14**	**53**	90% (8:1)
4	**15**	**54**	quant. (7:1)
5	**16**	**55**	quant. (8:1)
6	**17**	**56**	quant. (9:1)
7	**18**	**57**	99% (9:1)
8^a^	**19**	**58**	43% (13:1)
9^a^	**20**	**59**	50 % α only
10^a^	**21**	**60**	97% (9:1)
11	**22**	**61**	50% (4:1)
12^a^	**23**	**62**	94% (10:1)
13^a^	**24**	**63**	60% α only

^a^A minimum amount of CH_2_Cl_2_ was added for solubility.

**Scheme 1:**
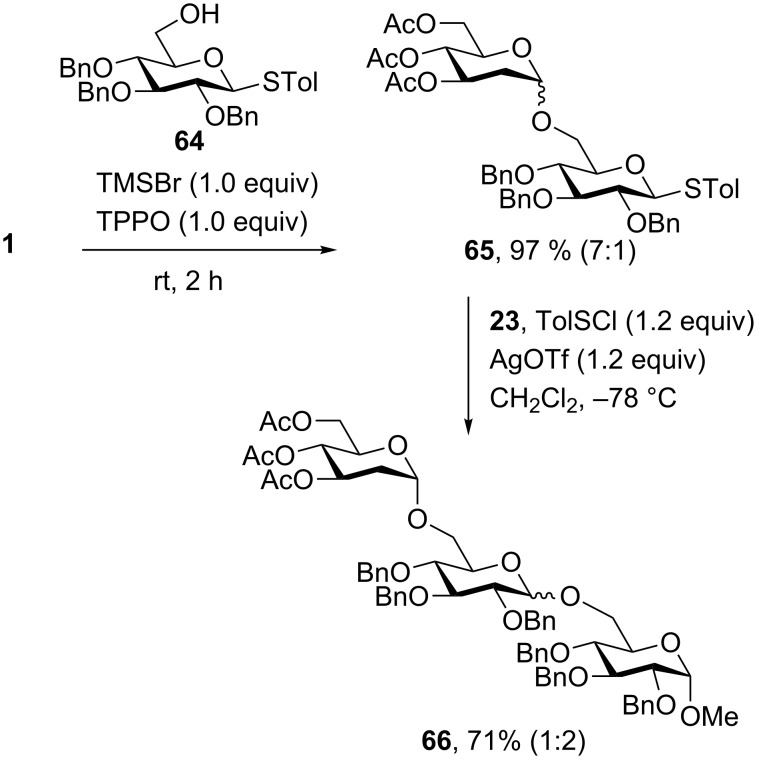
Iterative synthesis of trisaccharide **66**.

On the basis of these results, we demonstrated the applicability of the methodology in oligosaccharide synthesis by synthesising trisaccharide **66** in two sequential steps (Scheme 1). Monosaccharide acceptor **64** underwent the TMSBr-mediated nucleophilic addition to glucal **1** to produce exclusively disaccharide **65** (97%, α:β = 7:1) in an excellent yield with high α-selectivity. Remarkably, the 1-thiol group remained intact after the formation of disaccharide **65**. Subsequently, **65** was coupled with the primary hydroxy saccharide acceptor **23** through a chloride-mediated preactivation glycosylation to afford **66** in 71% yield with moderate selectivity (α:β = 1:2) [16].

**Scheme 2:**
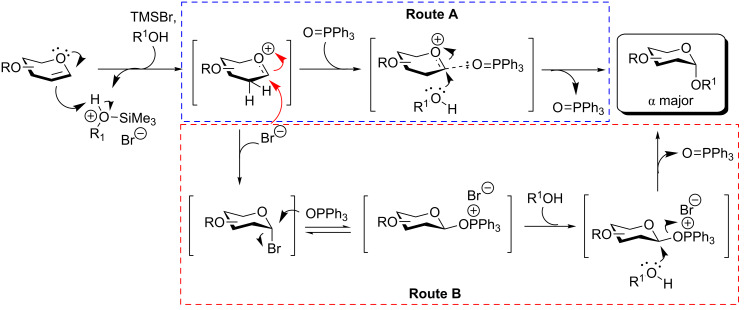
Proposed mechanisms for TMSBr-mediated synthesis of 2-deoxyglycosides in the presence of TPPO.

Two possible mechanisms are proposed for the α-selectivity observed here (Scheme 2). It is well-accepted that the acid-catalysed nucleophilic addition of an alcohol to a glycal is likely to proceed through the formation of an oxocarbenium ion via the protonation at C2 [6,63]. In the presence of TPPO, the oxocarbenium cation is stabilized by the ion–dipole interaction with TPPO oriented preferably at the pseudoequatorial position [78] and the ensuing S_N_2-like displacement by the alcohol contributes to the improvement of the α-selectivity (Scheme 2, route A). Alternatively, it is possible that a 2-deoxyglycosyl bromide is first generated mainly in the more stable α-form [61]. The glycosyl bromide intermediate then undergoes double S_N_2-like substitution by TPPO and the alcohol to give the α-glycoside as the major product [77] (Scheme 2, route B).

## Conclusion

A simple, efficient, and environmentally friendly method for preparing *S*- and *O*-2-deoxyglycosides was established. *S*-2-Deoxyglycosides were obtained with moderate α*-*selectivity when glycals and thiocresol were treated with a stoichiometric amount of TMSBr in neat conditions. Extension of this approach to hydroxy acceptors provided an efficient method to construct the glycosyl bonds between the 2-deoxysugars and the acceptors in good to excellent yields with high α-selectivity in the presence of TPPO, which served as an additive that improved both glycosylation yield and α-selectivity. Without the use of excess solvents, toxic reagents, special equipment, and high temperature, reactions were complete in a few hours at room temperature under ambient atmosphere. Ferrier rearranged products and other side products were not observed. As these reactions were clean, tedious work-up and extraction processes could be obviated prior to purification by flash column chromatography. The utility of this glycosylation method was highlighted by an iterative synthesis of trisaccharide **66**.

## Supporting Information

File 1 Detailed experimental procedures, compound characterization data, and copies of NMR spectra.
