# Supplementary material for: TMSBr-mediated solvent- and work-up-free synthesis of α-2-deoxyglycosides from glycals
Source: Beilstein J Org Chem. 2016 Aug 4;12:1758–64. doi: 10.3762/bjoc.12.164 (PMC4979735; doi:10.3762/bjoc.12.164)
Supplement: File 1 — Detailed experimental procedures, compound characterization data, and copies of NMR spectra. [file Beilstein_J_Org_Chem-12-1758-s001.pdf]

Supporting Information

for

TMSBr-mediated solvent- and work-up-free synthesis of α-2-deoxyglycosides from glycals

Mei-Yuan Hsu[1,2,3], Yi-Pei Liu[1,4], Sarah Lam[1], Su-Ching Lin[1] and Cheng-Chung Wang*[1,2]

Address: [1] Institute of Chemistry, Academia Sinica, Taipei 115 Taiwan, [2]Chemical Biology and Molecular Biophysics Program, Taiwan International Graduate Program, Academia Sinica, Taipei 115, Taiwan, [3]Department of Chemistry, National Taiwan University, Taipei 106, Taiwan and [4]Department of Chemistry, National Central University, Jhongli 320, Taiwan

Email: Cheng-Chung Wang* - wangcc@chem.sinica.edu.tw

*Corresponding author

Experimental section

General experimental. All reactions were conducted in flame-dried glassware under nitrogen atmosphere. Acetonitrile, dichloromethane, *N*,*N*-dimethylformamide (DMF), were purified and dried through activated alumina under argon atmosphere. All reagents obtained from commercial sources were utilized without purification unless otherwise specified. Flash column chromatography was carried out as recommended with silica gel 60 (230–400 mesh, E. Merk). Thin layer chromatography (TLC) was performed on pre-coated glass plates of Silica Gel 60 F254 (0.25 mm, E. Merck); detection was executed by spraying with a solution of $Ce(NH_4)_2(NO_3)_6$, $(NH_4)_6Mo_7O_{24}$ and H_2SO_4 in water and subsequent heating on a hot plate. Optical rotations were measured on a JASCO P-2000 polarimeter. ^{1}H and ^{13}C NMR spectra were recorded with Bruker AV400 and AVⅢ400 MHz instruments. Chemical shifts are in ppm from trimethylsilane (TMS), generated from the $CDCl_3$ lock signal at δ 7.24. Multiplicities are reported by the following abbreviations: s ＝ singlet, d = doublet, t= triplet, q = quartet, m = multiplet, br = broad; *J* = coupling constant values in Hertz. Mass spectra were analyzed by a Waters Premier XE mass spectrometer with ESI mode.

General procedures for preparation of 3,4,6-tri-*O*-acetyl-D-glycals. To a solution of *O*-acetyl-β-D-glycopyranose (10.0 g, 1.0 equiv) in CH_2Cl_2 (100 mL) was added 33% HBr/AcOH (1.20 equiv) at 0 °C. The reaction solution was gradually warm up to room temperature and stirred for 2 hours under nitrogen atmosphere. Then the reaction mixture was quenched by $NaHCO_3$ and then transferred to a separation funnel. The organic layer was separated, and the aqueous layer was extracted with CH_2Cl_2 (50 mL × 2). The organic layers were combined and washed with brine (100 mL × 2), dried over anhydrous $MgSO_4$, filtered, and then concentrated under reduced pressure. The volatiles were removed *in vacuo* for 1 h. Then the crude compound dissolved in a solution of H_2O (30 mL) and AcOH (60 mL) was slowly added activated Zn powder (1.0 equiv) at −15 °C. The reaction solution was stirred violently for 2 hours at 0 °C. The solution was filtered and then extracted with EtOAc (50 mL × 2) via a separation funnel. The combined organic layer was washed with $NaHCO_3$ (50 mL × 2) and brine (50 mL × 2), dried over anhydrous $MgSO_4$, filtered, and then concentrated under reduced pressure. The crude product was purified by flash column chromatography on silica gel and then the volatiles were removed *in vacuo* to obtain the product as colorless oil.

1,2-Dideoxy-3,4,6-tri-*O*-acetyl-D-arabino-1-hexenopyranose (1).[1] colorless oil, $[\alpha]^{28}_{D}$ -17.9 (*c* 1.05, $CHCl_3$); ^1H NMR (400 MHz, $CDCl_3$) δ 6.44 (d, J=6.4Hz, 1 H, H-1), 5.32-5.30 (m, 1 H, H-3), 5.19 (t, J=6.0 Hz, 1 H, H-4), 4.81 (dd, J=6.0, 3.2 Hz, 1 H, H-2), 4.37 (dd, J=11.6, 5.6 Hz, 1 H, H-6), 4.25-4.20 (m, 1 H, H-5), 4.17 (dd, J =7.6, 2.8 Hz, 1 H, H-6), 2.08, 2.05, 2.02 (s, 9 H, 3 OAc) ppm; ^{13}C NMR: δ 170.7-169.7 (C=O), 145.8 (C-1), 99.2 (C-2), 74.2 (C-5), 67.6 (C-3), 67.5 (C-4), 61.6 (C-2), 21.1-20.9 (OAc) ppm; HRMS (ESI, *m/z*) calcd for $C_{12}H_{16}O_7Na$ $[M + Na]^+$ requires 295.0794, found 295.0787.

1,2-Dideoxy-3,4,6-tri-*O*-benzyl-D-arabino-1-hexenopyranose (3). To a solution of tri-*O*-acetyl glucal **1** (10.0 g, 0.038 mol) in MeOH (100 mL) was slowly added sodium methoxide (0.594 g, 0.011 mol). After stirring for 4 hours at room temperature under ambient atmosphere, the reaction mixture was quenched via sequential addition of amberlite IR (120 H^+) acid resin. The solution was filtered and then concentrated under reduced pressure. The residue was dried *in vacuo* overnight. Then the crude product was dissolved in dried DMF. Benzyl bromide (16.0 mL, 0.135 mol) and NaH (60%, 5.40 g, 0.135 mol) was slowly added at 0 °C. The reaction solution was gradually warmed up to room temperature and stirred for 6 hours under nitrogen atmosphere. H_2O (30 mL) was added to quench the reaction. The solution was transferred to a separation funnel. The organic layer was separated, and the

aqueous layer was extracted with EtOAc (50 mL × 2). The combined organic layers were washed with brine (100 mL × 2), dried over anhydrous $MgSO_4$, filtered, and then concentrated under reduced pressure. The crude product was purified by flash column chromatography on silica gel (EtOAc/Hexane= 0/1 to 1/2) and then the volatiles were removed *in vacuo* to obtain the product as a white solid. $[\alpha]^{28}_D$ -16.8 (*c* 1.10, $CHCl_3$); ^1H NMR (400 MHz, $CDCl_3$) δ 7.32-7.23 (m, 15 H, -ArH), 6.42 (dd, *J*= 6.0, 1.2 Hz, 1 H, H-1), 4.87 (dd, *J*=6.0, 1.2 Hz, 1 H, H-2), 4.83 (d, *J*=11.2 Hz, 1 H, PhCH), 4.65-4.59 (m, 2 H, PhCH), 4.57-4.53 (m, 3 H, PhCH), 4.21-4.20 (m, 1 H, H-3), 4.08-4.04 (m, 1 H, H-5), 3.85 (dd, *J*=8.8, 6.4 Hz, 1 H, H-4), 3.78 (dd, *J*=9.6, 5.2 Hz, 1 H, H-6) ppm; ^{13}C NMR: δ 144.9 (C-1), 138.6-138.2 (Ph), 128.5-127.8 (Ph), 100.1 (C-2), 77.0 (C-5), 75.9 (C-3), 74.6 (C-4), 73.9 (CH2), 73.7 (CH2), 70.6 (CH2), 68.7 (CH2) ppm; HRMS (ESI, *m/z*) calcd for $C_{27}H_{28}O_4Na$ $[M + Na]^+$ requires 439.1885, found 439.1882.

1,2-Dideoxy-3,4,6-tri-*O*-acetyl-D-lyxo-1-hexenopyranose (4). Colorless oil, $[\alpha]^{28}_D$ -2.55 (*c* 0.32, $CHCl_3$); ^1H NMR (400 MHz, $CDCl_3$) δ 6.42 (d, *J*=6.4Hz, 1 H, H-1), 5.51 (br, 1 H, H-3), 5.39-5.38 (m, 1 H, H-4), 4.70-4.68 (m, 1 H, H-2), 4.30-4.27 (m, 1 H, H-5), 4.25-4.15 (m, 2 H, H-6), 2.08, 2.04, 1.98 (s, 9 H, 3-OAc) ppm; ^{13}C NMR: δ 170.7 (C=O), 170.4 (C=O), 170.3 (C=O), 145.6 (C-1), 99.0 (C-2), 72.98, 64.1, 64.0, 62.1, 20.9-20.8 (3 OAc) ppm; HRMS (ESI, *m/z*) calcd for $C_{27}H_{28}O_4Na$ $[M + Na]^+$ requires 295.0794, found 295.0786.

1,2,6-Trideoxy-3,4-di-*O*-benzyl-D-arabino-1-hexenopyranose (5). Colorless oil, $[\alpha]^{28}_D$ -61.4 (*c* 0.65, $CHCl_3$); ^1H NMR (400 MHz, $CDCl_3$) δ 6.41 (d, *J*=6.0Hz, 1 H, H-1), 5.33-5.30 (m, 1 H, H-3), 5.01 (dd, *J*=8.0, 2.0 Hz, 1 H, H-4), 4.76 (dd, *J*=6.0, 2.8 Hz, 1 H, H-2), 4.12-4.08 (m, 1 H, H-5), 2.06, 2.02 (s, 6 H, 2 OAc), 1.29 (d, *J*=6.8 Hz, 3 H, Me) ppm; ^{13}C NMR: δ 170.8 (C=O), 170.0 (C=O), 146.2 (C-1), 99.0 (C-2), 72.7 (C-5), 72.1 (C-3), 68.5 (C-4), 21.2 (OAc), 21.0 (OAc), 16.7 (Me) ppm; HRMS (APCI, *m/z*) calcd for $C_{10}H_{14}O_5Na$ $[M + Na]^+$ requires 237.0739, found 237.0733.

1,2,6-Trideoxy-3,4-di-*O*-acetyl-D-lyxo-1-hexenopyranose (6). Colorless oil, $[\alpha]^{28}_D$ -8.53 (*c* 0.85, $CHCl_3$); ^1H NMR (400 MHz, $CDCl_3$) δ 6.45 (dd, *J*=6.4, 1.6 Hz, 1 H, H-1), 5.56-5.55 (m, 1 H, H-3), 5.27-5.26 (m, 1 H, H-4), 4.62 (dd, *J*=8.0, 2.0 Hz, 1 H, H-2), 4.20 (q, *J*=6.4 Hz, 1 H, H-5), 2.14, 2.0 (s, 6 H, 2 OAc), 1.26 (d, *J*=6.5 Hz, 3 H, Me) ppm; ^{13}C NMR: δ 170.7 (C=O), 170.4 (C=O), 146.1 (C-1), 98.2 (C-2), 71.5 (C-3), 66.2 (C-4), 65.0 (C-5), 20.9 (OAc), 20.7 (OAc), 16.5 (Me) ppm; HRMS (APCI, *m/z*) calcd for $C_{10}H_{14}O_5Na$ $[M + Na]^+$ requires 237.0739, found 237.0739.

General procedures for preparation of *p*-tolyl 2-deoxy-1-thio-D-glycopyrano-sides. Glycal (100.0 mg, 1.0 equiv) and *p*-toluenethiol (1.20 equiv) were mixed in a dried round bottomed flask. After the reagents became homogeneous, bromotrimethylsilane (TMSBr, 1.0 equiv) was slowly added. After stirring 3 to 4 hours at room temperature under ambient atmosphere, the reaction mixture was directly purified by flash column chromatography on silica gel and then volatiles were removed *in vacuo* to afford expected products. The products and yields are shown in Table 1.

***p*-Tolyl 3,4,6-tri-*O*-acetyl-2-deoxy-1-thio-D-glucopyranoside (2)**. Colorless oil, $[\alpha]^{28}_{D}$ 127.57 (*c* 0.68, $CHCl_3$); ^1H NMR (400 MHz, $CDCl_3$) δ 7.39 (d, *J*=8.4 Hz, 2 H, Ph-β), 7.34 (d, *J*=8.0 Hz, 2 H, Ph-α), 7.10 (d, *J*=8.8Hz, 2 H, Ph), 5.58 (d, *J*=5.6 Hz, 1 H, H-1α), 5.31-5.26 (m, 1 H, H-3α), 4.98 (t, *J*=9.6 Hz, 1 H, H-4α), 4.92 (t, *J*=9.6 Hz, 1 H, H-4β), 4.71 (dd, *J*=11.6, 2.0 Hz, 1 H, H-1β), 4.54-4.50 (t, *J*=9.6 Hz, 1 H, H-5α), 4.27 (dd, *J*=12.4, 5.6 Hz, 1 H, H-6α), 4.22 (dd, *J*=12.0, 5.2 Hz, 1 H, H-6β), 4.12 (dd, *J*=12.0, 2.4 Hz, 1 H, H-6β), 3.62-2.58(m, 1 H, H-5β), 2.42 (dd, *J*=12.8, 4.8 Hz, 1 H, H-2eq.), 2.33 (s, 3 H, Me-β), 2.30 (s, 3 H, Me-α), 2.20 (ddd, *J*=18.0, 12.0, 6.0 Hz, α-H-2ax.), 2.06-1.99 (s, 9 H, 3 OAc), 1.80 (q, 1 H, β-H-2 ax.) ppm; ^{13}C NMR: δ 170.8-170.0 (C=O), 138.5 (C), 138.0 (C), 130.3-129.0 (Ph), 83.7 (α, C-1), 82.4 (β, C-1), 76.1 (β, C-5), 72.0 (β, C-3), 69.9 (α, C-5), 69.5 (α, C-3), 69.1 (β, C-4), 68.9 (α, C-4), 62.9 (β, C-6), 62.6 (α, C-6), 36.5 (β, C-2), 35.6 (α, C-2), 21.3-20.9 (Me) ppm; HRMS (APCI, *m/z*) calcd for $C_{19}H_{24}O_7NaS$ $[M + Na]^+$ requires 419.1140, found 419.1135.

***p*-Tolyl 3,4,6-tri-*O*-benzyl-2-deoxy-1-thio-D-glucopyranoside (7)**. Colorless oil, $[\alpha]^{28}_{D}$ 117.70 (*c* 0.85, $CHCl_3$); ^1H NMR (400 MHz, $CDCl_3$) δ 7.42 (d, *J*=8.0 Hz, 2 H, Ph-β), 7.40-7.20 (m, 13 H, Ph), 7.03 (d, *J*=7.6 Hz, 2 H, Ph-α), 5.60 (d, *J*=5.6 Hz, 1 H, H-1α), 4.90 (d, *J*=10.8 Hz, 1 H, PhCH-α), 4.88 (d, *J*=10.8 Hz, 1 H, PhCH-β), 4.68 (t, *J*=2.4 Hz, 1 H, Ph-β), 4.66-4.65 (m, 2 H, PhCH and H-1β), 4.62-4.51 (m, 3 H, Ph), 4.32-4.29 (m, 1 H, H-5α), 3.98-3.93 (m, 2 H, H-3α and H-5β), 3.81 (dd, *J*=10.8, 4.0 Hz, 1 H, H-6α), 3.72 (dd, *J*=10.8, 4.8 Hz, 1 H, H-6β), 3.69-3.59 (m, 3 H, H-6, H-4α, and H-3β), 3.49-3.45 (m, 1 H, H-4β), 2.45-2.42 (m, 1 H, H-2eq), 2.29 (s, 3 H, Me), 2.10 (ddd, 1 H, H-2ax), 1.88 (t, 1 H, β-H-2ax) ppm. ^{13}C NMR: δ 138.4-137.2 (Ph), 137.6-137.2 (Ph), 129.9-129.6 (Ph), 128.4-127.4 (Ph), 84.3 (α, C-1), 82.3 (β, C-1), 80.7 (β, C-5), 79.3 (β, C-3), 77.9 (α, C-5), 77.3 (α, C-3), 77.2 (α, C-4), 74.9 (PhCH2), 73.3 (PhCH2), 71.8 (PhCH2), 71.6 (β, C-4), 69.5 (β, C-6), 68.9 (α, C-6), 36.9 (β, C-2), 36.1 (α, C-2), 21.1 (Me) ppm. HRMS (APCI, *m/z*) calcd for $C_{34}H_{36}O_4NaS$ $[M + Na]^+$ requires 563.2232, found 563.2228.

***p*-Tolyl 3,4,6-tri-*O*-acetyl-2-deoxy-1-thio-D-galacopyranoside (8)**. Colorless oil, $[\alpha]^{28}_{D}$ 184.85 (*c* 0.55, $CHCl_3$); ^1H NMR (400 MHz, $CDCl_3$) δ 7.41 (d, *J*=8.0 Hz, 2 H, Ph-β), 7.35 (d, *J*=8.4 Hz, 2 H, Ph-α), 7.09 (d, *J*=8.0 Hz, 2 H, Ph), 5.66 (d, *J*=5.2 Hz, 1 H, H-1α), 5.36 (br, 1 H, H-4α), 5.29-5.23 (m, 2 H, H-3α and H-1β), 4.77-4.73 (m, 1 H, H-4β), 4.69 (t, *J*=6.4 Hz, 1 H, H-5α), 4.17-4.09 (m, 2 H, H-6β), 4.07 (d, *J*= 6.4 Hz, 2 H, H-6α), 3.80 (t, *J*=6.4 Hz, 1 H, H-5β), 2.45 (*J*=25.6, 12.8, 6.4 Hz, 1 H, H-2eq.), 2.32 (s, 3 H, PhMe-β), 2.31 (s, 3 H, PhMe-α), 2.11, 2.02, 1.98, 1.97 (s, 9 H, 3 OAc), 2.08-2.02 (m, 1 H, H-2ax.) ppm. ^{13}C NMR: δ 170.6-170.1 (C=O), 138.2 (Ph), 138.0 (Ph), 132.7-129.7 (Ph), 84.2 (α, C-1), 83.2 (β, C-1), 76.9 (β, C-5), 74.8 (β, C-3), 69.6 (α, C-5), 67.7 (α, C-3, α, C-4), 65.6 (β, C-4), 62.6 (α, C-6), 62.3 (β, C-6), 31.7 (β, C-2), 30.8 (α, C-2), 21.3-20.6 (Me) ppm. HRMS (APCI, *m/z*) calcd for $C_{19}H_{24}O_7NaS$ $[M + Na]^+$ requires 419.1140, found 419.1142.

(2*R*,3*R*,4*R*)-1-hydroxy-6,6-bis(*p*-tolyl)hexane-2,3,4-triyl triacetate (9). Colorless oil, ^1H NMR (400 MHz, $CDCl_3$) δ 7.38 (d, *J*=8.0 Hz, 2 H, Ph), 7.31 (d, *J*=8.0 Hz, 2 H, Ph), 7.12-7.09 (m, 4 H, Ph), 5.38-5.34 (m, 1 H, H-3), 5.29-5.27 (m, 1 H, H-4), 5.29-5.16 (m, 1 H, H-5), 4.18 (dd, *J*=5.6, 4.0 Hz, 1 H, H-1), 3.28 (d, *J*=6.4 Hz, 2 H, H-6), 2.30 (s, 6 H, 2 PhMe), 2.18-2.18 (m, 1 H, H-2), 2.04, 2.01, 1.96 (s, 9 H, 3 OAc), 1.88 (dddd, *J*=24.4, 15.2, 9.6, 2.0 Hz, 1 H, H-2) ppm. ^{13}C NMR: δ 170.1-169.7 (C=O), 138.5 (Ph), 138.3 (Ph), 133.9 (Ph), 133.5 (Ph), 129.9-129.3 (Ph), 71.7 (C-4), 70.0 (C-5), 69.2 (C-3), 55.6 (C-1), 36.6 (C-2), 28.8 (C-6), 21.2 (Me), 20.9-20.5 (3 OAc) ppm. HRMS (APCI, *m/z*) calcd for $C_{26}H_{32}O_7NaS_2$ $[M + Na]^+$ requires 543.1487, found 543.1481.

***p*-Tolyl 3,4-di-*O*-acetyl-2-deoxy-1-thio-D-rhamnopyranoside (10)**. Colorless oil, $[\alpha]^{28}_{D}$ -110.56 (*c* 0.74, $CHCl_3$); ^1H NMR (400 MHz, $CDCl_3$) δ 7.38 (d, *J*=8.0 Hz, 2 H, Ph-β), 7.32 (d, *J*=8.0 Hz, 2 H, Ph-α), 7.12-7.08 (m, 2 H, Ph), 5.50 (d, *J*=5.6 Hz, 1 H, H-1α), 5.28-5.21 (m, 1 H, H-3α), 4.98-4.91 (m, 1 H, H-3β), 4.75 (t, , *J*=9.2 Hz, 1 H, H-4α), 4.72-4.67 (m, 2 H, H-4β and H-1β), 4.39-4.32 (m, 1 H, H-5α), 3.50-3.47 (m, 1 H, H-5β), 2.42 (m, 1 H, 1 H, H-2eq.), 2.39 (s, 3 H, PhMe-β), 2.32 (s, 3 H, PhMe-α), 2.19-2.11 (m, 1 H, α-H-2ax.), 2.03-1.99 (s, 6 H, 2 OAc), 1.77 (q, *J*=12.4 Hz, 1 H, β-H-2ax), 1.23 (d, *J*=6.4 Hz, 1 H, Me-β), 1.14 (d, *J*=6.4 Hz, 1 H, Me-α) ppm. ^{13}C NMR: δ 170.3-170.0 (C=O), 138.1 (Ph), 137.5 (Ph), 133.0 (Ph), 131.9 (Ph), 130. 57 (Ph), 129.7 (Ph), 129.6 (Ph), 128.8 (Ph), 83.3 (α, C-1), 81.7 (β, C-1), 74.8 (α, C-4), 74.2 (β, C-4), 73.8 (β, C-5), 71.7 (β, C-3), 69.3 (α, C-3), 66.6 (α, C-5), 36.5 (β, C-2), 35.7 (α, C-2), 21.1-20.7 (PhMe and OAc), 17.8-17.3 (Me) ppm. HRMS (APCI, *m/z*) calcd for $C_{17}H_{22}O_5NaS$ $[M + Na]^+$ requires 361.1086, found 361.1082.

***p*-Tolyl 3,4-di-*O*-acetyl-2-deoxy-1-thio-D-fucopyranoside (11)**. White soild, $[\alpha]^{28}_{D}$ -181.14 (*c* 0.81, $CHCl_3$); ^{1}H NMR (400 MHz, CDCl3) δ 7.41 (d, *J*=8.4 Hz, 2 H, Ph-β), 7.32 (d, *J*=8.4 Hz, 2 H, Ph-α), 7.11-7.08 (m, 2 H, Ph), 5.64 (d, *J*=6.0 Hz, 1 H, H-1α), 5.28-5.23 (m, 1 H, H-3α), 5.21 (br, 1 H, H-4α), 5,10 (d, *J*=5.2 Hz, 1 H, H-1β), 5.0-4.95 (m, 1 H, H-3β), 4.75-4.72 (m, 1 H, H-4β), 4.54 (q, *J*=6.4 Hz, 1 H, H-5α), 3.67 (q, *J*=6.4 Hz, 1 H, H-5β), 2.41 (ddd, *J*=25.6, 12.8, 6.0 Hz, 1 H, H-2eq.), 2.32 (s, 3 H, PhMe-β), 2.30 (s, 3 H, PhMe-α), 2.13, 1.97 (s, 6 H, 2 OAc), 2.02-2.0 (m, 1 H, α-H-2ax), 1.29-1.24 (m, 1 H, β-H-2ax), 1.20 (d, *J*=6.4 Hz, 1 H, Me-β), 1.12 (d, *J*= 6.4 Hz, 1 H, Me-α) ppm. ^{13}C NMR: δ 170.8-170.1 (C=O), 37.9 (C), 137.5 (C), 132.5-129.7 (Ph), 84.2 (α, C-1), 82.8 (β, C-1), 73.3 (β, C-4), 70.1 (β, C-3), 69.9 (α, C-4), 68.7 (β, C-5), 67.4 (α, C-3), 65.8 (α, C-5), 31.5 (β, C-2), 30.6 (α, C-2), 21.2-20.8 (OAc), 17.1 (β, Me), 16.6 (α, Me) ppm. HRMS (ESI, *m/z*) calcd for $C_{17}H_{22}O_5NaS$ $[M + Na]^+$ requires 361.1086, found 361.1085.

General procedure for preparation of 2-deoxy-D-glycopyranosides. Glycals (50.0 mg, 1.0 equiv), acceptors (**12**–**24**, 2.0 equiv), and triphenylphosphine oxide (TPPO, 1.0 equiv) were mixed in a flame dried flask. After the reagents became homogeneous, TMSBr (1.0 equiv) was slowly added at room temperature under ambient atmosphere. After stirring for 1 to 2 hours, the mixture was directly purified by flash column chromatography on silica gel and then volatiles were removed *in vacuo* to afford expected products. The products and yields are shown in Tables 2–4.

Benzyl 3,4,6-tri-*O*-acetyl-2-deoxy-D-glucopyranoside (25). Colorless oil, $[\alpha]^{28}_{D}$ 80.56 (*c* 0.52, $CHCl_3$); ^{1}H NMR (400 MHz, $CDCl_3$) δ 7.34-7.27 (m, 5 H, Ph), 5.35-5.30 (m, 1 H, H-3), 5.02-4.97 (m, 2 H, H-4 and H-1), 4.66 (d, *J*=12.0 Hz, 1 H, PhCH), 4.49 (d, *J*=12.0 Hz, 1 H, PhCH), 4.27 (dd, *J*=12.4, 4.4 Hz, 1 H, H-6), 4.01-3.96 (m, 2 H, H-6 and H-5), 2.26 (dd, *J*=13.2, 5.6 Hz, 1 H, H-2eq), 2.08, 2.01, 1.98 (s, 9 H, 3 OAc), 1.82 (ddd, *J*=24.8, 12.0, 3.6 Hz, 1 H, H-2ax) ppm; ^{13}C NMR: δ 170.9 (C=O), 170.4 (C=O), 170.1 (C=O), 137.3 (Ph), 128.7 (Ph), 128.2 (Ph), 96.4 (α, C-1), 69.7 (α, C-5), 69.5 (PhCH2), 69.4 (α, C-3), 68.3 (α, C-4), 62.6 (α, C-6), 35.2 (α, C-2), 21.1-20.9 (OAc) ppm; HRMS (ESI, *m/z*) calcd for $C_{19}H_{24}O_8Na$ $[M + Na]^+$ requires 403.1369, found 403.1387.

Methyl 3,4,6-tri-*O*-acetyl-2-deoxy-D-glucopyranoside (26). Colorless oil, $[\alpha]^{28}_{D}$ 111.41 (*c* 0.61, $CHCl_3$); ^{1}H NMR (400 MHz, $CDCl_3$) δ 5.24 (m, 1 H, H-3), 4.95 (t, *J* =9.6 Hz, 1 H, H-4), 4.79 (d, *J*=3.2 Hz, 1 H, H-1), 4.25 (dd, *J*=12.4, 4.4 Hz, 1 H, H-6), 4.02 (dd, *J*=12.4, 2.4 Hz, 1 H, H-6), 3.89 (m, 1 H, H-5), 3.30 (s, 3 H, OCH3), 2.19 (ddd, *J*=12.8, 7.6, 0.8 Hz, 1 H, H-2eq), 2.04, 1.99, 1.96 (3s, 9 H, 3-OAc), 1.78

(ddd, *J*＝24.8, 12.4, 3.6 Hz, 1 H, H-2ax) ppm; ^{13}C NMR: δ 170.9 (C＝O), 170.3 (C＝O), 170.1 (C＝O), 98.2 (α, C-1), 69.6 (α, C-5), 69.3 (α, C-3), 67.9 (α, C-4), 62.6 (α, C-6), 55.0 (α, OCH3), 35.1 (α, C-2), 21.1–20.9 (OAc) ppm; HRMS (ESI, *m/z*) calcd for $C_{13}H_{20}O_8Na$ $[M + Na]^+$ requires 327.1056, found 327.1055.

Allyl 3,4,6-tri-*O*-acetyl-2-deoxy-D-glucopyranoside (27). Colorless oil, $[\alpha]^{28}_D$ 88.13 (*c* 0.58, $CHCl_3$); ^1H NMR (400 MHz, $CDCl_3$) δ 5.86 (m, 1H, -CH=C), 5.32-4.93 (m. 5H, H-3, H-4, -C=CH2, H-1), 4.25 (dd, *J* =12.4, 4.8 Hz, 1H, H-6a), 4.12-4.07 (m, 1H, -O-CH-), 4.02 (dd, *J* = 12.4, 2.0 Hz, 1H, H-6b), 3.96-3.90 (m, 2H, -OCH-, H-5), 2.21 (dd, *J* = 12.4, 2.0 Hz, 1H, H-2eq), 2.03, 1.99, 1.97 (3s, 9H, 3-COCH3), 1.78 (ddd, *J* = 15.6, 12.8, 4.0 Hz, 1H, H-2ax) ppm; ^{13}C NMR: δ 170.9 (C＝O), 1.79.3 (C＝O), 1.70.1 (C＝O), 133.8 (-C=CH2), 117.9 (=CH2), 98.6 (C-1), 70.1 (C-5), 69.6 (C-3), 68.3 (-O-CH2-), 68.1 (C-4), 62.6 (C-6), 55.0 (OCH3), 34.1 (C-2), 21.1–20.9 (OAc) ppm; HRMS (ESI, *m/z*) calcd for $C_{15}H_{22}O_8Na$ $[M + Na]^+$ requires 353.1212, found 353.1226.

Isopropyl 3,4,6-tri-*O*-acetyl-2-deoxy-D-glucopyranoside (28). Colorless oil, $[\alpha]^{28}_D$ 94.13 (*c* 0.50, $CHCl_3$); ^1H NMR (400 MHz, $CDCl_3$) δ 5.31 (m, 1 H, H-3), 5.03 (d, *J*＝3.2 Hz, 1 H, H-1),4.95 (t, *J*＝10 Hz, 1 H, H-4), 4.26 (dd, *J*＝12.4, 4.8 Hz, 1 H, H-6), 4.04- 3.98 (m, 2 H, H-5 and H-6), 3.83 (Sept., *J*＝6.0 Hz, 1 H, O-CH-Me_2), 2.14 (dd, *J*＝13.2, 5.8 Hz, 1 H, H-2eq), 2.05, 2.02, 1.98 (3s, 9 H, 3-OAc), 1.79 (ddd, *J*＝24.4, 12.4, 4.0 Hz, 1 H, H-2ax), 1.19 (d, *J*＝6.0 Hz, 3 H, CH3), 1.12 (d, *J*＝6.4 Hz, 3 H, CH3) ppm; ^{13}C NMR: δ 170.9 (C＝O), 170.4 (C＝O), 170.1 (C＝O), 98.2 (α, C-1), 70.5 (α, C-4), 69.9 (α, O-CH-Me_2), 69.4 (α, C-3), 68.0 (α, C-5), 62.7 (α, C-6), 35.8 (α, C-2), 23.3 (CH3), 22.0 (CH3), 21.1–20.9 (OAc) ppm; HRMS (ESI, *m/z*) calcd for $C_{15}H_{24}O_8Na$ $[M + Na]^+$ requires 355.1369, found 355.1365.

***tert*-Butyl 3,4,6-tri-*O*-acetyl-2-deoxy-D-glucopyranoside (29)**. Colorless oil, $[\alpha]^{28}_D$ 82.68 (*c* 0.60, $CHCl_3$); ^1H NMR (400 MHz, $CDCl_3$) δ 5.34 (m, 1 H, H-3), 5.22 (d, *J*＝3.2 Hz, 1 H, H-1), 4.95 (t, *J*＝9.6 Hz, 1 H, H-4), 4.27 (dd, *J*＝12.4, 4.4 Hz, 1 H, H-6), 4.13 (m, 1 H, H-5), 3.97 (dd, *J*＝12.0, 2.4 Hz, 1 H, H-6), 2.07-1.97 (m, 10H, H-2eq, 3 OAc), 1.80 (ddd, *J* = 24.4, 12.0, 3.6 Hz, 1H, H-2ax), 1.20 (S, 9H, tBu) ppm; ^{13}C NMR: δ 170.9 (C＝O), 170.4 (C＝O), 170.2 (C＝O), 91.7(C-1), 75.5 (-C-(CH3)3), 70.2 (C-5), 69.6 (C-3), 67.7 (C-4), 62.9 (C-6), 36.8 (C-2), 28.7 (-(CH3)3), 21.2-20.9 (OAc) ppm; HRMS (ESI, *m/z*) calcd for $C_{16}H_{26}O_8Na$ $[M + Na]^+$ requires 369.1525, found 369.1522.

5-Azido-1-pentyl 3,4,6-tri-*O*-acetyl-2-deoxy-D-glucopyranoside (30). Colorless oil, $[\alpha]^{28}_{D}$ 81.94 (*c* 0.64, $CHCl_3$); ^{1}H NMR (400 MHz, $CDCl_3$) δ 5.29 (m, 1 H, H-3), 5.04 (d, *J* = 3.2 Hz, 1 H, H-1), 4.90 (t, *J* = 10.0 Hz, 1 H, H-4), 4.21 (d, *J* = 2.8 Hz, 1 H, H-1), 4.25 (dd, *J* = 12.4, 4.8 Hz, 1 H, H-6), 4.02 (dd, *J* = 12.4, 2.4 Hz, 1 H, H-6), 3.90 (m, 1 H, H-5), 3.62-3.60 (m, 1 H), 3.36-3.33 (m, 1 H), 3.25 (t, *J* = 6.4 Hz, 2 H, -O-CH2-), 2.18 (dd, *J* =12.8, 5.2 Hz, 1 H, H-2eq), 2.04, 2.00, 1.97 (3 OAc), 1.78 (ddd, *J* = 24.8, 12.8, 3.6 Hz, 1H, H-2ax), 1.63-1.43 (m, 4 H), 1.45-40 (m, 2 H) ppm; ^{13}C NMR: δ170.9-170.1 (C = O), 97.1 (α, C-1), 69.7 (α, C-4), 69.5 (α, C-3), 68.0 (α, C-5), 67.6 (α, -O-CH2-), 62.7 (α, C-6), 51.5 (-CH2-N3), 35.2 (α, C-2), 29.2 (CH2), 28.8 (CH2), 23.7 (CH2), 21.06–20.89 (OAc) ppm; HRMS (ESI, *m/z*) calcd for $C_{15}H_{43}N_3O_8Na$ $[M + Na]^+$ requires 424.1696, found 424.1705.

Cyclohexyl 3,4,6-tri-*O*-acetyl-2-deoxy-D-glucopyranoside (31). Colorless oil, $[\alpha]^{28}_{D}$ 86.58 (*c* 0.71, $CHCl_3$); ^{1}H NMR (400 MHz, $CDCl_3$) δ 5.28 (m, 1 H, H-3), 4.94 (t, *J* = 10.0 Hz, 1 H, H-4), 4.89 (d, *J* = 2.8 Hz, 1 H, H-1), 4.21 (dd, *J* = 12.8, 5.6 Hz, 1 H, H-6), 4.20 (dd, *J* = 12.4, 2.4 Hz, 1 H, H-6), 4.05-4.0 (m, 2 H, H-6 and H-5), 3.48 (m, 1H, -O-CH(CH2)2-), 2.12 (dd, *J* =12.4 , 4.8 Hz, 1 H, H-2eq), 2.02, 1.97, 1.94 (3 OAc), 1.83-1.47 (m, 11 H, H-2ax, -Cy) ppm; ^{13}C NMR: δ 170.8 (C = O), 170.4 (C = O), 170.1 (C = O), 95.1 (α, C-1), 75.6 (α, -O-CH(CH2)2-), 71.0 (α, C-4), 69.5 (α, C-3), 68.0 (α, C-5), 62.8 (α, C-6), 35.8 (α, C-2), 33.6 (CH2), 32.3 (CH2), 25.7 (CH2), 24.2 (CH2), 24.0 (CH2), 21.1–20.9 (OAc) ppm; HRMS (ESI, *m/z*) calcd for $C_{18}H_{28}O_8Na$ $[M + Na]^+$ requires 395.1682, found 395.1689.

2-Adamantyl 3,4,6-tri-*O*-acetyl-2-deoxy-D-glucopyranoside (32). Colorless oil, $[\alpha]^{28}_{D}$ 82.66 (*c* 0.82, $CHCl_3$); ^{1}H NMR (400 MHz, $CDCl_3$) δ 5.37-5.31 (m, 1 H, H-3), 5.16 (d, *J* = 3.2 Hz, 1 H, H-1), 4.93 (t, *J* = 9.6 Hz, 1 H, H-4), 4.24 (dd, *J* = 12.4, 4.8 Hz, 1 H, H-6), 4.02-4.0 (m, *J* = 9.2 Hz, 2 H, H-6 and H-5), 3.68 (m, 1 H), 2.21 (dd, *J* = 12.8, 5.6 Hz, 1 H, H-2eq), 2.04, 1.99, 1.97, (3 OAc), 2.03-1.41 (m, 13 H, H-2ax and adamantanyl) ppm; ^{13}C NMR: δ 170.8 (C = O), 170.4 (C = O), 170.1 (C = O), 95.6 (α, C-1), 79.8 (-O-CH-), 70.0 (α, C-4), 69.5 (α, C-3), 68.8 (α, C-5), 62.9 (α, C-6), 37.6-27.5 (adamantanyl), 35.8 (α, C-2), 23.3 (-CH3), 22.0 (-CH3), 21.1–20.9 (OAc) ppm; HRMS (ESI, *m/z*) calcd for $C_{22}H_{32}O_8Na$ $[M + Na]^+$ requires 447.1995, found 447.1991.

***O*-[3,4,6-Tri-*O*-acetyl-2-deoxy-D-glucopyranosyl]-N-carbobenzyloxy-L-serine methyl ester (33)**. Colorless oil, $[\alpha]^{28}_{D}$ 66.17 (*c* 1.05, $CHCl_3$); ^{1}H NMR (400 MHz, $CDCl_3$) δ 7.36-7.30 (m, 5 H, Ph), 5.69 (d, *J* = 8.0 Hz, 1 H, NH), 5.23-5.16 (m, 1 H, H-3), 5.12 (s, 2 H, PhCH2), 4.93 (d, *J* = 10.0 Hz, 1 H, H-4), 4.89 (d, *J* = 3.2 Hz, 1 H, H-1), 4.53-4.51 (m, 1 H), 4.23 (dd, *J* = 12.4, 4.8 Hz, 1 H, H-6), 4.01 (dd, *J* = 12.4, 2.0

Hz, 1 H, H-6), 3.91-3.86 (m, 3 H), 3.76 (s, 3 H, OMe), 2.17 (dd, *J*＝12.4, 5.6 Hz, 1 H, H-2eq), 2.05, 2.02, 1.98 (s, 9 H, 3 OAc), 1.78 (ddd, *J* = 24.8, 12.0, 4.0 Hz, 1H, H-2ax) ppm; ^{13}C NMR: δ 170.9-170.1 (C=O), 156.1 (C=O), 136.3 (Ph), 128.8-128.4 (Ph), 97.9 (α, C-1), 69.4 (α, C-4), 69.0 (α, C-3), 68.6 (α, C-5), 68.6 (CH2), 67.4 (CH2), 62.4 (α, C-6), 54.5 (CH), 52.9 (OMe), 35.0 (α, C-2), 21.1-20.9 (OAc) ppm; HRMS (ESI, *m/z*) calcd for $C_{24}H_{31}NO_{12}Na$ $[M + Na]^+$ requires 548.1744, found 548.1745.

***O*-[3,4,6-Tri-*O*-acetyl-2-deoxy-D-glucopyranosyl]-*N*-carbobenzyloxy-L-threonine methyl ester (34)**. Colorless oil, $[\alpha]^{28}_{D}$ 35.68 (*c* 0.54, $CHCl_3$); ^{1}H NMR (400 MHz, $CDCl_3$) δ 7.38-7.29 (m, 5 H, Ph), 5.46 (d, *J*＝9.6 Hz, 1 H, NH), 5.21-5.14 (m, 1 H, H-3), 5.11 (s, 2 H, PhCH2), 5.08 (d, *J*＝3.2 Hz, 1 H, H-1), 4.92-4.88 (m, 2 H), 4.36 (d, *J*＝9.6 Hz, 1 H), 4.29 (d, *J*＝8.4 Hz, 1 H), 4.22 (dd, *J*＝12.0, 8.4 Hz, 1 H, H-6), 4.0 (dd, *J*＝12.8, 1.6 Hz, 1 H, H-6), 3.97-3.94 (m, 1 H, H-5), 3.70 (s, 3 H, OMe), 2.06-1.95 (m, 10 H, H-2eq and 3 OAc), 1.73 (ddd, *J* = 24.4, 12.0, 3.2 Hz, 1H, H-2ax), 1.25 (d, *J*＝6.4 Hz, 3 H, Me). ^{13}C NMR: δ 171.08 (C＝O), 170.77 (C＝O), 170.25 (C＝O), 169.95 (C＝O), 156.73 (C＝O), 136.31 (Ph), 128.67-128.18 (Ph), 98.6 (α, C-1), 97.1 (CH), 76.40 (CH), 69.61 (α, C-4), 69.09 (α, C-3), 68.79 (α, C-5), 67.40 (CH2), 62.57 (α, C-6), 58.81 (CH), 52.62 (OMe), 35.34 (α, C-2), 21.01–20.79 (OAc), 18.40 (Me). HRMS (ESI, *m/z*) calcd for $C_{25}H_{33}NO_{12}Na$ $[M + Na]^+$ requires 562.1900, found 562.1908.

6-*O*-[3,4,6-Tri-*O*-acetyl-2-deoxy-D-glucopyranosyl]-1,2,3,4-di-*O*-isopropylidene-D-galactopyranose (35). Colorless oil, $[\alpha]^{28}_{D}$ 10.91 (*c* 0.76, $CHCl_3$); ^{1}H NMR (400 MHz, $CDCl_3$) δ 5.47 (d, *J*＝4.8 Hz, 1 H, H-1'), 5.27 (ddd, *J*＝20.8, 10.8, 5.6 Hz, 1 H, H-3), 4.99-4.93 (m, 2 H, H-1 and H-4), 4.59 (d, *J*＝8.0 Hz, 1 H, H-6), 4.30-4.27 (m, 2 H, H-2' and H-3'), 4.21 (d, *J*＝8.0 Hz, 1 H, H-6), 4.01-3.96 (m, 2 H, H-5' and H-4'), 3.91 (t, *J*＝6.0 Hz, 1 H, H-5), 3.71 (dd, *J*＝10.4 and 6.4 Hz, 1 H, H-6'), 3.62 (dd, *J*＝10.0 and 6.8 Hz, 1 H, H-6'), 2.23 (dd, *J*＝12.8 and 5.2 Hz, 1 H, H-2eq), 2.04, 1.99, 1.96 (s, 9 H, 3 OAc), 1.77 (ddd, *J*＝24.0, 12.0, 3.6 Hz, 1 H, H-2ax), 1.51, 1.39, 1.31 (s, 12 H, 4 Me) ppm; ^{13}C NMR: δ 170.7-169.7 (C=O), 109.4 (C), 108.6 (C), 97.1 (α, C-1'), 96.3 (α, C-1), 72.9 (CH), 70.6 (CH), 69.3 (CH), 69.2 (CH), 67.9 (CH), 66.3 (α, C-6'), 66.2 (CH), 62.3 (α, C-6), 35.0 (α, C-2), 26.1-24.4 (Me), 20.9-20.8 (OAc) ppm; HRMS (ESI, *m/z*) calcd for $C_{24}H_{36}O_{13}Na$ $[M + Na]^+$ requires 555.2054, found 555.2068.

Methyl 2,3,4-tri-*O*-benzyl-6-*O*-(3,4,6-tri-*O*-acetyl-2-deoxy-Dglucopyranosyl)-*α*-D-glucopyranoside (36). Colorless oil, $[\alpha]^{28}_{D}$ 64.71 (*c* 0.84, $CHCl_3$); ^{1}H NMR (400 MHz, $CDCl_3$) δ 7.36-7.25 (m, 15 H, -Ar-H), 5.31-5.20 (m, 1 H, H-3), 4.99-4.95 (m, 4

H, H-1, H-4, and 2PhCH), 4.80-4.76 (m, 2 H, 2PhCH), 4.67 (d, *J*=12.0 Hz, 1 H, PhCH), 4.61-4.57 (m, 2 H, 2PhCH), 4.12 (dd, *J*=11.6, 4.0 Hz, 1 H, H-6), 4.0 (d, *J*= 9.2 Hz, 1 H, H-3), 3.93-3.86 (m, 2 H, H-5 and H-6), 3.79-3.73 (m, 2 H, H-5 and H-6), 3.60-3.46 (m, 3 H, H-2, H-6, and H-5), 3.34 (s, 3 H, OMe), 2.25 (dd, 1 H, H-2eq), 2.01, 2.0, 1.99 (3s, 9 H, 3 OAc), 1.76 (ddd, *J* = 15.6, 12.0, 4.0 Hz, 1H, H-2ax) ppm; ^{13}C NMR: δ 170.8- 169.8 (C=O), 138.8-138.3 (Ph), 128-6-127.5 (Ph), 98.1(α, C-1), 97.4 (α, C-1), 82.3 (CH), 80.3 (CH), 78.0 (CH), 75.86 (CH2), 75.0 (CH2), 73.5 (CH2), 69.9 (CH), 69.4 (CH), 69.2 (CH), 68.06 (CH), 66.3 (α, C-6), 62.3 (α, C-6), 55.1 (OMe), 35.0 (α, C-2), 21.1–20.8 (OAc) ppm; HRMS (ESI, *m/z*) calcd for $C_{40}H_{48}O_{13}Na$ $[M + Na]^+$ requires 759.2993, found 759.2972.

Methyl 2,3,6-tri-*O*-benzyl-4-*O*-(3,4,6-tri-*O*-acetyl-2-deoxy-α-Dglucopyranosyl)-α-D-glucopyranoside (37). Colorless oil, $[\alpha]^{28}_{D}$ 60.74 (*c* 1.10, $CHCl_3$); ^{1}H NMR (400 MHz, $CDCl_3$) δ 7.31-7.23 (m, 15 H, -ArH), 5.40 (d, *J*=2.8 Hz, 1 H, H-1), 5.22-5.16 (m, 1 H, H-3), 5.02 (d, *J*=11.2 Hz, 1 H, PhCH), 4.88 (t, *J*=9.6 Hz, 1 H, H-4), 4.71 (d, *J*=12.0 Hz, 1 H, PhCH), 4.65-4.59 (m, 4 H, 4PhCH), 4.51 (d, *J*=12.0 Hz, 1 H, PhCH), 4.11 (dd, *J*=12.0, 4.0 Hz, 1 H, H-6), 3.92 (t, *J*=9.2 Hz, 1 H, H-3), 3.90-3.86 (m, 1 H), 3.78-3.74 (m, 2 H), 3.67-3.62 (m, 3 H), 3.50 (dd, *J*=9.6, 3.6 Hz, 1 H, H-6), 3.39 (s, 3 H), 2.03-1.68 (m, 10 H, H-2eq and 3 OAc), 1.60 (m, 1 H, H-2ax) ppm; ^{13}C NMR: δ 170.8 (C=O), 170.3 (C=O), 169.9 (C=O), 138.8 (Ph), 138.3 (Ph), 138.2 (Ph), 128.6-127.7 (Ph), 98.8 (α, C-1), 98.0 (α, C-1), 82.0 (CH), 80.4 (CH), 76.6 (CH), 75.5 (CH2), 73.6 (CH2), 73.4 (CH2), 69.9 (CH), 69.5 (α, C-6), 69.0 (CH), 68.9 (CH), 62.4 (α, C-6), 55.5 (OMe), 35.4 (α, C-2), 21.1–20.9 (OAc) ppm; HRMS (ESI, *m/z*) calcd for $C_{40}H_{48}O_{13}Na$ $[M + Na]^+$ requires 759.2993, found 759.3027.

Benzyl 3,4,6-tri-*O*-benzyl-2-deoxy-D-glucopyranoside (38). Colorless oil, $[\alpha]^{28}_{D}$ 54.99 (*c* 0.59, $CHCl_3$); ^{1}H NMR (400 MHz, $CDCl_3$) δ 7.37-7.17 (m, 20 H, -ArH), 5.67 (d, *J*=3.2 Hz, 1 H, H-1), 4.90 (d, *J*=10.8 Hz, 1 H, PhCH), 4.69-4.62 (m, 4 H, PhCH), 4.54 (d, *J*=11.6 Hz, 1 H, PhCH), 4.44 (d, *J*=11.6 Hz, 1 H, PhCH), 4.05 (m, 1 H, H-3), 3.85-3.77 (m, 2 H, H-5 and H-6), 3.68-3.62 (m, 2 H, H-6 and H-4), 2.34 (dd, *J* =12.8, 5.6 Hz, 1 H, H-2eq), 1.76 (ddd, *J*=24.4, 12.4, 3.6 Hz, 1 H, H-2ax) ppm; ^{13}C NMR: δ 138.9-137.9 (Ph), 128.5-127.9 (Ph), 97.0 (α, C-1), 78.6 (α, C-4), 78.4 (α, C-3), 75.2 (PhCH2), 73.7 (PhCH2), 72.0 (PhCH2), 71.3 (α, C-5), 69.2 (α,C-6), 69.1 (PhCH2), 35.7 (α, C-2) ppm; HRMS (ESI, *m/z*) calcd for $C_{34}H_{36}O_5Na$ $[M + Na]^+$ requires 547.2460, found 547.2483.

Methyl 3,4,6-tri-*O*-benzyl-2-deoxy-D-glucopyranoside (39). Colorless oil, $[\alpha]^{28}_{D}$ 62.98 (*c* 0.65, $CHCl_3$); ^1H NMR (400 MHz, $CDCl_3$) δ 7.37-7.19 (m, 15 H, -ArH), 4.91 (d, *J* = 10.8 Hz, 1 H, PhCH), 4.86 (d, *J* = 2.8 Hz, 1 H, H-1), 4.70-4.52 (m, 5 H, PhCH), 4.01-3.95 (m, 1 H, H-3), 3.80-3.68 (m, 4 H, H-6, H-4, and H-5), 3.32 (s, 3 H), 2.29 (dd, *J* = 13.2, 5.2 Hz, 1 H, H-2eq), 1.72 (ddd, *J* = 24.4, 12.8, 3.6 Hz, 1 H, H-2ax) ppm; ^{13}C NMR: δ 138.9-138.4 (Ph), 128.5-127.7 (Ph), 98.7 (α, C-1), 79.6 (α, C-4), 78.5 (α, C-3), 75.1 (PhCH2), 73.7 (PhCH2), 71.9 (PhCH2), 70.9 (α, C-5), 69.3 (α,C-6), 54.8 (OMe), 35.6 (α, C-2) ppm; HRMS (ESI, *m/z*) calcd for $C_{28}H_{32}O_5Na$ $[M + Na]^+$ requires 471.2147, found 471.2169.

Allyl 3,4,6-tri-*O*-benzyl-2-deoxy-D-glucopyranoside (40). Colorless oil, $[\alpha]^{28}_{D}$ 61.49 (*c* 0.58, $CHCl_3$); ^1H NMR (400 MHz, $CDCl_3$) δ 7.37-7.17 (m, 15 H,-ArH), 5.87-5.83 (m, 1 H, H-3), 5.28 (dd, *J* = 17.2, 1.2 Hz, 1 H, CH=CH), 5.16 (dd, *J* = 10.4, 1.2 Hz, 1 H, CH=CH), 5.01 (d, *J* = 2.4 Hz, 1 H, H-1), 4.91 (d, *J* = 10.8 Hz, 1 H, PhCH), 4.69-4.62 (m, 3 H, PhCH), 4.55-4.51 (m, 2 H, PhCH), 4.16-4.14 (m, 2 H, -O-CH2-), 4.05-4.01 (m, 1 H, H-3), 3.95 (dd, *J* = 13.2, 6.4 Hz, 1 H, H-6), 3.81-3.61 (m, 3 H, H-4, H-5, and H-6), 2.31 (dd, *J* = 12.4, 4.8 Hz, 1 H, H-2eq), 1.75 (ddd, *J* = 24.8, 12.8, 4.0 Hz, 1 H, H-2ax) ppm; ^{13}C NMR: δ 139.0-138.4 (Ph), 128.5-128.7 (Ph), 134.4 (CH=), 117.2 (=CH2), 96.9 (α, C-1), 79.7 (α, C-4), 78.6 (α, C-3), 75.6 (PhCH2), 74.0 (PhCH2), 72.0 (PhCH2), 71.1 (α, C-5), 69.1 (α,C-6), 67.9 (-O-CH2-), 35.8 (α, C-2) ppm; HRMS (ESI, *m/z*) calcd for $C_{30}H_{34}O_5Na$ $[M + Na]^+$ requires 497.2304, found 497.2321.

Isoproropyl 3,4,6-tri-*O*-benzyl-2-deoxy-D-glucopyranoside (41). Colorless oil, $[\alpha]^{28}_{D}$ 60.39 (*c* 0.65, $CHCl_3$); ^1H NMR (400 MHz, $CDCl_3$) δ 7.37-7.17 (m, 15 H, -ArH), 5.09 (d, *J* = 3.2 Hz, 1 H, H-1), 4.90 (d, *J* = 10.8 Hz, 1 H, PhCH), 4.69-4.61 (m, 3 H, PhCH), 4.54-4.50 (m, 2 H, PhCH), 4.05-3.91 (m, 1 H, H-3), 3.88 (sept, *J* = 6.4 Hz, 1 H, CH(Me)2), 3.86-3.79 (m, 2 H, H-5 and H-6), 3.68-3.61 (m, 2 H, H-6 and H-4), 2.25 (dd, *J* = 12.4, 4.8 Hz, 1 H, H-2eq), 1.75 (ddd, *J* = 24.8, 12.8, 4.0 Hz, 1 H, H-2ax) ppm; ^{13}C NMR: δ 139.1-138.5 (Ph), 128.5-127.7 (Ph), 95.3 (α, C-1), 78.8 (α, C-4), 78.5 (α, C-3), 75.2 (PhCH2), 73.7 (PhCH2), 71.9 (PhCH2), 71.5 (α, C-5), 69.3 (α, C-6), 68.4 (α, -CH(Me)2), 36.2 (α, C-2), 23.5 (Me), 21.5 (Me) ppm; HRMS (ESI, *m/z*) calcd for $C_{30}H_{36}O_4Na$ $[M + Na]^+$ requires 499.2460, found 499.2463.

***tert*-Butyl 3,4,6-tri-*O*-benzyl-2-deoxy-D-glucopyranoside (42)**. Colorless oil, $[\alpha]^{28}_{D}$ 48.64 (*c* 0.53, $CHCl_3$); ^1H NMR (400 MHz, $CDCl_3$) δ 7.36-7.17 (m, 15 H, -ArH), 5.27 (d, *J* = 2.4 Hz, 1 H, H-1), 4.88 (d, *J* = 10.8 Hz, 1 H, PhCH), 4.69-4.61 (m, 3 H, PhCH), 4.52-4.62 (m, 2 H, PhCH), 4.06-4.01 (m, 1 H, H-3), 3.95 (dt, *J* = 6.8, 2.8 Hz, 1 H,

H-5), 3.80 (dd, *J* = 10.4, 3.6 Hz, 1 H, H-6), 3.65-3.60 (m, 2 H, H-4, and H-6), 2.10 (dd, *J* = 12.4, 4.8 Hz, 1 H, H-2eq), 1.72 (ddd, *J* = 24.8, 12.8, 4.0 Hz, 1 H, H-2ax) ppm; ^{13}C NMR: δ 139.2-138.0 (Ph), 128.5-127.6 (Ph), 92.2 (α, C-1), 80.2 (CH(Me)3), 78.9 (α, C-4), 78.1 (α, C-3), 75.1 (PhCH2), 73.8 (PhCH2), 71 .9 (PhCH2), 70.6 (PhCH2), 69.2 (α,C-6), 37.3 (α, C-2), 29.9 (Me) ppm; HRMS (ESI, *m/z*) calcd for $C_{31}H_{38}O_5Na$ [M + Na]$^+$ requires 513. 2617, found 513.2620.

5-Azido-1-pentyl 3,4,6-tri-*O*-benzyl-2-deoxy-D-glucopyranoside (43). Colorless oil, $[\alpha]^{28}_D$ 52.13 (*c* 0.60, $CHCl_3$); ^{1}H NMR (400 MHz, $CDCl_3$) δ7.36-7.17 (m, 15 H, -ArH), 4.94 (d, *J* = 3.2 Hz, 1 H, H-1), 4.90 (d, *J* = 10.8 Hz, 1 H, PhCH), 4.70-4.61 (m, 3 H, PhCH), 5.40-5.10 (d, *J* = 11.2 Hz, 2 H, PhCH), 4.0 (m, 1 H, H-3), 3.80-3.73 (m, 2 H, H-6 and H-4), 3.62-3.58 (m, 3 H, H-6 and H-5), 3.37-3.35 (m, 1 H), 3.25 (t, *J* = 6.8 Hz, 2 H, -O-CH2-), 2.28 (dd, *J* = 12.8, 4.8 Hz, 1 H, H-2eq), 1.72 (ddd, *J* = 24.8, 12.8, 2.4 Hz, 1 H, H-2ax), 1.64-1.56 (m, 5 H), 1.45-1.40 (m, 2 H) ppm; ^{13}C NMR: δ 139.0-138.5 (Ph), 128.5-127.7 (Ph), 97.6 (α, C-1), 78.6 (α, C-4), 77.9 (α, C-3), 75.2 (PhCH2), 74.0 (PhCH2), 71.6 (PhCH2),71.1 (α, C-5), 69.4 (-O-CH2-), 67.2 (α, C-6), 51.5 (-CH2-N3), 35.8 (α, C-2), 29.3 (CH2), 28.9 (CH2), 13.7 (CH2) ppm; HRMS (ESI, *m/z*) calcd for $C_{32}H_{39}N_3O_5Na$ [M + Na]$^+$ requires 568.2787, found 568.2795.

Cyclohexyl 3,4,6-tri-*O*-benzyl-2-deoxy-D-glucopyranoside (44). Colorless oil, $[\alpha]^{28}_D$ 63.57 (*c* 0.52, $CHCl_3$); ^{1}H NMR (400 MHz, $CDCl_3$) δ 7.36-7.17 (m, 15 H, -ArH), 5.12 (d, *J* = 2.8 Hz, 1 H, H-1), 4.90 (d, *J* = 10.8 Hz, 1 H, PhCH), 4.70-4.64 (m, 3 H, PhCH), 4.52-4.49 (m, 2 H, PhCH), 4.03 (m, 1 H, H-3), 3.87-3.82 (m, 2 H, H-5), 3.79 (dd, *J* = 10.4, 4.0 Hz, 1 H, H-6), 3.68 (dd, *J* = 10.4, 2.4 Hz, 1 H, H-6), 3.61 (t, *J* = 9.2 Hz, 1 H, H-3), 3.56 (m, 1 H, Cy), 2.24 (dd, *J* = 12.8, 4.4 Hz, 1 H, H-2eq), 1.86-1.84 (m, 2 H, Cy), 1.77-1.70 (m, 3 H, Cy and H-2ax), 1.52 (m, 1 H, Cy), 1.32-1.19 (m, 3 H, Cy) ppm; ^{13}C NMR: δ 139.1-138.5 (Ph), 128.5-127.7 (Ph), 95.3 (α, C-1), 78.8 (α, C-4), 78.1 (α, C-3), 75.4 (PhCH2), 74.6 (Cy), 72.0 (PhCH2), 71.0 (α, C-5), 69.1 (α, C-6), 36.3 (α, C-2), 33.6 (CH2), 31.7 (CH2), 24.5 (CH2), 24.3 (CH2), 24.2 (CH2). HRMS (ESI, *m/z*) calcd for $C_{33}H_{40}O_5Na$ [M + Na]$^+$ requires 539.2773, found 539.2773.

2-Adamantyl 3,4,6-tri-*O*-benzyl-2-deoxy-D-glucopyranoside (45). Colorless oil, $[\alpha]^{28}_D$ 73.30 (*c* 0.61, $CHCl_3$); ^{1}H NMR (400 MHz, $CDCl_3$) δ 7.23-7.14 (m, 15 H, -ArH), 5.09 (d, *J* = 2.8 Hz, 1 H, H-1), 4.88 (d, *J* = 10.8 Hz, 1 H, PhCH), 4.69-4.62 (m, 3 H, PhCH), 4.50 (d, *J* = 10.4 Hz, 1 H, PhCH), 4.49 (d, *J* = 10.4 Hz, 1 H, PhCH), 4.07-4.02 (m, 1 H, H-3), 3.85 (dt, *J* = 8.0, 2.0 Hz, 1 H, H-5), 3.78 (dd, *J* = 10.4, 4.0 Hz, 1 H, H-6), 3.65 (dd, *J* = 10.4, 1.6 Hz, 1 H, H-6), 3.60 (d, *J* = 9.2 Hz, 1 H, H-4), 2.21

(dd, J = 12.8, 4.4 Hz, 1 H, H-2eq), 2.05-1.59 (m, 12 H, adamantanyl), 1.45 (d, J = 12.0 Hz, 2 H, adamantanyl) ppm; ^{13}C NMR: δ 139.0-138.5 (Ph), 128.5-127.7 (Ph), 95.5 (α, C-1), 79.0 (CH), 78.9 (CH), 78.0 (α, C-3), 75.2 (PhCH2), 73.6 (PhCH2), 72.0 (PhCH2), 71.2 (α, C-5), 69.4 (α, C-6), 37.8 (CH2), 36.9 (CH2), 36.6 (CH2), 36.5 (CH2), 33.7 (CH), 32.0 (CH2), 31.8 (CH2), 31.3 (CH), 27.7 (CH), 27.5 (CH) ppm; HRMS (ESI, *m/z*) calcd for $C_{37}H_{44}O_7Na$ [M + Na]$^+$ requires 591.3086, found 591.3094.

***O*-[3,4,6-Tri-*O*-benzyl-2-deoxy-D-glucopyranosyl]-*N*-carbobenzyloxy-L-serine methyl ester (46)**. Colorless oil, $[\alpha]^{28}_{D}$ 53.16 (*c* 1.32, $CHCl_3$); ^{1}H NMR (400 MHz, $CDCl_3$) δ 7.23-7.14 (m, 20 H, -ArH), 5.73 (d, J = 6.4 Hz, 1 H, NH), 5.09 (t, d, J = 12.8 Hz, 2 H, CH2Ph), 4.88 (d, J = 2.8 Hz, 1 H, H-1), 4.84 (d, J = 10.8 Hz, 1 H, PhCH), 4.61 (s, 2 H, CH2Ph), 4.58 (d, J = 10.8 Hz, 1 H, PhCH), 4.48-4.42 (m, 3 H, PhCH), 4.92-3.82 (m, 3 H), 3.73 (s, 3 H, OMe), 3.71-3.66 (m, 2 H), 3.64-3.75 (m, 2 H), 2.22 (dd, J = 12.8, 4.4 Hz, 1 H, H-2eq), 1.68 (ddd, J = 24.8, 12.8, 2.4 Hz, 1 H, H-2ax) ppm; ^{13}C NMR: δ 170.8 (C=O), 156.2 (C=O), 138.8-138.3 (Ph), 136.4 (Ph), 128.7-127.8 (Ph), 98.7 (α, C-1), 78.3 (α, C-4), 78.0 (α, C-3), 75.1 (PhCH2), 76.3 (PhCH2), 72.1 (PhCH2), 71.6 (α, C-5), 68.9 (α, C-6), 68.5 (PhCH2), 67.3 (CH2), 54.6 (CH), 52.7 (OMe), 35.5 (α, C-2) ppm; HRMS (ESI, *m/z*) calcd for $C_{39}H_{43}O_9Na$ [M + Na]$^+$ requires 692.2836, found 692.2842.

***O*-[3,4,6-Tri-*O*-benzyl-2-deoxy-D-glucopyranosyl]-*N*-carbobenzyloxy-L-threonine methyl ester (47)**. Colorless oil, $[\alpha]^{28}_{D}$ 45.10 (*c* 0.60, $CHCl_3$); $[\alpha]^{28}_{D}$ 82.66 (*c* 0.82, $CHCl_3$); ^{1}H NMR (400 MHz, CDCl3) δ 7.37-7.14 (m, 30 H, -ArH), 5.36 (d, J = 9.6 Hz, 1 H, NH), 5.13 (s, 2 H, PHCH), 4.88-4.84 (m, 2 H), 4.62-4.60 (m, 3 H), 4.49-4.46 (m, 2 H), 4. 33-4.30 (m, 2 H), 3.89-3.82 (m, 1 H, H-3), 3.78-3.75 (m, 2H), 3.73 (s, 3 H, OCH3), 3.63-3.61 (m, 2 H), 3.55 (t, J = 9.6 Hz, 1 H,), 2.16 (dd, J = 12.8, 4.4 Hz, 1 H, H-2eq), 1.62 (ddd, J = 24.8, 12.8, 2.4 Hz, 1 H, H-2ax) ppm; ^{13}C NMR: δ 171.4 (C=O), 156.8 (C=O), 138.7-138.3 (Ph), 136.4 (Ph), 128.8-127.8 (Ph), 99.3 (α, C-1), 78.3 (α, C-4), 76.9 (α, C-3), 75.8 (CH), 75.2 (PhCH2), 73.7 (PhCH2), 72.0 (PhCH2), 71.7 (α, C-5), 69.1 (PHCH2), 67.5 (α, C-6), 59.0 (NCH), 52.6 (OMe), 35.9 (α, C-2), 29.9 (CH), 18.8 (Me) ppm; HRMS (ESI, *m/z*) calcd for $C_{40}H_{45}O_9Na$ [M + Na]$^+$ requires 706.2992, found 706.3023.

6-*O*-[3,4,6-Tri-*O*-benzyl-2-deoxy-D-glucopyranosyl]-1,2,3,4-di-*O*-isopropylidene-D-galactopyranose (48). Colorless oil, $[\alpha]^{28}_{D}$ 10.44 (*c* 0.92, $CHCl_3$); ^{1}H NMR (400 MHz, $CDCl_3$) δ 7.34-7.15 (m, 15 H, -ArH), 5.50 (d, J = 5.2 Hz, 1 H, H-1’), 5.01 (d, J = 3.2 Hz, 1 H, H-1), 4.86 (d, J = 10.8 Hz, 1 H, PhCH), 4.66-4.47 (m, 7 H), 4.29 (dd, J

=5.2, 2.4 Hz, 1H, H-6), 4.20 (dd, *J*=8.0, 2.0 Hz, 1H, H-6), 4.03-3.97 (m, 1 H), 3.93-3.80 (m, 1 H), 3.80-3.70 (m, 3 H), 3.67-3.61 (m, 3 H), 2.30 (dd, *J*=12.8, 4.4 Hz, 1 H, H-2eq), 1.72 (ddd, *J*=24.8, 12.8, 2.4 Hz, 1 H, H-2ax), 1.50 (s, 3 H, Me), 1.42 (s, 3 H, Me), 1.32 (s, 3 H, Me), 1.30 (s, 3 H, Me) ppm; ^{13}C NMR: δ 139.0-138.5 (Ph), 128.5-127.7 (Ph), 109.5 (C), 108.7 (C), 97.5 (α, C-1'), 96.6 (α, C-1), 78.5 (α, C-4), 78.3 (α, C-3), 75.1 (PhCH2), 73.7 (PhCH2), 72.0 (PhCH2), 70.9 (CH), 70.7 (CH), 69.1 (α, C-6), 65.6 (CH), 66.0 (α, C-6'), 35.7 (α, C-2), 26.4 (Me), 26.3 (Me), 25.1 (Me), 25.0 (Me) ppm; HRMS (ESI, *m/z*) calcd for $C_{39}H_{48}O_{10}Na$ $[M + Na]^+$ requires 699.3145, found 699.3170.

Methyl 2,3,4-tri-*O*-benzyl-6-*O*-(3,4,6-tri-*O*-benzyl-2-deoxy-Dglucopyranosyl)-α-D-glucopyranoside (49). White solid, $[\alpha]^{28}_D$ 59.04 (*c* 0.61, $CHCl_3$); ^{1}H NMR (400 MHz, $CDCl_3$) δ 7.35-7.18 (m, 30 H, -ArH), 4.97 (d, *J*=10.8 Hz, 1 H, PhCH), 4.85 (dd, *J*=10.8, 2.0 Hz, 2 H, PhCH), 4.78 (d, *J*=10.8 Hz, 2 H, PhCH), 4.64 (d. *J*=11.2 Hz, 1 H, PhCH), 4.69-4.50 (m, 7 H, PhCH, H-1, and H-1'), 4.15 (dd, *J*=9.6, 1.6 Hz, 1 H), 4.06 (dd, *J*=10.4, 1.6 Hz, 1 H), 3.98 (t, *J*=9.2 Hz, 1 H), 3.74-3.69 (m, 3 H, H-3), 3.56-3.50 (m, 4 H, H-2'), 3.42 (t, *J*=9.6 Hz, 1 H), 3.34 (s, 3 H, OMe), 2.19 (dd, *J*=12.8, 4.4 Hz, 1 H, H-2eq), 1.68 (ddd, *J*=24.8, 12.8, 2.4 Hz, 1 H, H-2ax) ppm; ^{13}C NMR: δ 139.1-138.4 (Ph), 128.7-127.7 (Ph), 100.3 (α, C-1'), 98.3 (α, C-1), 82.5(CH), 80.1 (CH), 79.6 (CH), 78.5 (CH), 75.9 (PHCH2), 75.6 (CH), 75.2 (PhCH2), 75.0 (PhCH2), 73.7 (PhCH2), 73.6 (PhCH2), 71.7 (PhCH2), 70.0 (CH), 69.8 (α, C-6), 67.9 (α, C-6), 55.3 (OMe), 36.8 (α, C-2) ppm; HRMS (ESI, *m/z*) calcd for $C_{55}H_{60}O_{10}Na$ $[M + Na]^+$ requires 903.4084, found 903.4099.

Methyl 2,3,6-tri-*O*-benzyl-4-*O*-(3,4,6-tri-*O*-benzyl-2-deoxy-α-D-glucopyranosyl)-α-D-glucopyranoside (50). Colorless oil, $[\alpha]^{28}_D$ 44.80 (*c* 0.54, $CHCl_3$); ^{1}H NMR (400 MHz, $CDCl_3$) δ 7.33-7.15 (m, 15 H, -ArH), 5.42 (d, *J*=2.8 Hz, 1 H, H-1), 5.02 (d, *J* =11.2 Hz, 1 H, PhCH), 4.83 (d, *J*=10.8 Hz, 1 H, PhCH), 4.72 (d, *J*=11.6 Hz, 1 H, PhCH), 4.63-4.59 (m, 3 H, PhCH), 4.54-5.51 (m, 4 H, PhCH and H-1'), 4.47-4.2 (m, 2 H, PhCH), 4.35 (d, *J*=12.4 Hz, 1 H, PhCH), 3.88-3.84 (m, 2 H), 3.70-3.62 (m, 5 H), 4.60-3.58 (m, 1 H), 3.53-3.50 (m, 2 H), 3.38 (s, 3 H, OMe), 2.06 (dd, *J*=12.8, 4.4 Hz, 1 H, H-2eq), 1.55 (ddd, *J*=24.8, 12.8, 2.4 Hz, 1 H, H-2ax) ppm; ^{13}C NMR: δ 138.7-138.0 (Ph), 128.4-127.4 (Ph), 99.4(α, C-1'), 97.6 (α, C-1), 82.1 (CH), 80.1 (CH), 78.1 (CH), 77.1 (CH), 76.2 (CH), 75.4 (PhCH2), 74.8 (PhCH2), 73.5 (PhCH2), 73.2 (PhCH2), 71.8 (CH), 71.7 (PHCH), 69.8 (CH), 69.5 (α, C-6), 68.75 (α, C-6), 55.2 (OMe), 35.8 (α, C-2) ppm; HRMS (ESI, *m/z*) calcd for $C_{55}H_{60}O_{10}Na$ $[M + Na]^+$ requires 903.4084, found 903.4074.

Benzyl 3,4,6-tri-*O*-acetyl-2-deoxy-D-galactcopyranoside (51). Colorless oil, $[\alpha]^{28}_{D}$ 109.56 (*c* 0.66, $CHCl_3$); ^1H NMR (400 MHz, $CDCl_3$) δ 7.36-7.27 (m, 5 H,-ArH), 5.33-5.28 (m, 2 H, H-4 and H-3), 5.29 (d, *J*=3.2 Hz, 1 H, H-1), 4.67 (d, *J*=12.4 Hz, 1 H, PhCH), 4.17 (d, *J*=12.4 Hz, 1 H, PhCH), 4.19 (t, *J*=6.8 Hz, 1 H, H-5), 4.11-4.07 (m, 2 H, H-6), 2.11, 2.04, 1.97 (s, 9 H, 3 OAc), 2.09-2.04 (m, 1 H, H-2eq.), 1.91-1.88 (m, 1 H, H-2ax.) ppm; ^{13}C NMR: δ 170.7 (C=O), 128.7-127.8 (Ph), 98.7 (α, C-1), 71.3 (α, C-4), 70.8 (PhCH2), 68.7 (α, C-3), 66.5 (α, C-5), 62.1 (α, C-6), 32.2 (α, C-2), 21.1-20.9 (OAc) ppm; HRMS (ESI, *m/z*) calcd for $C_{19}H_{24}O_8Na$ $[M + Na]^+$ requires 403.1369, found 403.1365.

Methyl 3,4,6-tri-*O*-acetyl-2-deoxy-D-galactcopyranoside (52). Colorless oil, $[\alpha]^{28}_{D}$ 133.20 (*c* 0.55, $CHCl_3$); ^1H NMR (400 MHz, $CDCl_3$) δ 5.31-5.23 (m, 2 H, H-4 and H-3), 4.89 (d, *J*=2.8 Hz, 1 H, H-1), 4.13-4.07 (m, 3 H, H-5 and H-6), 3.34 (s, 3 H, OMe), 2.10, 2.01, 1.96 (s, 9 H, 3 OAc), 2.02-1.95 (m, 1 H, H-2eq.), 1.86-1.80 (m, 1 H, H-2ax.) ppm; ^{13}C NMR: δ 170.7 (C=O), 137.4-137.1 (Ph), 128.7-127.7 (Ph), 98.7 (α, C-1), 66.9 (α, C-4), 66.8 (α, C-3), 66.4 (α, C-5), 62.7 (α, C-6), 30.3 (α, C-2), 21.0-20.8 (OAc) ppm; HRMS (ESI, *m/z*) calcd for $C_{19}H_{24}O_8Na$ $[M + Na]^+$ requires 327.1056, found 327.1059.

Allyl 3,4,6-tri-*O*-acetyl-2-deoxy-D-galactcopyranoside (53). Colorless oil, $[\alpha]^{28}_{D}$ 133.13 (*c* 0.51, $CHCl_3$); ^1H NMR (400 MHz, $CDCl_3$) δ 5.87-5.83 (m, 1 H, -CH=), 5.30-5.23 (m, 4 H, =CH2, H-3 and H-4), 5.02 (d, *J*=3.2 Hz, 1 H, H-1), 4.15-4.05 (m, 4 H, H-5, H-6, and –O-CH2-), 3.94 (dd, *J*=12.0, 5.6 Hz , 1 H, -O-CH2-), 2.09, 2.01, 1.94 (s, 9 H, 3 OAc), 2.07-2.02 (m, 1 H, H-2eq.), 1.87-1.83 (m, 1 H, H-2ax.) ppm; ^{13}C NMR: δ 170.6-170.1 (C=O), 133. 9 (-CH=), 117.6 (=CH2), 98.7 (α, C-1), 68.4 (-O-CH2-), 67.0 (α, C-4), 66.9 (α, C-3), 66.4 (α, C-5), 62.6 (α, C-6), 30.3 (α, C-2), 21.0-20.8 (OAc) ppm; HRMS (ESI, *m/z*) calcd for $C_{15}H_{22}O_8Na$ $[M + Na]^+$ requires 355.1212, found 355.1207.

Isopropyl 3,4,6-tri-*O*-acetyl-2-deoxy-D-galactcopyranoside (54). Colorless oil, $[\alpha]^{28}_{D}$ 129.86 (*c* 0.73, $CHCl_3$); ^1H NMR (400 MHz, $CDCl_3$) δ 5.27-5.22 (m, 2 H, H-4 and H-3), 5.08 (d, *J*=3.6 Hz, 1 H, H-1), 4.16 (t, *J*=6.8 Hz, 1 H, H-5), 4.09-4.01 (m, 2 H, H-6), 3.82 (sept., *J*=6.4 Hz, 1 H, H-5), 2.10, 2.01, 1.92 (s, 9 H, 3 OAc), 2.08-2.03 (m, 1 H, H-2eq.), 1.77-1.73 (m, 1 H, H-2ax.), 1.19 (d, *J*=6.0 Hz, 3 H, Me), 1.13 (d, *J*=6.0 Hz, 3 H, Me) ppm; ^{13}C NMR: δ 170.6-170.2 (C=O), 95.8 (α, C-1), 69.72 (-O-C(Me)2), 67.1 (α, C-4), 66.8 (α, C-3), 66.6 (α, C-5), 62.7 (α, C-6), 30.9 (α, C-2), 23.3(CH), 21.7 (2 Me), 21.0-20.84 (OAc) ppm; HRMS (ESI, *m/z*) calcd for $C_{15}H_{24}O_8Na$ $[M + Na]^+$ requires 355.1369, found 355.1373.

***tert*-Butyl 3,4,6-tri-*O*-acetyl-2-deoxy-D-galactcopyranoside (55)**. Colorless oil, $[\alpha]^{28}_{D}$ 114.78 (*c* 0.69, $CHCl_3$); ^1H NMR (400 MHz, $CDCl_3$) δ 5.32-5.27 (m, 3 H, H-3, H-4, and H-1), 4.30 (t, d, *J* = 6.8 Hz, 1 H, H-5), 5.07-3.99 (m, 2 H, H-6), 2.09, 2.02, 1.95 (s, 9 H, 3 OAc), 2.07-2.03 (m, 1 H, H-2eq.), 1.69-1.65 (m, 1 H, H-2ax.), 1.21 (s, 9 H, 3 Me) ppm; ^{13}C NMR: δ 170.7-170.3 (C=O), 92.2 (α, C-1), 75.2 (α, C), 67.2 (α, C-4), 66.73 (α, C-3), 66.4 (α, C-5), 62.7 (α, C-6), 31.9 (α, C-2), 28.7 (α, C-2), 21.0-20.8 (OAc) ppm; HRMS (ESI, *m/z*) calcd for $C_{16}H_{26}O_8Na$ $[M + Na]^+$ requires 369.1525, found 355.1524.

5-Azido-1-pentyl 3,4,6-tri-*O*-acetyl-2-deoxy-D-galactcopyranoside (56). Colorless oil, $[\alpha]^{28}_{D}$ 82.04 (*c* 0.77, $CHCl_3$); ^1H NMR (400 MHz, $CDCl_3$) δ 5.28-5.22 (m, 2 H, H-4 and H-3), 4.96 (d, *J* = 2.8 Hz, 1 H, H-1), 4.11-4.04 (m, 3 H, H-5 and H-6), 3.64-3.59 (m, 1 H, -OCH2-), 3.39-3.34 (m, 1 H, -OCH2-), 3.25 (t, *J* = 6.8 Hz, 1 H, -CH2-N3), 2.09, 2.03, 1.96 (s, 9 H, 3 OAc), 2.07-2.04 (m, 1 H, H-2eq.), 1.84-1.80 (m, 1 H, H-2ax.), 1.63-1.53 (m, 4 H, -CH2CH2CH2-), 1.46-1.39 (m, 2 H, -CH2CH2CH2-) ppm; ^{13}C NMR: δ 170.5-170.1 (C=O), 128.6-128.1 (C=O), 97.6 (α, C-1), 68.0 (α, -O-CH2-), 66.8 (α, C-4), 66.8 (α, C-3), 66.7 (α, C-5), 62.6 (α, C-6), 51.4 (α, -CH2-N3), 30.4 (α, C-2), 29.1 (CH2), 28.7 (CH2), 23.62 (CH2), 20.9-20.8 (3 OAc) ppm; HRMS (ESI, *m/z*) calcd for $C_{17}H_{27}N_3O_8Na$ $[M + Na]^+$ requires 424.1696, found 424.1699.

Cyclohexyl 3,4,6-tri-*O*-acetyl-2-deoxy-D-galactcopyranoside (57). Colorless oil, $[\alpha]^{28}_{D}$ 121.02 (*c* 0.53, $CHCl_3$); ^1H NMR (400 MHz, CDCl3) δ 5.31-5.26 (m, 2 H, H-4 and H-3), 5.14 (d, *J* = 2.8 Hz, 1 H, H-1), 4.21 (t, *J* = 6.4 Hz, 1 H, H-5), 4.20-4.33 (m, 2 H, H-6), 3.54-3.51 (m, 1 H, -O-CH-), 2.11, 2.02, 1.96 (s, 9 H, 3 OAc), 2.09-2.03 (m, 1 H, H-2eq.), 1.85-1.77 (m, 3 H, H-2ax. and Cy), 1.73-1.65 (m, 2 H, Cy), 1.51 (m, 1 H, Cy), 1.46-1.19 (m, 5 H, Cy) ppm; ^{13}C NMR: δ 170.6-170.2 (C=O), 95.7 (α, C-1), 75.6 (α, -O-CH-), 67.0 (α, C-4), 66.8 (α, C-3), 66.6 (α, C-5), 62.8 (α, C-6), 33.5 (CH2), 31.7 (CH2), 31.0 (α, C-2), 25.8 (CH2), 24.6 (CH2), 24.1 (CH2), 20.99-20.81 (OAc) ppm; HRMS (ESI, *m/z*) calcd for $C_{18}H_{28}O_8Na$ $[M + Na]^+$ requires 395.1682, found 395.1687.

2-Adamantyl 3,4,6-tri-*O*-acetyl-2-deoxy-D-galactopyranoside (58). Colorless oil, $[\alpha]^{28}_{D}$ 114.81 (*c* 0.77, $CHCl_3$); ^1H NMR (400 MHz, $CDCl_3$) δ 5.34-5.15 (m, 2 H, H-4 and H-3), 5.14 (d, *J* = 2.8 Hz, 1 H, H-1), 4.21 (t, *J* = 6.4 Hz, 1 H, H-5), 4.09-4.0 (m, 2 H, H-6), 3.70 (br, 1 H, -O-CH-), 2.10, 2.01, 1.90 (s, 9 H, 3 OAc), 2.08-1.96 (m, 1 H, H-2eq.), 1.86 (m, 1 H, H-2ax.), 1.84-1.60 (m, 12 H, adamantanyl), 1.45 (t, *J* = 12.4 Hz, 1 H, adamantanyl) ppm; ^{13}C NMR: δ 170.6-170.3 (C=O), 95.8 (α, C-1), 79.9 (α,

-O-CH-), 67.1 (α, C-4), 67.0 (α, C-3), 66.8 (α, C-5), 62.9 (α, C-6), 37.7 (CH2), 37.0 (CH2), 36.6 (CH2), 33.6 (CH), 32.0 (CH2), 31.7 (CH2), 31.4 (CH), 31.1 (α, C-2), 27.6 (CH), 27.4 (CH), 21.1-20.9 (OAc) ppm; HRMS (ESI, *m/z*) calcd for $C_{22}H_{32}O_8Na$ $[M + Na]^+$ requires 447.1995, found 447.1981.

***O*-[3,4,6-Tri-*O*-acetyl-2-deoxy-D-galactopyranosyl]-N-carbobenzyloxy-L-serine methyl ester (59)**. Colorless oil, $[\alpha]^{28}_D$ 87.46 (*c* 0.54, $CHCl_3$); ^1H NMR (400 MHz, $CDCl_3$) δ 7.40-5.29 (m, 5 H, -ArH), 5.71 (d, *J* = 8.0 Hz, 1 H, NH), 5.28 (s, 1 H, H-4), 5.17 (dt, *J* = 12.4, 2.8 Hz, 1 H, H-3), 5.08 (s, 1 H, PhCH2), 4.95 (d, *J* = 3.2 Hz, 1 H, H-1), 4.53-4.51 (m, 1 H, CH2), 4.10-4.01 (m, 4 H, CH2, C-5, and C-6), 3.89 (br, 2 H, CH and C-6), 3.75 (OMe), 2.09, 2.0, 1.95 (3 OAc), 2.07-2.03 (m, 1 H, H-2eq.), 1.80 (dd, *J* = 12.8, 5.2 Hz, 1 H, H-2ax.) ppm; ^{13}C NMR: δ 170.7-170.2 (C=O), 156.1 (C=O), 136.4 (Ph), 128.7-128.3 (Ph), 98.4 (α, C-1), 68.6 (PhCH2), 67.5 (α, C-4), 67.3 (CH2), 66.7 (α, C-3), 66.1 (α, C-5), 62.6 (α, C-6), 54.5 (α, NCH), 52.9 (α, OMe), 30.1 (α, C-2), 21.0-20.8 (OAc) ppm; HRMS (ESI, *m/z*) calcd for $C_{24}H_{31}O_{12}Na$ $[M + Na]^+$ requires 548.1744, found 548.1748.

***O*-[3,4,6-Tri-*O*-acetyl-2-deoxy-D-galactopyranosyl]-N-carbobenzyloxy-L-threonine methyl ester (60)**. Colorless oil, $[\alpha]^{28}_D$ 65.42 (*c* 0.64, $CHCl_3$); ^1H NMR (400 MHz, $CDCl_3$) δ 7.36-7.30 (m, 5 H, -ArH), 5.45 (d, *J* = 9.6 Hz, 1 H, NH), 5.28 (s, 1 H, H-4), 5.17-5.11 (m, 3 H, H-3 and PhCH2), 4.96 (d, *J* = 3.2 Hz, 1 H, H-1), 4.38-4.30, 4.15 (t, *J* = 6.4 Hz, 1 H, H-5), 4.05-4.02 (m, 2 H, H-6), 3.72 (s, 3 H, OMe), 2.09, 2.01, 1.95 (s, 9 H, 3 OAc), 2.03-1.98 (m, 1 H, H-2eq.), 1.70-1.66 (m, 1 H, H-2ax.), 1.28 (d, *J* = 6.4 Hz, 3 H, Me) ppm; ^{13}C NMR: δ 171.2, 179.5, 170.3, 170.2 (C=O), 156.7 (C=O), 128.7-127.8 (Ph), 99.2 (α, C-1), 98.4 (β, C-1), 81.7 (CH), 79.9 (CH), 77.6 (CH), 76.9 (α, CH), 75.6 (CH2), 73.8 (CH2), 73.0 (CH2), 51.1 (CH), 70.2 (CH), 69.8 (CH2), 67.4 (α, C-4), 67.4 (PHCH2), 66.8 (α, C-3), 66.1 (α, C-5), 62.7 (α, C-6), 58.9 (α, NCH), 55.4 (β, OMe), 52.6 (α, OMe), 30.5 (α, C-2), 20.9-20.8 (OAc), 18.5 (Me) ppm; HRMS (ESI, *m/z*) calcd for $C_{25}H_{33}NO_{12}Na$ $[M + Na]^+$ requires 562.1900, found 562.1912.

6-*O*-[3,4,6-tri-*O*-acetyl-2-deoxy-D-galactopyranosyl]-1,2,3,4-di-*O*-isopropylidene-D-galactopyranose (61). Colorless oil, $[\alpha]^{28}_D$ 25.53 (*c* 0.84, $CHCl_3$); ^1H NMR (400 MHz, $CDCl_3$) δ 5.46 (d, *J* = 5.2 Hz, 1 H, H-1'), 5.39 (br, 1 H, H-4), 5.29-5.25 (m, 1 H, H-3), 5.04 (d, *J* = 3.2 Hz, 1 H, H-1), 4.60 (dd, *J* = 8.4, 2.0 Hz, 1 H), 4.27-4.25 (m, 1 H), 4.19-4.16 (m, 2 H), 4.09-4.02 (m, 2 H), 3.90 (m, 1 H), 3.73-3.69 (m, 1 H), 3.63-3.60 (m, 1 H), 2.07, 1.99, 1.92 (s, 9 H, 3 OAc), 2.05-2.01 (m, 1 H, H-2eq.), 1.87-1.83 (m, 1 H, H-2ax.), 1.49 (s, 3 H, Me), 1.38 (s, 3 H, Me), 1.28 (s, 6 H, 2 Me)

ppm; ^{13}C NMR: δ 171.3-170.2 (C=O), 110.0 (C), 208.8 (C), 97.6 (α, C-1'), 96.5 (α, C-1), 71.7 (CH), 70.9 (CH), 70.6 (CH), 67.1 (α, C-4), 67.0 (α, C-3), 66.3 (α, C-5), 65.7 (α, C-6'), 62.6 (α, C-6), 30.3 (α, C-2), 26.3 (Me), 26.2 (Me), 25.1 (Me), 24.7 (Me), 21.2-21.0 (OAc) ppm; HRMS (ESI, *m/z*) calcd for $C_{24}H_{36}O_{13}Na$ $[M + Na]^+$ requires 555.2054, found 555.2048.

Methyl 2,3,4-tri-*O*-benzyl-6-*O*-(3,4,6-tri-*O*-acetyl-2-deoxy-D-galactopyranosyl)-α-D-glucopyranoside (62). Colorless oil, $[\alpha]^{28}_D$ 85.13 (*c* 0.64, $CHCl_3$); ^1H NMR (400 MHz, $CDCl_3$) δ 7.33-7.25 (m, 15 H, -ArH), 5.25-5.20 (m, 2 H, H-4 and H-3), 5.03 (d, *J* = 2.8 Hz, 1 H, H-1), 4.96 (t, *J* = 11.2 Hz, 2 H, PhCH), 4.80-4.75 (m, 2 H, PhCH), 4.66 (d, *J* = 12.0 Hz, 2 H, PhCH), 4.60-4.57 (m, 2 H, PhCH and H-1'), 4.03-3.93 (m, 4 H), 3.77-3.74 (m, 2 H), 3.62-3.60 (m, 1 H), 3.51 (dd, *J* = 9.6, 3.6 Hz, 1 H), 3.43 (t, *J* = 9.2 Hz, 1 H), 3.36 (s, 3 H, OMe), 2.09, 1.83, 1.88 (s, 9 H, 3 OAc), 2.07-2.04 (m, 1 H, H-2eq.), 1.88-1.84 (dd, *J* = 12.8, 5.6 Hz, 1 H, H-2ax.) ppm; ^{13}C NMR: δ 171.2-170.1 (C=O), 138.8-138.3 (Ph), 128.6-127.6 (Ph), 98.1 (α, C-1), 97.8 (α, C-1'), 82.3 (α, C-3'), 78.1 (α, C-2'), 77.6 (α, C-4'), 75.9 (PhCH2), 75.1 (PhCH2), 73.4 (PhCH2), 70.0 (CH), 66.9 (α, C-4), 66.8 (α, C-3), 66.3 (α, C-5), 66.3 (α, C-6'), 62.6 (α, C-6), 55.3 (OMe), 30.2 (α, C-2), 21.1-20.8 (OAc) ppm; HRMS (ESI, *m/z*) calcd for $C_{28}H_{32}O_6Na$ $[M + Na]^+$ requires 759.2993, found 759.2996.

Methyl 2,3,6-tri-*O*-benzyl-4-*O*-(3,4,6-tri-*O*-acetyl-2-deoxy-α-D-galactopyranosyl)-α-D-glucopyranoside (63). Colorless oil, $[\alpha]^{28}_D$ 63.82 (*c* 0.75, $CHCl_3$);^1H NMR (400 MHz, $CDCl_3$) δ 7.32-7.23 (m, 15 H, -ArH), 5.46 (d, *J* = 2.8 Hz, 1 H, H-1), 5.18-5.13 (m, 2 H, H-4 and H-3), 5.03 (d, *J* = 11.2 Hz, 1 H, PhCH), 4.71 (d, *J* = 11.2 Hz, 1 H, PhCH), 4.64-4.58 (m, 4 H, H-1' and PhCH), 4.52 (d, *J* = 12.4 Hz, 1 H, PhCH), 3.95 (t, *J* = 6.4 Hz, 1 H, H-5), 3.95-3.87 (m, 3 H, C-6 and C-3'), 3.74 (m, 1 H), 3.66-3.64 (m, 3 H, C-5' and C=6'), 3.50 (dd, *J* = 9.6, 3.2 Hz, 1 H, H-2'), 3.39 (s, 3 H, OMe), 2.10, 1.95 (s, 9 H, 3 OAc), 1.91-1.89 (m, 1 H, H-2eq.), 1.71-1.64 (m, 1 H, H-2ax.) ppm; ^{13}C NMR: δ 170.4-170.1 (C=O), 138.8-138.2 (Ph), 128.6-127.7 (Ph), 99.4 (α, C-1'), 97.9 (α, C-1), 82.0 (α, C-3'), 80.4 (α, C-2'), 76.5 (α, C-4'), 75.5 (PhCH2), 73.4 (PhCH2), 69.8 (α, C-5'), 69.4 (α, C-6'), 67.5 (α, C-4), 66.7 (α, C-3), 66.1 (α, C-5), 62.5 (α, C-6), 55.4 (α, OMe), 30.6 (α, C-2), 21.0-20.83 (OAc) ppm; HRMS (ESI, *m/z*) calcd for $C_{40}H_{48}O_{13}Na$ $[M + Na]^+$ requires 759.2993, found 759.2988.

_p_-Tolyl 2,3,4-tri-_O_-benzyl-1-thio-β-D-glucopyranoside (64).[2]

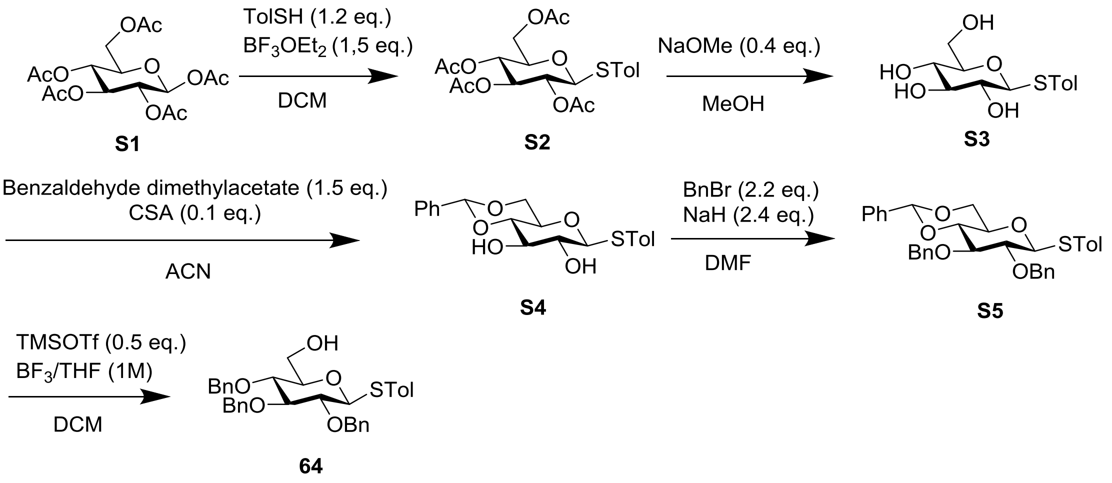

1,2,3,4,6-Penta-*O*-acetyl-D-glucopyranose **S1** (10.0 g, 0.0267 mol) and *p*-thiocresol (4.0 g, 0.032 mol) was dissolved in CH_2Cl_2 (100mL) in a flame dried flask under nitrogen atmosphere. Boron trifluoride diethyl etherate (5.60 mL, 0.04mol) was slowly added at 0 °C. After stirring for 16 hours at room temperature, the reaction mixture was diluted with CH_2Cl_2 (100mL), washed with $NaHCO_3$ (100 mL × 2) and brine (100 mL), dried over anhydrous $MgSO_4$, filtered, and then concentrated under reduced pressure. The crude product was purified by flash column chromatography on silica gel (EtOAc/Hexane = 0/1 to 1/2) to obtain 2,3,4-tri-*O*-acetyl-1-thio-β-D-glucopyranoside **S2**.

To a solution of compound **S2** in methanol (100 mL) was added sodium methoxide (432.0 mg, 0.008mol) at room temperature under ambient atmosphere. After stirring for 4 hours, amberlite IR (120 H^+) acid resin was added protionwise until the solution was neutralized. The mixture was filtered, concentrated under reduced pressure, and then volatiles were removed *in vacuo* to afford tetrol glycoside **S3**.

To a solution of tetrol glucopyranoside **S3** and benzaldehyde dimethyl acetal (6.10 g, 0.040 mol) in dried acetonitrile (100 mL) was added camphorsulfonic acid (0.620 g, 2.670 mmol) in a flame dried flask under nitrogen atmosphere. After stirring for 6 hours at room temperature, the reaction solution was diluted with ethyl acetate (100 mL), washed with $NaHCO_3$ (50 mL × 2) and brine (50 mL), dried over anhydrous $MgSO_4$, filtered, concentrated under reduced pressure, and then volatiles were removed *in vacuo* to obtain 4,6-O-benzylidine-D-glucopyranoside **S4**.

To a solution of 4,6-*O*-benzylidine-D-glucopyranoside **S4** in dried DMF (80 mL) was added benzyl bromide (7.0 mL, 0.059 mol) in a flame dried flask under nitrogen atmosphere. Sodium hydride (60% dispersion in mineral oil, 2.560 g, 0.064 mol) was added portionwise and gradually in the reaction solution at 0 °C. After

stirring for 16 hours at room temperature, the mixture was diluted with ethyl acetate (100 mL), quenched by water (50 mL×2) and washed with brine (50 mL), dried over anhydrous $MgSO_4$, filtered, and then concentrated under reduced pressure. The crude product was purified by flash column chromatography on silica gel (EtOAc/ Hexane= 0/1 to 1/4) and then volatiles were removed *in vacuo* to acquire the benzyl glycoside **S5** as a white solid.

Compound **S5** was dissolved in dried CH_2Cl_2 (50 mL) in a flame dried flask under nitrogen atmosphere. Borane-tetrahydrofurane complex (1M in THF, 130 mL, 0.13 mol) and trimethyl silyltrifluoromethanesulfonate (2.30 mL, 0.013 mol) was added subsequently in the reaction solution at 0 °C After stirring for 6 hours at 0 °C, the reaction mixture was quenched by $NaHCO_3$ (50 mL×3), washed with brine (50 mL), dried over anhydrous $MgSO_4$, filtered, and then concentrated under reduced pressure. The crude product was purified by flash column chromatography on silica gel (EtOAc/ Hexane= 0/1 to 1/2) and then volatiles were removed *in vacuo* to acquire the product (**64**) as white solid. $[\alpha]^{28}_{D}$ 4.93 (*c* 0.58, $CHCl_3$); ^1H NMR (400 MHz, $CDCl_3$) δ 7.42-7.27 (m, 17 H, Ph), 7.11 (d, *J*＝8.0 Hz, 2 H, Ph), 4.93- 4.83 (m, 3 H, PhCH), 4.76 (d, *J*＝10.4 Hz, 1 H, PhCH), 4.61 (d, *J*＝12.4 Hz, 2 H, PhCH and H-1), 3.89-3.83 (m, 1 H, H-6), 3.71 (t, *J*＝9.2 Hz, 1 H, H-3), 3.70-3.65 (m, 1 H, H-6), 3.55 (t, *J*＝9.2 Hz, 1 H, H-4), 3.45 (t, *J*＝9.2 Hz, 1 H, H-2),3.38-3.33 (m, 1 H, H-5), 2.33 (s, 3 H, Me), 1.89 (t, *J*＝6.8 Hz, 1 H, OH) ppm; ^{13}C NMR: δ 138.3-137.8 (C), 132.5 (CH), 129.7 (CH), 129.4 (CH), 128.4-127.6 (CH), 87.7 (CH), 86.5 (CH), 81.0 (CH), 79.2 (CH), 77.6 (CH), 75.6 (CH2), 75.3 (CH2), 75.0 (CH2), 62.0 (CH2), 20.9 (CH3) ppm; HRMS (ESI, *m/z*) calcd for $C_{34}H_{36}O_5NaS$ $[M + Na]^+$ requires 579.2181, found 579.2183.

***p*-Tolyl 2,3,4-tri-*O*-benzyl-6-(3,4,6-tri-*O*-acetyl-2-deoxy-D-glucopyranosyl)-1-thio-β-D-glucopyranoside (65)**. 3,4,6-Tri-*O*-acetyl glucal **1** (55.0 mg, 0.20 mmol), *p*-tolyl-2,3,4-tri-*O*-benzyl-1-thio-β-D-glucopyranoside **64** (170.0 mg, 0.30 mmol), and TPPO (56.0 mg, 0.20 mmol) were dissolved in minimal CH_2Cl_2 in a flame dried flask under ambient atmosphere. After stirring homogeneously for 2 hours at room temperature, the reaction mixture was directly purified by flash column chromatography on silica gel (EtOAc/ Hexane= 0/1 to 1/1) and then volatiles were removed *in vacuo* to acquire *p*-tolyl-2,3,4-tri-*O*-benzyl-6-(3,4,6-tri-*O*-acetyl-2-deoxy-D-glucopyranosyl)-1-thio-β-D-glucopyranoside **65** (96.8%, *α/β* =7/1) as a white solid. α-isomer **(65*α*)**: White solid, $[\alpha]^{28}_{D}$ 48.33 (*c* 0.57, $CHCl_3$); ^1H NMR (400 MHz, $CDCl_3$) δ 7.39-7.34 (m, 5 H, Ph), 7.28 (br, 12 H, Ph), 7.09 (d, *J*＝7.6 Hz, 2 H, Ph), 5.24-5.23 (m, 1 H), 4.97-4.93 (m, 2 H), 4.89-4.85 (m, 2 H), 4.79-4.76 (d, *J*＝10.4 Hz, 1 H), 4.60-4.63 (dd, *J*＝20.0, 11.8 Hz, 1 H), 4.17 (d, *J* ＝8.8 Hz, 1 H), 3.93 (d, *J*＝8.8 Hz, 2 H), 3.76-3.72 (m, 1 H), 3.68-3.63 (m, 2 H),

3.50-3.39 (m, 2 H), 2.28 (s, 3 H, Me), 2.25-2.22 (m, 1 H, H-2eq.), 2.01, 1.98, 1.92 (s, 9 H, 3 OAc), 1.72 (t, *J*＝10.0 Hz, 1 H, H-2ax.) ppm; ^{13}C NMR: δ 170.9-170.0 (C=O), 138.5-137.89 (Ph), 132.5 (Ph), 130.1 (Ph), 28.6-127.8 (Ph), 97.6 (C-1), 88.0 (CH), 86.9 (CH), 81.1 (CH), 78.4 (CH), 78.0 (CH), 76.0 (CH2), 75.6 (CH2), 75.1 (CH2), 69.4 (CH), 69.3 (CH), 68.1 (CH2), 62.4 (CH2), 35.1 (CH2), 21.4-20.8 (OAc) ppm; HRMS (ESI, *m/z*) calcd for $C_{46}H_{52}O_{12}NaS$ $[M + Na]^+$ requires 851.3077, found 851.3065. β-isomer **(65β)**: White solid, $[\alpha]^{28}_{D}$ 7.27 (*c* 0.59, $CHCl_3$); ^{1}H NMR (400 MHz, $CDCl_3$) δ 7.43 (d, *J*＝8.0 Hz, 2 H, Ph), 7.38 (d, *J*＝6.8 Hz, 2 H, Ph), 7.33-7.23 (m, 13 H, Ph), 7.09 (d, *J*＝8.0 Hz, 2 H, Ph), 4.99-4.88 (m , 4 H), 4.82 (t, *J*＝10.0 Hz, 2 H), 4.72 (d, *J*＝10.4 Hz, 1 H), 3.63 (d, *J*＝10.4 Hz, 1 H), 4.58 (d, *J*＝9.2 Hz, 1 H), 4.46 (d, *J*＝8.4 Hz, 1 H), 4.24 (dd, *J*＝12.0, 4.8 Hz, 1 H), 4.08 (d, *J*＝11.6 Hz, 2 H), 3.69 (t, *J*＝8.8 Hz, 1 H), 3.60 (dd, *J*＝11.2, 6.8 Hz, 1 H), 3.51-3.47 (m, 2 H), 3.47-3.38 (m, 2 H), 2.31 (s, 3 H, Me), 2.16 (dd, *J*＝12.8, 2.4 Hz, 1 H, H-2eq.), 2.02 (br, 9 H, 3 OAc), 2.04-2.02 (m, 1 H, H-2ax.) ppm; ^{13}C NMR: δ 171.0-170.0 (C=O), 138.5-138.1 (Ph), 132.3 (Ph), 129.9 (Ph), 128.6-127.8(Ph), 100.0 (CH), 88.0 (CH), 86.9 (CH), 81.1 (CH), 79.0 (CH), 78.2 (CH), 76.0 (CH2), 75.6 (CH2), 75.1 (CH2), 72.0 (CH), 70.7 (CH), 69.4 (CH), 68.6 (CH2), 62.6 (CH2), 36.3 (CH2), 21.2-20.9 (OAc) ppm; HRMS (ESI, *m/z*) calcd for $C_{46}H_{52}O_{12}NaS$ $[M + Na]^+$ requires 851.3077, found 851.3065.

Methyl 2,3,4-tri-*O*-benzyl-6-*O*-(2,3,4-tri-*O*-benzyl-6-*O*-(3,4,6-tri-*O*-acetyl-2-deoxy-α-D-glucopyranosyl)-D-glucopyranosyl)-α-D-glucopyranoside (66). A suspension of disaccharide **65α** (50.0 mg, 0.060 mmol), activated molecular sieves (4 Å, 300 mg), and AgOTf (20.0 mg, 0.072 mmol) in dried CH_2Cl_2 (0.5 mL) was stirred at −78 °C under nitrogen atmosphere for 1 hour. *p*-Toluenesulfenyl chloride (10.0 μL, 0.072mmol) was added to the reaction mixture at −78 °C. After the reaction mixture was stirred for 15 min at –78 °C, monosaccharide acceptor **23** (34.0 mg, 0.072 mmol) in dried CH_2Cl_2 (0.5 mL) was injected in the reaction mixture and stirred for 4 hours at −78 °C. Upon completion of the reaction, the reaction soluiton was quenched with Et_3N and filtered through a short pad of Celite®. The filtrate was evaporated *in vacuo* to furnish the crude product. Crude was purified by flash column chromatography (EtOAc/ Hexane= 0/1 to 1/1) on silica gel and then volatiles were removed *in vacuo* to give desired products **66** (71%, *α*/*β* =1/2). α-isomer **(66α)**: Colorless oil, $[\alpha]^{28}_{D}$ 29.06 (*c* 0.97, $CHCl_3$); ^{1}H NMR (400 MHz, $CDCl_3$) δ 7.33-7.25 (m, 26 H, Ph), 5.24-7.14 (m, 4 H, Ph), 5.29-5.22 (m, 1 H), 5.02 (d, *J*＝2.8 Hz, 1 H), 4.99 (s, 1 H), 4.96-4.89 (m, 5 H), 4.78-4.73 (m, 4 H), 4.68-4.60 (m, 4 H), 4.48 (d, *J*＝11.2 Hz, 1 H), 4.28 (d, *J*＝8.0 Hz, 1 H), 4.19 (dd, 1 H), 4.11 (d, *J*＝8.0 Hz, 1 H), 3.97 (t, *J*＝9.6 Hz, 1 H), 3.94 (dd, *J*＝2.0 Hz, 1 H), 3.88-3.85 (m, 1 H), 3.78-3.75 (m, 2 H), 3.68-3.43 (m,

8 H), 3.32 (s, 3 H, 3 OMe), 2.25 (dd, J = 12.4, 2.0 Hz, 1H, H-2eq), 2.03, 2.0, 1.96 (3 OAc), 1.76 (ddd, J = 15.6, 12.8, 4.0 Hz, 1H, H-2ax). ^{13}C NMR: δ 170.9-170.0 (C=O), 138.6-138.4 (Ph), 128.6-127.6 (Ph), 104.1 (CH), 98.4 (CH), 97.7 (CH), 85.0 (CH), 82.3 (CH), 82.2 (CH), 80.0 (CH), 78.1 (CH), 77.8 (CH), 75.9 (CH2), 75.8 (CH2), 75.2 (CH2), 75.1 (CH2), 74.8 (CH), 73,6 (CH2), 70.1 (CH), 69.5 (CH), 69.2 (CH), 68.6 (CH2), 68.2 (CH), 66.0 (CH2), 62.4 (CH2), 55.5 (CH3), 35.1 (CH2), 29.9 (CH2), 21.1-20.9 (OAc). HRMS (ESI, *m/z*) calcd for $C_{67}H_{76}O_{18}Na$ $[M + Na]^+$ requires 1191.4929, found 1191.4934. β-isomer **(66β):** Colorless oil, $[\alpha]^{28}_{D}$ 103.44 (*c* 0.44, $CHCl_3$); ^{1}H NMR (400 MHz, $CDCl_3$) δ 7.33-7.22 (m, 30 H, -ArH), 5.28-5.21 (m, 1 H), 4.99-4.91 (m, 8 H), 4.81-4.72 (m, 4 H), 4.69 (s, 2 H), 4.64-4.55 (m, 4 H), 4.0 (dd, J= 12.0, 4.0 Hz, 1 H), 3.86 (dd, J=12.4, 1.6 Hz, 1 H), 3.82-3.77(m, 4 H), 3.69-3.66 (m, 3 H), 3.53-3.49 (m, 3 H), 3.43 (dd, J=9.6, 3.6 Hz, 1 H), 3.36 (s, 3 H, OMe), 2.21 (dd, J = 12.4, 2.0 Hz, 1H, H-2eq), 2.01, 1.99, 1.98 (s, 9 H, 3 OAc), 1.75-1.69 (m 1H, H-2ax) ppm; ^{13}C NMR: δ 170.9-170.0 (C=O), 139.0-138.4 (Ph), 128.6-127.4 (Ph), 98.2(CH), 97.4 (CH), 97.2 (CH), 82.3 (CH), 81.9 (CH), 80.5 (CH), 80.4 (CH), 78.0 (CH), 77.8 (CH), 75.9 (CH2), 75. 7 (CH2), 75.1 (CH2), 74.9 (CH2), 73.6 (CH2), 72.6 (CH2), 70.6 (CH), 70.1 (CH), 69.5 (CH), 69.2 (CH), 68.1 (CH), 66.2 (CH2), 66.1 (CH2), 62.3 (CH2), 55.4 (CH3), 35.0 (CH2), 21.1-20.9 (OAc) ppm; HRMS (ESI, *m/z*) calcd for $C_{67}H_{76}O_{18}Na$ $[M + Na]^+$ requires 1191.4929, found 1191.4934.

----

1. C.-S. Chao, C.-Y. Lin, S. Mulani, W.-C. Hung, K.-K. T. Mong, *Chem. Eur. J.* **2011**, *17*, 12193 – 12202.

2. R. R. France, R. G. Compton, B. G. Davis, A. J. Fairbanks, N. V. Rees, J. D. Wadhawan, *Org. Biomol. Chem.*, **2004**, *2*, 2195–2202.

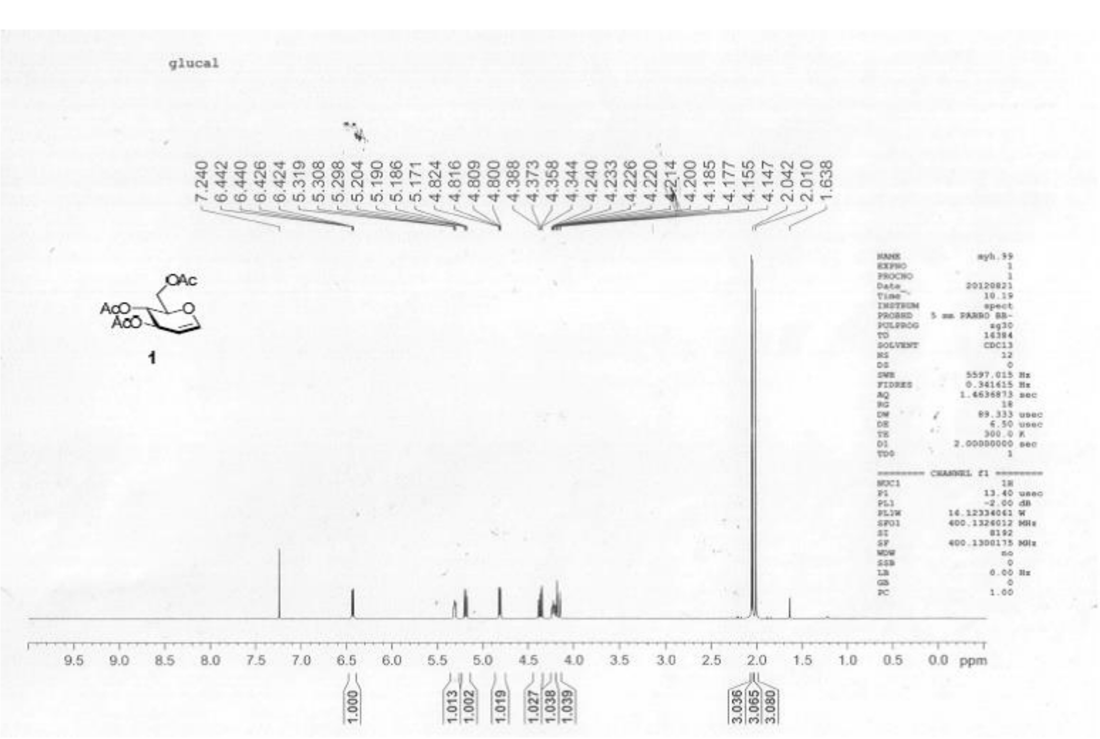

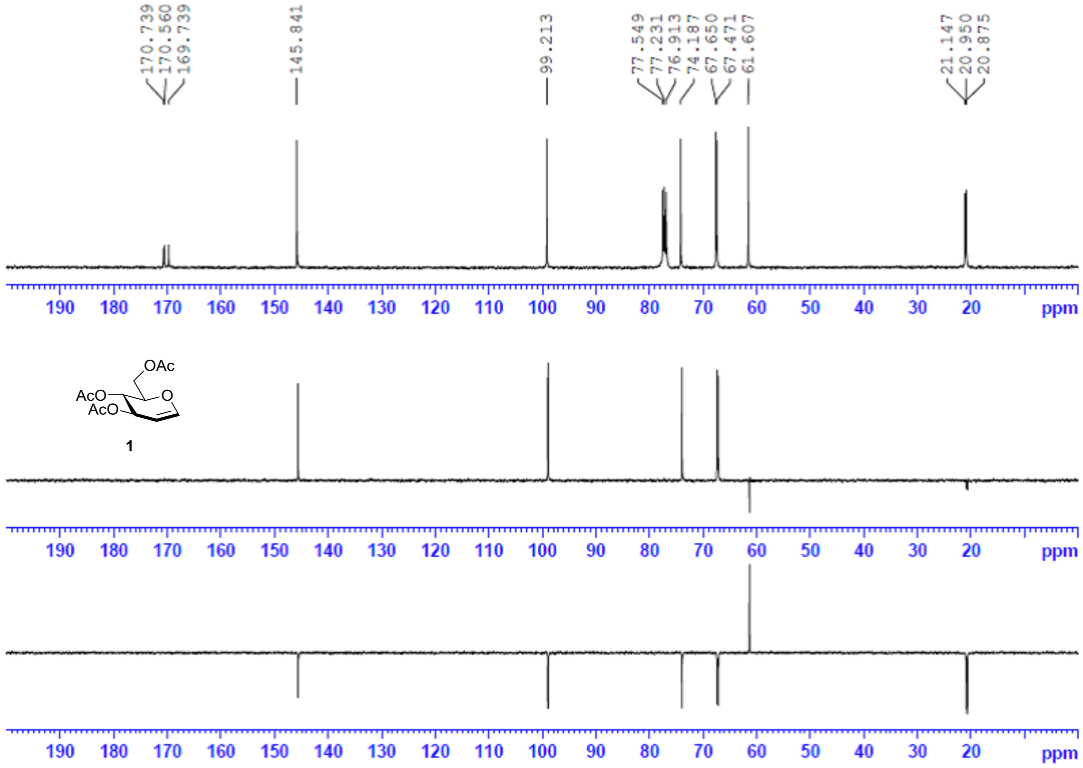
170.739
170.560
169.739
145.841
99.213
77.549
77.231
76.913
74.187
67.650
67.471
61.607
21.147
20.950
20.875
190 180 170 160 150 140 130 120 110 100 90 80 70 60 50 40 30 20 ppm
OAc
AcO
AcO
O
1
190 180 170 160 150 140 130 120 110 100 90 80 70 60 50 40 30 20 ppm
190 180 170 160 150 140 130 120 110 100 90 80 70 60 50 40 30 20 ppm

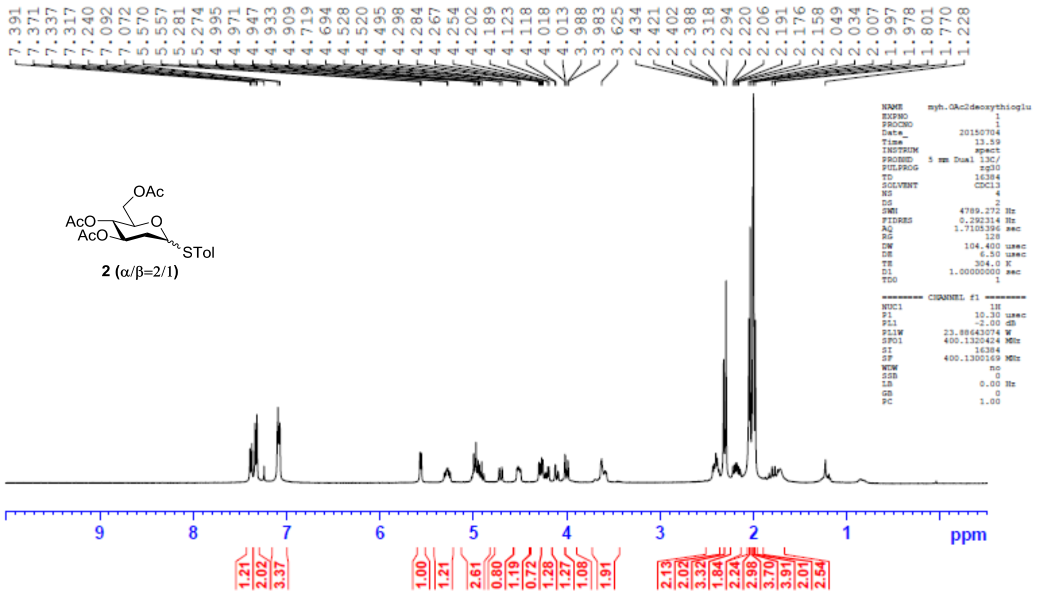
7.391
7.371
7.337
7.317
7.240
7.092
7.072
5.570
5.557
5.281
5.274
4.995
4.971
4.947
4.933
4.909
4.719
4.694
4.528
4.520
4.495
4.298
4.284
4.267
4.254
4.202
4.189
4.123
4.118
4.018
4.013
3.988
3.983
3.625
2.434
2.421
2.402
2.388
2.318
2.294
2.220
2.206
2.191
2.176
2.158
2.049
2.034
2.007
1.997
1.978
1.801
1.770
1.228
OAc
AcO
AcO
O
STol
2 (α/β=2/1)
9 8 7 6 5 4 3 2 1 ppm
1.21
2.02
3.37
1.00
1.21
2.61
0.80
1.19
0.72
1.28
1.27
1.08
1.91
2.13
2.02
3.32
1.84
2.24
2.98
3.70
3.91
2.01
2.54

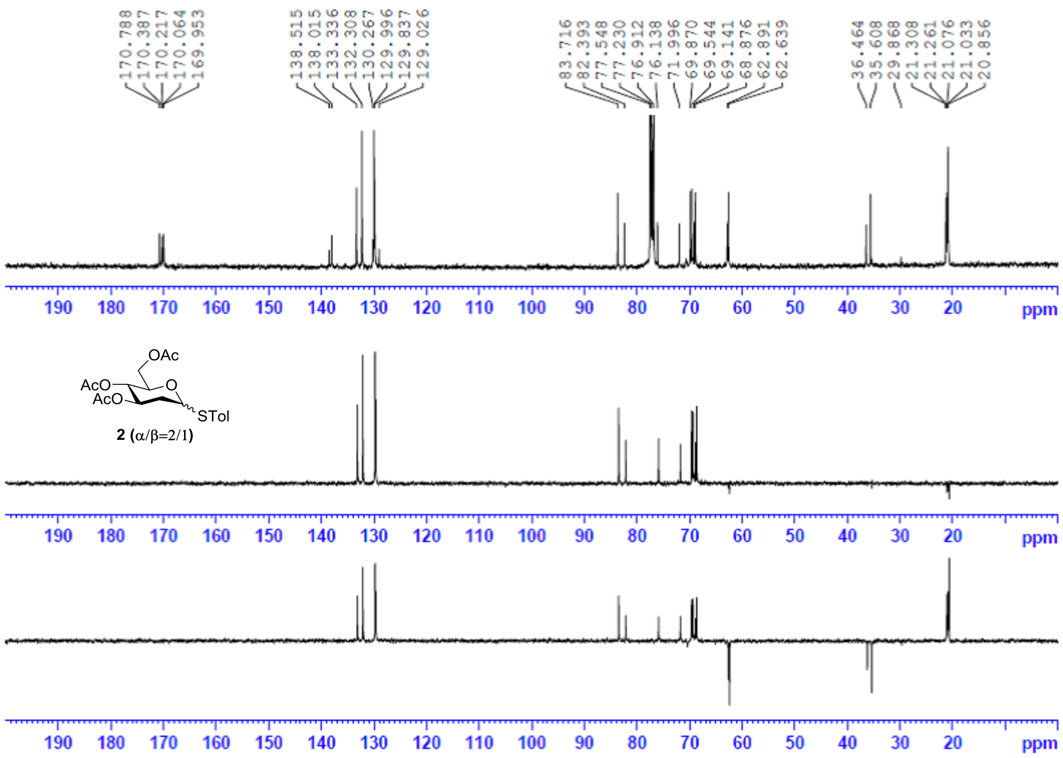

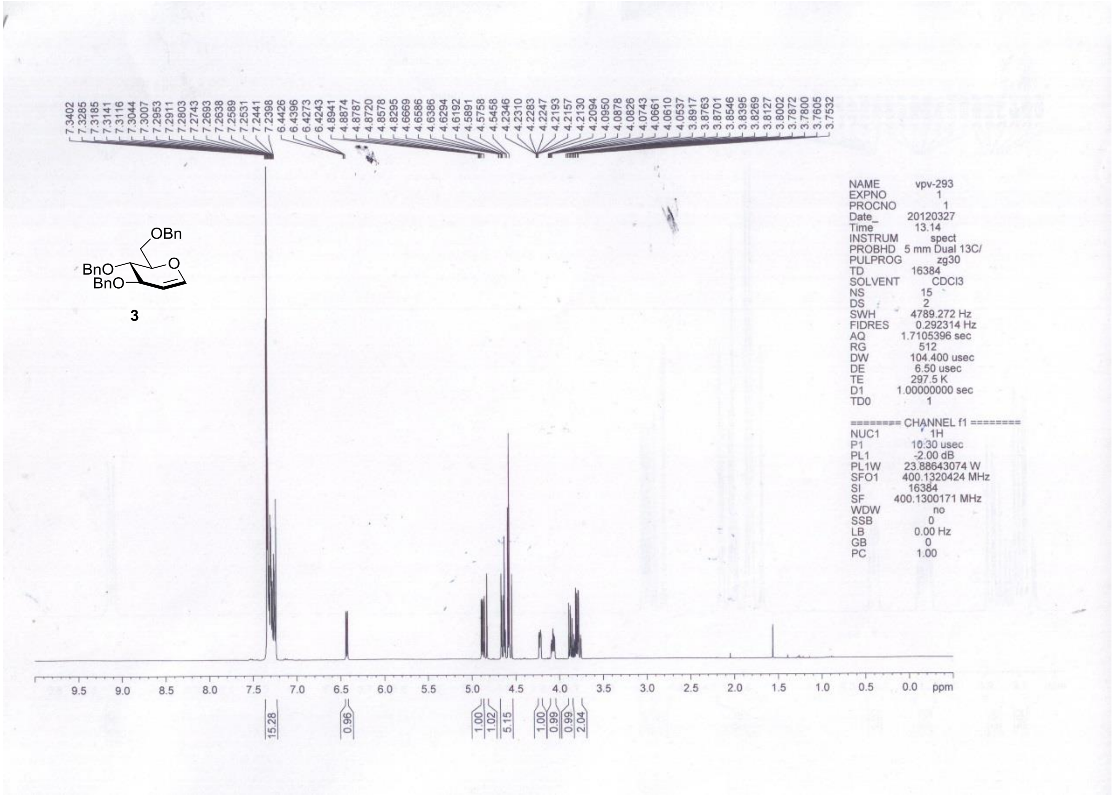

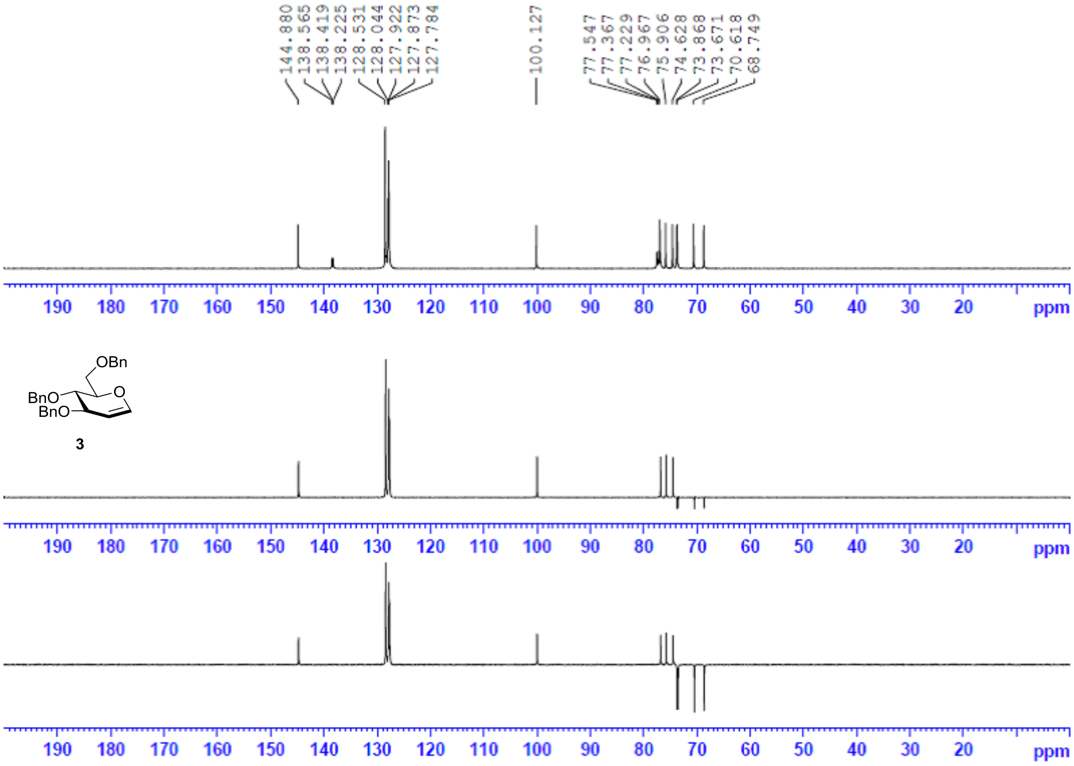
144.880
138.565
138.419
138.225
128.531
128.044
127.922
127.873
127.784
100.127
77.547
77.367
77.229
76.967
75.906
74.628
73.868
73.671
70.618
68.749
ppm
OBn
BnO
BnO
O

3

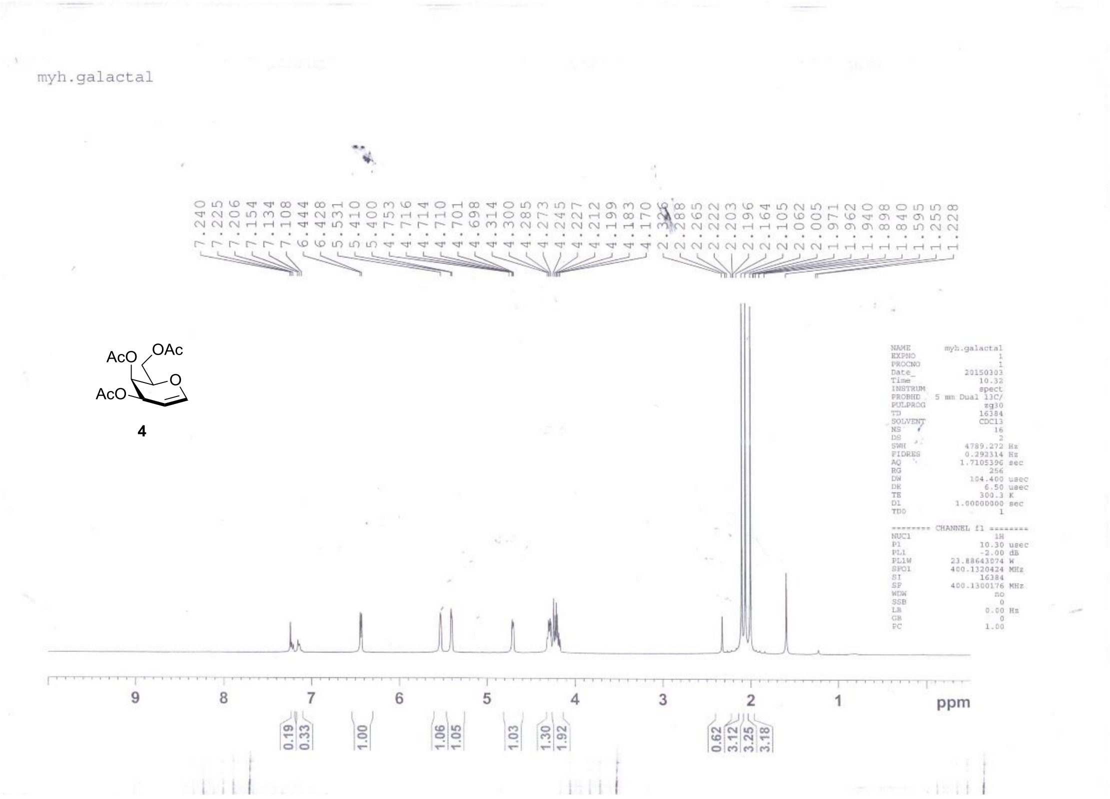
myh.galactal
AcO
OAc
AcO
O
ppm

4

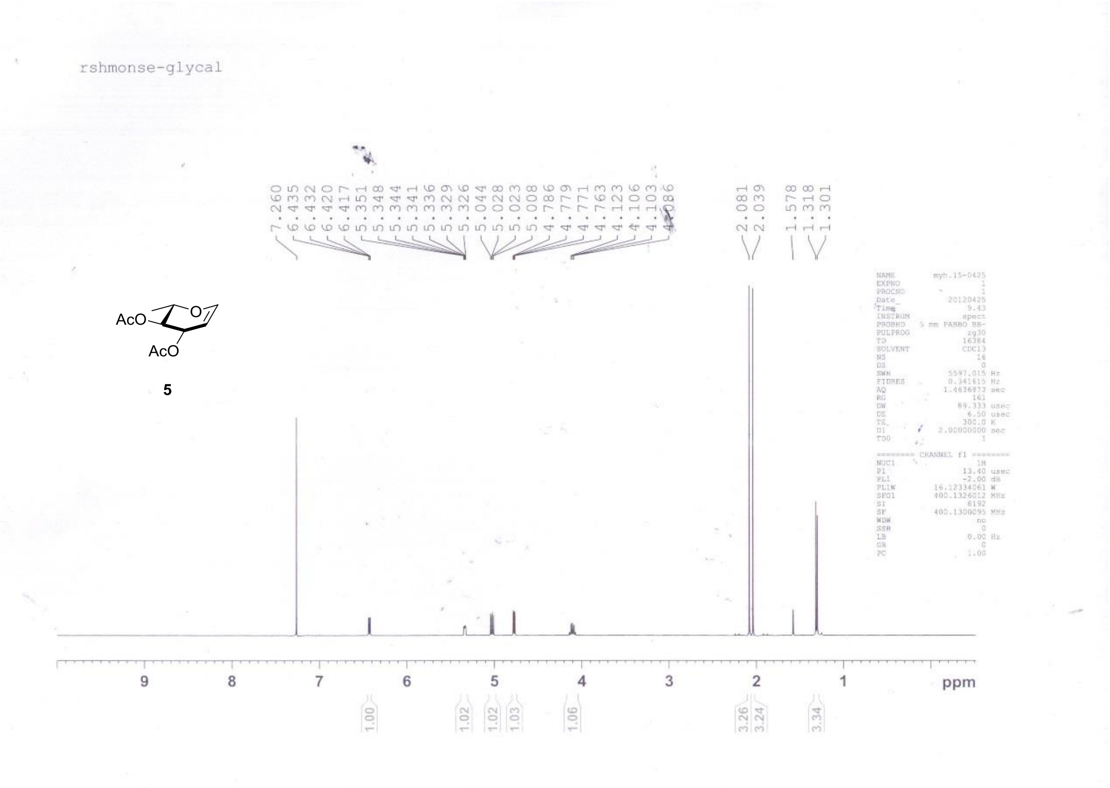
rshmonse-glycal
7.260
6.435
6.432
6.420
6.417
5.351
5.348
5.344
5.341
5.336
5.329
5.326
5.044
5.028
5.023
5.008
4.786
4.779
4.771
4.763
4.123
4.106
4.103
4.086
2.081
2.039
1.578
1.318
1.301
AcO
O
AcO
5
9 8 7 6 5 4 3 2 1 ppm
1.00
1.02
1.02
1.03
1.06
3.26
3.24
3.34

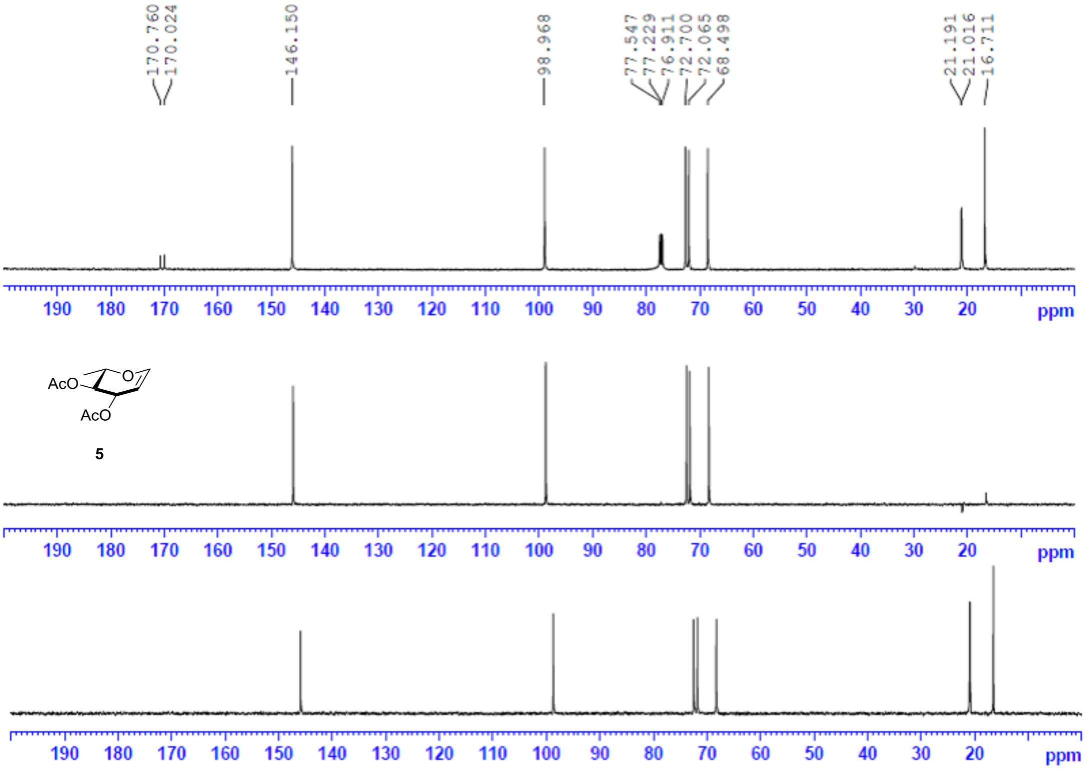
170.760
170.024
146.150
98.968
77.547
77.229
76.911
72.700
72.065
68.498
21.191
21.016
16.711
190 180 170 160 150 140 130 120 110 100 90 80 70 60 50 40 30 20 ppm
AcO
O
AcO
5
190 180 170 160 150 140 130 120 110 100 90 80 70 60 50 40 30 20 ppm
190 180 170 160 150 140 130 120 110 100 90 80 70 60 50 40 30 20 ppm

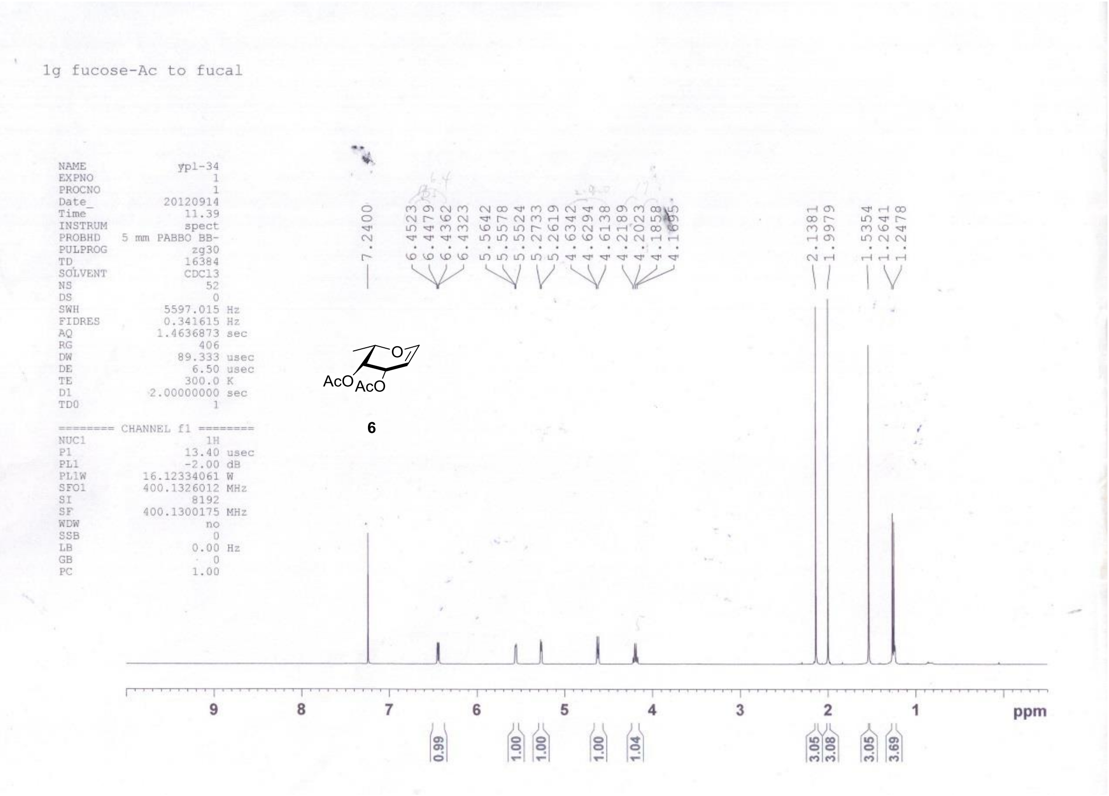

1g fucose-Ac to fucal
NAME ypl-34
EXPNO 1
PROCNO 1
Date_ 20120914
Time 11.39
INSTRUM spect
PROBHD 5 mm PABBO BB-
PULPROG zg30
TD 16384
SOLVENT CDCl3
NS 52
DS 0
SWH 5597.015 Hz
FIDRES 0.341615 Hz
AQ 1.4636873 sec
RG 406
DW 89.333 usec
DE 6.50 usec
TE 300.0 K
D1 2.00000000 sec
TD0 1
======== CHANNEL f1 ========
NUC1 1H
P1 13.40 usec
PL1 -2.00 dB
PL1W 16.12334061 W
SFO1 400.1326012 MHz
SI 8192
SF 400.1300175 MHz
WDW no
SSB 0
LB 0.00 Hz
GB 0
PC 1.00
7.2400
6.4523
6.4479
6.4362
6.4323
5.5642
5.5578
5.5524
5.2733
5.2619
4.6342
4.6294
4.6138
4.2189
4.2023
4.1858
2.1381
1.9979
1.5354
1.2641
1.2478
AcO
AcO
O
6
9 8 7 6 5 4 3 2 1 ppm
0.99
1.00
1.00
1.00
1.04
3.05
3.08
3.05
3.69

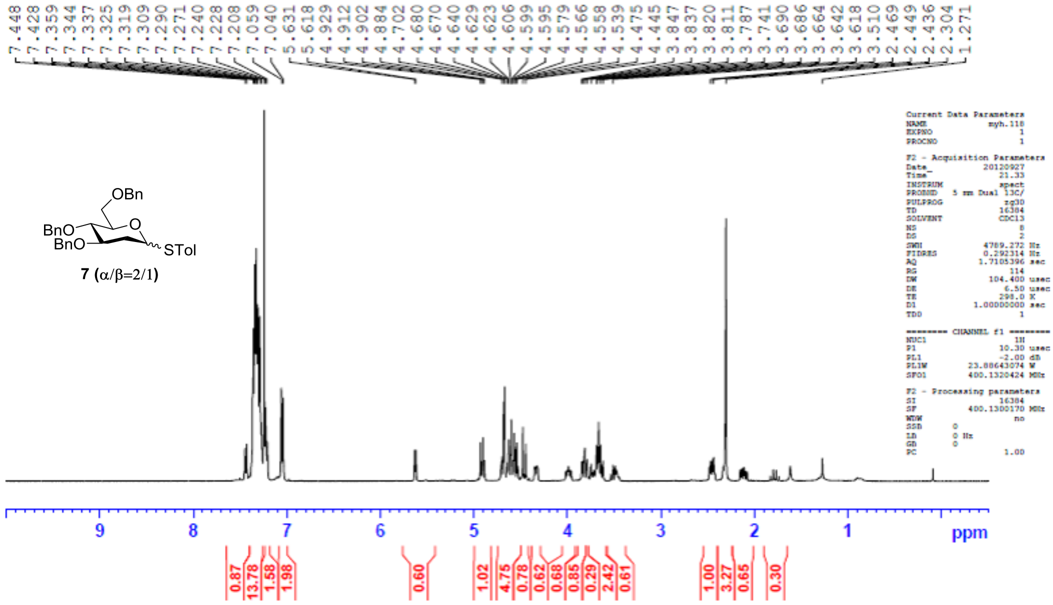

7.448
7.428
7.359
7.344
7.337
7.325
7.319
7.309
7.290
7.271
7.240
7.228
7.208
7.059
7.040
5.631
5.618
4.929
4.912
4.902
4.884
4.702
4.680
4.670
4.640
4.629
4.623
4.606
4.599
4.595
4.579
4.566
4.558
4.539
4.475
4.445
3.847
3.837
3.820
3.811
3.787
3.741
3.690
3.686
3.664
3.642
3.618
3.510
2.469
2.449
2.436
2.304
1.271
OBn
BnO
BnO
O
STol
7 (α/β=2/1)
Current Data Parameters
NAME myh.118
Date_ 20120927
Time 21.33
SOLVENT CDCl3
SF 400.1300170 MHz
9 8 7 6 5 4 3 2 1 ppm
0.87
13.78
1.58
1.98
0.60
1.02
4.75
0.78
0.62
0.68
0.85
0.29
2.42
0.61
1.00
3.27
0.65
0.30

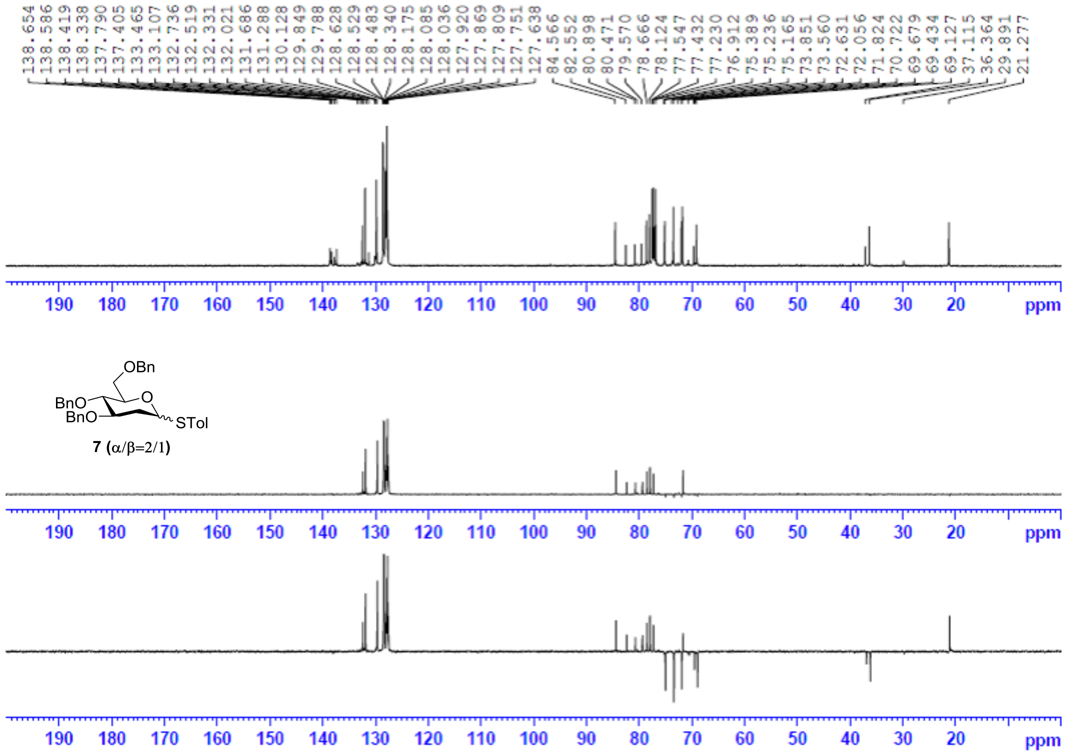
138.654
138.586
138.419
138.338
137.790
137.405
133.465
133.107
132.736
132.519
132.331
132.021
131.686
131.288
130.128
129.849
129.788
128.628
128.529
128.483
128.340
128.175
128.085
128.036
127.920
127.869
127.809
127.751
127.638
84.566
82.552
80.898
80.471
79.570
78.666
78.124
77.547
77.432
77.230
76.912
75.389
75.236
75.165
73.851
73.560
72.631
72.056
71.824
70.722
69.679
69.434
69.127
37.115
36.364
29.891
21.277
190 180 170 160 150 140 130 120 110 100 90 80 70 60 50 40 30 20 ppm

7 (α/β=2/1)

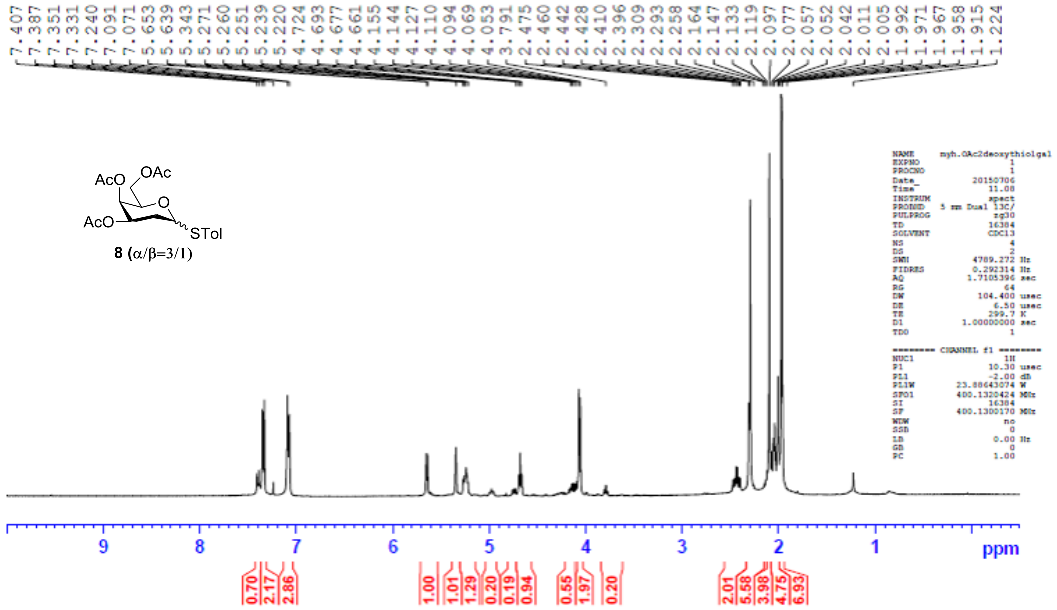
7.407
7.387
7.351
7.331
7.240
7.091
7.071
5.653
5.639
5.343
5.271
5.260
5.251
5.239
5.220
4.724
4.693
4.677
4.661
4.155
4.144
4.127
4.110
4.094
4.069
4.053
3.791
2.475
2.460
2.442
2.428
2.410
2.396
2.309
2.293
2.258
2.164
2.147
2.133
2.119
2.097
2.077
2.057
2.052
2.042
2.011
2.005
1.992
1.971
1.967
1.958
1.915
1.224
NAME myh.OAc2deoxythiolgal
SOLVENT CDCl3
9 8 7 6 5 4 3 2 1 ppm
0.70 2.17 2.86 1.00 1.01 1.29 0.20 0.19 0.94 0.55 1.97 0.20 2.01 5.58 3.98 4.75 6.93

8 (α/β=3/1)

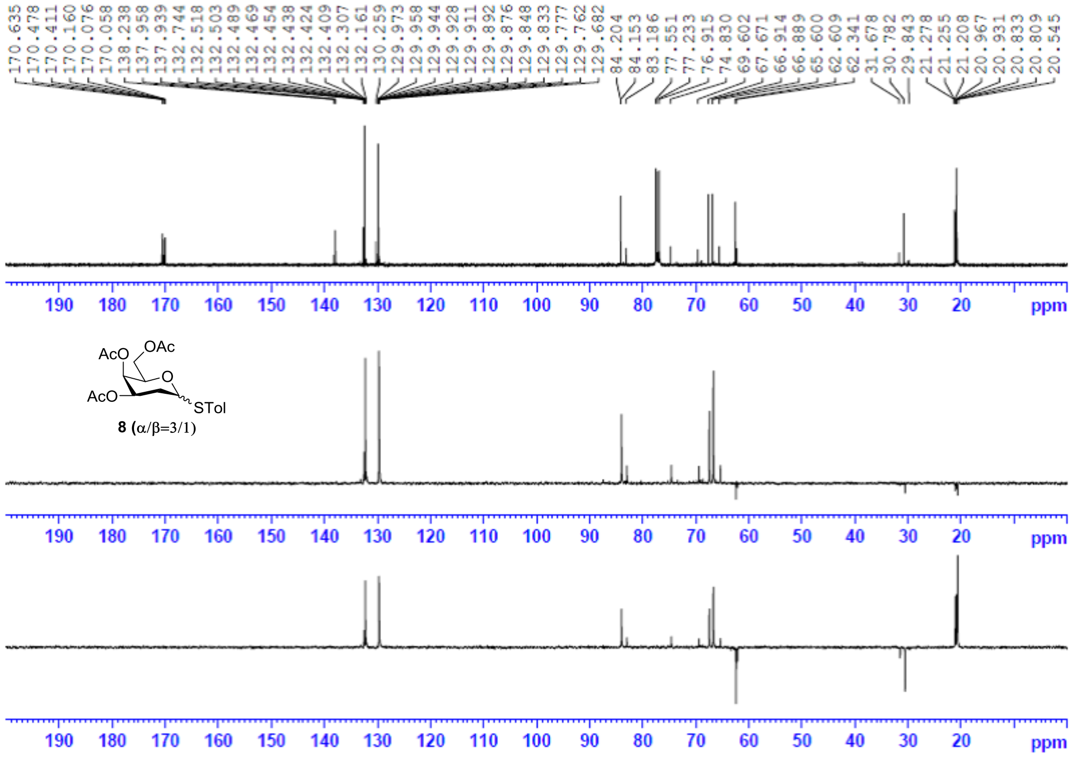
170.635 170.478 170.411 170.160 170.076 170.058 138.238 137.958 137.939 132.744 132.518 132.503 132.489 132.469 132.454 132.438 132.424 132.409 132.307 132.161 130.259 129.973 129.958 129.944 129.928 129.911 129.892 129.876 129.848 129.833 129.777 129.762 129.682 84.204 84.153 83.186 77.551 77.233 76.915 74.830 69.602 67.671 66.914 66.889 65.600 65.609 62.341 31.678 30.782 29.843 21.278 21.255 21.208 20.967 20.931 20.833 20.545
AcO
OAc
O
AcO
STol
8 (α/β=3/1)
190 180 170 160 150 140 130 120 110 100 90 80 70 60 50 40 30 20 ppm

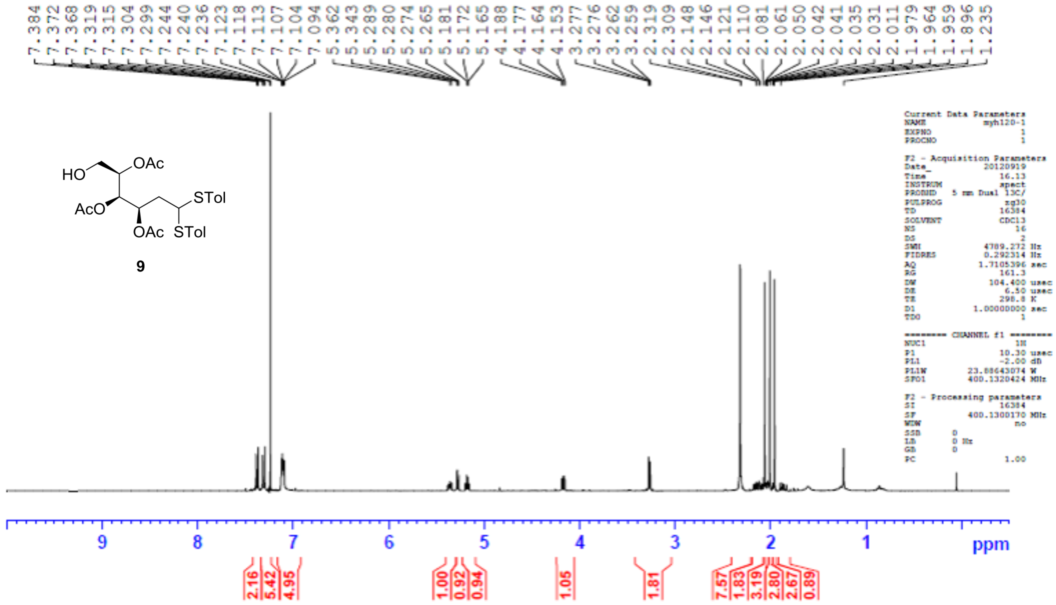
7.384 7.372 7.368 7.319 7.315 7.304 7.299 7.244 7.240 7.236 7.123 7.118 7.113 7.107 7.104 7.094 5.362 5.343 5.289 5.280 5.274 5.265 5.181 5.172 5.165 4.188 4.177 4.164 4.153 3.277 3.276 3.262 3.259 2.319 2.309 2.148 2.146 2.121 2.110 2.081 2.061 2.050 2.042 2.041 2.035 2.031 2.011 1.979 1.964 1.959 1.896 1.235
HO
OAc
AcO
STol
OAc
STol
9
9 8 7 6 5 4 3 2 1 ppm
2.16 5.42 4.95 1.00 0.92 0.94 1.05 1.81 7.57 1.83 3.19 2.80 2.67 0.89

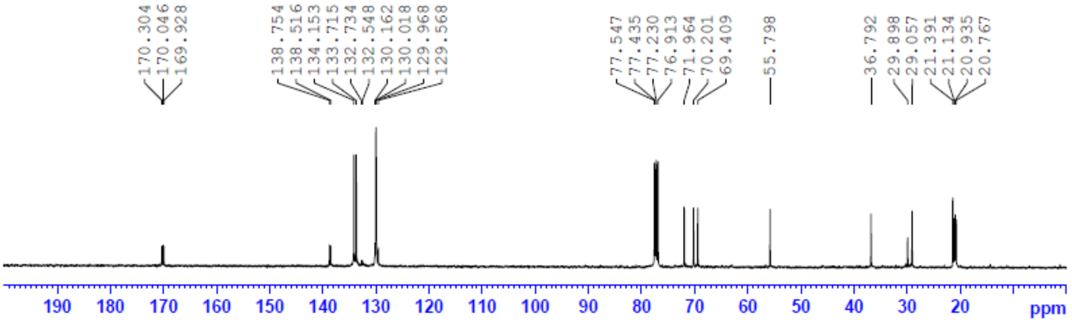
170.304
170.046
169.928
138.754
138.516
134.153
133.715
132.734
132.548
130.162
130.018
129.968
129.568
77.547
77.435
77.230
76.913
71.964
70.201
69.409
55.798
36.792
29.898
29.057
21.391
21.134
20.935
20.767
190 180 170 160 150 140 130 120 110 100 90 80 70 60 50 40 30 20 ppm

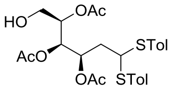
HO
OAc
AcO
STol
OAc
STol

9

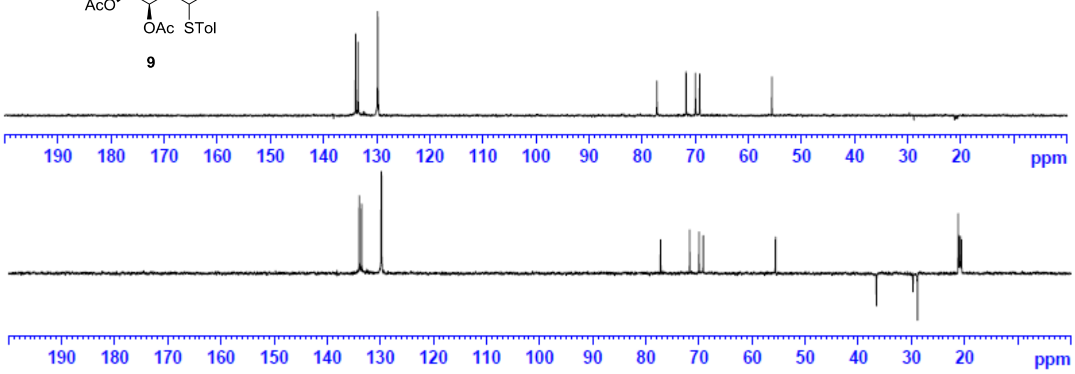
190 180 170 160 150 140 130 120 110 100 90 80 70 60 50 40 30 20 ppm
190 180 170 160 150 140 130 120 110 100 90 80 70 60 50 40 30 20 ppm

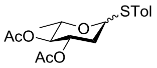
STol
AcO
O
AcO

10 (α/β=2/1)

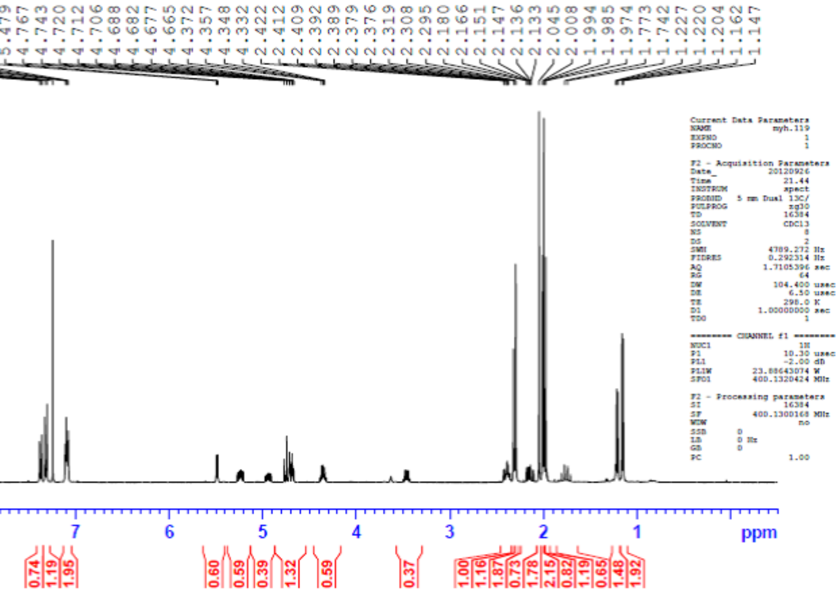
7.381
7.364
7.360
7.323
7.303
7.240
7.107
7.095
7.088
7.075
5.493
5.479
4.767
4.743
4.720
4.712
4.706
4.688
4.682
4.677
4.665
4.372
4.357
4.348
4.332
4.322
2.422
2.412
2.409
2.392
2.389
2.379
2.376
2.319
2.308
2.295
2.180
2.166
2.151
2.147
2.136
2.133
2.045
2.008
1.994
1.985
1.974
1.773
1.742
1.227
1.220
1.204
1.162
1.147
9 8 7 6 5 4 3 2 1 ppm
0.74
1.19
1.95
0.60
0.59
0.39
1.32
0.59
0.37
1.00
1.16
1.87
0.73
1.78
2.15
0.82
1.19
0.65
1.48
1.92

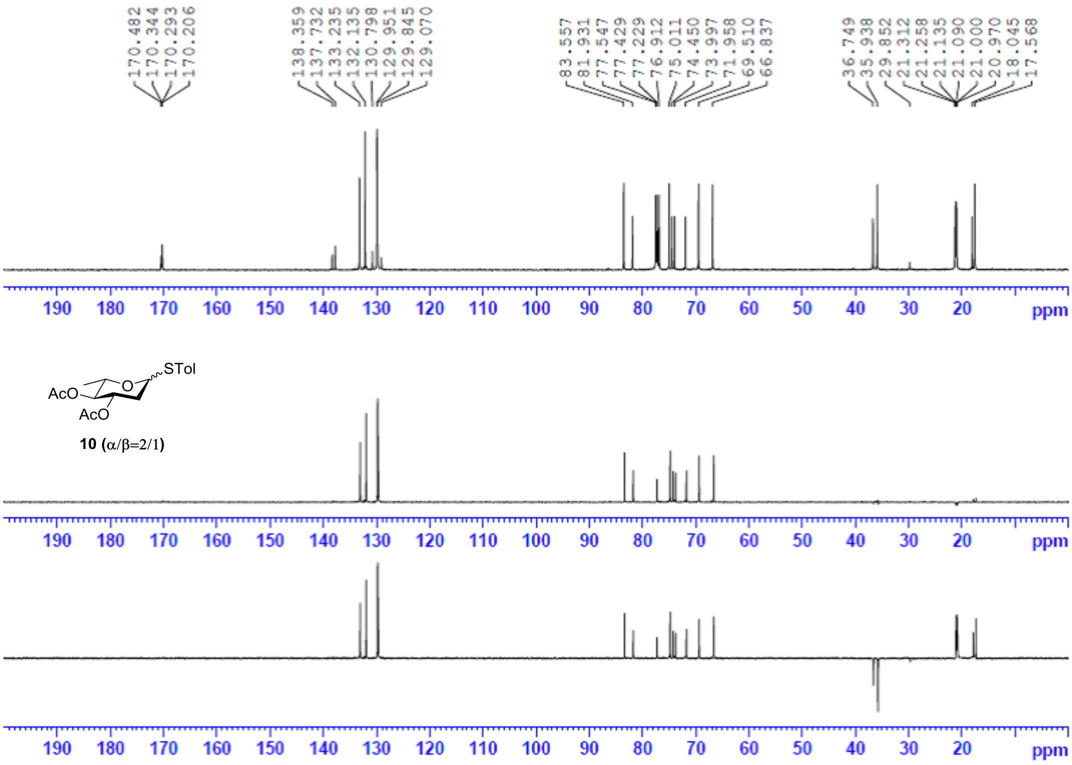

10 (α/β=2/1)

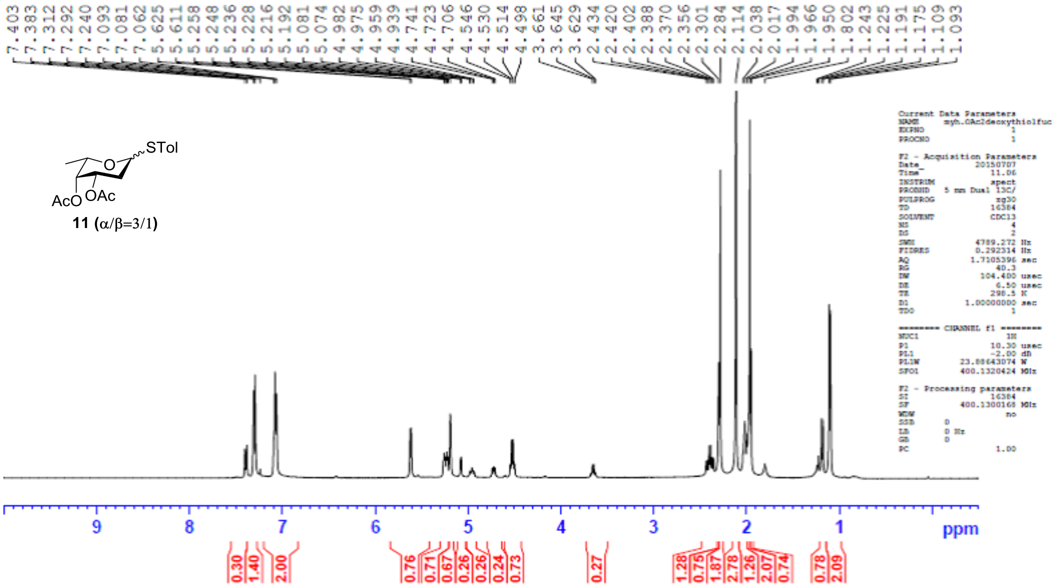

11 (α/β=3/1)

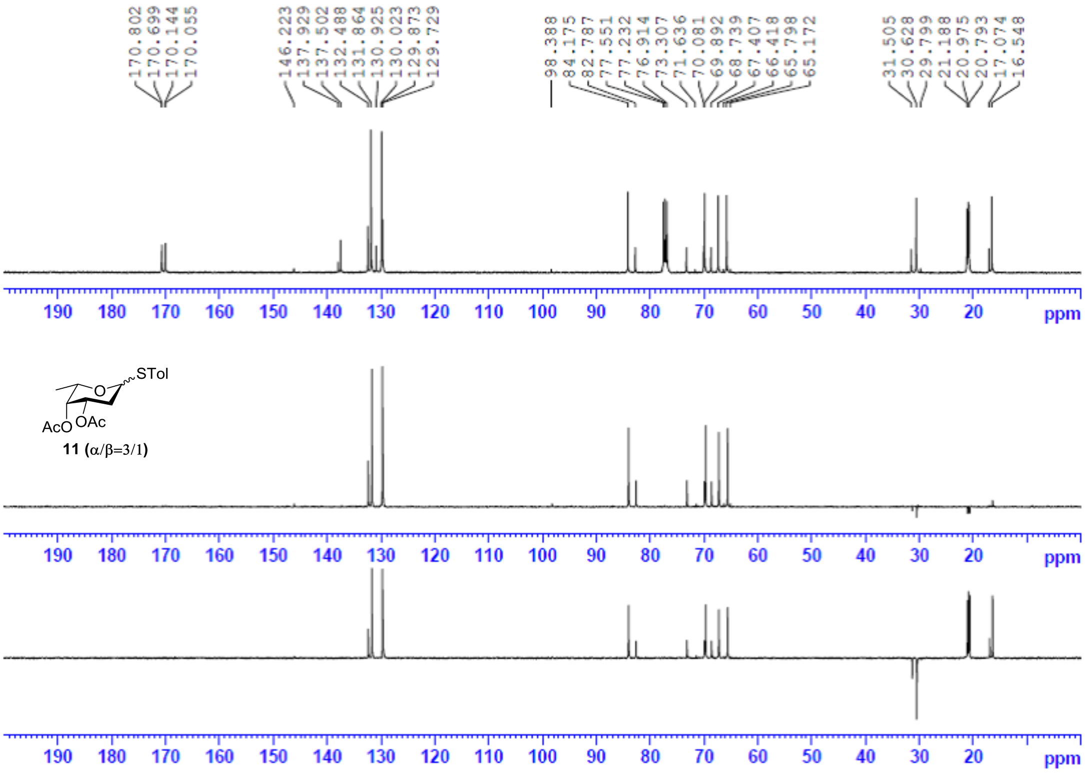
170.802
170.699
170.144
170.055
146.223
137.929
137.502
132.488
131.864
130.925
130.023
129.873
129.729
98.388
84.175
82.787
77.551
77.232
76.914
73.307
71.636
70.081
69.892
68.739
67.407
66.418
65.798
65.172
31.505
30.628
29.799
21.188
20.975
20.793
17.074
16.548
190 180 170 160 150 140 130 120 110 100 90 80 70 60 50 40 30 20 ppm
STol
O
AcO OAc
11 (α/β=3/1)

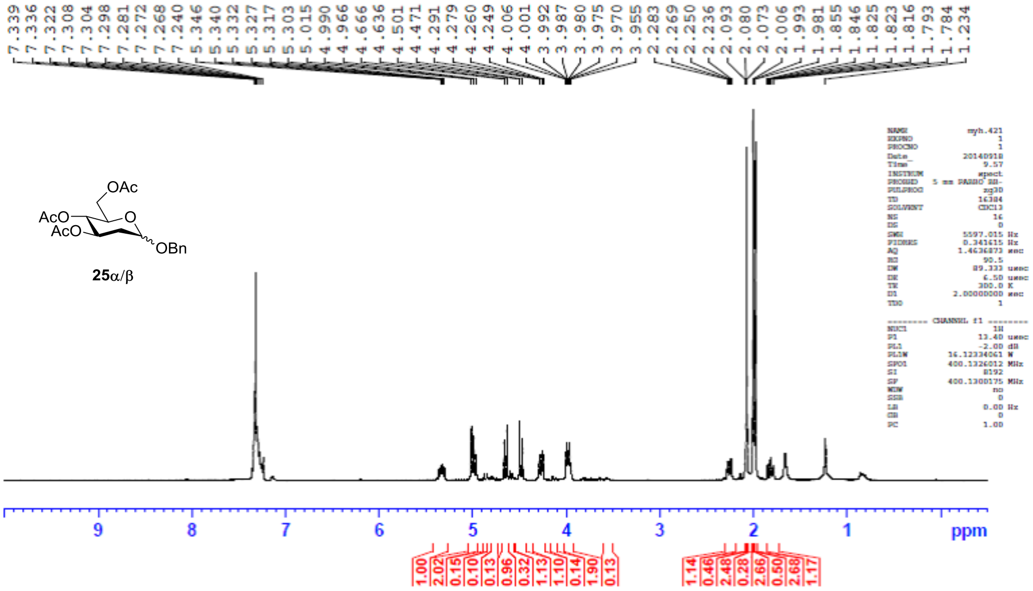
7.339
7.336
7.322
7.308
7.304
7.298
7.281
7.272
7.268
7.240
5.346
5.340
5.332
5.327
5.317
5.303
5.015
4.990
4.966
4.666
4.636
4.501
4.471
4.291
4.279
4.260
4.249
4.006
4.001
3.992
3.987
3.980
3.975
3.970
3.955
2.283
2.269
2.250
2.236
2.093
2.080
2.073
2.006
1.993
1.981
1.855
1.846
1.825
1.823
1.816
1.793
1.784
1.234
OAc
AcO
AcO
O
OBn
25α/β
9 8 7 6 5 4 3 2 1 ppm
1.00 2.02 0.15 0.10 0.13 0.96 0.32 1.13 1.10 0.14 1.90 0.13
1.14 0.46 2.48 0.28 2.66 0.50 2.68 1.17

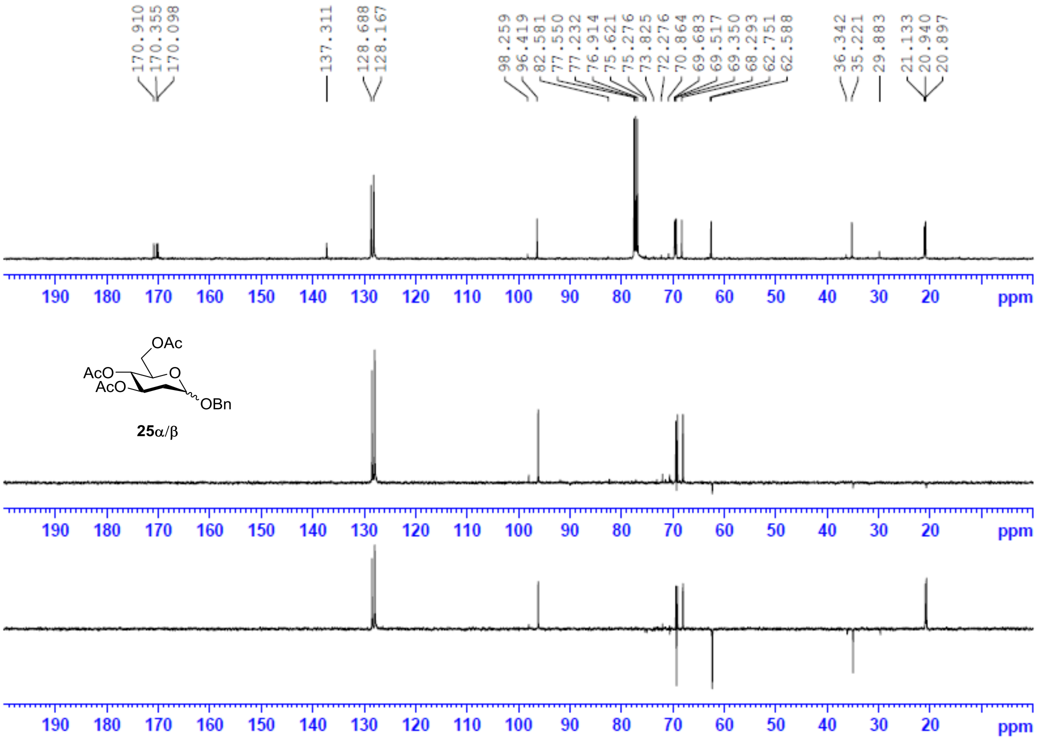
170.910
170.355
170.098
137.311
128.688
128.167
98.259
96.419
82.581
77.550
77.232
76.914
75.621
75.276
73.825
72.276
70.864
69.683
69.517
69.350
68.293
62.751
62.588
36.342
35.221
29.883
21.133
20.940
20.897
190 180 170 160 150 140 130 120 110 100 90 80 70 60 50 40 30 20 ppm
OAc
AcO
AcO
O
OBn
25α/β
190 180 170 160 150 140 130 120 110 100 90 80 70 60 50 40 30 20 ppm
190 180 170 160 150 140 130 120 110 100 90 80 70 60 50 40 30 20 ppm

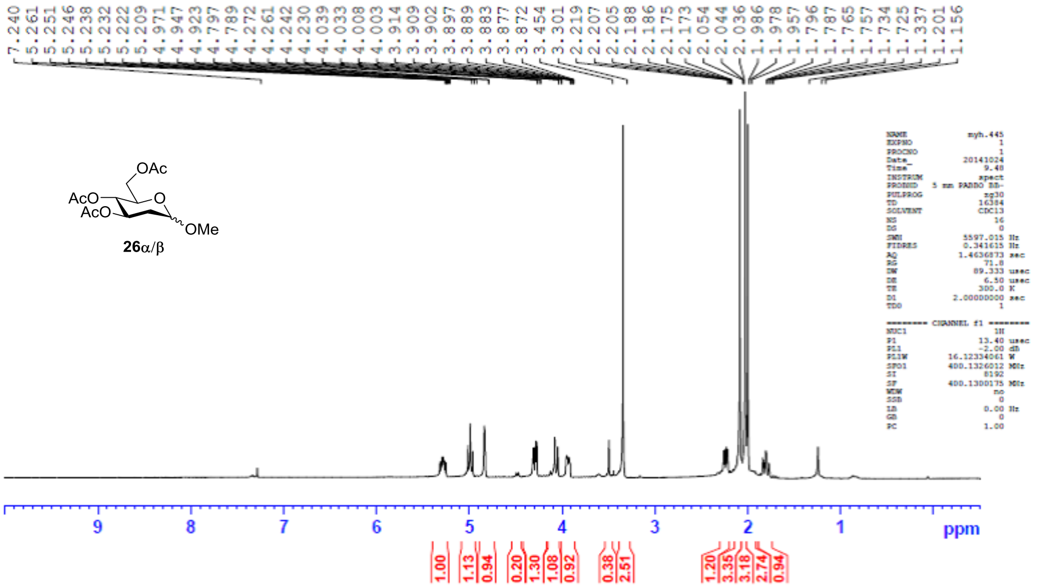
7.240
5.261
5.251
5.246
5.238
5.232
5.222
5.209
4.971
4.947
4.923
4.797
4.789
4.272
4.261
4.242
4.230
4.039
4.033
4.008
4.003
3.914
3.909
3.902
3.897
3.889
3.883
3.877
3.872
3.454
3.301
2.219
2.207
2.205
2.188
2.186
2.175
2.173
2.054
2.044
2.036
1.986
1.978
1.957
1.796
1.787
1.765
1.757
1.734
1.725
1.337
1.201
1.156
OAc
AcO
AcO
O
OMe
26α/β
NAME myh.445
EXPNO 1
PROCNO 1
Date_ 20141024
Time 9.48
INSTRUM spect
PROBHD 5 mm PABBO BB-
PULPROG zg30
TD 16384
SOLVENT CDCl3
NS 16
DS 0
SWH 5597.015 Hz
FIDRES 0.341615 Hz
AQ 1.4636873 sec
RG 71.8
DW 89.333 usec
DE 6.50 usec
TE 300.0 K
D1 2.00000000 sec
TD0 1
======== CHANNEL f1 ========
NUC1 1H
P1 13.40 usec
PL1 -2.00 dB
PL1W 16.12334061 W
SFO1 400.1326012 MHz
SI 8192
SF 400.1300175 MHz
WDW no
SSB 0
LB 0.00 Hz
GB 0
PC 1.00
9 8 7 6 5 4 3 2 1 ppm
1.00
1.13
0.94
0.20
1.30
1.08
0.92
0.38
2.51
1.20
3.35
3.18
2.74
0.94

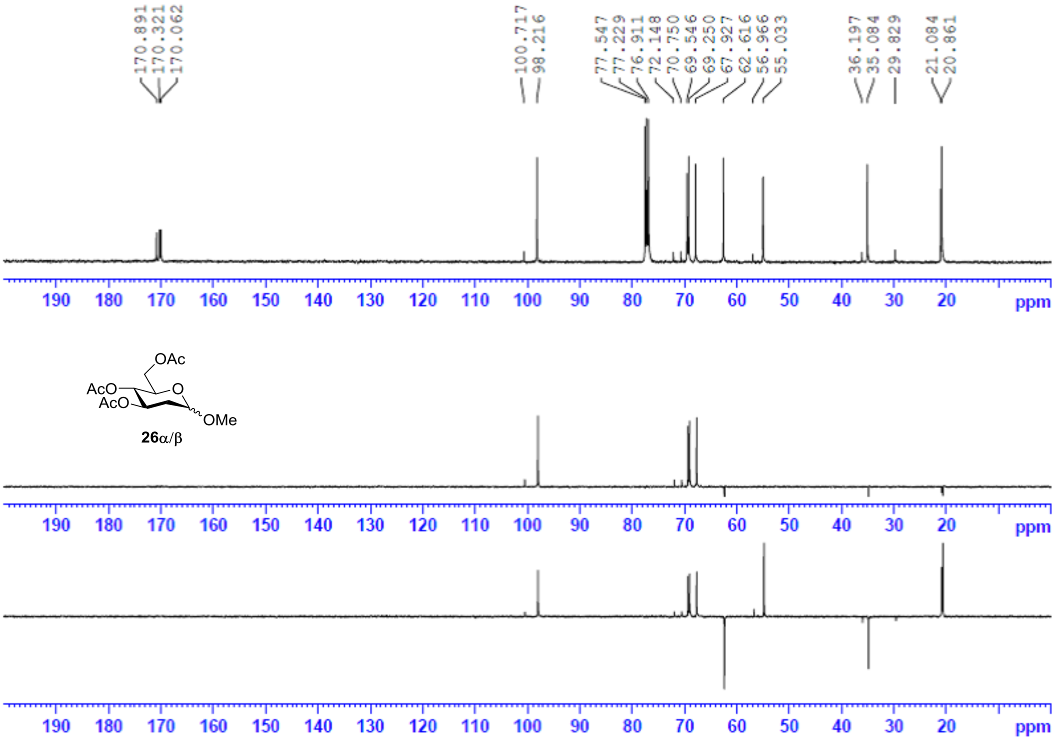
170.891
170.321
170.062
100.717
98.216
77.547
77.229
76.911
72.148
70.750
69.546
69.250
67.927
62.616
56.966
55.033
36.197
35.084
29.829
21.084
20.861
ppm
OAc
AcO
AcO
O
OMe
26α/β

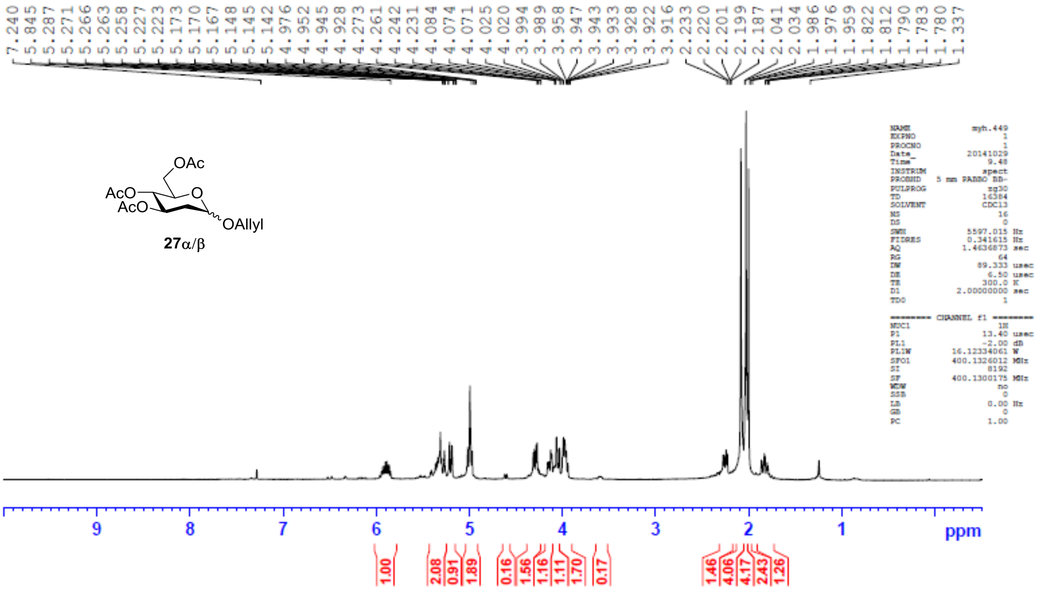
7.240
5.845
5.287
5.271
5.266
5.263
5.258
5.227
5.223
5.173
5.170
5.167
5.148
5.145
5.142
4.976
4.952
4.945
4.928
4.273
4.261
4.242
4.231
4.084
4.074
4.071
4.025
4.020
3.994
3.989
3.958
3.947
3.943
3.933
3.928
3.922
3.916
2.233
2.220
2.201
2.199
2.187
2.041
2.034
1.986
1.976
1.959
1.822
1.812
1.790
1.783
1.780
1.337
OAc
AcO
AcO
O
OAllyl
27α/β
ppm
1.00
2.08
0.91
1.89
0.16
1.56
1.16
1.11
1.70
0.17
1.46
4.06
4.17
2.43
1.26

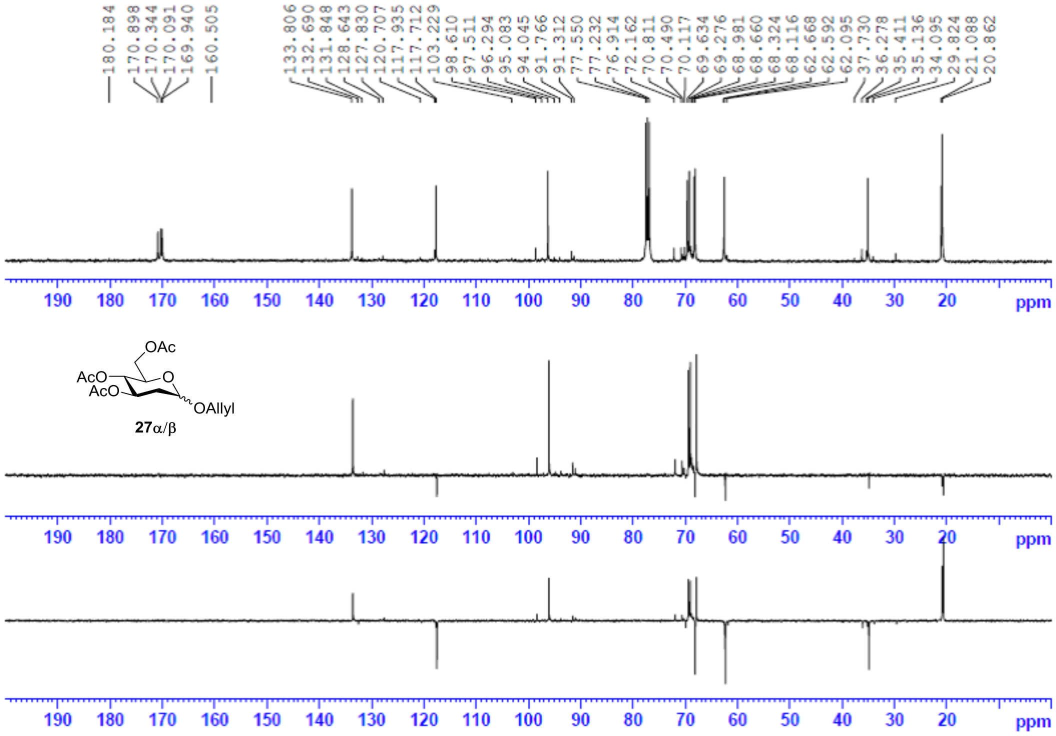

180.184
170.898
170.344
170.091
169.940
160.505
133.806
132.690
131.848
128.643
127.830
120.707
117.935
117.712
103.229
98.610
97.511
96.294
95.083
94.045
91.766
91.312
77.550
77.232
76.914
72.162
70.811
70.490
70.117
69.634
69.276
68.981
68.660
68.324
68.116
62.668
62.592
62.095
37.730
36.278
35.411
35.136
34.095
29.824
21.088
20.862
190 180 170 160 150 140 130 120 110 100 90 80 70 60 50 40 30 20 ppm
OAc
AcO
AcO
O
OAllyl
27α/β
190 180 170 160 150 140 130 120 110 100 90 80 70 60 50 40 30 20 ppm
190 180 170 160 150 140 130 120 110 100 90 80 70 60 50 40 30 20 ppm

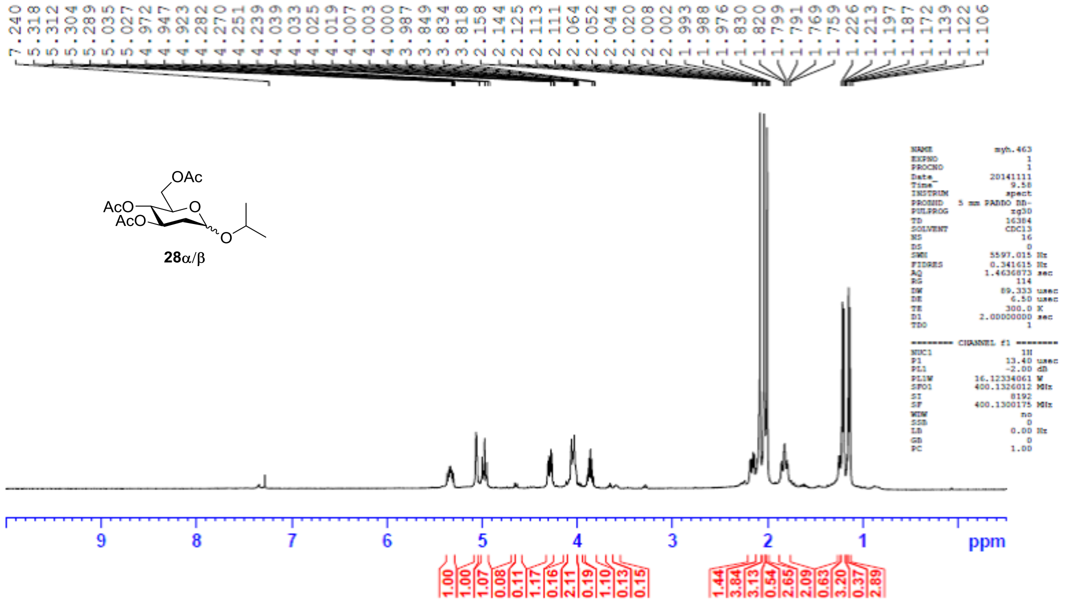

7.240
5.318
5.312
5.304
5.289
5.035
5.027
4.972
4.947
4.923
4.282
4.270
4.251
4.239
4.039
4.033
4.025
4.019
4.007
4.003
4.000
3.987
3.849
3.834
3.818
2.158
2.144
2.125
2.113
2.111
2.064
2.052
2.044
2.020
2.008
2.002
1.993
1.988
1.976
1.830
1.820
1.799
1.791
1.769
1.759
1.226
1.213
1.197
1.187
1.172
1.139
1.122
1.106
OAc
AcO
AcO
O
O
28α/β
9 8 7 6 5 4 3 2 1 ppm
1.00
1.00
1.07
0.08
0.11
1.17
0.16
2.11
0.19
1.10
0.13
0.15
1.44
3.84
3.13
0.54
2.65
2.09
0.63
3.20
0.37
2.89

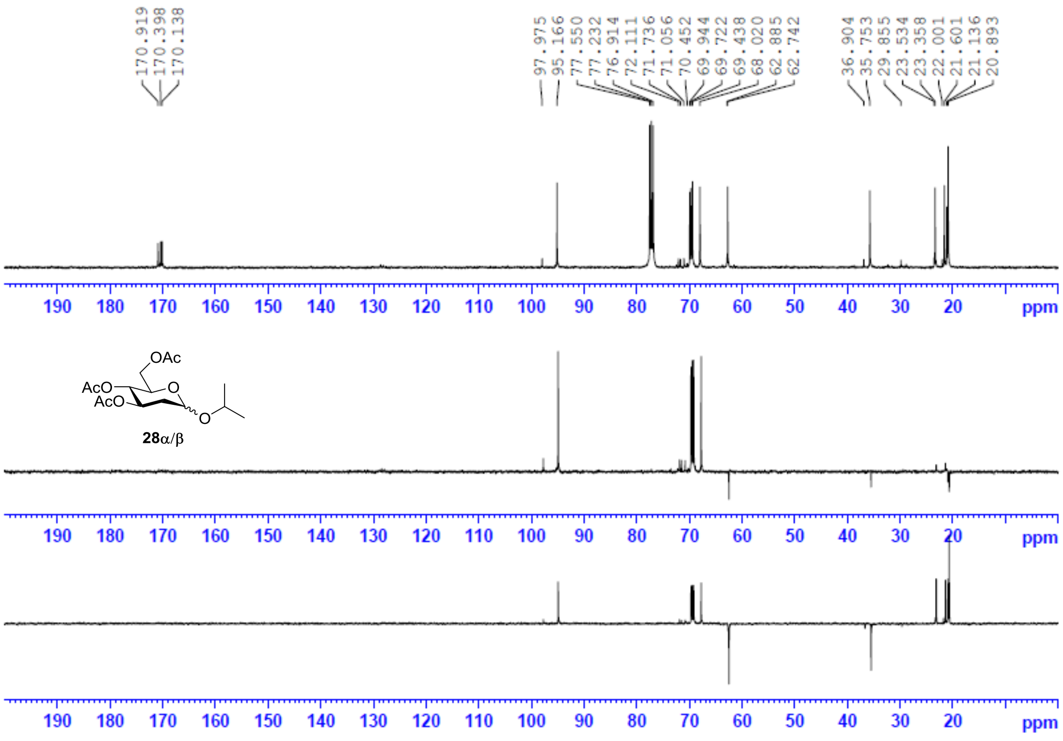
170.919
170.398
170.138
97.975
95.166
77.550
77.232
76.914
72.111
71.736
71.056
70.452
69.944
69.722
69.438
68.020
62.885
62.742
36.904
35.753
29.855
23.534
23.358
22.001
21.601
21.136
20.893
ppm
OAc
AcO
AcO
O
O
28α/β

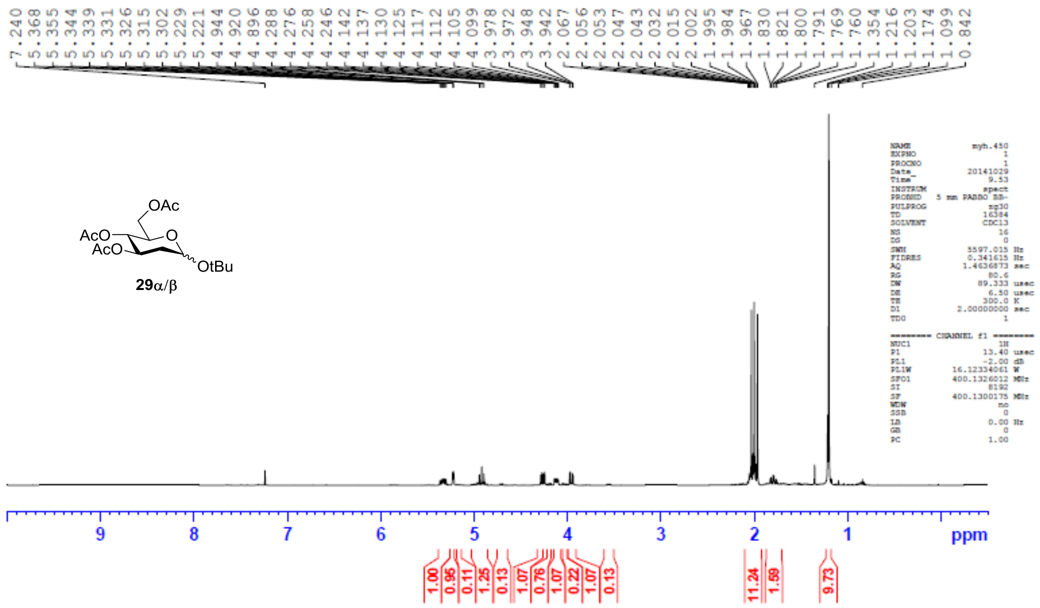
7.240
5.368
5.355
5.344
5.339
5.331
5.326
5.315
5.302
5.229
5.221
4.944
4.920
4.896
4.288
4.276
4.258
4.246
4.142
4.137
4.130
4.125
4.117
4.112
4.105
4.099
3.978
3.972
3.948
3.942
2.067
2.056
2.053
2.047
2.043
2.032
2.015
2.002
1.995
1.984
1.967
1.830
1.821
1.800
1.791
1.769
1.760
1.354
1.216
1.203
1.174
1.099
0.842
OAc
AcO
AcO
O
OtBu
29α/β
ppm
1.00
0.95
0.11
1.25
0.13
1.07
0.76
1.07
0.22
1.07
0.13
11.24
1.59
9.73

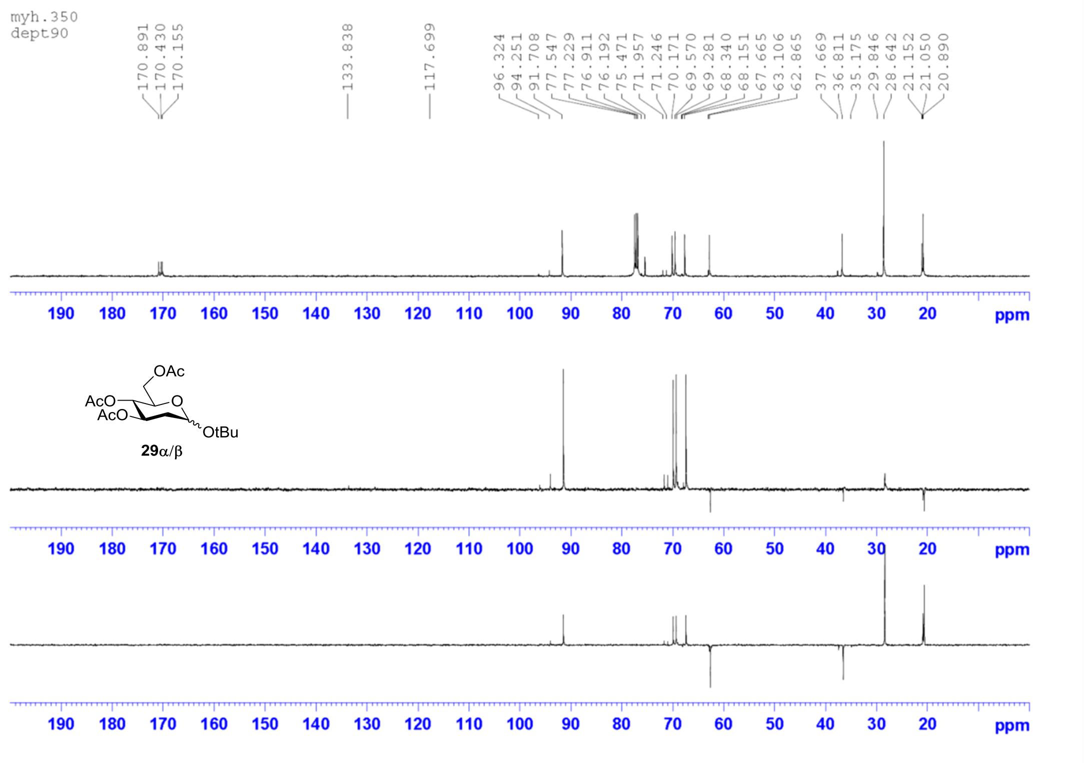
myh.350
dept90
170.891
170.430
170.155
133.838
117.699
96.324
94.251
91.708
77.547
77.229
76.911
76.192
75.471
71.957
71.246
70.171
69.570
69.281
68.340
68.151
67.665
63.106
62.865
37.669
36.811
35.175
29.846
28.642
21.152
21.050
20.890
ppm
OAc
AcO
AcO
O
OtBu
29α/β

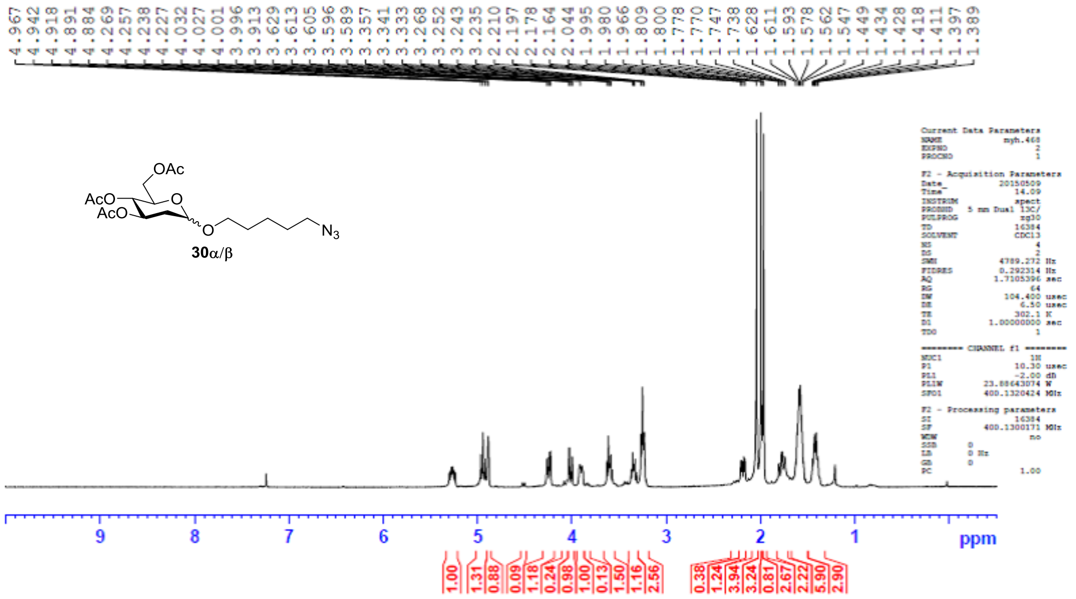
4.967
4.942
4.918
4.891
4.884
4.269
4.257
4.238
4.227
4.032
4.027
4.001
3.996
3.913
3.629
3.613
3.605
3.596
3.589
3.357
3.341
3.333
3.268
3.252
3.243
3.235
2.210
2.197
2.178
2.164
2.044
1.995
1.980
1.966
1.809
1.800
1.778
1.770
1.747
1.738
1.628
1.611
1.593
1.578
1.562
1.547
1.449
1.434
1.428
1.418
1.411
1.397
1.389
OAc
AcO
AcO
O
O
N_3
30α/β
ppm
1.00
1.31
0.88
0.09
1.18
0.24
0.98
1.00
0.13
1.50
1.16
2.56
0.38
1.24
3.94
3.24
0.81
2.67
2.22
5.90
2.90

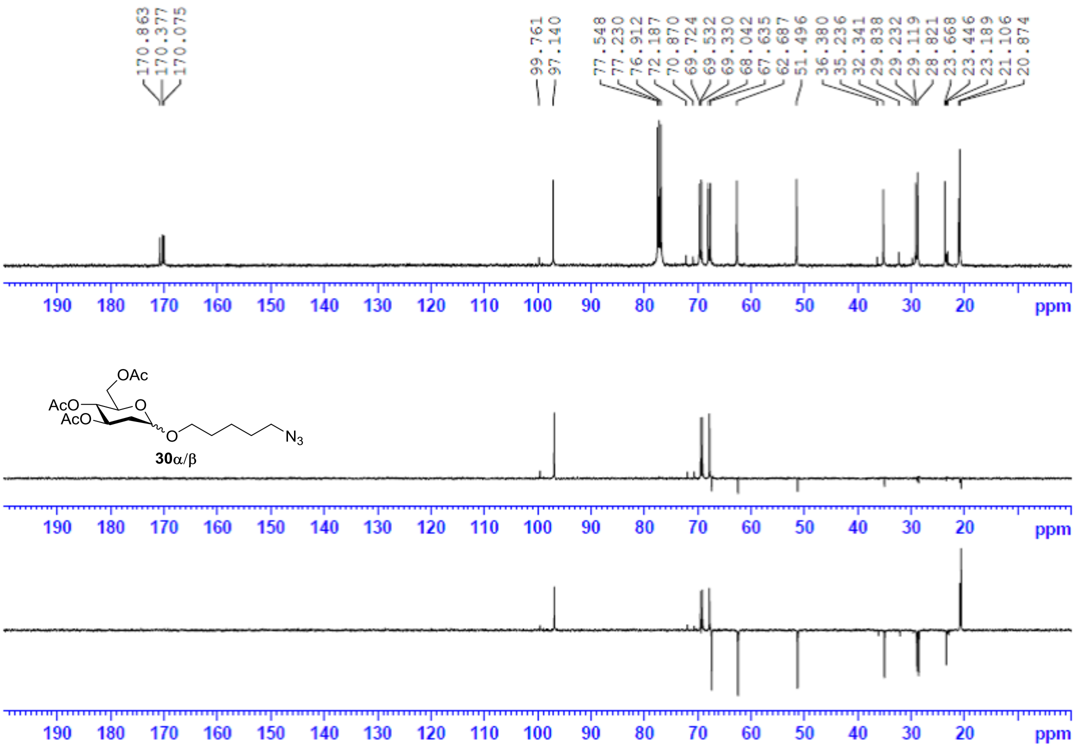
170.863
170.377
170.075
99.761
97.140
77.548
77.230
76.912
72.187
70.870
69.724
69.532
69.330
68.042
67.635
62.687
51.496
36.380
35.236
32.341
29.838
29.232
29.119
28.821
23.668
23.446
23.189
21.106
20.874
ppm
OAc
AcO
AcO
O
O
N_3
30α/β

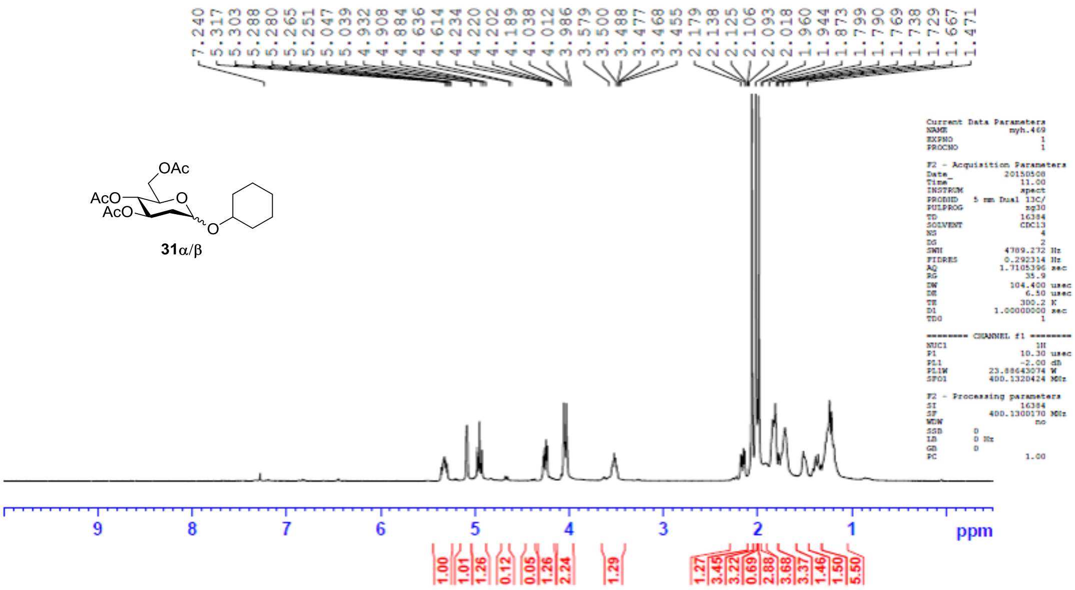
OAc
AcO
AcO
O
O
31α/β
ppm

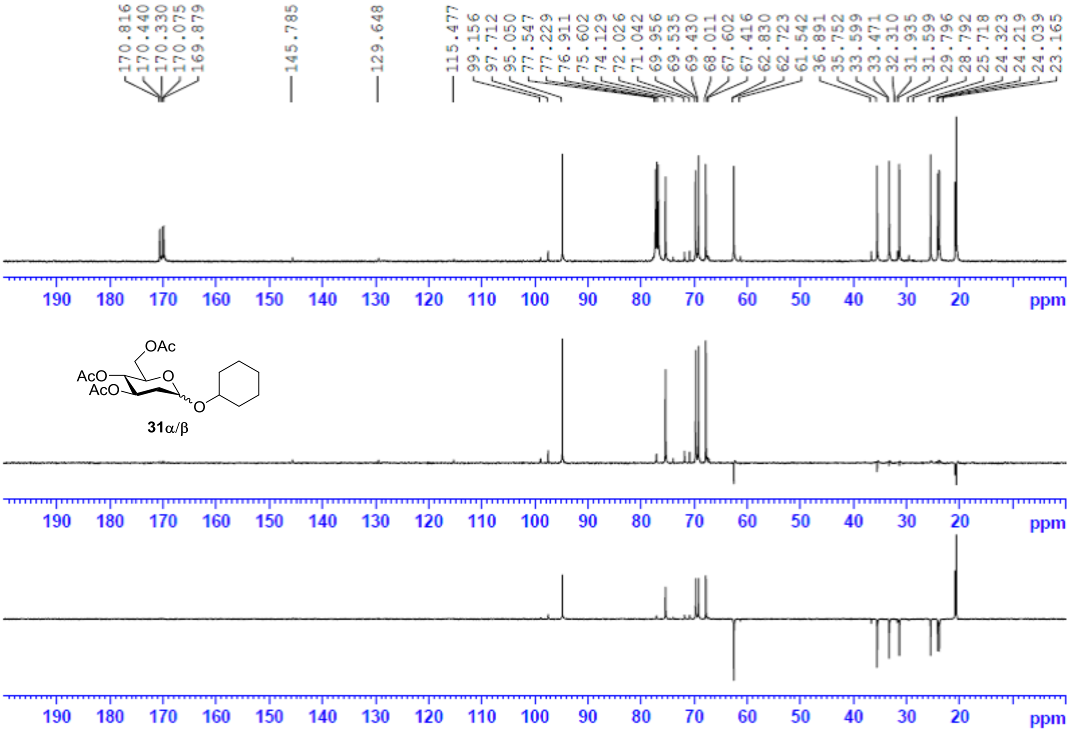
170.816
170.440
170.330
170.075
169.879
145.785
129.648
115.477
99.156
97.712
95.050
77.547
77.229
76.911
75.602
74.129
72.026
71.042
69.956
69.535
69.430
68.011
67.602
67.416
62.830
62.723
61.542
36.891
35.752
33.599
33.471
32.310
31.935
31.599
29.796
28.792
25.718
24.323
24.219
24.039
23.165
190 180 170 160 150 140 130 120 110 100 90 80 70 60 50 40 30 20 ppm
OAc
AcO
AcO
O
O
31α/β

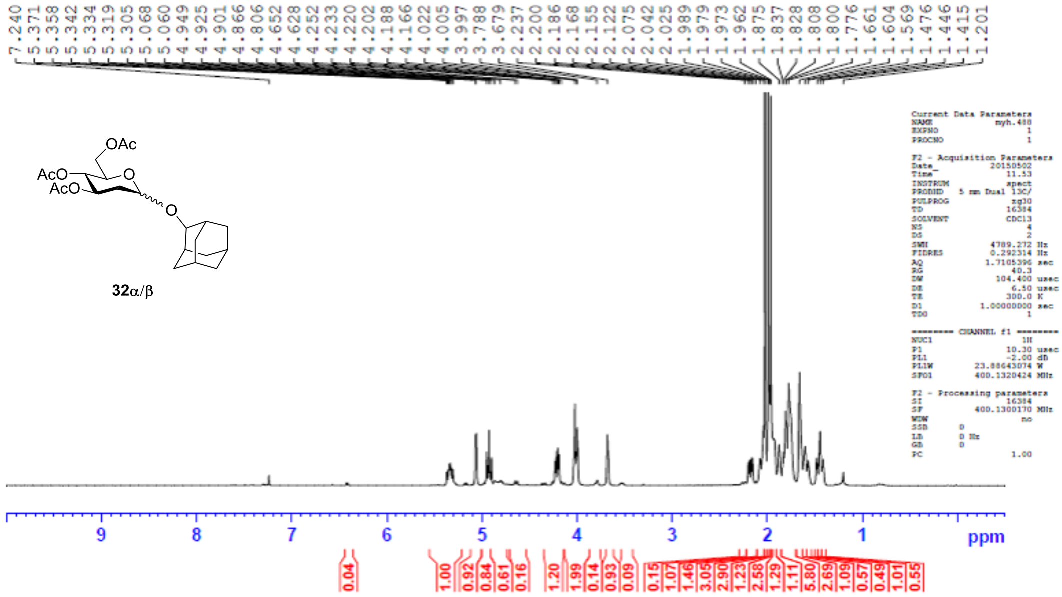
7.240
5.371
5.358
5.342
5.334
5.319
5.305
5.068
5.060
4.949
4.925
4.901
4.866
4.806
4.652
4.628
4.252
4.233
4.220
4.202
4.188
4.166
4.022
4.005
3.997
3.788
3.679
2.237
2.200
2.186
2.168
2.155
2.122
2.075
2.042
2.025
1.989
1.979
1.973
1.962
1.875
1.837
1.828
1.808
1.800
1.776
1.661
1.604
1.569
1.476
1.446
1.415
1.201
OAc
AcO
AcO
O
O
32α/β
9 8 7 6 5 4 3 2 1 ppm
0.04
1.00
0.92
0.84
0.61
0.16
1.20
1.99
0.14
0.93
0.09
0.15
1.07
1.46
3.05
2.90
1.23
2.58
1.29
1.11
5.80
2.69
1.09
0.57
0.49
1.01
0.55

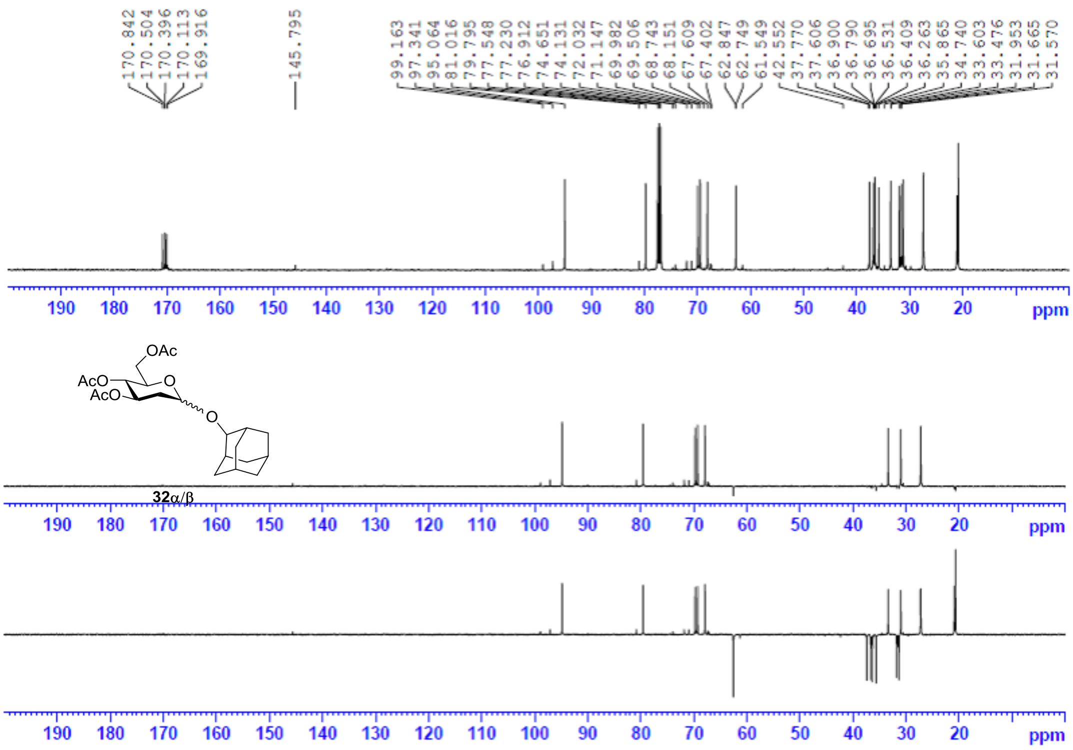
170.842
170.504
170.396
170.113
169.916
145.795
99.163
97.341
95.064
81.016
79.795
77.548
77.230
76.912
74.651
74.131
72.032
71.147
69.982
69.506
68.743
68.151
67.609
67.402
62.847
62.749
61.549
42.552
37.770
37.606
36.900
36.790
36.695
36.531
36.409
36.263
35.865
34.740
33.603
33.476
31.953
31.665
31.570
OAc
AcO
AcO
O
O
190 180 170 160 150 140 130 120 110 100 90 80 70 60 50 40 30 20 ppm

32α/β

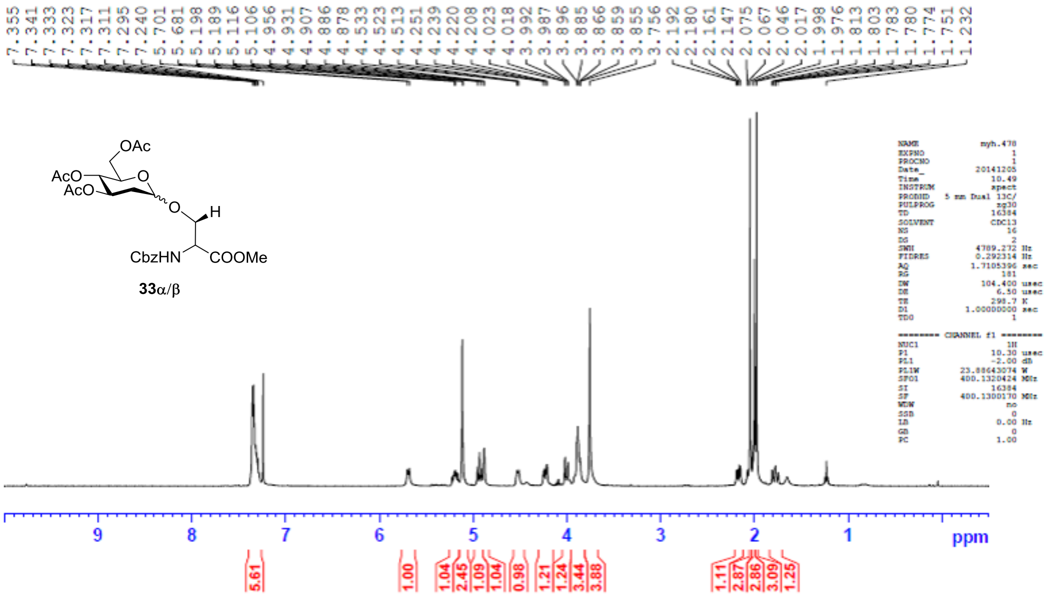
7.355
7.341
7.333
7.323
7.317
7.311
7.295
7.240
5.701
5.681
5.198
5.189
5.116
5.106
4.956
4.931
4.907
4.886
4.878
4.533
4.523
4.513
4.251
4.239
4.220
4.208
4.023
4.018
3.992
3.987
3.896
3.885
3.866
3.859
3.855
3.756
2.192
2.180
2.161
2.147
2.075
2.067
2.046
2.017
1.998
1.976
1.813
1.803
1.783
1.780
1.774
1.751
1.232
OAc
AcO
AcO
O
O
H
CbzHN
COOMe
9 8 7 6 5 4 3 2 1 ppm
5.61
1.00
1.04
2.45
1.09
1.04
0.98
1.21
1.24
3.44
3.88
1.11
2.87
2.86
3.09
1.25

33α/β

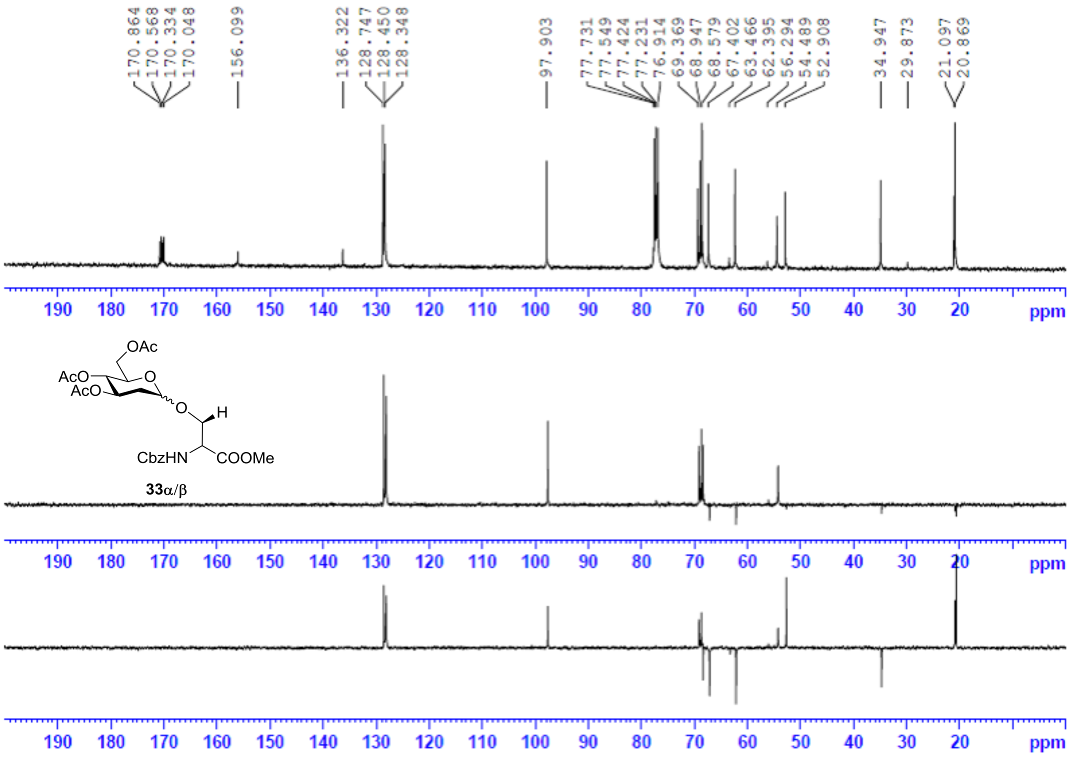
170.864
170.568
170.334
170.048
156.099
136.322
128.747
128.450
128.348
97.903
77.731
77.549
77.424
77.231
76.914
69.369
68.947
68.579
67.402
63.466
62.395
56.294
54.489
52.908
34.947
29.873
21.097
20.869
ppm
OAc
AcO
AcO
O
O
H
CbzHN
COOMe
33α/β

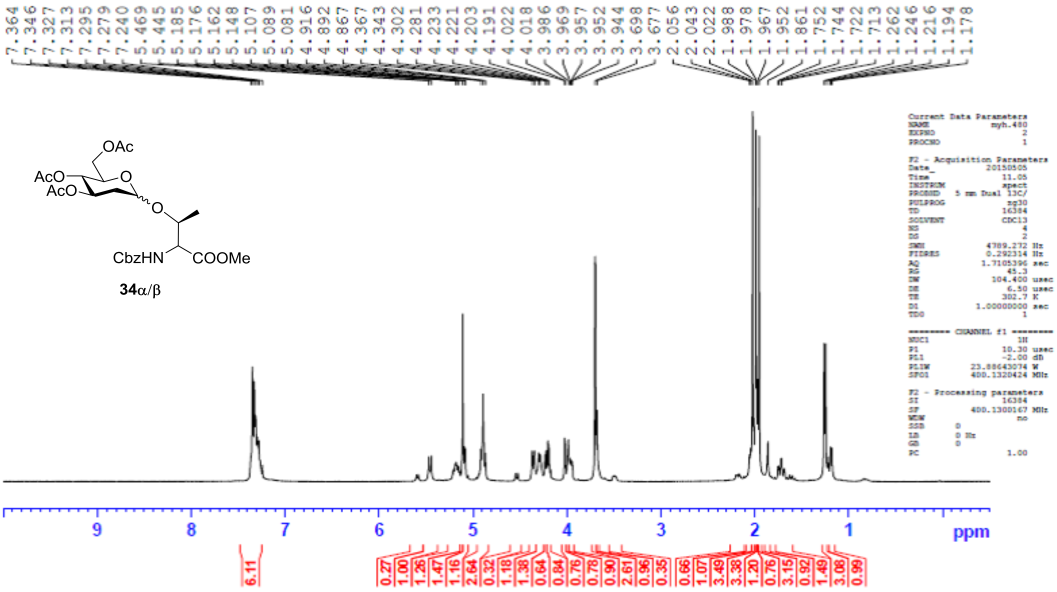
7.364
7.346
7.327
7.313
7.295
7.279
7.240
5.469
5.445
5.185
5.176
5.162
5.148
5.107
5.089
5.081
4.916
4.892
4.867
4.367
4.343
4.302
4.281
4.233
4.221
4.203
4.191
4.022
4.018
3.986
3.969
3.957
3.952
3.944
3.698
3.677
2.056
2.043
2.022
1.988
1.978
1.967
1.952
1.861
1.752
1.744
1.722
1.713
1.262
1.246
1.216
1.194
1.178
OAc
AcO
AcO
O
O
CbzHN
COOMe
34α/β
ppm

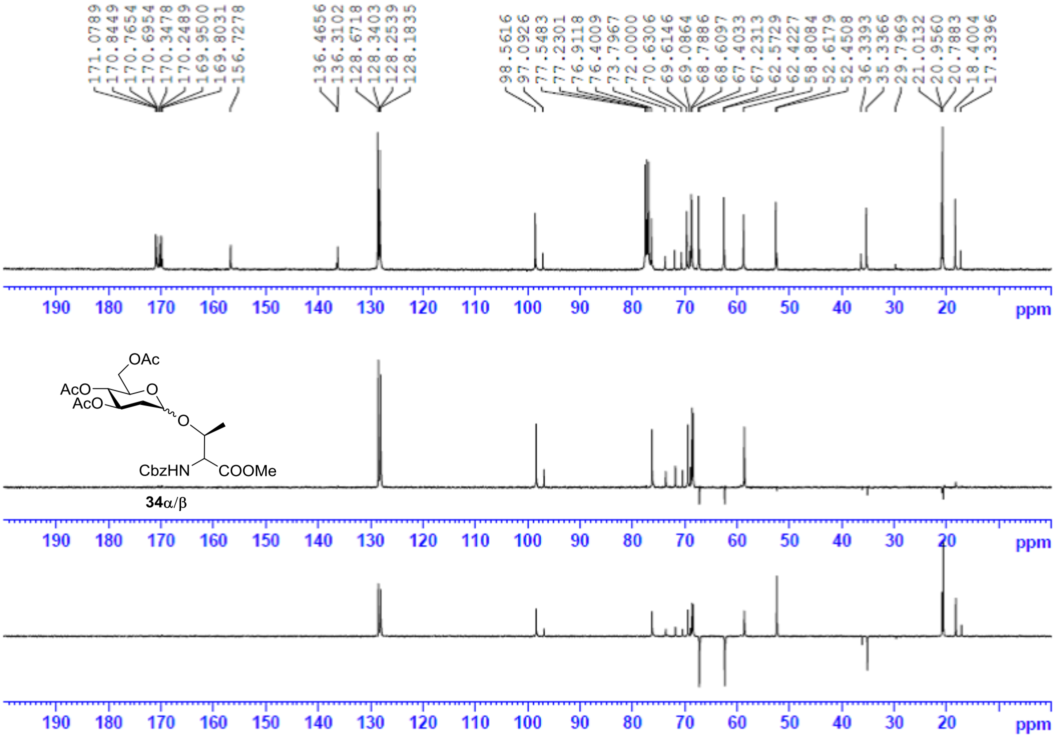
171.0789
170.8449
170.7654
170.6954
170.3478
170.2489
169.9500
169.8031
156.7278
136.4656
136.3102
128.6718
128.3403
128.2539
128.1835
98.5616
97.0926
77.5483
77.2301
76.9118
76.4009
73.7967
72.0000
70.6306
69.6146
69.0864
68.7886
68.6097
67.4033
67.2313
62.5729
62.4227
58.8084
52.6179
52.4508
36.3393
35.3366
29.7969
21.0132
20.9560
20.7883
18.4004
17.3396
ppm
OAc
AcO
AcO
O
O
CbzHN
COOMe

34α/β

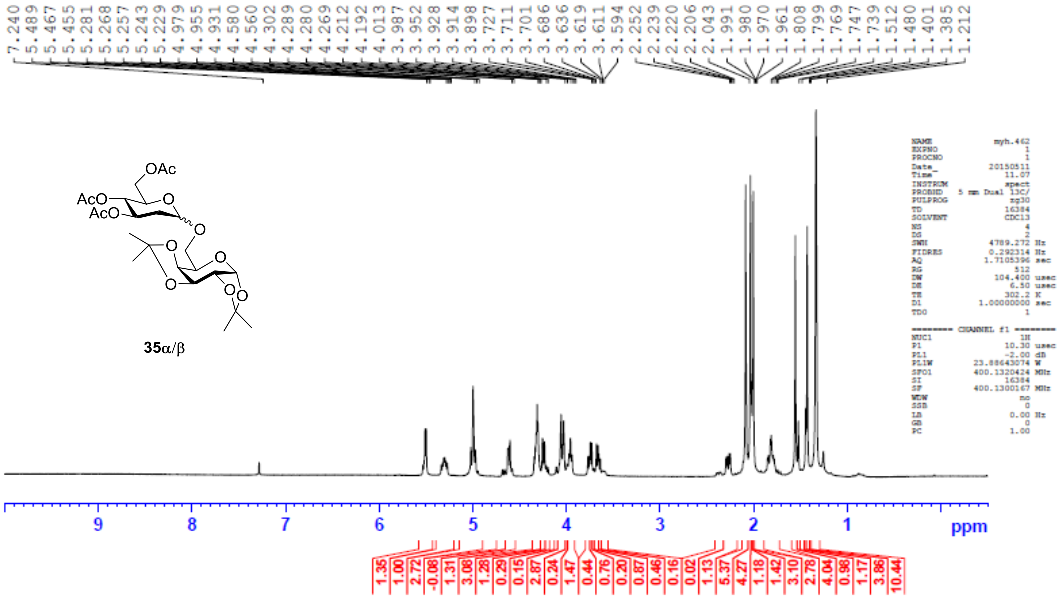
7.240
5.489
5.467
5.455
5.281
5.268
5.257
5.243
5.229
4.979
4.955
4.931
4.580
4.560
4.302
4.289
4.280
4.269
4.212
4.192
4.013
3.987
3.952
3.928
3.914
3.898
3.727
3.711
3.701
3.686
3.636
3.619
3.611
3.594
2.252
2.239
2.220
2.206
2.043
1.991
1.980
1.970
1.961
1.808
1.799
1.769
1.747
1.739
1.512
1.480
1.401
1.385
1.212
ppm
1.35
1.00
2.72
-0.08
1.31
3.08
1.28
0.29
0.15
2.87
0.24
1.47
0.44
0.76
0.20
0.87
0.46
0.16
0.02
1.13
5.37
4.27
1.18
1.42
3.10
2.78
4.04
0.98
1.17
3.86
10.44
OAc
AcO
AcO
O
O
O
O
O
O
O

35α/β

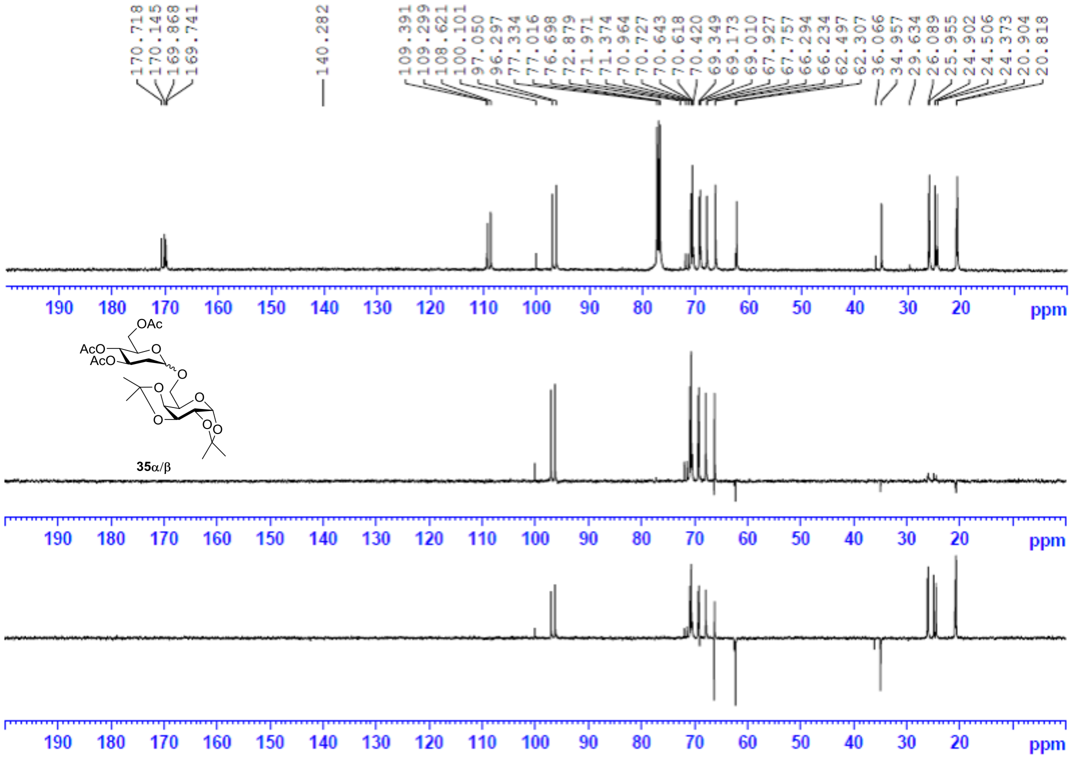
170.718
170.145
169.868
169.741
140.282
109.391
109.299
108.621
100.101
97.050
96.297
77.334
77.016
76.698
72.879
71.971
71.374
70.964
70.727
70.643
70.618
70.420
69.349
69.173
69.010
67.927
67.757
66.294
66.234
62.497
62.307
36.066
34.957
29.634
26.089
25.955
24.902
24.506
24.373
20.904
20.818
ppm
OAc
AcO
AcO
O
O
35α/β

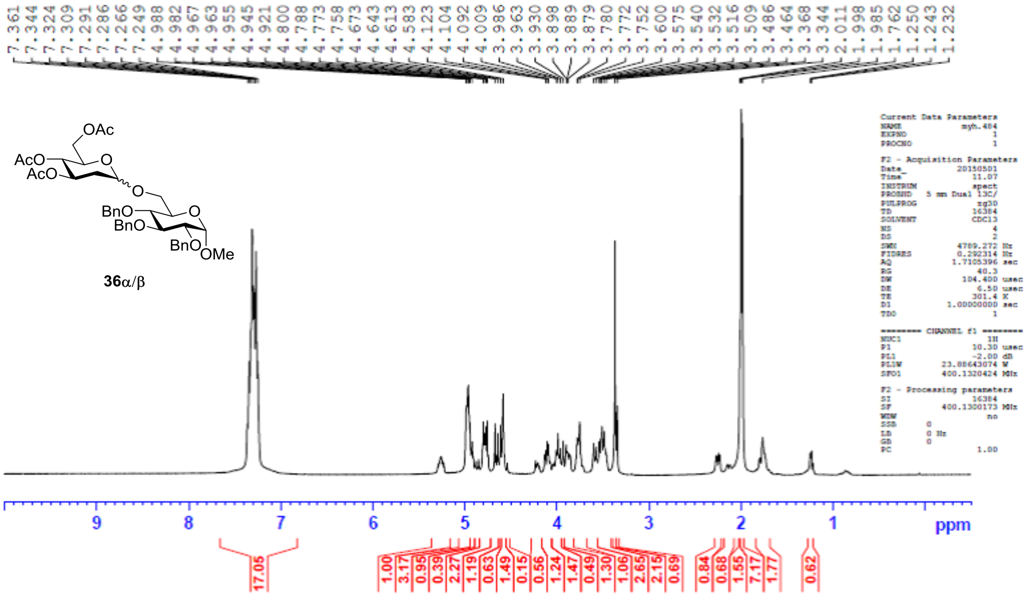
7.361
7.344
7.324
7.309
7.291
7.286
7.266
7.249
4.988
4.967
4.963
4.955
4.945
4.921
4.800
4.788
4.773
4.758
4.673
4.643
4.613
4.583
4.123
4.104
4.092
4.009
3.986
3.963
3.930
3.898
3.889
3.879
3.780
3.772
3.752
3.600
3.575
3.540
3.532
3.516
3.509
3.486
3.464
3.368
3.344
2.011
1.998
1.985
1.762
1.250
1.243
1.232
OAc
AcO
AcO
BnO
BnO
BnO
OMe
36α/β
ppm
17.05
1.00
3.17
0.95
0.39
2.27
1.19
0.63
1.49
0.15
0.56
1.24
1.47
0.49
1.30
1.06
2.65
2.15
0.69
0.84
0.68
1.55
7.17
1.77
0.62

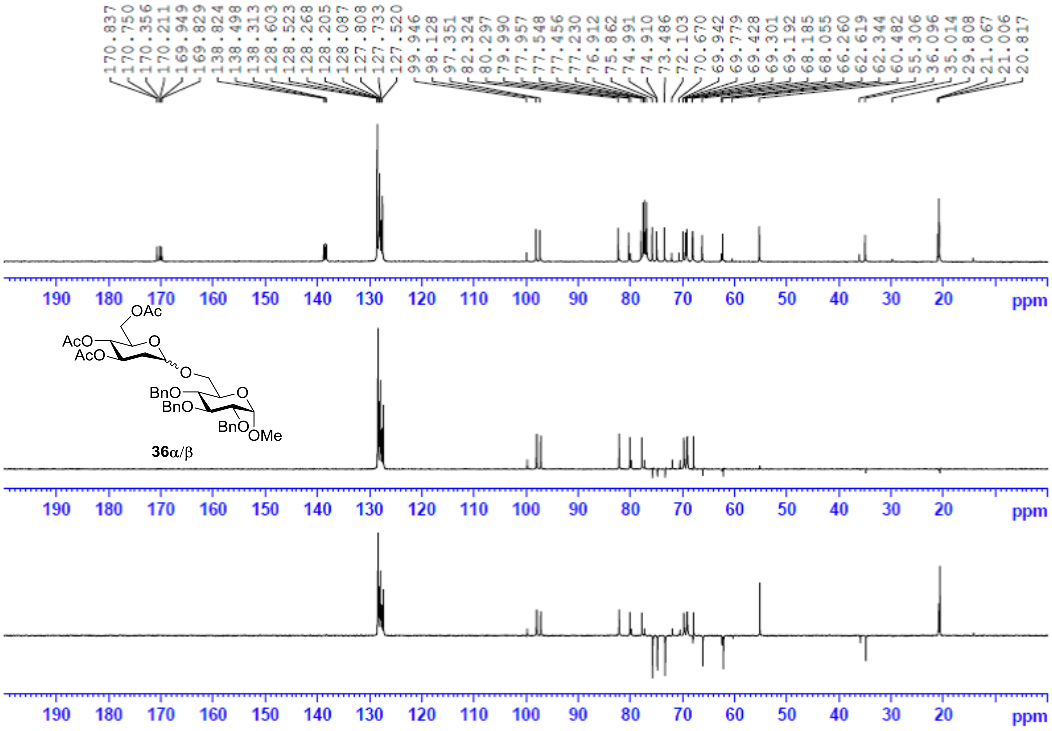
170.837
170.750
170.356
170.211
169.949
169.829
138.824
138.498
138.313
128.603
128.523
128.268
128.205
128.087
127.808
127.733
127.520
99.946
98.128
97.351
82.324
80.297
79.990
77.957
77.548
77.456
77.230
76.912
75.862
74.991
74.910
73.486
72.103
70.670
69.942
69.779
69.428
69.301
69.192
68.185
68.055
66.260
62.619
62.344
60.482
55.306
36.096
35.014
29.808
21.067
21.006
20.817
190 180 170 160 150 140 130 120 110 100 90 80 70 60 50 40 30 20 ppm
OAc
AcO
AcO
O
O
BnO
BnO
O
BnO
OMe
36α/β

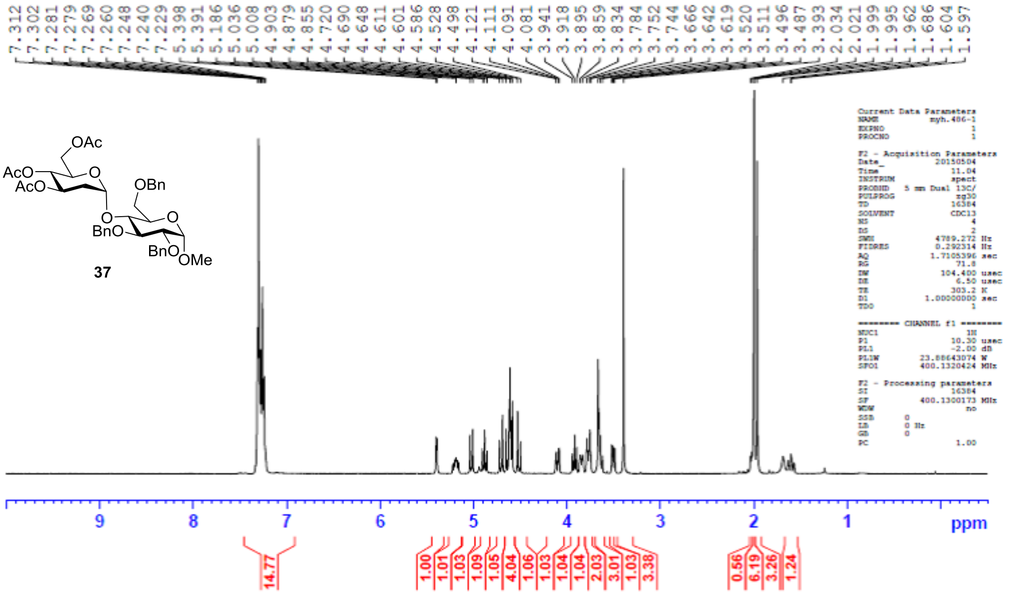
7.312
7.302
7.281
7.279
7.260
7.248
7.240
7.229
5.398
5.391
5.186
5.036
5.003
4.903
4.879
4.855
4.720
4.690
4.648
4.611
4.601
4.586
4.528
4.498
4.121
4.111
4.091
4.081
3.941
3.918
3.895
3.859
3.834
3.784
3.752
3.744
3.666
3.642
3.619
3.520
3.511
3.496
3.487
3.393
2.034
2.021
1.999
1.995
1.962
1.686
1.604
1.597
OAc
AcO
AcO
O
OBn
O
BnO
O
BnO
OMe
37
9 8 7 6 5 4 3 2 1 ppm
14.77
1.00
1.01
1.03
1.09
1.05
4.04
1.06
1.03
1.04
1.04
2.03
3.01
1.03
3.38
0.56
6.19
3.26
1.24

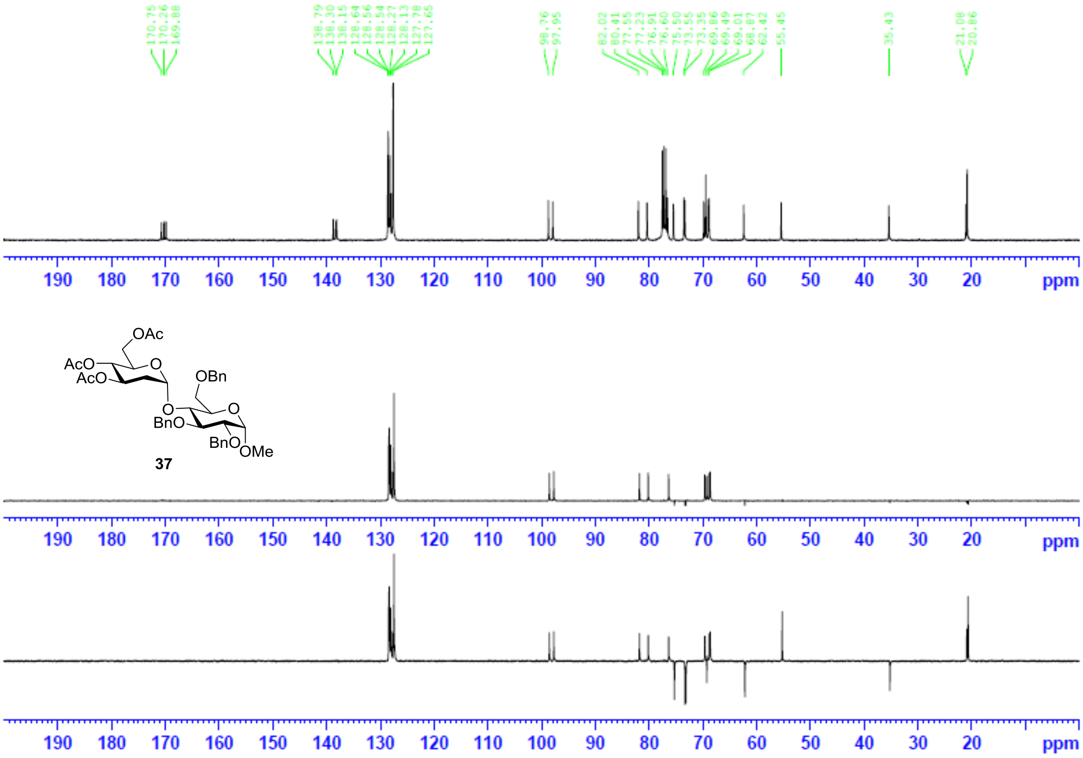
37
ppm

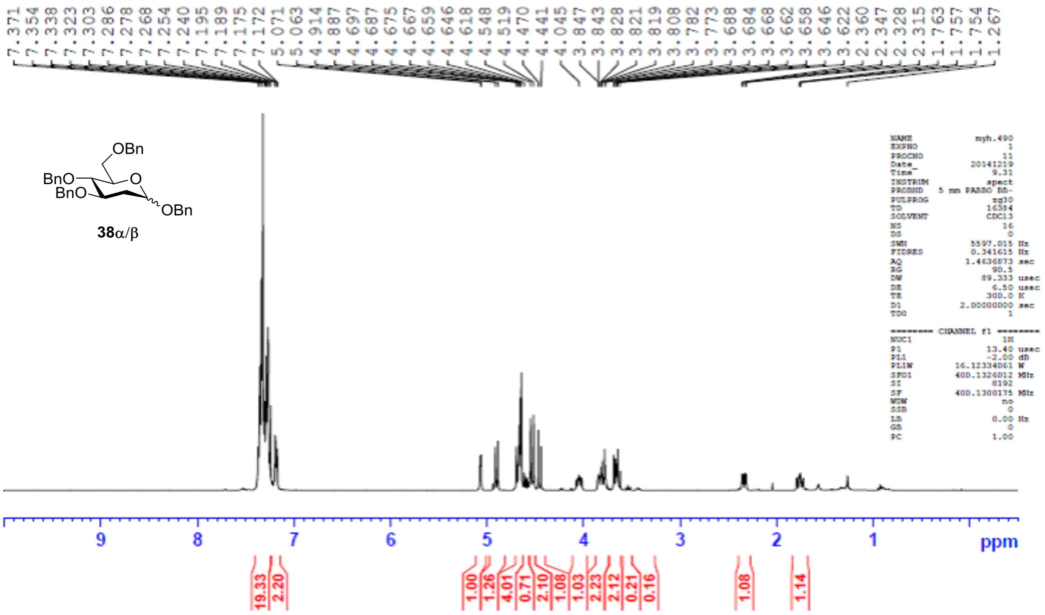
38α/β
ppm

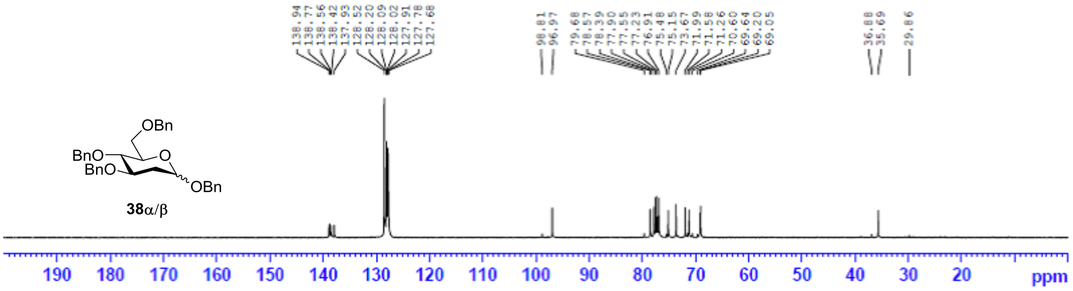
OBn
BnO
BnO
O
OBn
38α/β
ppm

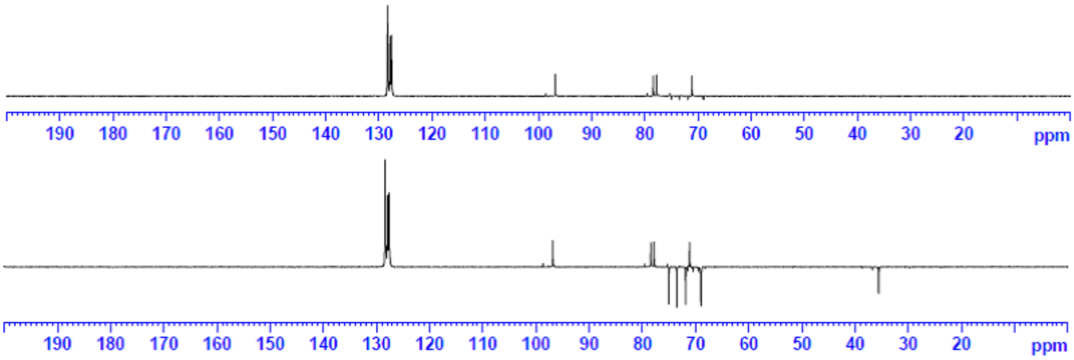
ppm

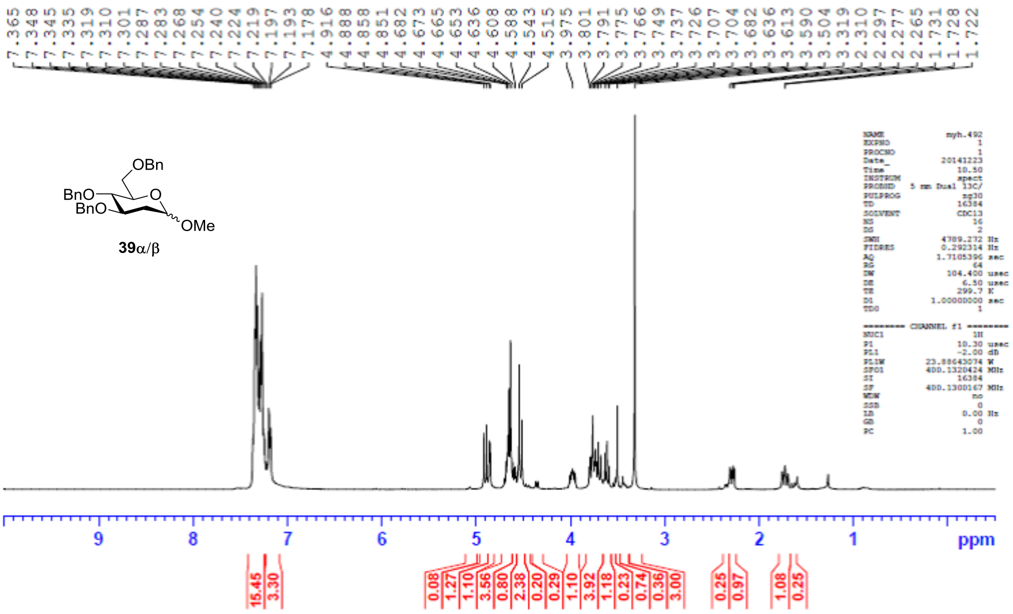
OBn
BnO
BnO
O
OMe
39α/β
ppm

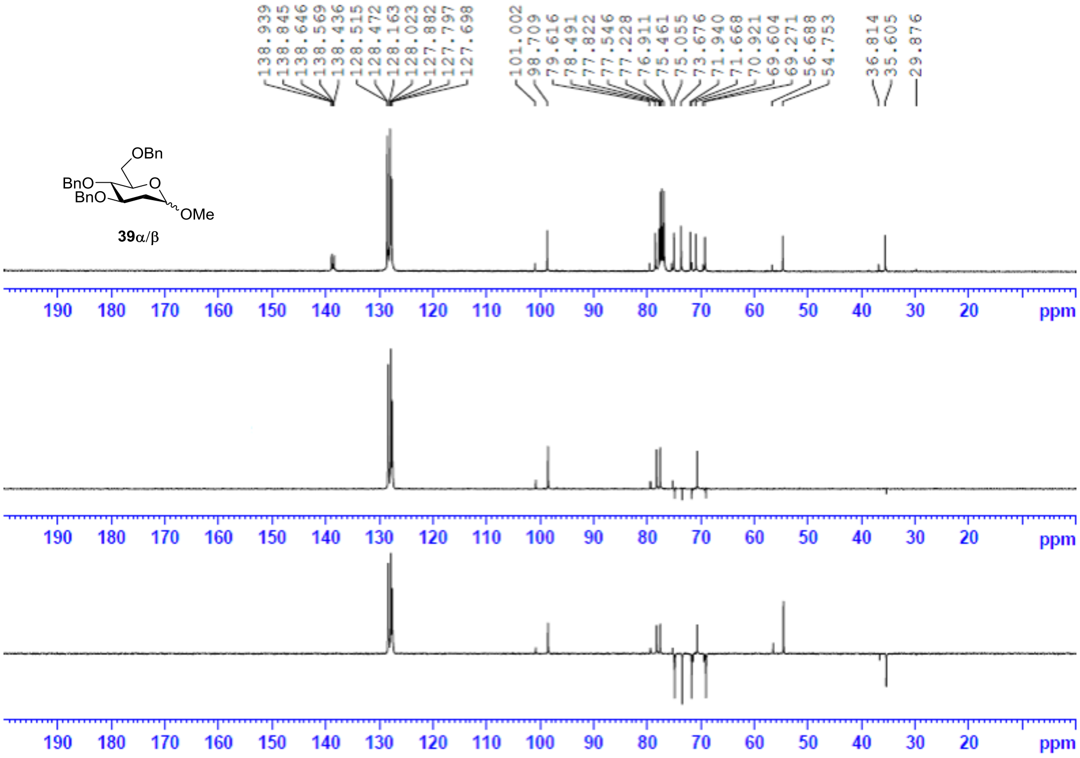
138.939
138.845
138.646
138.569
138.436
128.515
128.472
128.163
128.023
127.882
127.797
127.698
101.002
98.709
79.616
78.491
77.822
77.546
77.228
76.911
75.461
75.055
73.676
71.940
71.668
70.921
69.604
69.271
56.688
54.753
36.814
35.605
29.876
OBn
BnO
BnO
O
OMe
39α/β
ppm

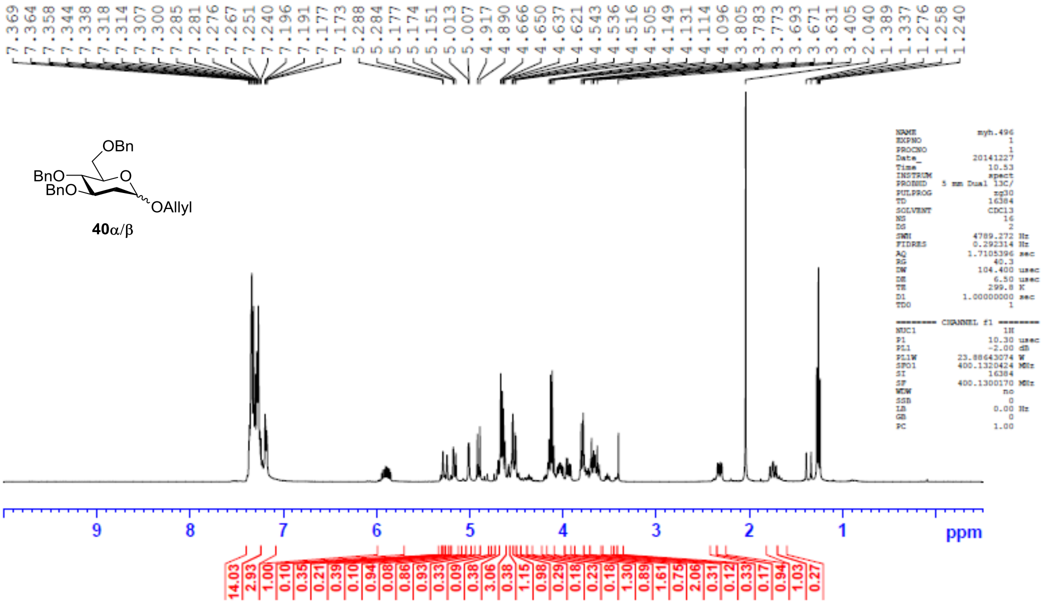
7.369
7.364
7.358
7.344
7.338
7.318
7.314
7.307
7.300
7.285
7.281
7.276
7.267
7.251
7.240
7.196
7.191
7.177
7.173
5.288
5.284
5.177
5.174
5.151
5.013
5.007
4.917
4.890
4.666
4.650
4.637
4.621
4.543
4.536
4.516
4.505
4.149
4.131
4.114
4.096
3.805
3.783
3.773
3.693
3.671
3.631
3.405
2.040
1.389
1.337
1.276
1.258
1.240
OBn
BnO
BnO
O
OAllyl
40α/β
ppm

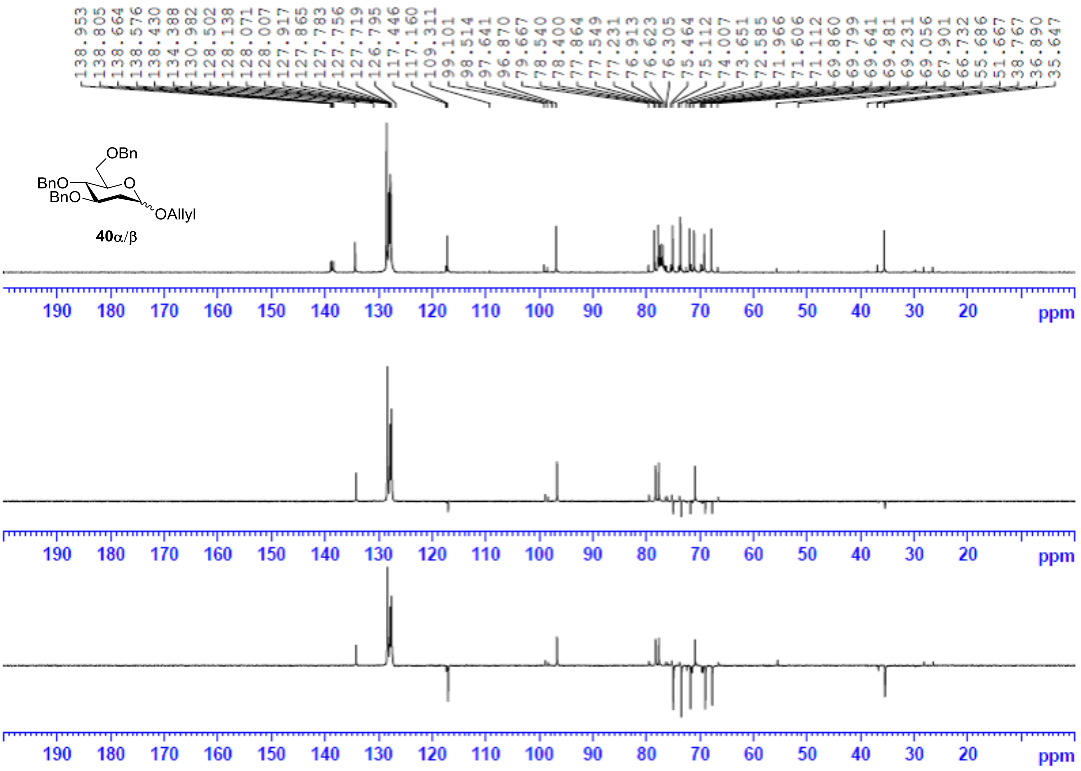
138.953 138.805 138.664 138.576 138.430 134.388 130.982 128.502 128.138 128.071 128.007 127.917 127.865 127.783 127.756 127.719 126.795 117.446 117.160 109.311 99.101 98.514 97.641 96.870 79.667 78.540 78.400 77.864 77.549 77.231 76.913 76.623 76.305 75.464 75.112 74.007 73.651 72.585 71.966 71.606 71.112 69.860 69.799 69.641 69.481 69.231 69.056 67.901 66.732 55.686 51.667 38.767 36.890 35.647
OBn
BnO
BnO
O
OAllyl
40α/β
190 180 170 160 150 140 130 120 110 100 90 80 70 60 50 40 30 20 ppm

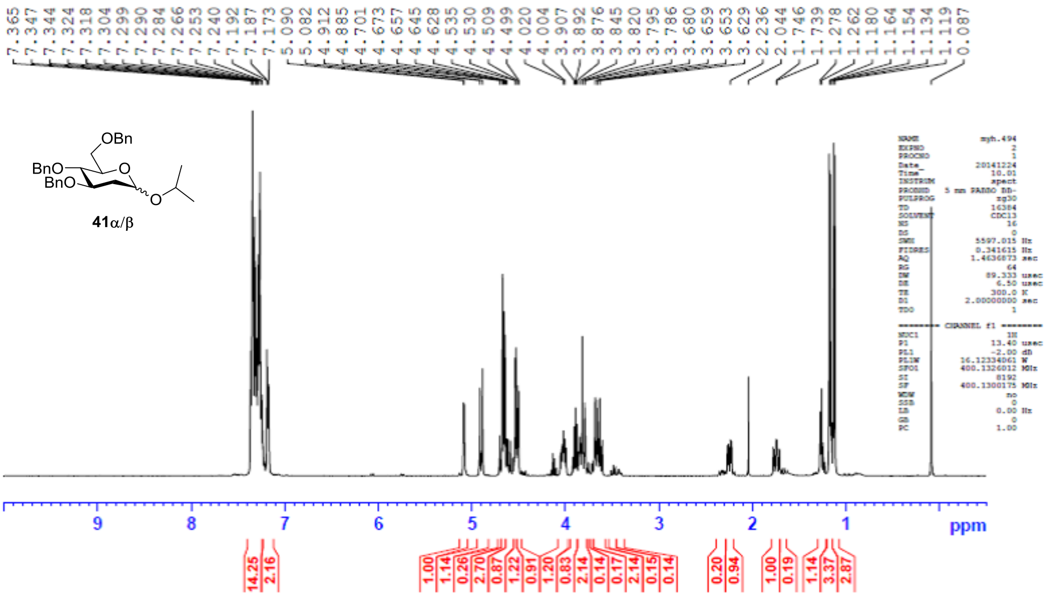
7.365 7.347 7.344 7.324 7.318 7.304 7.299 7.290 7.284 7.266 7.253 7.240 7.192 7.187 7.173 5.090 5.082 4.912 4.885 4.701 4.673 4.657 4.645 4.628 4.535 4.530 4.509 4.499 4.020 4.004 3.907 3.892 3.876 3.845 3.820 3.795 3.786 3.680 3.659 3.653 3.629 2.236 2.044 1.746 1.739 1.278 1.262 1.180 1.164 1.154 1.134 1.119 0.087
OBn
BnO
BnO
O
O
41α/β
9 8 7 6 5 4 3 2 1 ppm
14.25 2.16 1.00 1.14 0.26 2.70 0.87 1.22 0.91 1.20 0.83 2.14 0.14 0.17 2.14 0.15 0.14 0.20 0.94 1.00 0.19 1.14 3.37 2.87

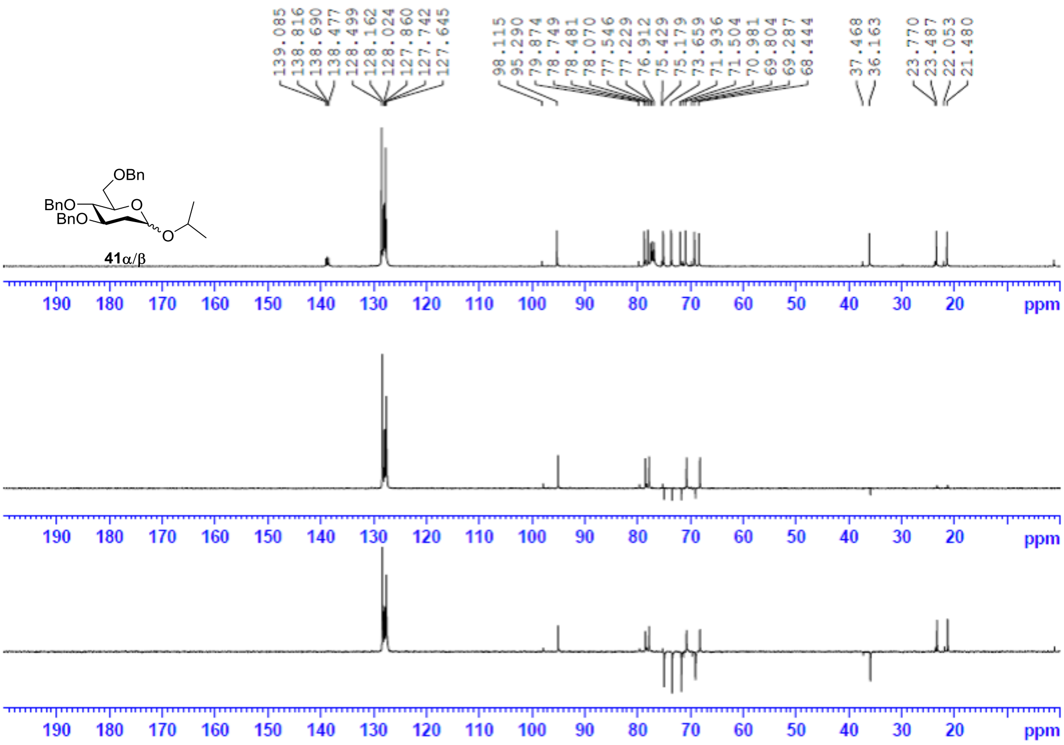

139.085
138.816
138.690
138.477
128.499
128.162
128.024
127.860
127.742
127.645
98.115
95.290
79.874
78.749
78.481
78.070
77.546
77.229
76.912
75.429
75.179
73.659
71.936
71.504
70.981
69.804
69.287
68.444
37.468
36.163
23.770
23.487
22.053
21.480
OBn
BnO
BnO
O
O
190 180 170 160 150 140 130 120 110 100 90 80 70 60 50 40 30 20 ppm
190 180 170 160 150 140 130 120 110 100 90 80 70 60 50 40 30 20 ppm
190 180 170 160 150 140 130 120 110 100 90 80 70 60 50 40 30 20 ppm

41α/β

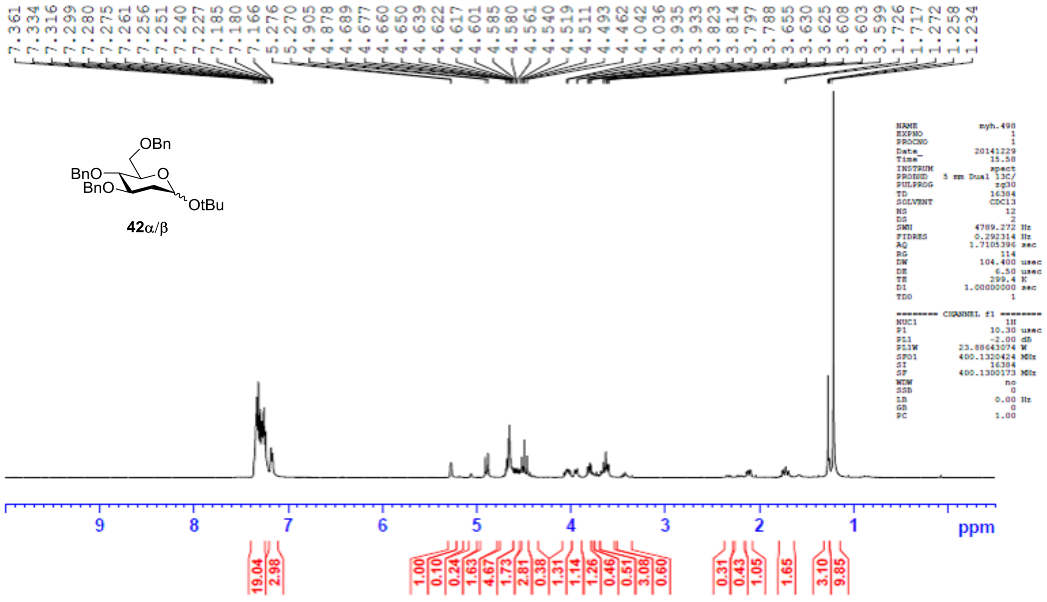

7.361
7.334
7.316
7.299
7.280
7.275
7.261
7.256
7.251
7.240
7.227
7.185
7.180
7.166
5.276
5.270
4.905
4.878
4.689
4.677
4.660
4.650
4.639
4.622
4.617
4.601
4.585
4.580
4.561
4.540
4.519
4.511
4.493
4.462
4.042
4.036
3.935
3.933
3.823
3.814
3.797
3.788
3.655
3.630
3.625
3.603
3.599
1.726
1.717
1.272
1.258
1.234
OBn
BnO
BnO
O
OtBu
NAME myh.498
EXPNO 1
PROCNO 1
Date_ 20141229
Time 15.58
INSTRUM spect
PROBHD 5 mm Dual 13C/
PULPROG zg30
TD 16384
SOLVENT CDCl3
NS 12
DS 2
SWH 4789.272 Hz
FIDRES 0.292314 Hz
AQ 1.7105396 sec
RG 114
DW 104.400 usec
DE 6.50 usec
TE 299.4 K
D1 1.00000000 sec
TD0 1
======= CHANNEL f1 =======
NUC1 1H
P1 10.30 usec
PL1 -2.00 dB
PL1W 23.88643074 W
SFO1 400.1320424 MHz
SI 16384
SF 400.1300173 MHz
WDW no
SSB 0
LB 0.00 Hz
GB 0
PC 1.00
9 8 7 6 5 4 3 2 1 ppm
19.04
2.98
1.00
0.10
0.24
1.63
4.67
1.73
2.81
0.38
1.31
1.14
1.26
0.46
0.51
3.08
0.60
0.31
0.43
1.05
1.65
3.10
9.85

42α/β

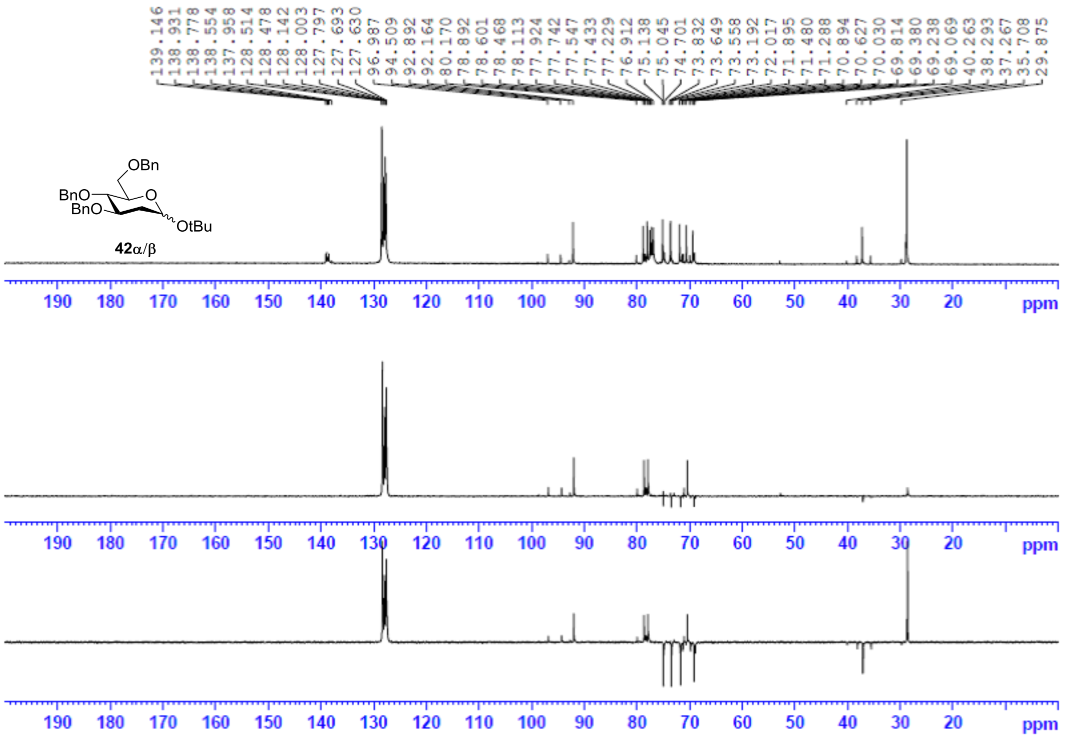
139.146
138.931
138.778
138.554
137.958
128.514
128.478
128.142
128.003
127.797
127.693
127.630
96.987
94.509
92.892
92.164
80.170
78.892
78.601
78.468
78.113
77.924
77.742
77.547
77.433
77.229
76.912
75.138
75.045
74.701
73.832
73.649
73.558
73.192
72.017
71.895
71.480
71.288
70.894
70.627
70.030
69.814
69.380
69.238
69.069
40.263
38.293
37.267
35.708
29.875
OBn
BnO
BnO
O
OtBu
42α/β
190
180
170
160
150
140
130
120
110
100
90
80
70
60
50
40
30
20
ppm

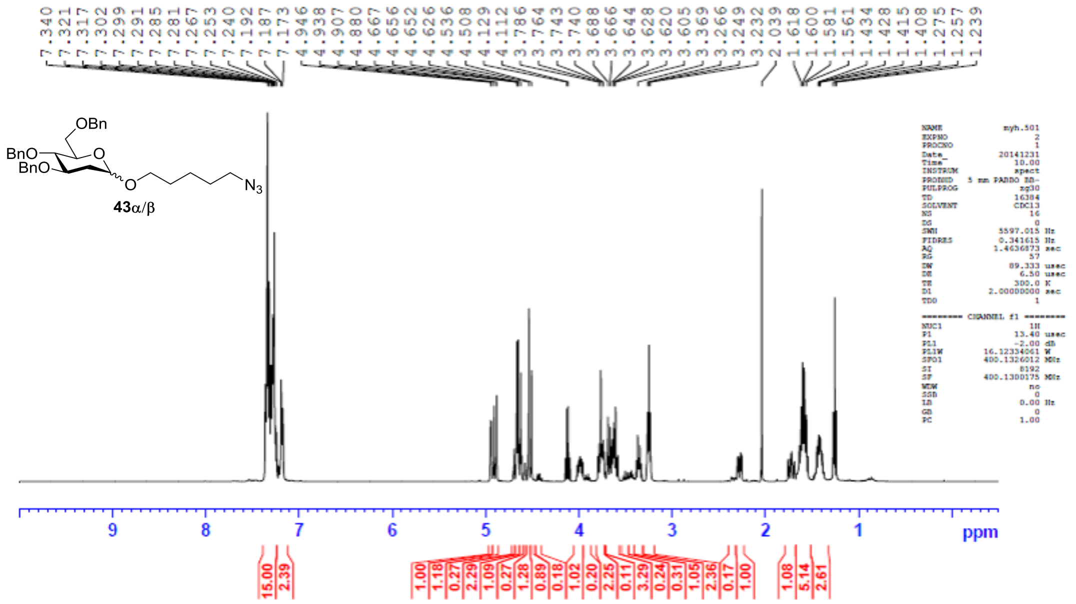
7.340
7.321
7.317
7.302
7.299
7.291
7.285
7.281
7.267
7.253
7.240
7.192
7.187
7.173
4.946
4.938
4.907
4.880
4.667
4.656
4.652
4.626
4.536
4.508
4.129
4.112
3.786
3.764
3.743
3.740
3.688
3.666
3.644
3.628
3.620
3.605
3.369
3.266
3.249
3.232
2.039
1.618
1.600
1.581
1.561
1.434
1.428
1.415
1.408
1.275
1.257
1.239
OBn
BnO
BnO
O
O
N3
43α/β
NAME myh.501
EXPNO 2
PROCNO 1
Date_ 20141231
SOLVENT CDCl3
9
8
7
6
5
4
3
2
1
ppm
15.00
2.39
1.00
1.18
0.27
2.29
1.09
0.27
1.28
0.89
0.18
1.02
0.20
2.25
0.11
3.29
0.24
0.31
1.05
2.36
0.17
1.00
1.08
5.14
2.61

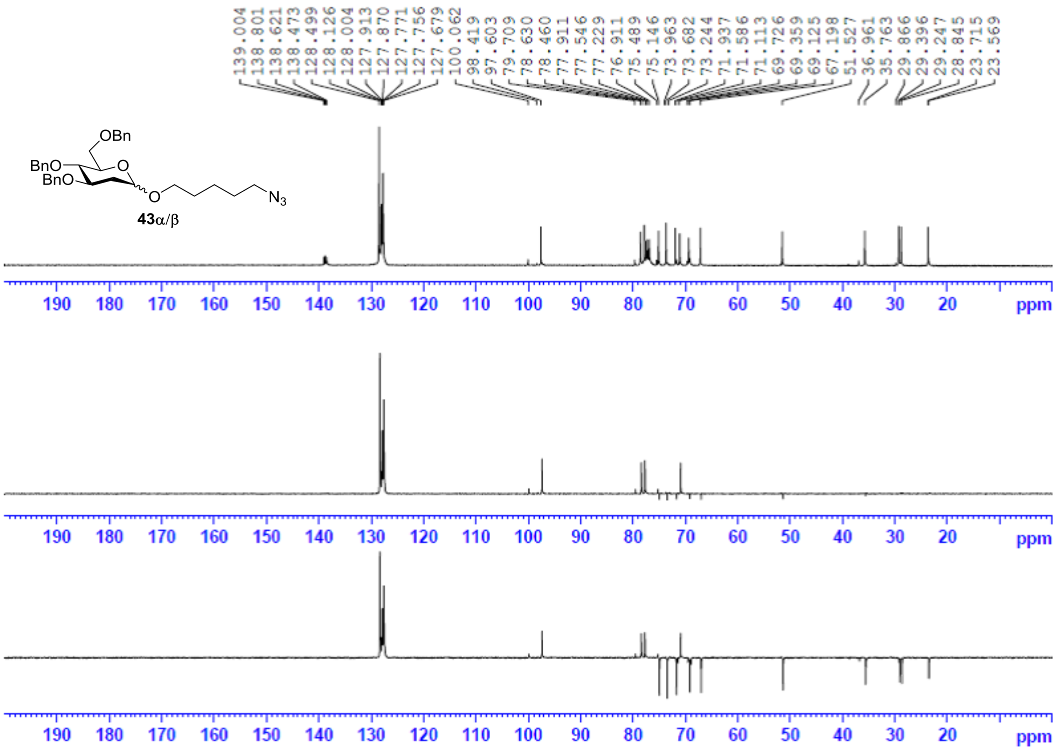
139.004
138.801
138.621
138.473
128.499
128.126
128.004
127.913
127.870
127.771
127.756
127.679
100.062
98.419
97.603
79.709
78.630
78.460
77.911
77.546
77.229
76.911
75.489
75.146
73.963
73.682
73.244
71.937
71.586
71.113
69.726
69.359
69.125
67.198
51.527
36.961
35.763
29.866
29.396
29.247
28.845
23.715
23.569
OBn
BnO
BnO
O
O
N_3
43α/β
ppm

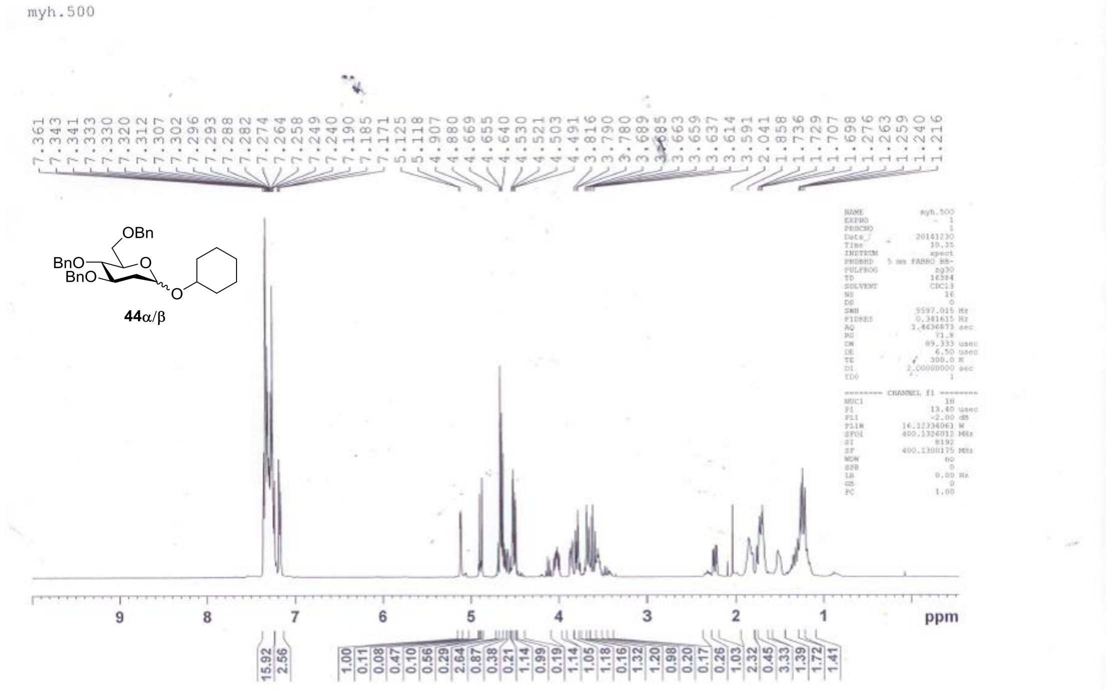
myh.500
7.361
7.343
7.341
7.333
7.330
7.320
7.312
7.307
7.302
7.296
7.293
7.288
7.282
7.274
7.264
7.258
7.249
7.240
7.190
7.185
7.171
5.125
5.118
4.907
4.880
4.669
4.655
4.640
4.530
4.521
4.503
4.491
3.816
3.790
3.780
3.689
3.685
3.663
3.659
3.637
3.614
3.591
2.041
1.858
1.736
1.729
1.707
1.698
1.276
1.263
1.259
1.240
1.216
OBn
BnO
BnO
O
O
44α/β
ppm
15.92
2.56
1.00
0.11
0.08
0.47
0.10
0.56
0.29
2.64
0.87
0.38
0.21
1.14
0.99
0.19
1.14
1.05
1.18
0.16
1.32
1.20
0.98
0.20
0.17
0.26
1.03
2.32
0.45
3.33
1.39
1.72
1.41

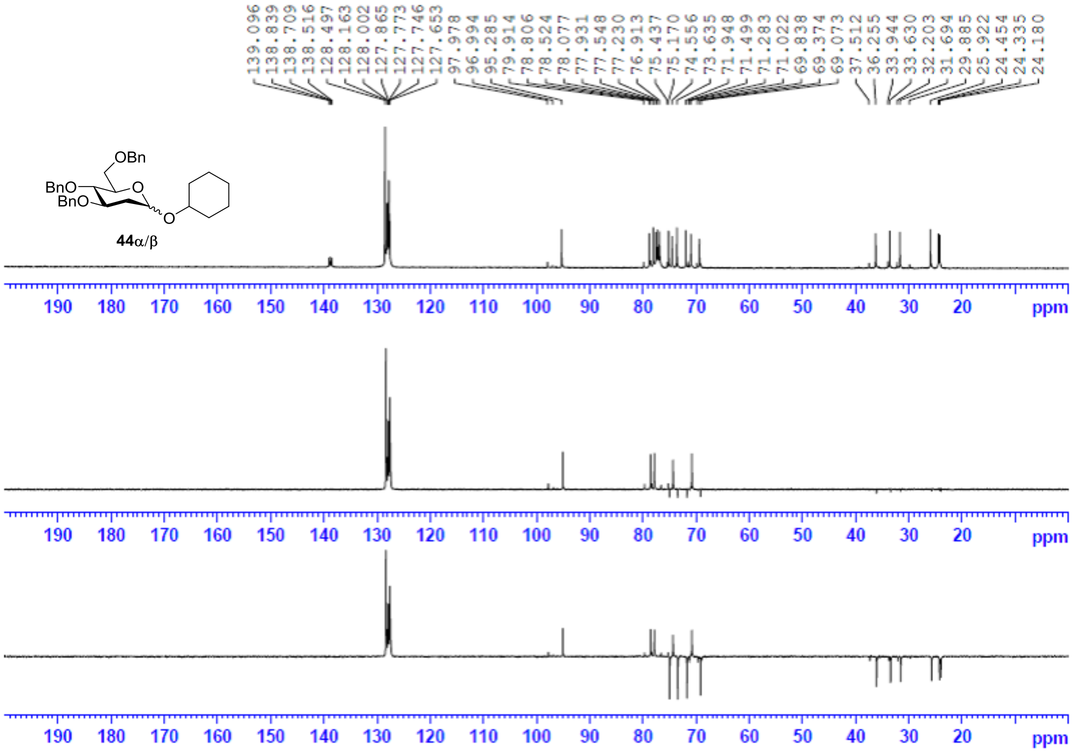
139.096
138.839
138.709
138.516
128.497
128.163
128.002
127.865
127.773
127.746
127.653
97.978
96.994
95.285
79.914
78.806
78.524
78.077
77.931
77.548
77.230
76.913
75.437
75.170
74.556
73.635
71.948
71.499
71.283
71.022
69.838
69.374
69.073
37.512
36.255
33.944
33.630
32.203
31.694
29.885
25.922
24.454
24.335
24.180
OBn
BnO
BnO
O
O
44α/β
190 180 170 160 150 140 130 120 110 100 90 80 70 60 50 40 30 20 ppm

myh.50602

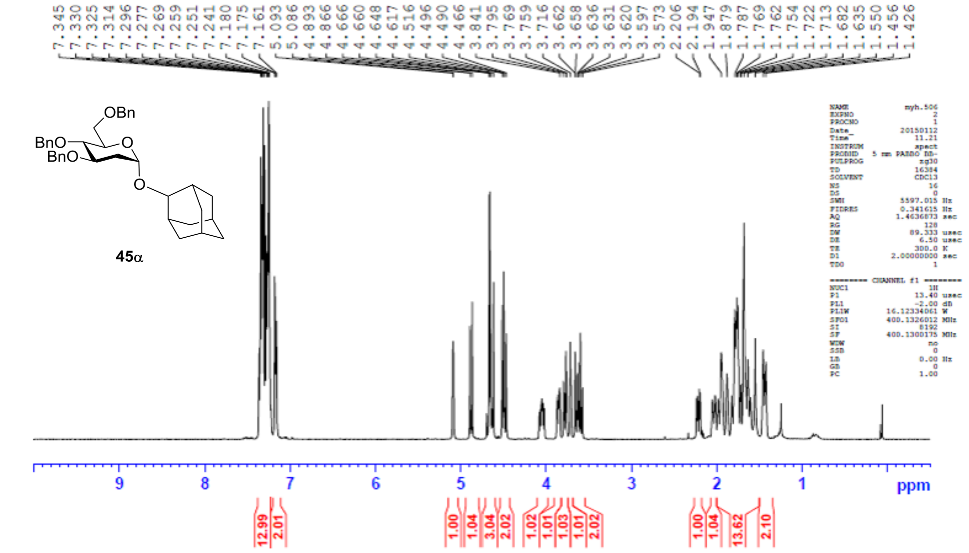
7.345
7.330
7.325
7.314
7.296
7.277
7.269
7.259
7.251
7.241
7.180
7.175
7.161
5.093
5.086
4.893
4.866
4.666
4.660
4.648
4.617
4.516
4.496
4.490
4.466
3.841
3.795
3.769
3.759
3.716
3.662
3.658
3.636
3.631
3.620
3.597
3.573
2.206
2.194
1.947
1.879
1.787
1.769
1.762
1.754
1.722
1.713
1.682
1.635
1.550
1.456
1.426
OBn
BnO
BnO
O
O
45α
9 8 7 6 5 4 3 2 1 ppm
12.99
2.01
1.00
1.04
3.04
2.02
1.02
1.01
1.03
1.01
2.02
1.00
1.04
13.62
2.10

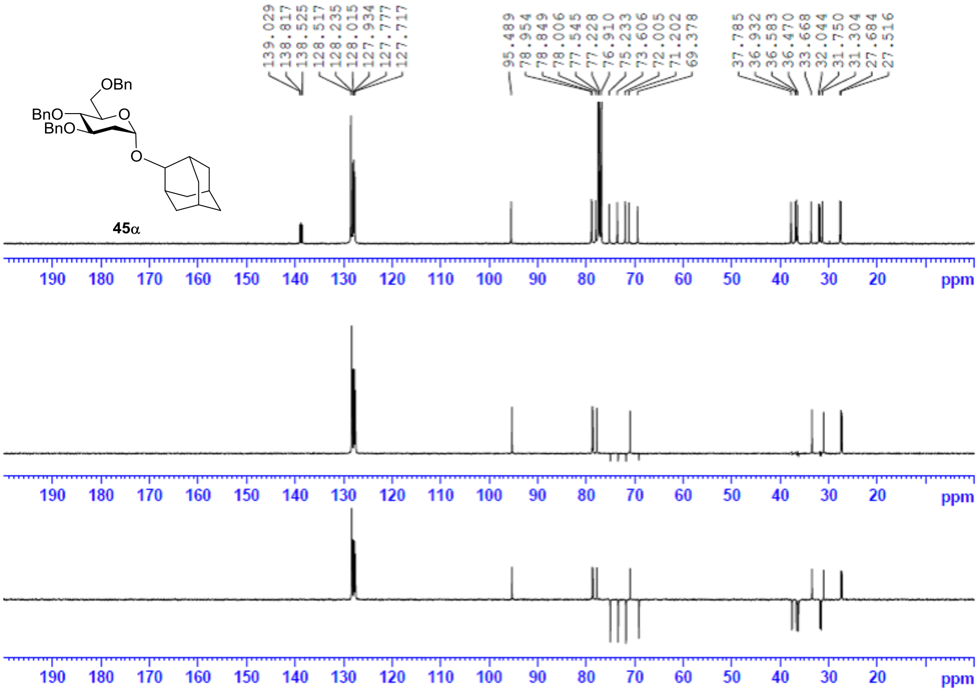
139.029
138.817
138.525
128.517
128.235
128.015
127.934
127.777
127.717
95.489
78.954
78.849
78.006
77.545
77.228
76.910
75.233
73.606
72.005
71.202
69.378
37.785
36.932
36.583
36.470
33.668
32.044
31.750
31.304
27.684
27.516
OBn
BnO
BnO
O
O
45α
190 180 170 160 150 140 130 120 110 100 90 80 70 60 50 40 30 20 ppm
190 180 170 160 150 140 130 120 110 100 90 80 70 60 50 40 30 20 ppm
190 180 170 160 150 140 130 120 110 100 90 80 70 60 50 40 30 20 ppm

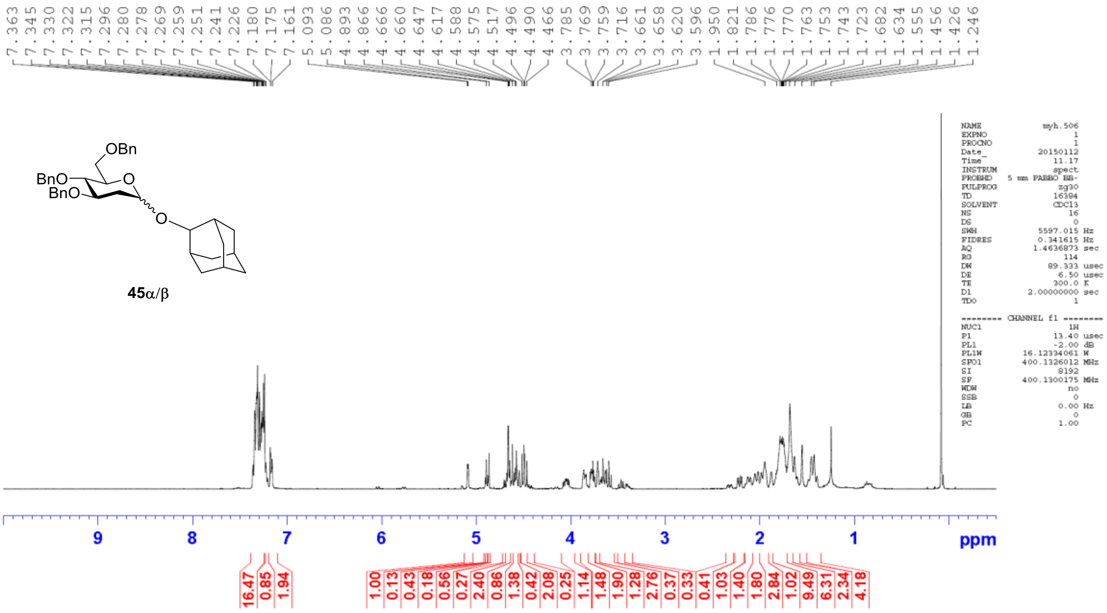
7.363
7.345
7.330
7.322
7.315
7.296
7.280
7.278
7.269
7.259
7.251
7.241
7.226
7.180
7.175
7.161
5.093
5.086
4.893
4.866
4.666
4.660
4.647
4.617
4.588
4.575
4.517
4.496
4.490
4.466
3.785
3.769
3.759
3.716
3.661
3.658
3.620
3.596
1.950
1.821
1.786
1.776
1.770
1.763
1.753
1.743
1.723
1.682
1.634
1.555
1.456
1.426
1.246
OBn
BnO
BnO
O
O
45α/β
NAME myh.506
EXPNO 1
PROCNO 1
Date_ 20150112
Time 11.17
INSTRUM spect
PROBHD 5 mm PABBO BB-
PULPROG zg30
TD 16384
SOLVENT CDCl3
NS 16
DS 0
SWH 5597.015 Hz
FIDRES 0.341615 Hz
AQ 1.4636873 sec
RG 114
DW 89.333 usec
DE 6.50 usec
TE 300.0 K
D1 2.00000000 sec
TD0 1
======== CHANNEL f1 ========
NUC1 1H
P1 13.40 usec
PL1 -2.00 dB
PL1W 16.12334061 W
SFO1 400.1326012 MHz
SI 8192
SF 400.1300175 MHz
WDW no
SSB 0
LB 0.00 Hz
GB 0
PC 1.00
9 8 7 6 5 4 3 2 1 ppm
16.47
0.85
1.94
1.00
0.13
0.43
0.18
0.56
0.27
2.40
0.86
1.38
0.42
2.08
0.25
1.14
1.48
1.90
1.28
2.76
0.37
0.33
0.41
1.03
1.40
1.80
2.84
1.02
9.49
6.31
2.34
4.18

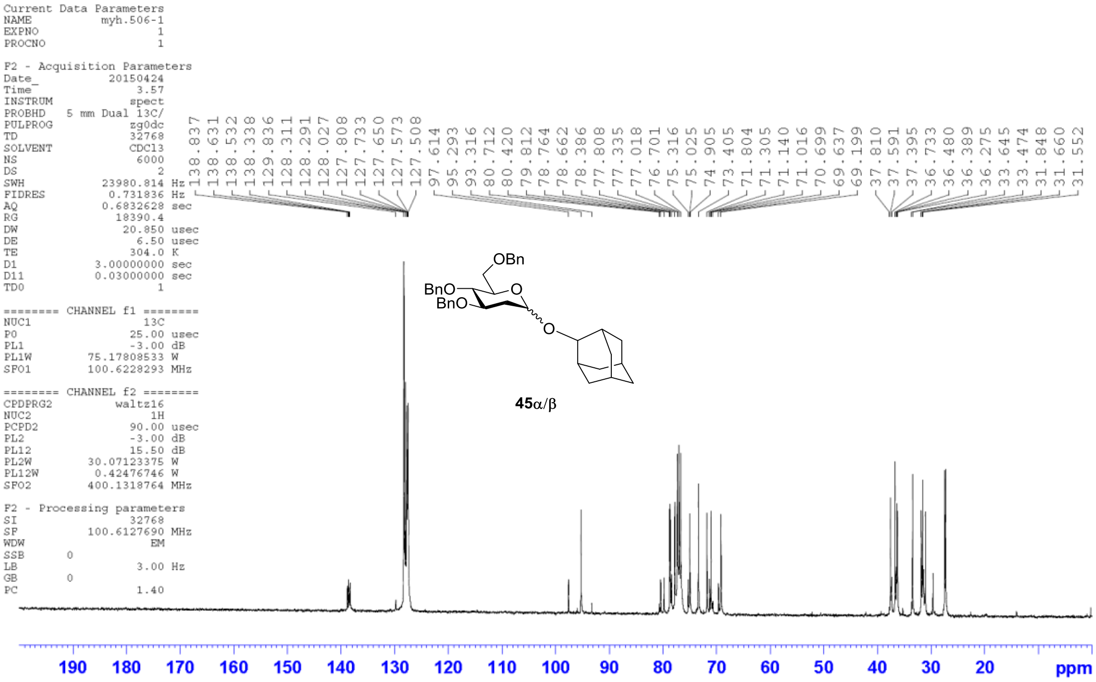
Current Data Parameters
NAME myh.506-1
EXPNO 1
PROCNO 1
F2 - Acquisition Parameters
Date_ 20150424
Time 3.57
INSTRUM spect
PROBHD 5 mm Dual 13C/
PULPROG zg0dc
TD 32768
SOLVENT CDCl3
NS 6000
DS 2
SWH 23980.814 Hz
FIDRES 0.731836 Hz
AQ 0.6832628 sec
RG 18390.4
DW 20.850 usec
DE 6.50 usec
TE 304.0 K
D1 3.00000000 sec
D11 0.03000000 sec
TD0 1
======== CHANNEL f1 ========
NUC1 13C
P0 25.00 usec
PL1 -3.00 dB
PL1W 75.17808533 W
SFO1 100.6228293 MHz
======== CHANNEL f2 ========
CPDPRG2 waltz16
NUC2 1H
PCPD2 90.00 usec
PL2 -3.00 dB
PL12 15.50 dB
PL2W 30.07123375 W
PL12W 0.42476746 W
SFO2 400.1318764 MHz
F2 - Processing parameters
SI 32768
SF 100.6127690 MHz
WDW EM
SSB 0
LB 3.00 Hz
GB 0
PC 1.40
138.837 138.631 138.532 138.338 129.836 128.311 128.291 128.027 127.808 127.733 127.650 127.573 127.508 97.614 95.293 93.316 80.712 80.420 79.812 78.764 78.662 78.386 77.335 77.018 76.701 75.316 75.025 74.905 73.405 71.804 71.305 71.140 71.016 70.699 69.637 69.199 37.810 37.591 37.395 36.733 36.480 36.389 36.275 33.645 33.474 31.848 31.660 31.552
OBn
BnO
BnO
O
O
45α/β
190 180 170 160 150 140 130 120 110 100 90 80 70 60 50 40 30 20 ppm

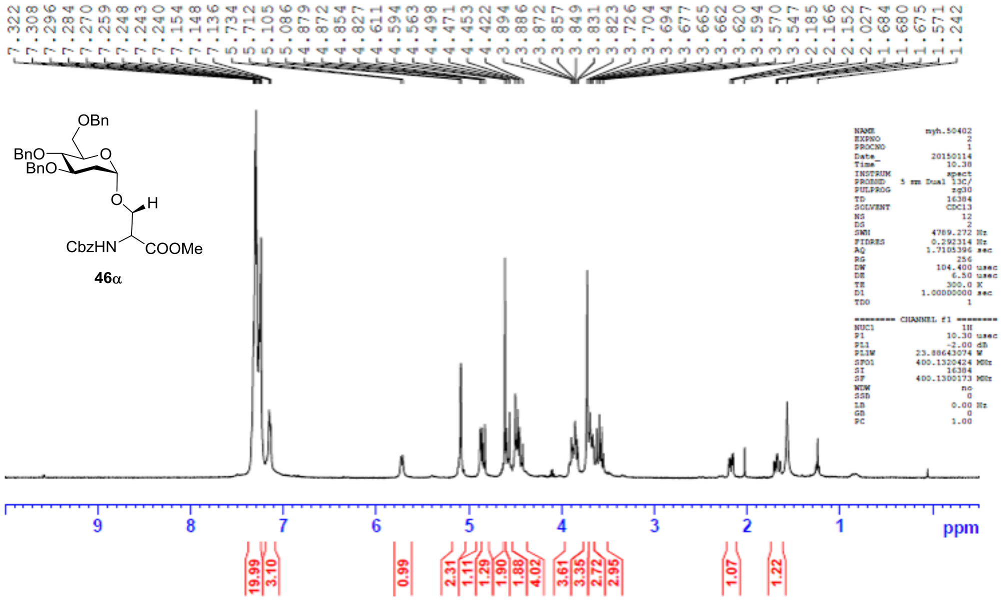
7.322 7.308 7.296 7.284 7.270 7.259 7.248 7.243 7.240 7.154 7.148 7.136 5.734 5.712 5.105 5.086 4.879 4.872 4.854 4.827 4.611 4.594 4.563 4.498 4.471 4.453 4.422 3.894 3.886 3.872 3.857 3.849 3.831 3.823 3.726 3.704 3.694 3.677 3.665 3.662 3.620 3.594 3.570 3.547 2.185 2.166 2.152 2.027 1.684 1.680 1.675 1.571 1.242
OBn
BnO
BnO
O
O
H
CbzHN
COOMe
46α
NAME myh.50402
EXPNO 2
PROCNO 1
Date_ 20150114
Time 10.38
INSTRUM spect
PROBHD 5 mm Dual 13C/
PULPROG zg30
TD 16384
SOLVENT CDCl3
NS 12
DS 2
SWH 4789.272 Hz
FIDRES 0.292314 Hz
AQ 1.7105396 sec
RG 256
DW 104.400 usec
DE 6.50 usec
TE 300.0 K
D1 1.00000000 sec
TD0 1
======== CHANNEL f1 ========
NUC1 1H
P1 10.30 usec
PL1 -2.00 dB
PL1W 23.88643074 W
SFO1 400.1320424 MHz
SI 16384
SF 400.1300173 MHz
WDW no
SSB 0
LB 0.00 Hz
GB 0
PC 1.00
9 8 7 6 5 4 3 2 1 ppm
19.99 3.10 0.99 2.31 1.11 1.29 1.90 1.88 4.02 3.61 3.35 2.72 2.95 1.07 1.22

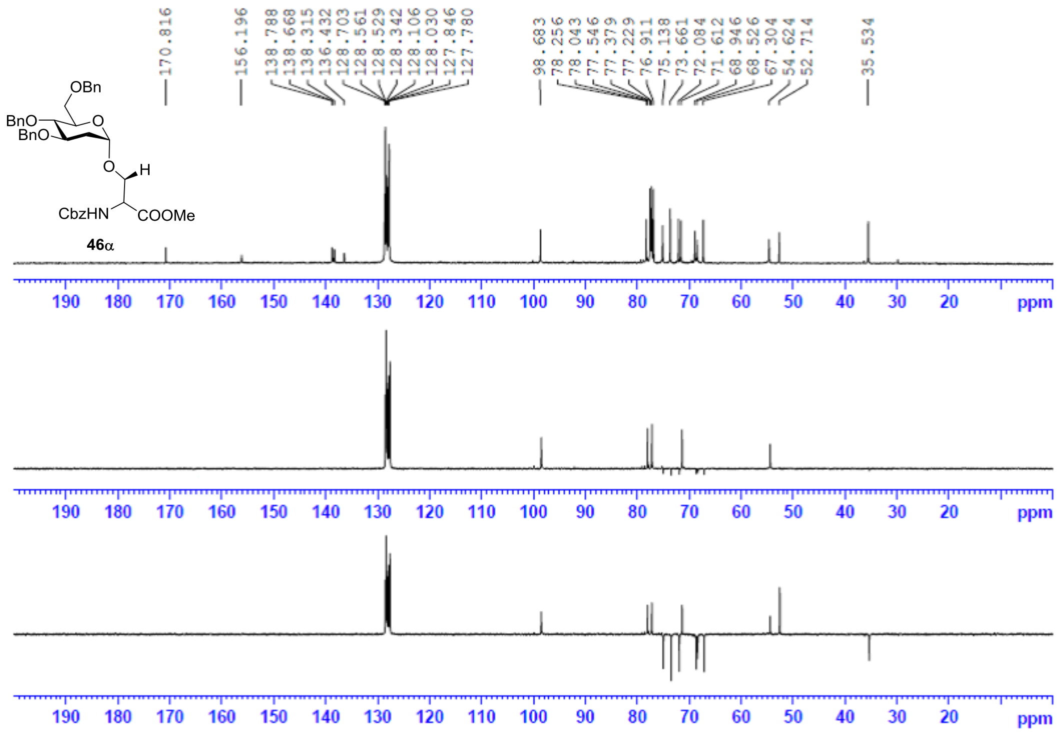
170.816
156.196
138.788
138.668
138.315
136.432
128.703
128.561
128.529
128.342
128.106
128.030
127.846
127.780
98.683
78.256
78.043
77.546
77.379
77.229
76.911
75.138
73.661
72.084
71.612
68.946
68.526
67.304
54.624
52.714
35.534
OBn
BnO
BnO
O
O
H
CbzHN
COOMe
46α
ppm
ppm
ppm

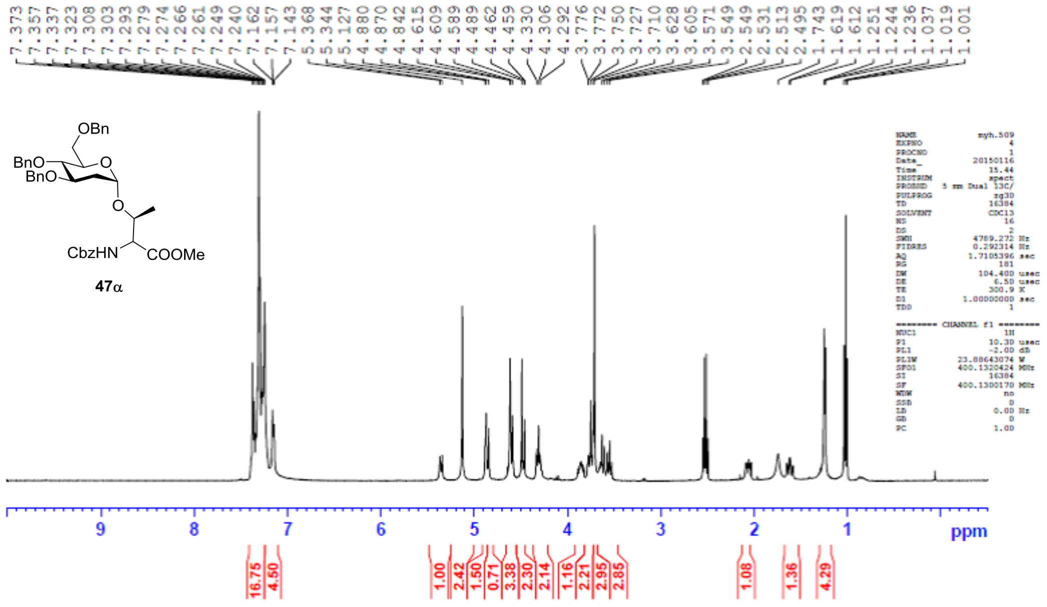
7.373
7.357
7.337
7.323
7.308
7.303
7.293
7.279
7.274
7.266
7.261
7.249
7.240
7.162
7.157
7.143
5.368
5.344
5.127
4.880
4.870
4.842
4.615
4.609
4.589
4.489
4.462
4.459
4.330
4.306
4.292
3.776
3.772
3.750
3.727
3.710
3.628
3.605
3.571
3.549
2.549
2.531
2.513
2.495
1.743
1.619
1.612
1.251
1.244
1.236
1.037
1.019
1.001
OBn
BnO
BnO
O
O
CbzHN
COOMe
47α
ppm
16.75
4.50
1.00
2.42
1.50
0.71
3.38
2.30
2.14
1.16
2.21
2.95
2.85
1.08
1.36
4.29

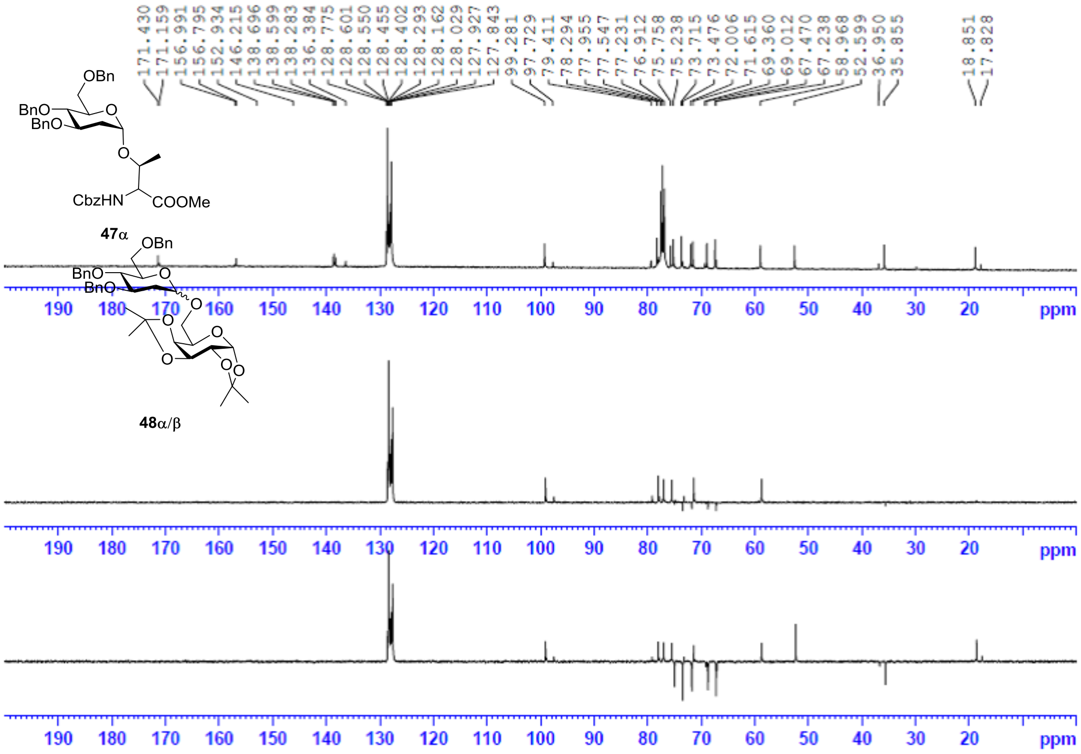

47α

48α/β

171.430
171.159
156.991
156.795
152.934
146.215
138.696
138.599
138.283
136.384
128.775
128.601
128.550
128.455
128.402
128.293
128.162
128.029
127.927
127.843
99.281
97.729
79.411
78.294
77.955
77.547
77.231
76.912
75.758
75.238
73.715
73.476
72.006
71.615
69.360
69.012
67.470
67.238
58.968
52.599
36.950
35.855
18.851
17.828
190 180 170 160 150 140 130 120 110 100 90 80 70 60 50 40 30 20 ppm

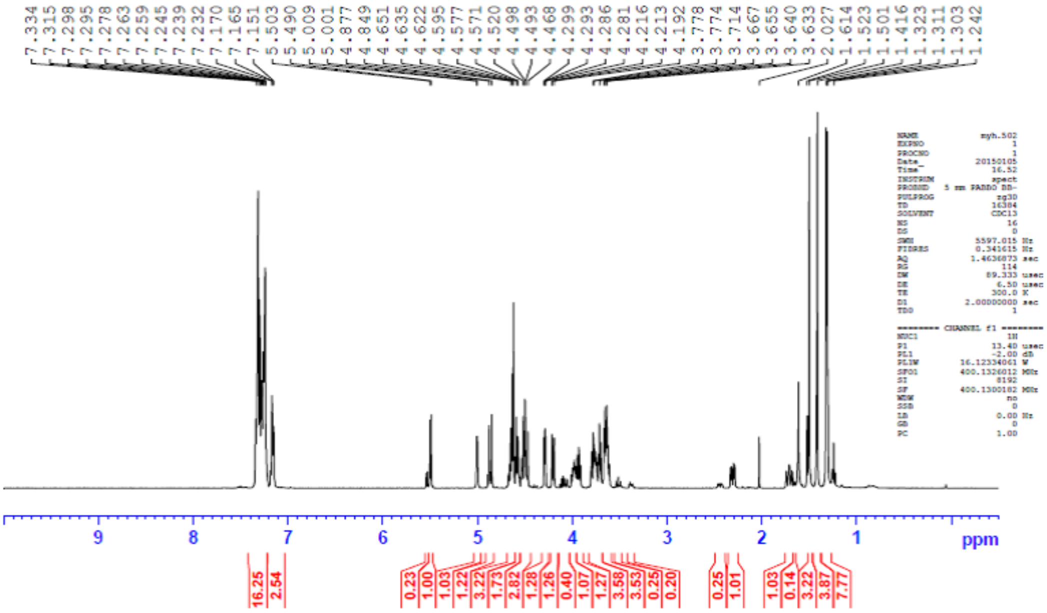
7.334
7.315
7.298
7.295
7.278
7.263
7.259
7.245
7.239
7.232
7.170
7.165
7.151
5.503
5.490
5.009
5.001
4.877
4.849
4.651
4.635
4.622
4.595
4.577
4.571
4.520
4.498
4.493
4.468
4.299
4.293
4.286
4.281
4.216
4.213
4.192
3.778
3.774
3.714
3.667
3.655
3.640
3.633
2.027
1.614
1.523
1.501
1.416
1.323
1.311
1.303
1.242
9 8 7 6 5 4 3 2 1 ppm
16.25
2.54
0.23
1.00
1.03
1.22
3.22
1.73
2.82
1.28
1.26
0.40
1.07
1.27
3.58
3.53
0.25
0.20
0.25
1.01
1.03
0.14
3.22
3.87
7.77

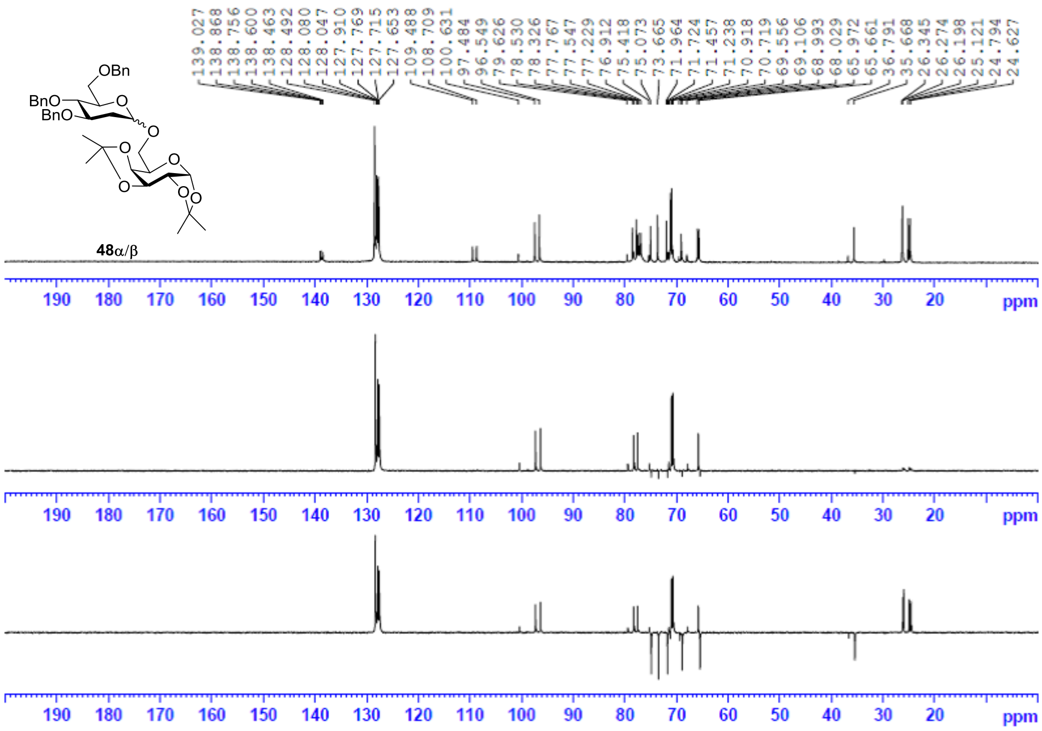
48α/β
ppm

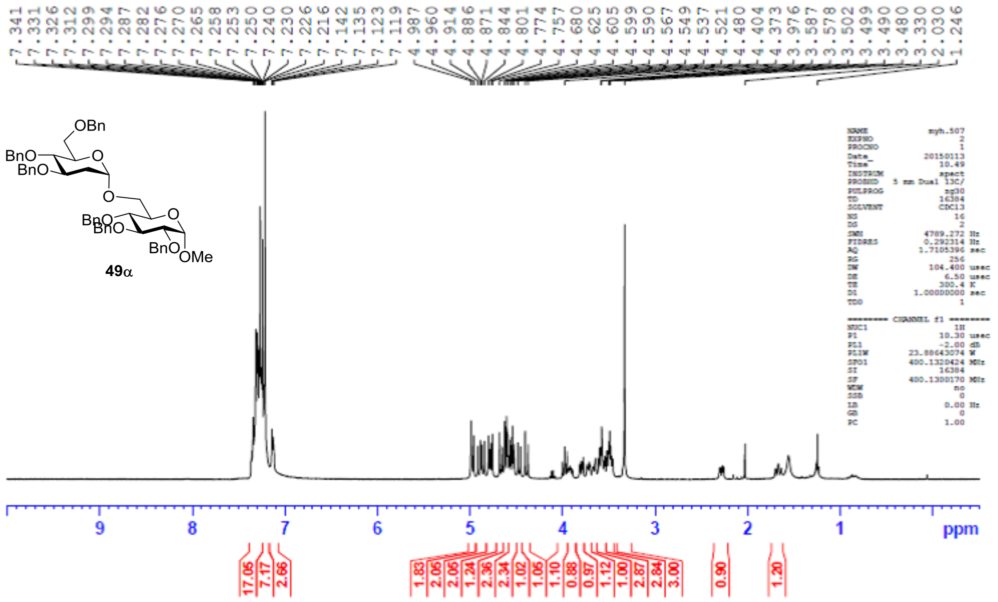
49α
ppm

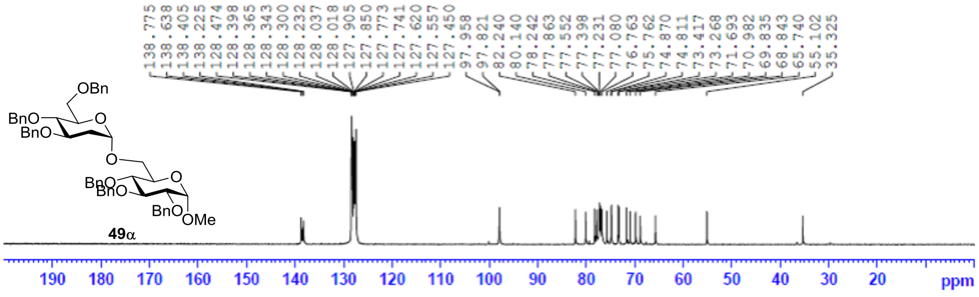
138.775
138.638
138.405
138.225
128.474
128.398
128.365
128.343
128.300
128.232
128.037
128.018
127.905
127.850
127.773
127.741
127.620
127.557
127.450
97.958
97.821
82.240
80.140
78.242
77.863
77.552
77.398
77.231
77.080
76.763
75.762
74.870
74.811
73.417
73.268
71.693
70.982
69.835
68.843
65.740
55.102
35.325
OBn
BnO
BnO
O
O
BnO
BnO
O
BnO
OMe
49α
190 180 170 160 150 140 130 120 110 100 90 80 70 60 50 40 30 20 ppm

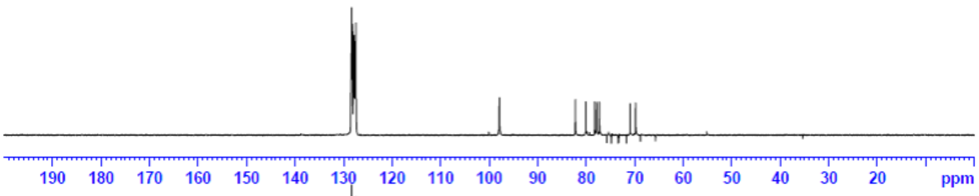
190 180 170 160 150 140 130 120 110 100 90 80 70 60 50 40 30 20 ppm

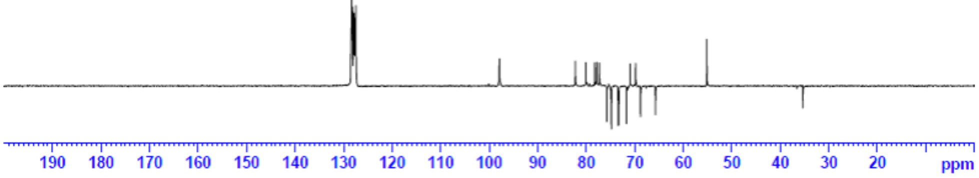
190 180 170 160 150 140 130 120 110 100 90 80 70 60 50 40 30 20 ppm

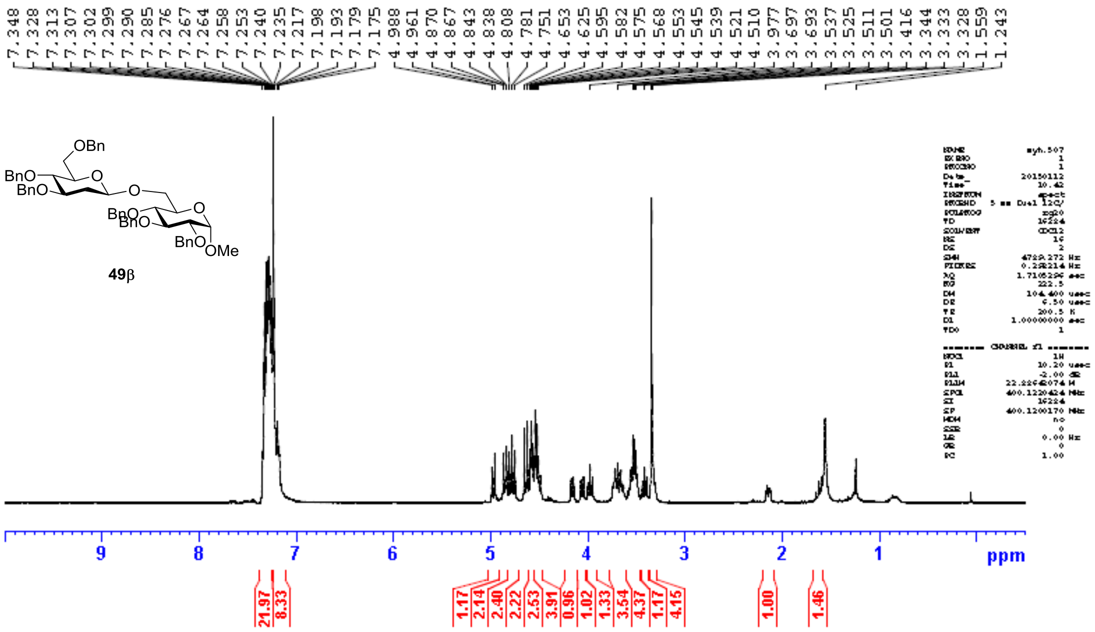
7.348
7.328
7.313
7.307
7.302
7.299
7.290
7.285
7.276
7.267
7.264
7.258
7.253
7.240
7.235
7.217
7.198
7.193
7.179
7.175
4.988
4.961
4.870
4.867
4.843
4.838
4.808
4.781
4.751
4.653
4.625
4.595
4.582
4.575
4.568
4.553
4.545
4.539
4.521
4.510
3.977
3.697
3.693
3.537
3.525
3.511
3.501
3.416
3.344
3.333
3.328
1.559
1.243
OBn
BnO
BnO
O
O
BnO
BnO
O
BnO
OMe
49β
9 8 7 6 5 4 3 2 1 ppm
21.97
8.33
1.17
2.14
2.40
2.22
2.53
3.91
0.96
1.02
1.33
3.54
4.37
1.17
4.15
1.00
1.46

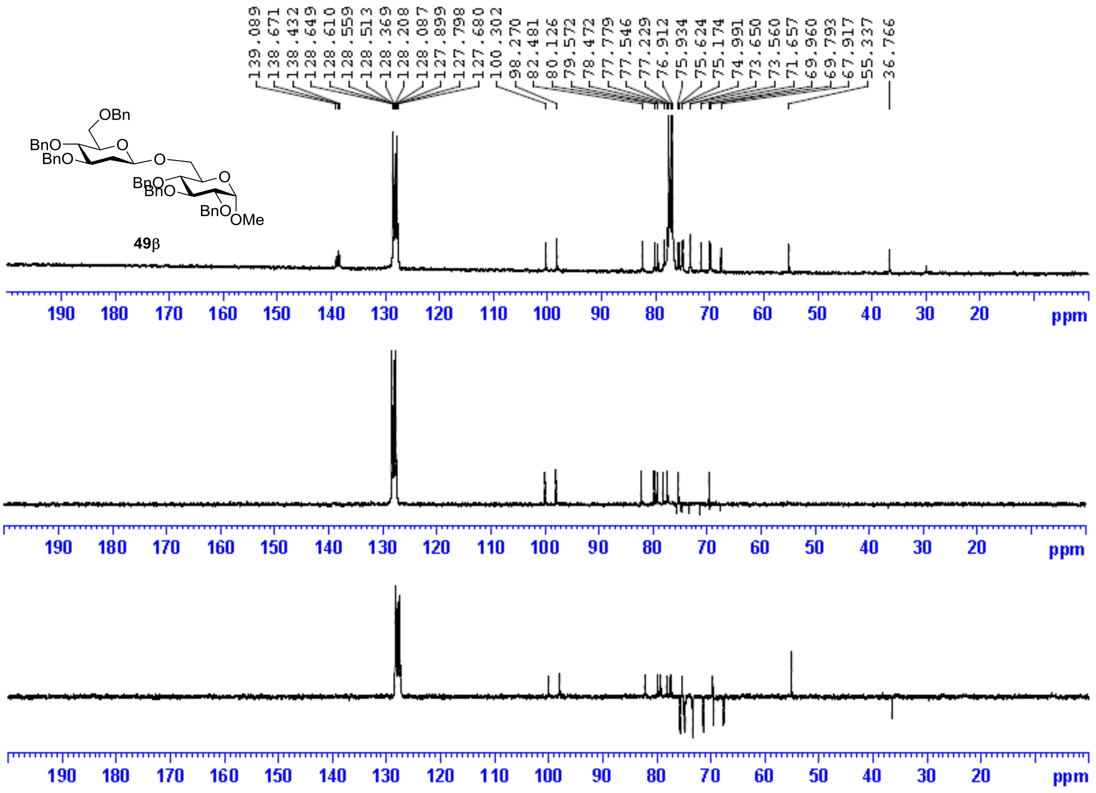
139.089
138.671
138.432
128.649
128.610
128.559
128.513
128.369
128.208
128.087
127.899
127.798
127.680
100.302
98.270
82.481
80.126
79.572
78.472
77.779
77.546
77.229
76.912
75.934
75.624
75.174
74.991
73.650
73.560
71.657
69.960
69.793
67.917
55.337
36.766
OBn
BnO
BnO
O
O
BnO
BnO
O
BnO
OMe
49β
ppm

myh.510-1

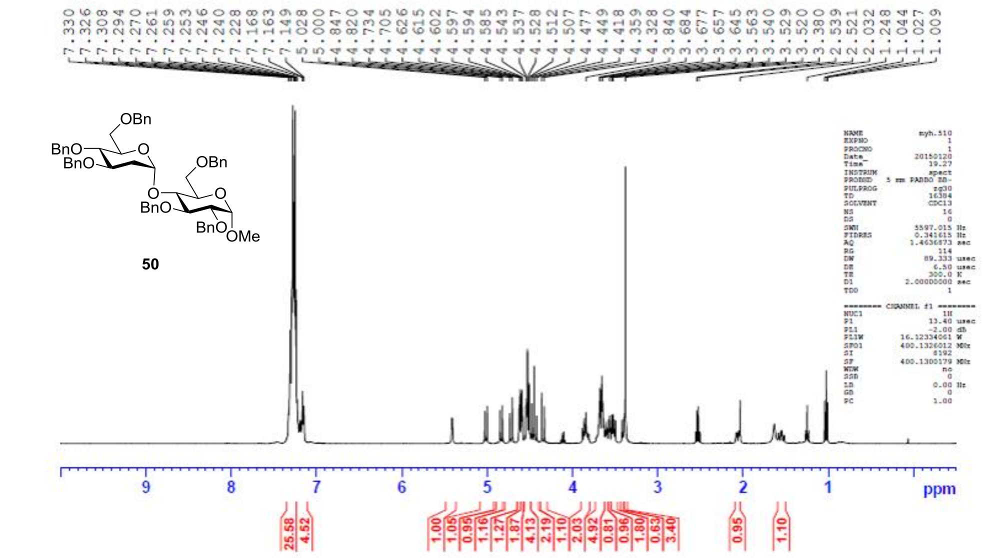
7.330
7.326
7.308
7.294
7.270
7.261
7.259
7.253
7.246
7.240
7.228
7.163
7.149
5.028
5.000
4.847
4.820
4.734
4.705
4.626
4.615
4.602
4.597
4.594
4.585
4.543
4.537
4.528
4.512
4.507
4.477
4.449
4.418
4.359
4.328
3.840
3.684
3.677
3.657
3.645
3.563
3.540
3.529
3.520
3.380
2.539
2.521
2.032
1.248
1.044
1.027
1.009
OBn
BnO
BnO
O
O
OBn
BnO
BnO
OMe
50
ppm
25.58
4.52
1.00
1.06
0.95
1.16
1.27
1.87
4.13
2.19
1.10
2.03
4.92
0.81
0.96
1.80
0.63
3.40
0.95
1.10

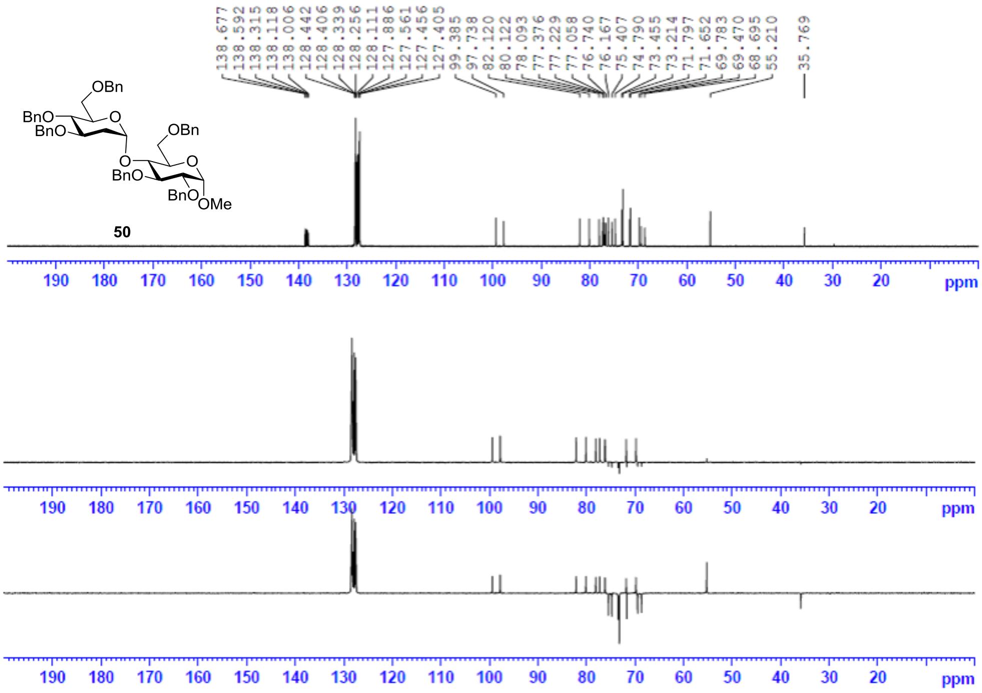
138.677
138.592
138.315
138.118
138.006
128.442
128.406
128.339
128.256
128.111
127.886
127.561
127.456
127.405
99.385
97.738
82.120
80.122
78.093
77.376
77.229
77.058
76.740
76.167
75.407
74.790
73.455
73.214
71.797
71.652
69.783
69.470
68.695
55.210
35.769
OBn
BnO
BnO
O
O
OBn
O
BnO
BnO
OMe
50
190 180 170 160 150 140 130 120 110 100 90 80 70 60 50 40 30 20 ppm

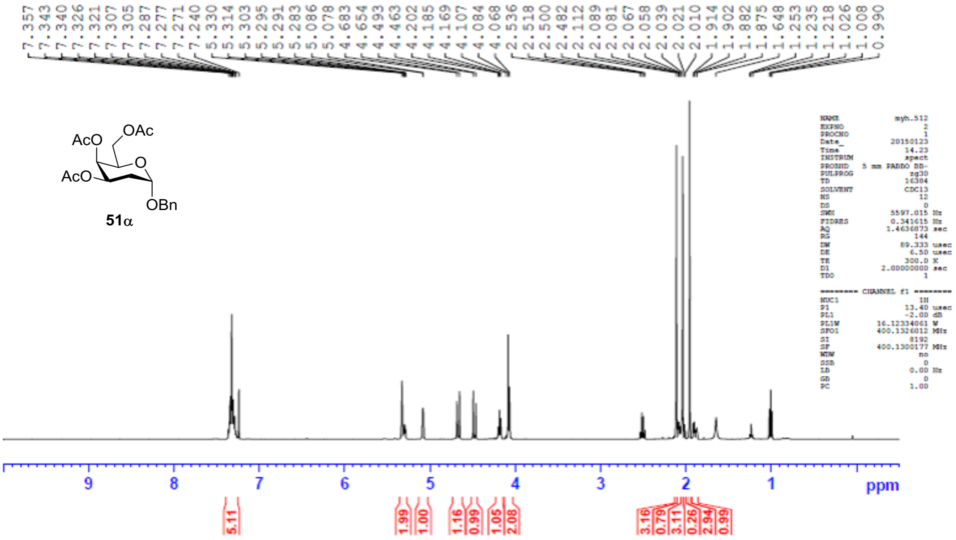
7.357
7.343
7.340
7.326
7.321
7.307
7.305
7.287
7.277
7.271
7.240
5.330
5.314
5.303
5.295
5.291
5.283
5.086
5.078
4.683
4.654
4.493
4.463
4.202
4.185
4.169
4.107
4.084
4.068
2.536
2.518
2.500
2.482
2.112
2.089
2.081
2.067
2.058
2.039
2.021
2.010
1.914
1.902
1.882
1.875
1.648
1.253
1.235
1.218
1.026
1.008
0.990
AcO
OAc
O
AcO
OBn
51α
9 8 7 6 5 4 3 2 1 ppm
5.11
1.99
1.00
1.16
0.99
1.05
2.08
3.16
0.79
3.11
0.26
2.94
0.99

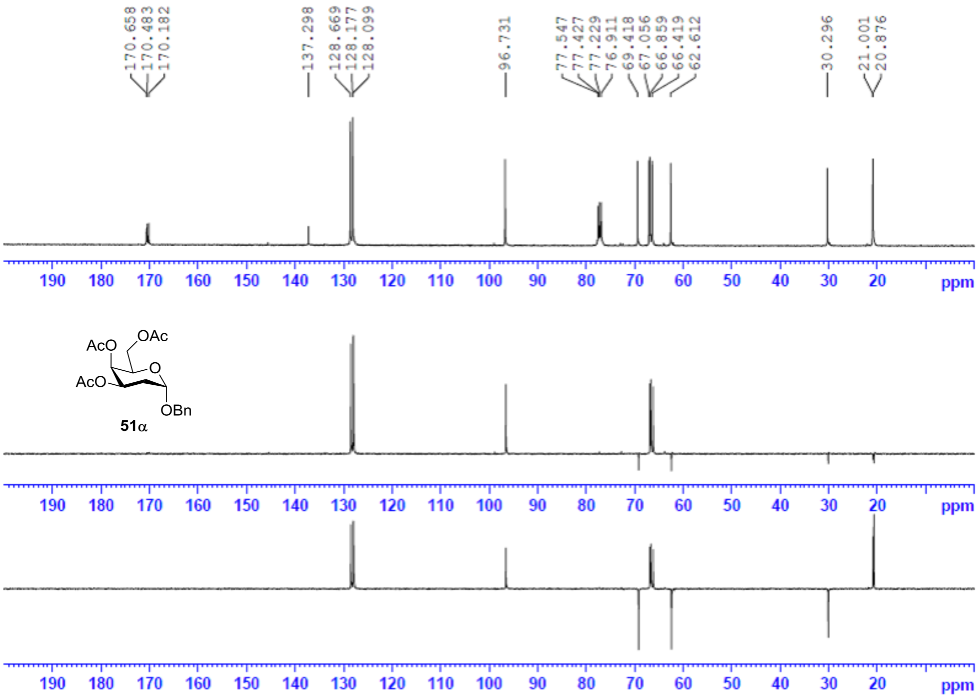
AcO
OAc
AcO
O
OBn
51α
ppm

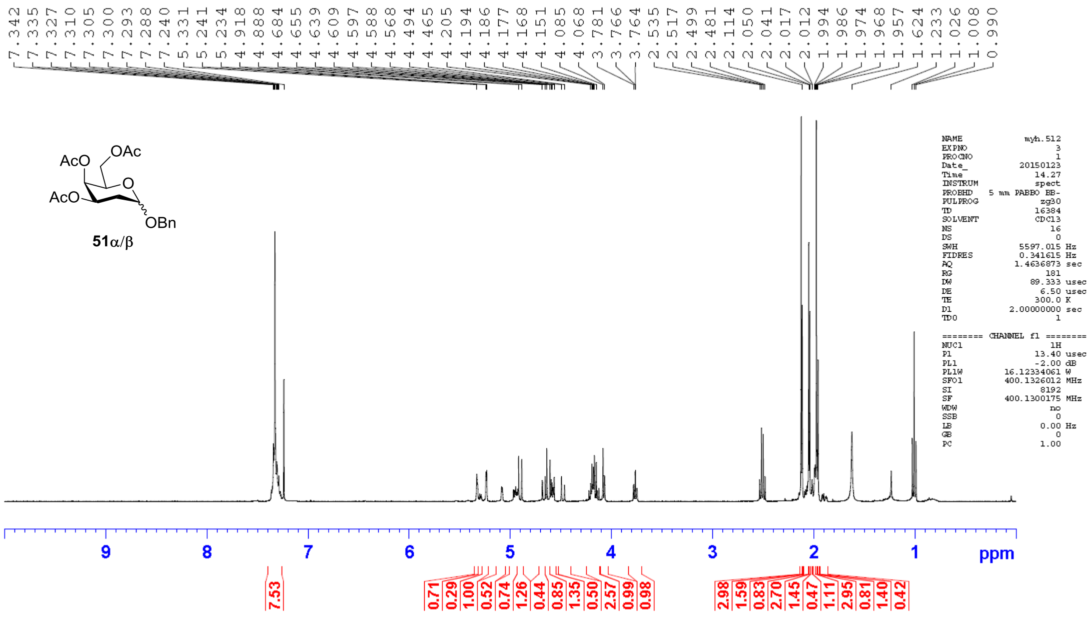
AcO
OAc
AcO
O
OBn
51α/β
ppm

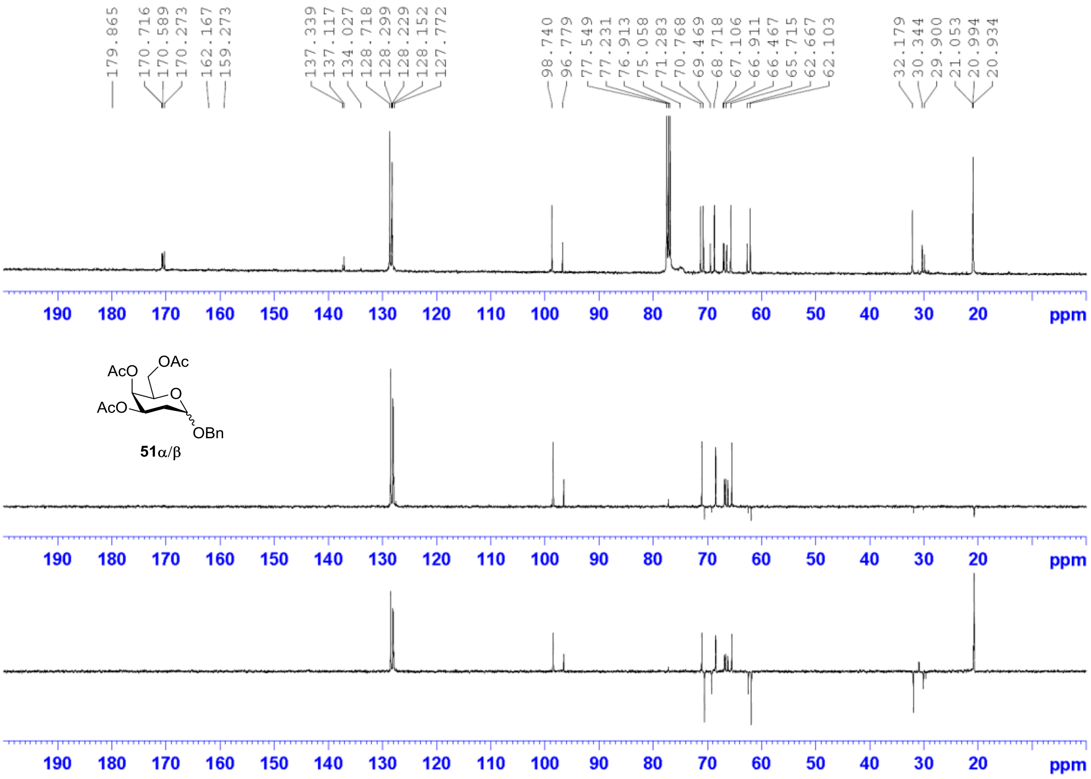
179.865
170.716
170.589
170.273
162.167
159.273
137.339
137.117
134.027
128.718
128.299
128.229
128.152
127.772
98.740
96.779
77.549
77.231
76.913
75.058
71.283
70.768
69.469
68.718
67.106
66.911
66.467
65.715
62.667
62.103
32.179
30.344
29.900
21.053
20.994
20.934
ppm
AcO
OAc
O
AcO
OBn
51α/β

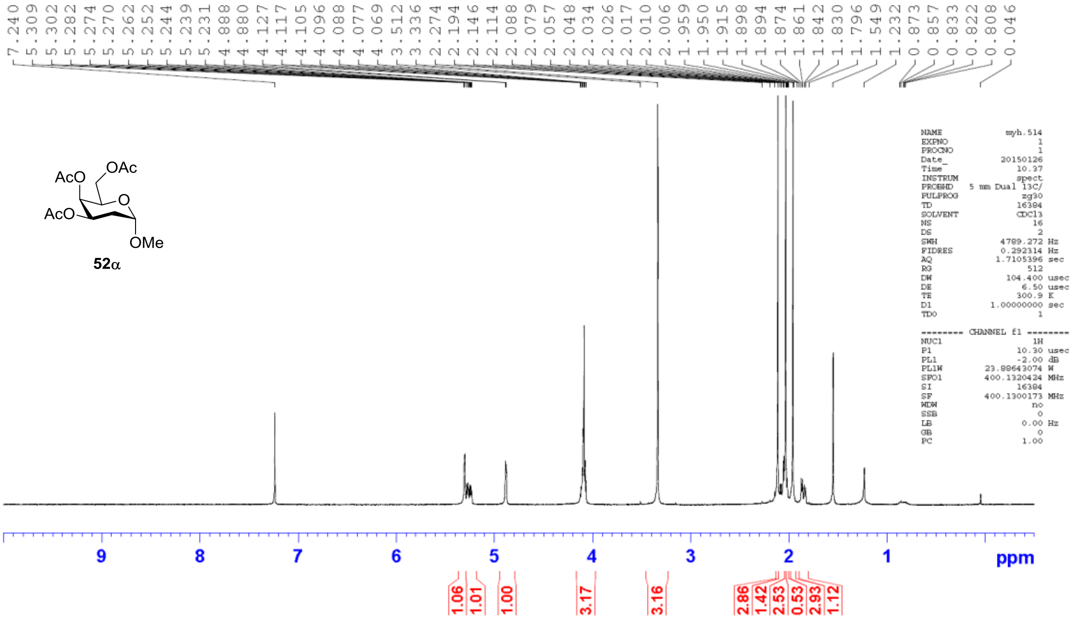
7.240
5.309
5.302
5.282
5.274
5.270
5.262
5.252
5.244
5.239
5.231
4.888
4.880
4.127
4.117
4.105
4.096
4.088
4.077
4.069
3.512
3.336
2.274
2.194
2.146
2.114
2.088
2.079
2.057
2.048
2.034
2.026
2.017
2.010
2.006
1.959
1.950
1.915
1.898
1.894
1.874
1.861
1.842
1.830
1.796
1.549
1.232
0.873
0.857
0.833
0.822
0.808
0.046
AcO
OAc
O
AcO
OMe
52α
NAME myh.514
EXPNO 1
PROCNO 1
Date_ 20150126
Time 10.37
INSTRUM spect
PROBHD 5 mm Dual 13C/
PULPROG zg30
TD 16384
SOLVENT CDCl3
NS 16
DS 2
SWH 4789.272 Hz
FIDRES 0.292314 Hz
AQ 1.7105396 sec
RG 512
DW 104.400 usec
DE 6.50 usec
TE 300.9 K
D1 1.00000000 sec
TD0 1
======== CHANNEL f1 ========
NUC1 1H
P1 10.30 usec
PL1 -2.00 dB
PL1W 23.88643074 W
SFO1 400.1320424 MHz
SI 16384
SF 400.1300173 MHz
WDW no
SSB 0
LB 0.00 Hz
GB 0
PC 1.00
ppm
1.06
1.01
1.00
3.17
3.16
2.86
1.42
2.53
0.53
2.93
1.12

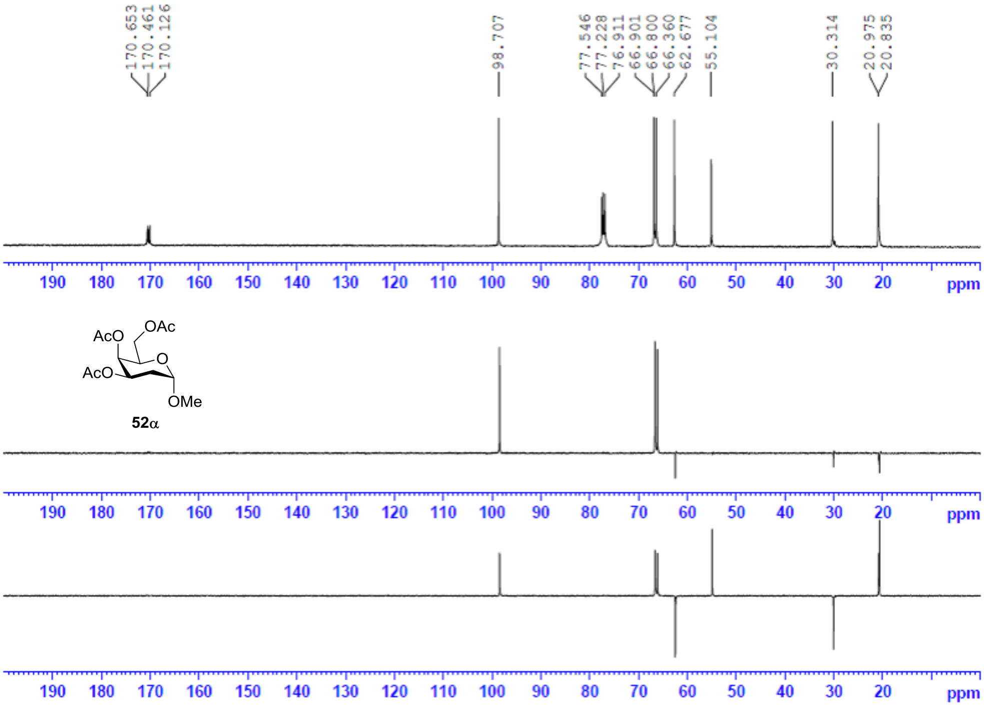
170.653
170.461
170.126
98.707
77.546
77.228
76.911
66.901
66.800
66.360
62.677
55.104
30.314
20.975
20.835
ppm
AcO
OAc
AcO
O
OMe
52α

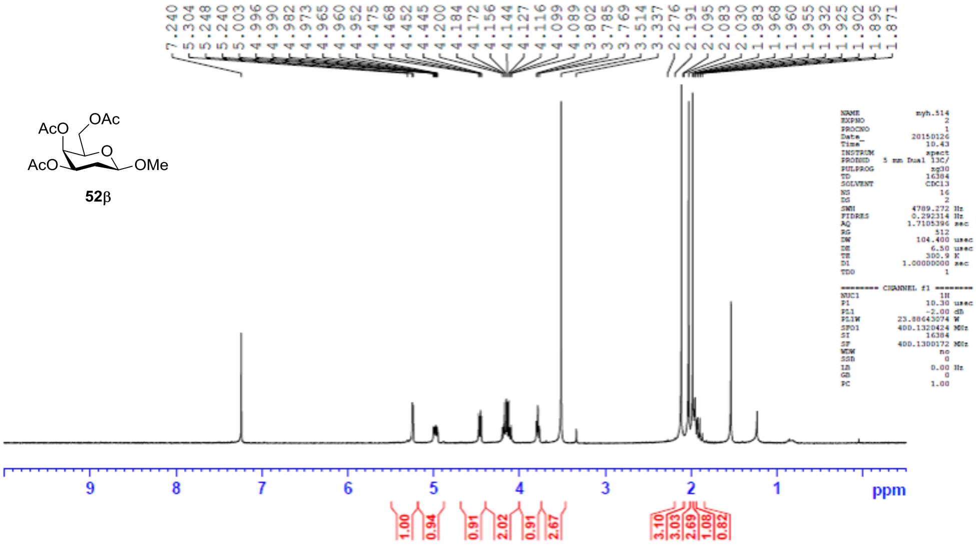
7.240
5.304
5.248
5.240
5.003
4.996
4.990
4.982
4.973
4.965
4.960
4.952
4.475
4.468
4.452
4.445
4.200
4.184
4.172
4.156
4.144
4.127
4.116
4.099
4.089
3.802
3.785
3.769
3.514
3.337
2.276
2.191
2.095
2.083
2.030
1.983
1.968
1.960
1.955
1.932
1.925
1.902
1.895
1.871
AcO
OAc
AcO
O
OMe
52β
ppm
1.00
0.94
0.91
2.02
0.91
2.67
3.10
3.03
2.69
1.08
0.82

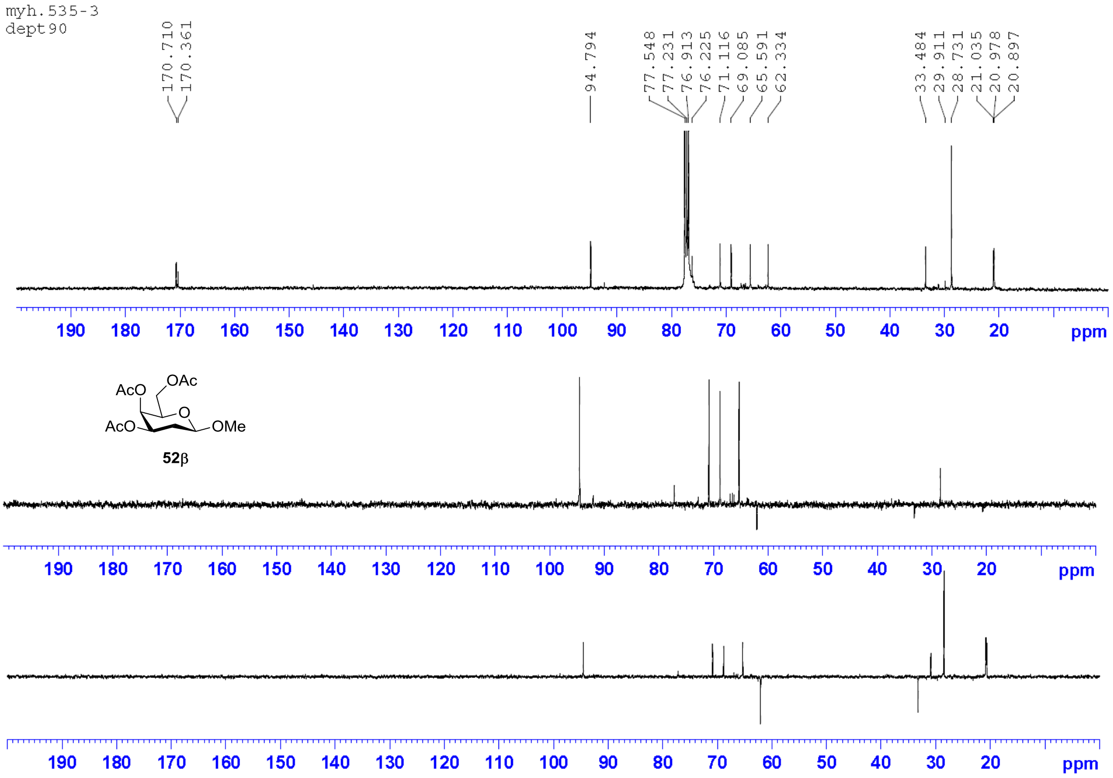
myh.535-3
dept90
170.710
170.361
94.794
77.548
77.231
76.913
76.225
71.116
69.085
65.591
62.334
33.484
29.911
28.731
21.035
20.978
20.897
190 180 170 160 150 140 130 120 110 100 90 80 70 60 50 40 30 20 ppm
AcO
OAc
O
AcO
OMe
52β

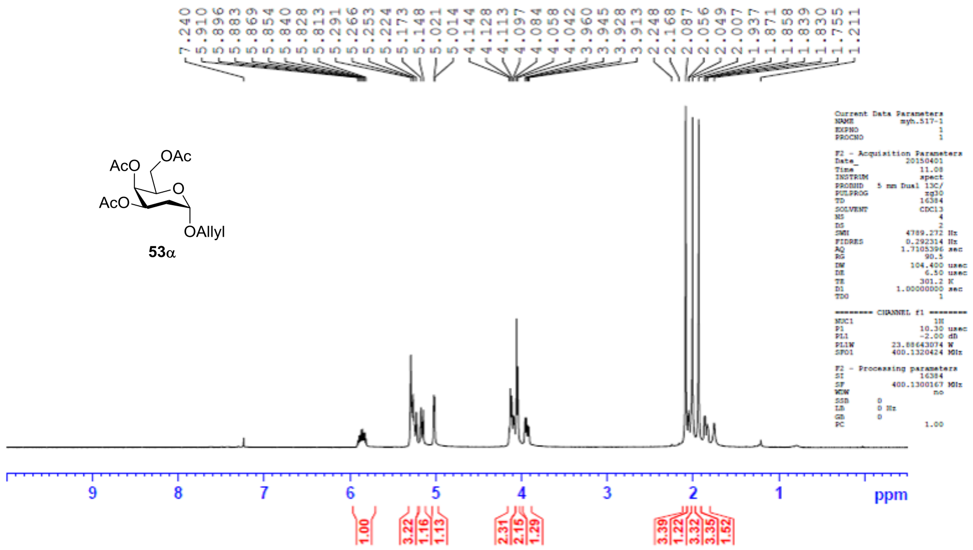
7.240
5.910
5.896
5.883
5.869
5.854
5.840
5.828
5.813
5.291
5.266
5.253
5.224
5.173
5.148
5.021
5.014
4.144
4.128
4.113
4.097
4.084
4.058
4.042
3.960
3.945
3.928
3.913
2.248
2.168
2.087
2.056
2.049
2.007
1.937
1.871
1.858
1.839
1.830
1.755
1.211
AcO
OAc
O
AcO
OAllyl
53α
9 8 7 6 5 4 3 2 1 ppm
1.00
3.22
1.16
1.13
2.31
2.15
1.29
3.39
1.22
3.32
3.35
1.52
Current Data Parameters
NAME myh.517-1
EXPNO 1
PROCNO 1

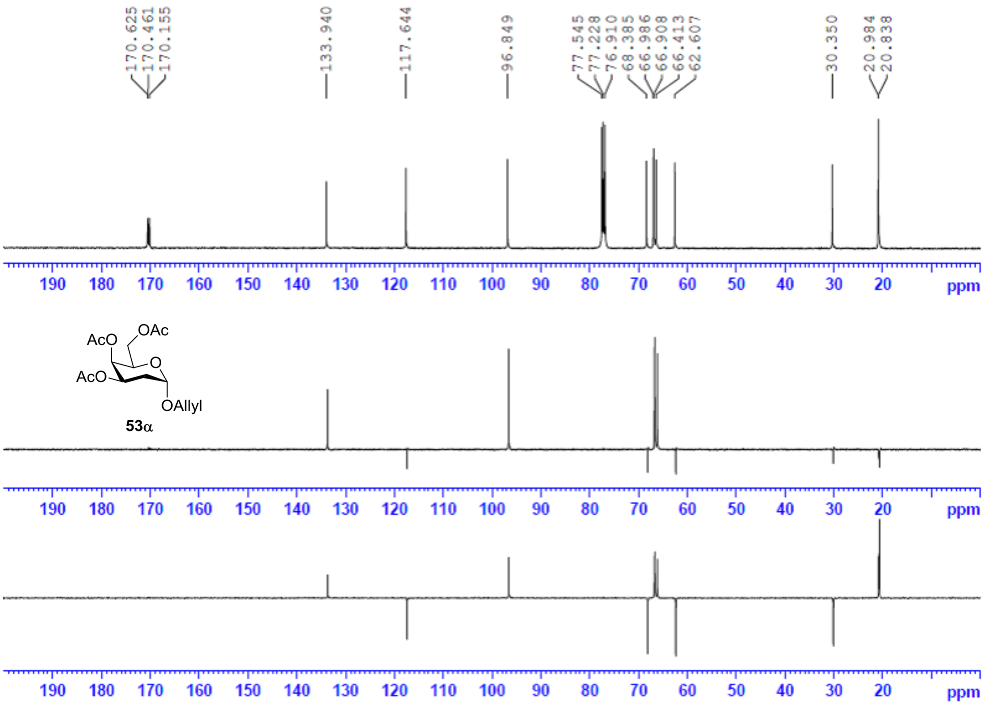
170.625
170.461
170.155
133.940
117.644
96.849
77.545
77.228
76.910
68.385
66.986
66.908
66.413
62.607
30.350
20.984
20.838
190 180 170 160 150 140 130 120 110 100 90 80 70 60 50 40 30 20 ppm
AcO
OAc
O
AcO
OAllyl
53α

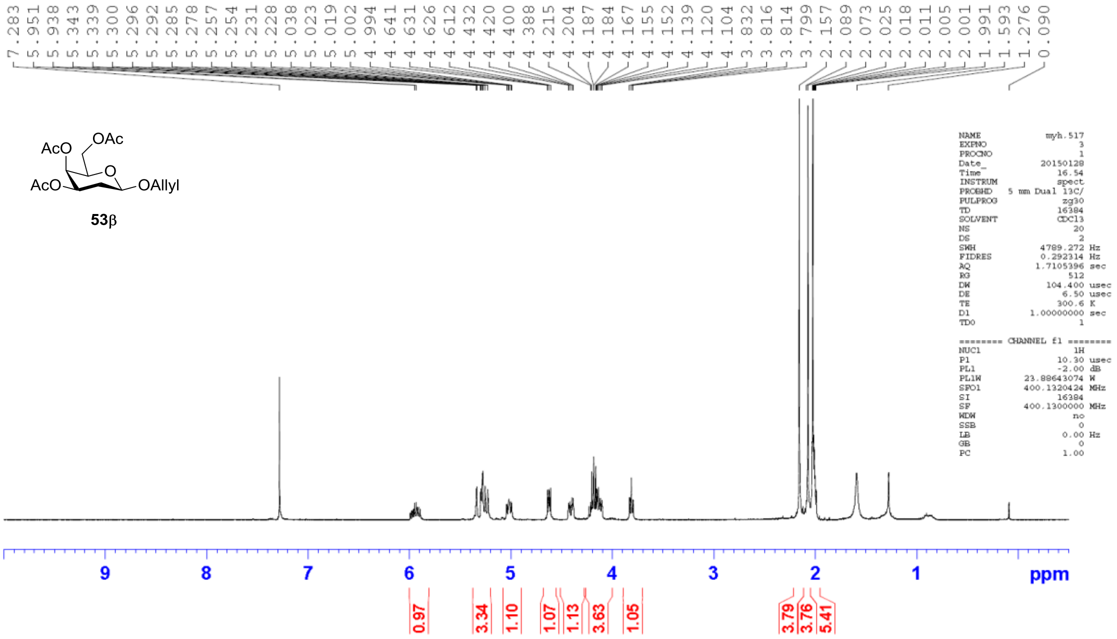
7.283
5.951
5.938
5.343
5.339
5.300
5.296
5.292
5.285
5.278
5.257
5.254
5.231
5.228
5.038
5.023
5.019
5.002
4.994
4.641
4.631
4.626
4.612
4.432
4.420
4.400
4.388
4.215
4.204
4.187
4.184
4.167
4.155
4.152
4.139
4.120
4.104
3.832
3.816
3.814
3.799
2.157
2.089
2.073
2.025
2.018
2.011
2.005
2.001
1.991
1.593
1.276
0.090
AcO
OAc
O
AcO
OAllyl
53β
9 8 7 6 5 4 3 2 1 ppm
0.97
3.34
1.10
1.07
1.13
3.63
1.05
3.79
3.76
5.41

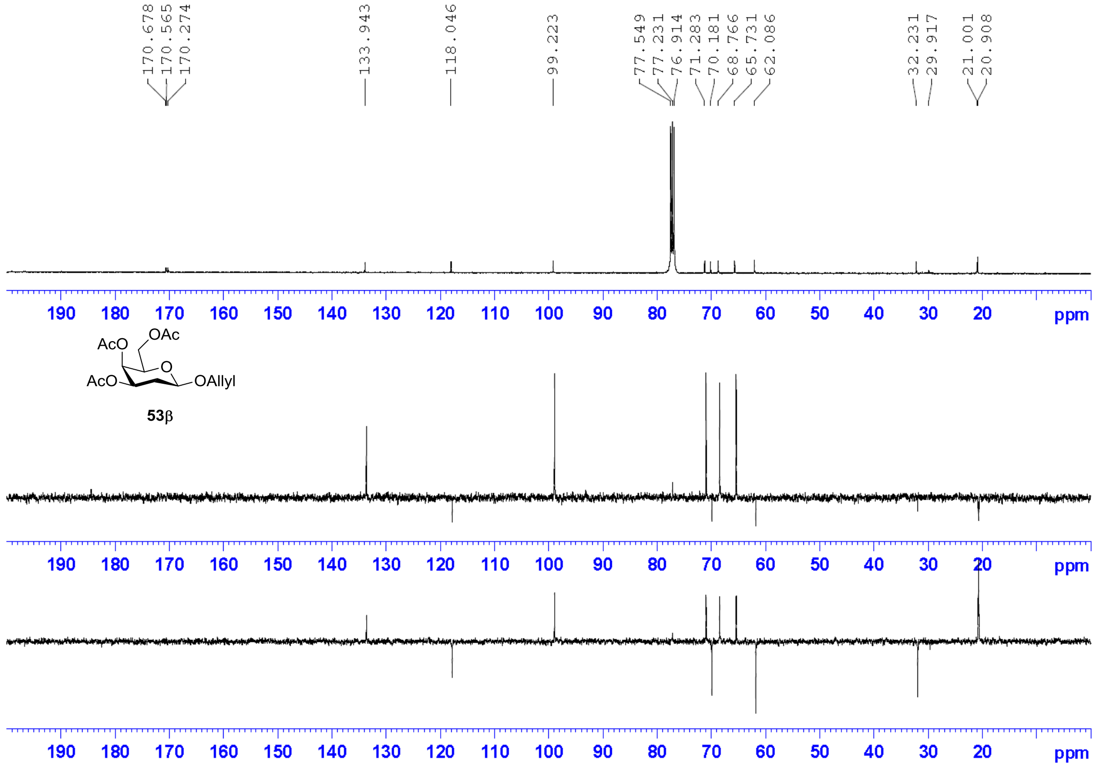
170.678
170.565
170.274
133.943
118.046
99.223
77.549
77.231
76.914
71.283
70.181
68.766
65.731
62.086
32.231
29.917
21.001
20.908
190 180 170 160 150 140 130 120 110 100 90 80 70 60 50 40 30 20 ppm
AcO
OAc
AcO
O
OAllyl
53β

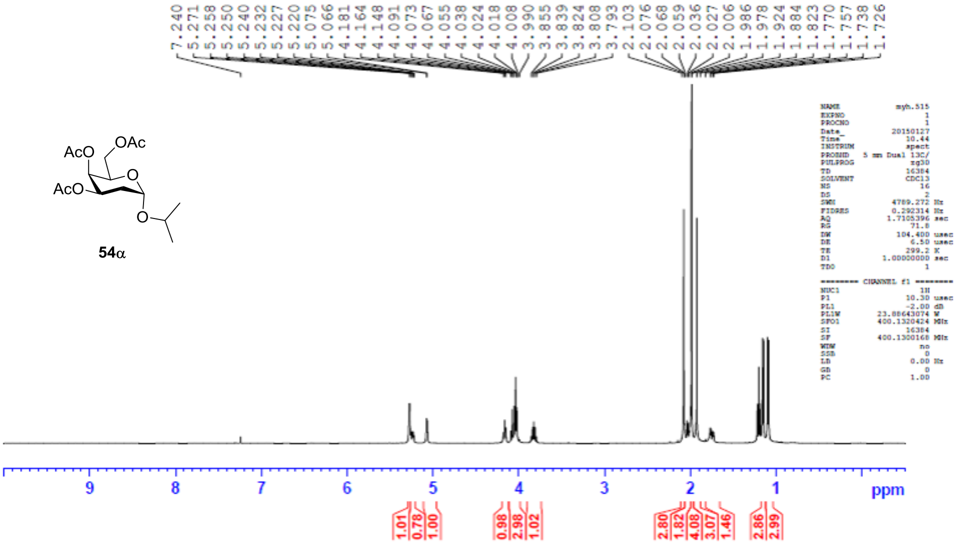
7.240
5.271
5.258
5.250
5.240
5.232
5.227
5.220
5.075
5.066
4.181
4.164
4.148
4.091
4.073
4.067
4.055
4.038
4.024
4.018
4.008
3.990
3.855
3.839
3.824
3.808
3.793
2.103
2.076
2.068
2.059
2.036
2.027
2.006
1.986
1.978
1.924
1.884
1.823
1.770
1.757
1.738
1.726
AcO
OAc
AcO
O
54α
9 8 7 6 5 4 3 2 1 ppm
1.01
0.78
1.00
0.98
2.98
1.02
2.80
1.82
4.08
3.07
1.46
2.86
2.99

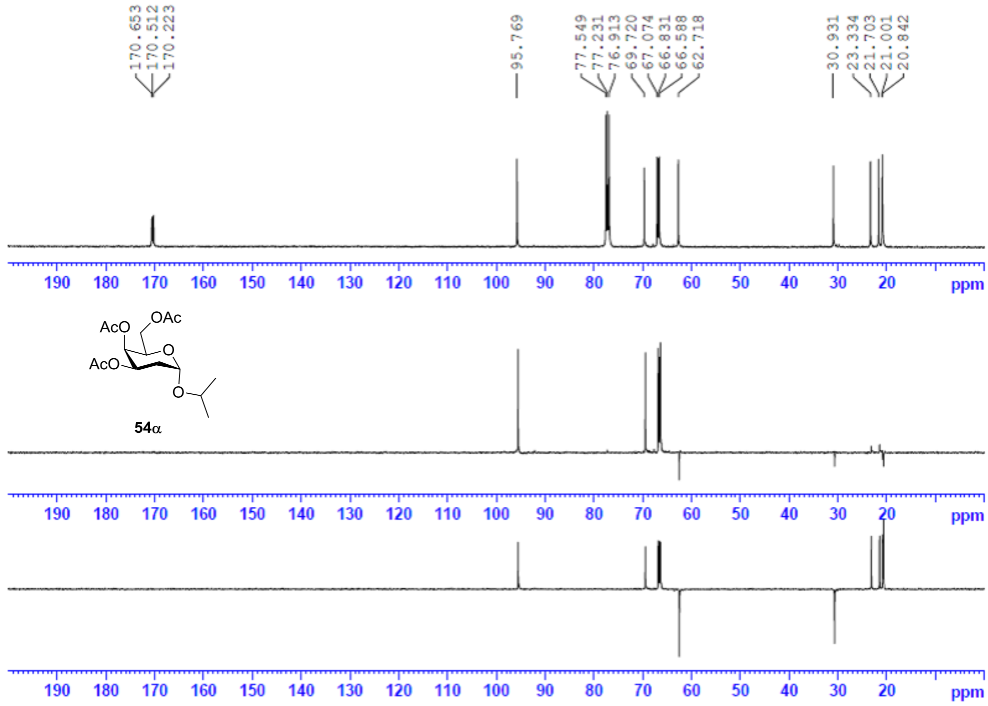
170.653
170.512
170.223
95.769
77.549
77.231
76.913
69.720
67.074
66.831
66.588
62.718
30.931
23.334
21.703
21.001
20.842
190 180 170 160 150 140 130 120 110 100 90 80 70 60 50 40 30 20 ppm
AcO
OAc
AcO
O
O

54α

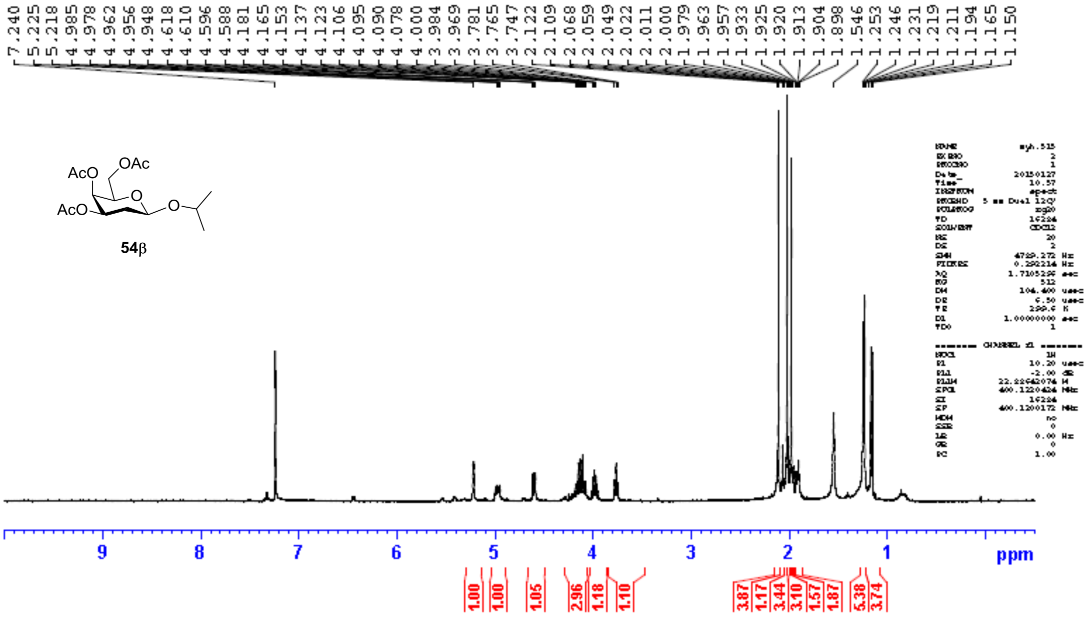
7.240
5.225
5.218
4.985
4.978
4.962
4.956
4.948
4.618
4.610
4.596
4.588
4.181
4.165
4.153
4.137
4.123
4.106
4.095
4.090
4.078
4.000
3.984
3.969
3.781
3.765
3.747
2.122
2.109
2.068
2.059
2.049
2.022
2.011
2.000
1.979
1.963
1.957
1.933
1.925
1.920
1.913
1.904
1.898
1.546
1.253
1.246
1.231
1.219
1.211
1.194
1.165
1.150
AcO
OAc
AcO
O
O
9 8 7 6 5 4 3 2 1 ppm
1.00
1.00
1.05
2.96
1.18
1.10
3.87
1.17
3.44
3.10
1.57
1.87
5.38
3.74

54β

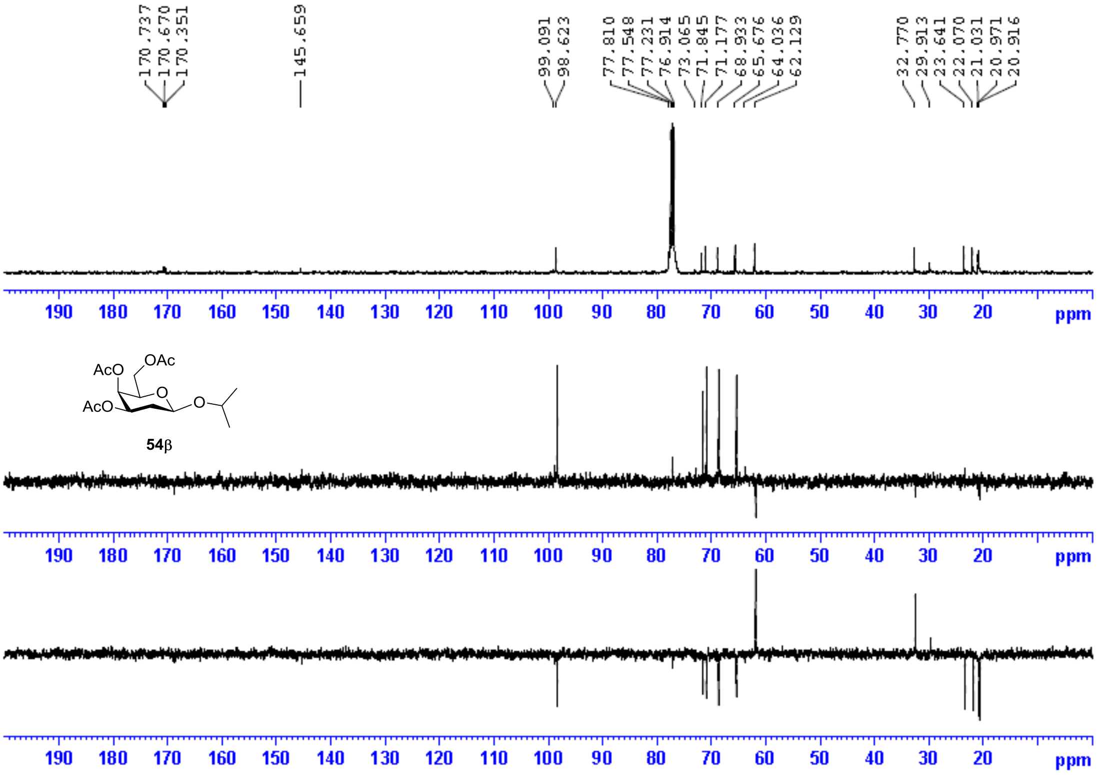
170.737
170.670
170.351
145.659
99.091
98.623
77.810
77.548
77.231
76.914
73.065
71.845
71.177
68.933
65.676
64.036
62.129
32.770
29.913
23.641
22.070
21.031
20.971
20.916
ppm
AcO
OAc
O
AcO
O
54β

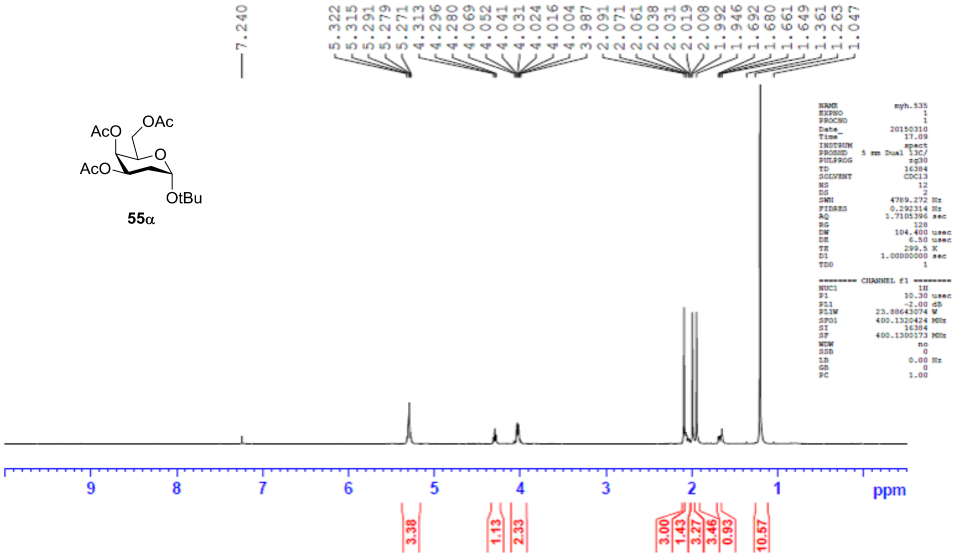
7.240
5.322
5.315
5.291
5.279
5.271
4.313
4.296
4.280
4.069
4.052
4.041
4.031
4.024
4.016
4.004
3.987
2.091
2.071
2.061
2.038
2.031
2.019
2.008
1.992
1.946
1.692
1.680
1.661
1.649
1.361
1.263
1.047
AcO
OAc
O
AcO
OtBu
55α
ppm
3.38
1.13
2.33
3.00
1.43
3.27
3.46
0.93
10.57

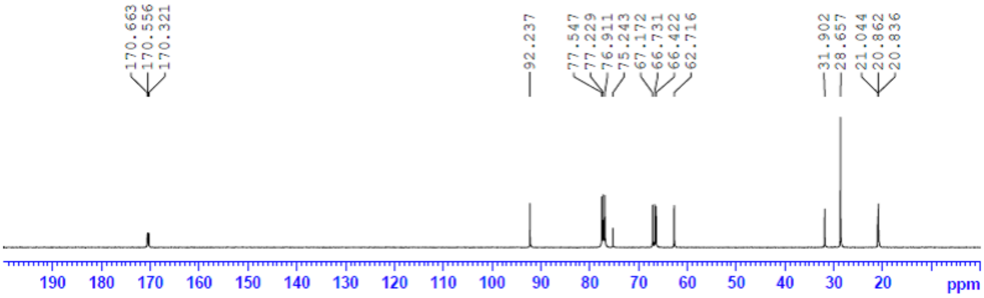
170.663
170.556
170.321
92.237
77.547
77.229
76.911
75.243
67.172
66.731
66.422
62.716
31.902
28.657
21.044
20.862
20.836
190 180 170 160 150 140 130 120 110 100 90 80 70 60 50 40 30 20 ppm

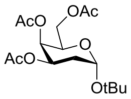
AcO
OAc
AcO
O
OtBu

55α

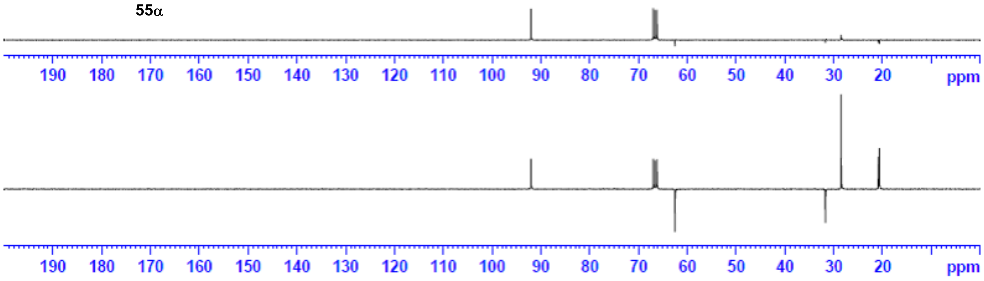
190 180 170 160 150 140 130 120 110 100 90 80 70 60 50 40 30 20 ppm
190 180 170 160 150 140 130 120 110 100 90 80 70 60 50 40 30 20 ppm

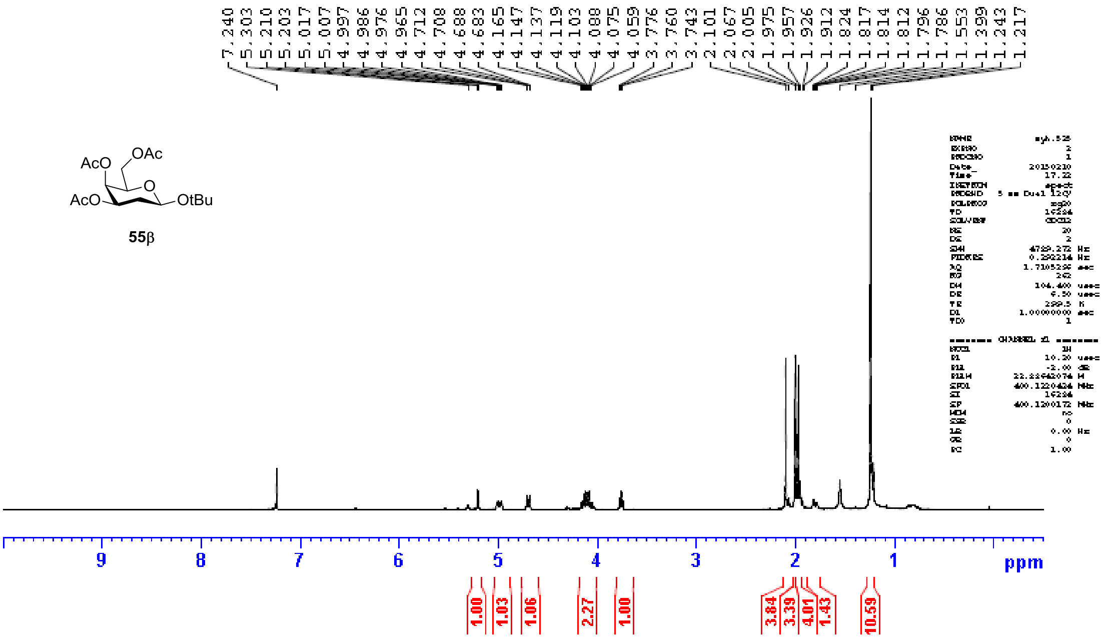
AcO
OAc
AcO
O
OtBu
7.240
5.303
5.210
5.203
5.017
5.007
4.997
4.986
4.976
4.965
4.712
4.708
4.688
4.683
4.165
4.147
4.137
4.119
4.103
4.088
4.075
4.059
3.776
3.760
3.743
2.101
2.067
2.005
1.975
1.957
1.926
1.912
1.824
1.817
1.814
1.812
1.796
1.786
1.553
1.399
1.243
1.217
9 8 7 6 5 4 3 2 1 ppm
1.00
1.03
1.06
2.27
1.00
3.84
3.39
4.01
1.43
10.59

55β

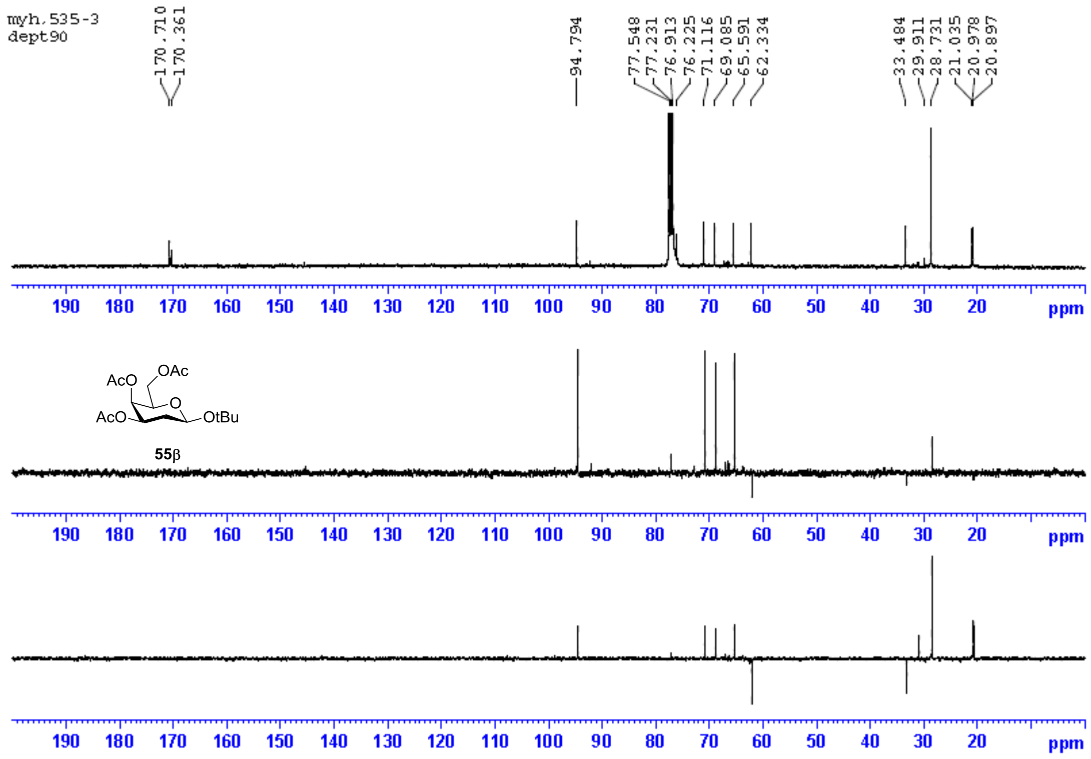
myh.535-3
dept90
170.710
170.361
94.794
77.548
77.231
76.913
76.225
71.116
69.085
65.591
62.334
33.484
29.911
28.731
21.035
20.978
20.897
AcO
OAc
O
AcO
OtBu
55β
190 180 170 160 150 140 130 120 110 100 90 80 70 60 50 40 30 20 ppm

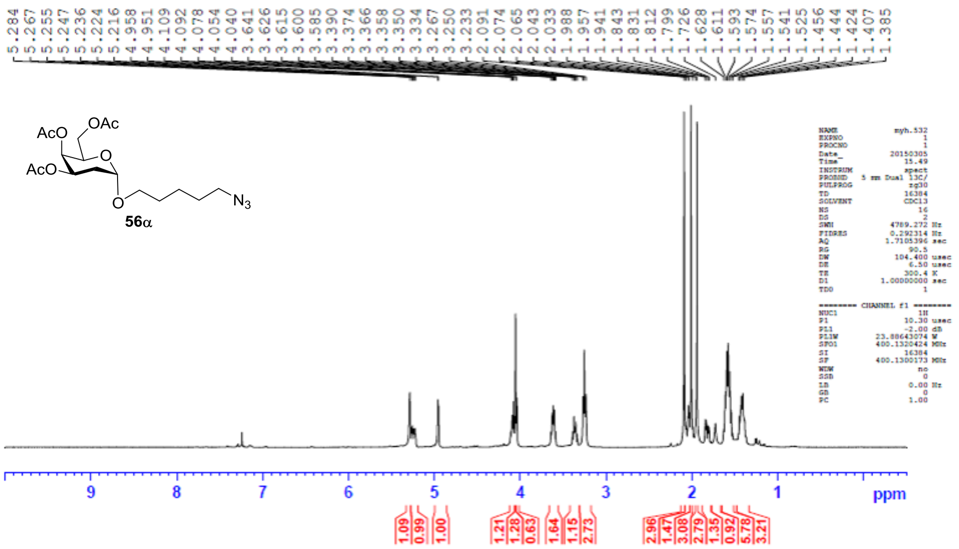
5.284
5.267
5.255
5.247
5.236
5.224
5.216
4.958
4.951
4.109
4.092
4.078
4.054
4.040
3.641
3.626
3.615
3.600
3.585
3.390
3.374
3.366
3.358
3.350
3.334
3.267
3.250
3.233
2.091
2.074
2.065
2.043
2.033
1.988
1.957
1.941
1.843
1.831
1.812
1.799
1.726
1.628
1.611
1.593
1.574
1.557
1.541
1.525
1.456
1.444
1.424
1.407
1.385
AcO
OAc
O
AcO
O
N3
56α
9 8 7 6 5 4 3 2 1 ppm
1.09
0.99
1.00
1.21
1.28
0.63
1.64
1.15
2.73
2.96
1.47
3.08
2.79
1.35
0.92
5.78
3.21

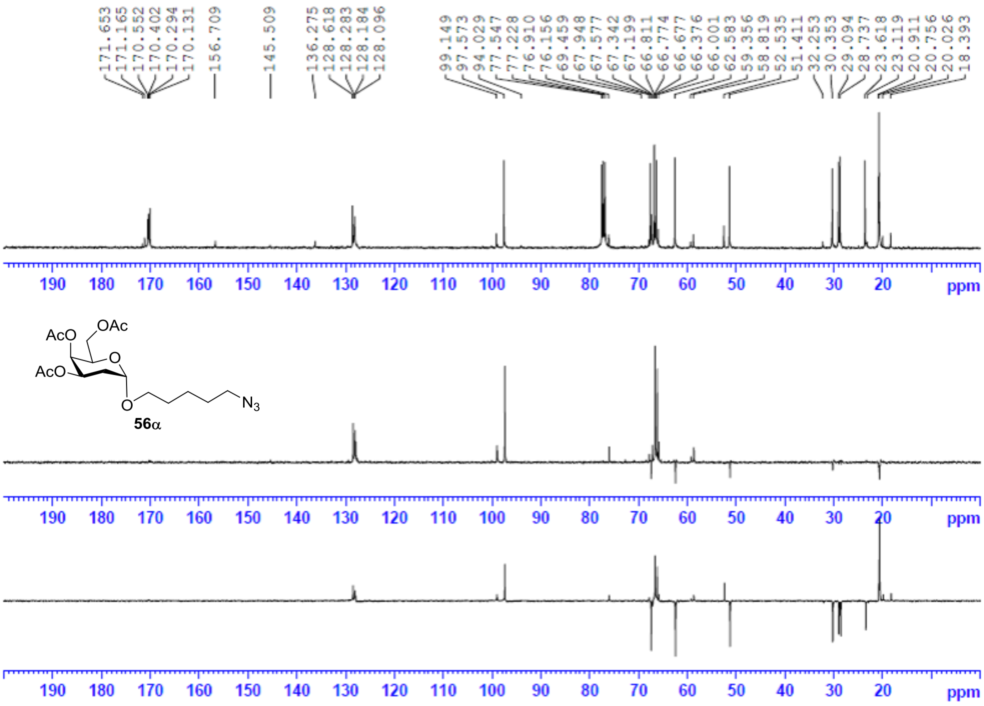

56α

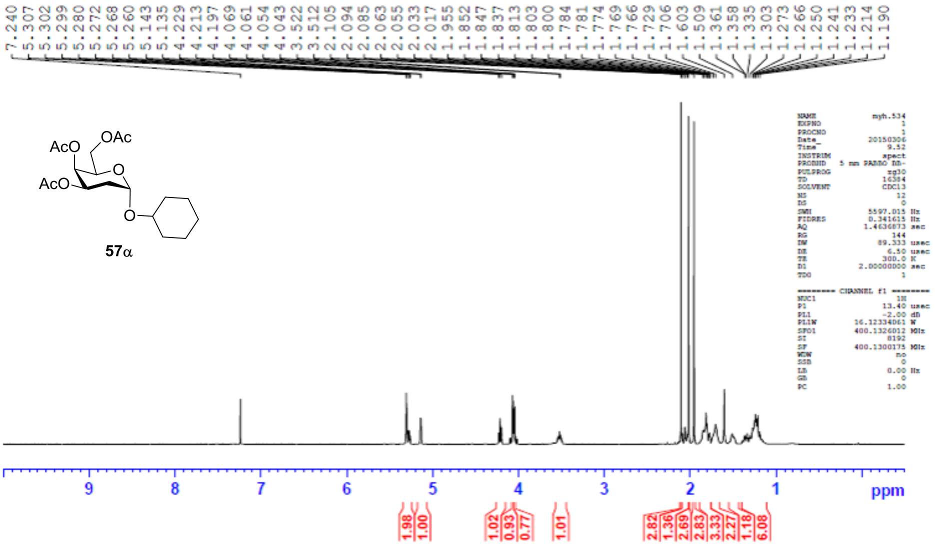

57α

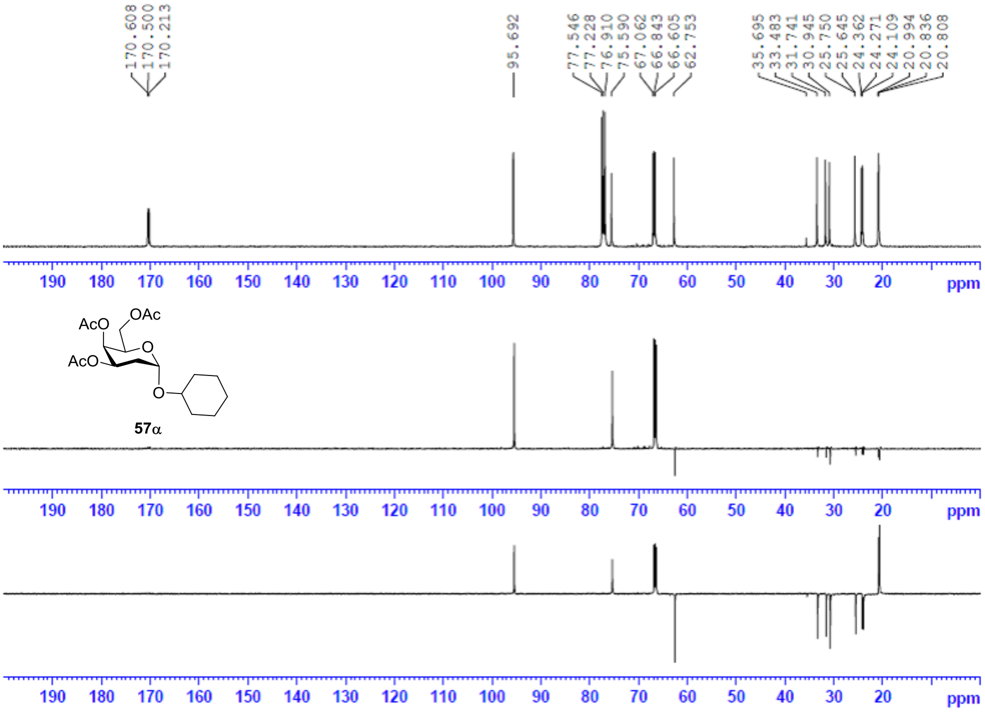
AcO
OAc
AcO
O
O
57α
ppm

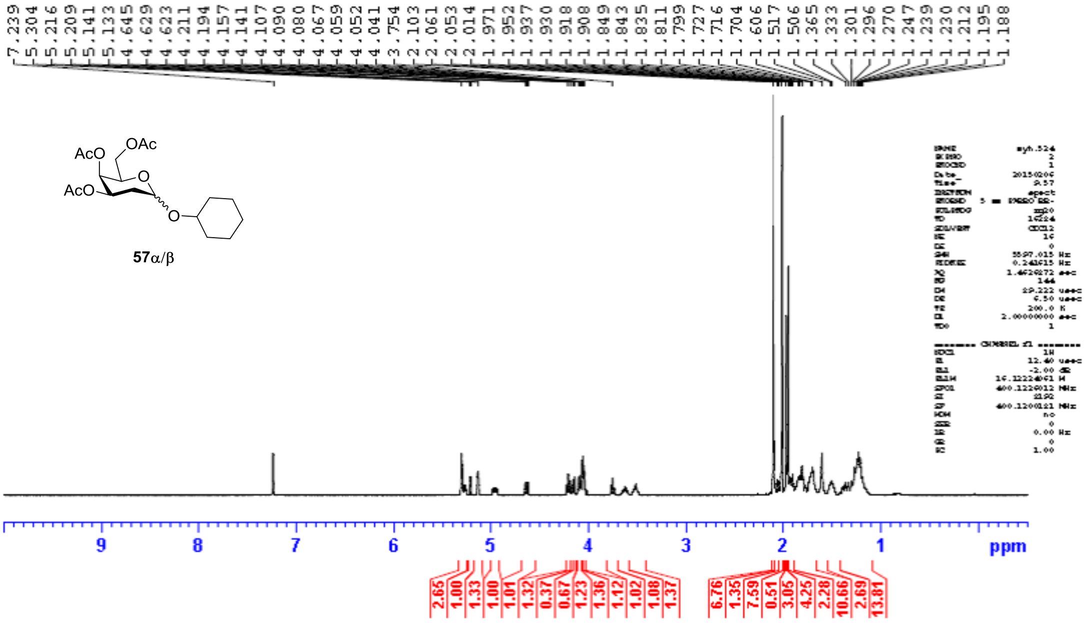
AcO
OAc
AcO
O
O
57α/β
ppm

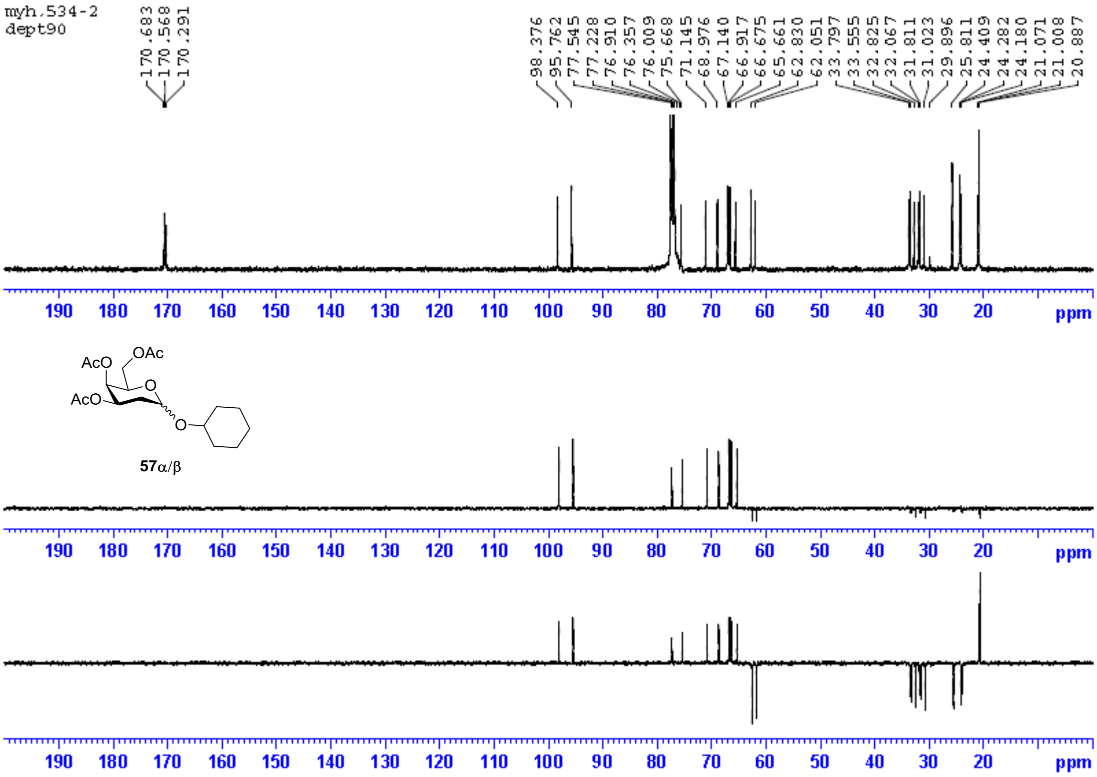
myh.534-2
dept90
170.683 170.568 170.291
98.376 95.762 77.545 77.228 76.910 76.357 76.009 75.668 71.145 68.976 67.140 66.917 66.675 65.661 62.830 62.051 33.797 33.555 32.825 32.067 31.811 31.023 29.896 25.811 24.409 24.282 24.180 21.071 21.008 20.887
AcO
OAc
AcO
O
O
57α/β
ppm

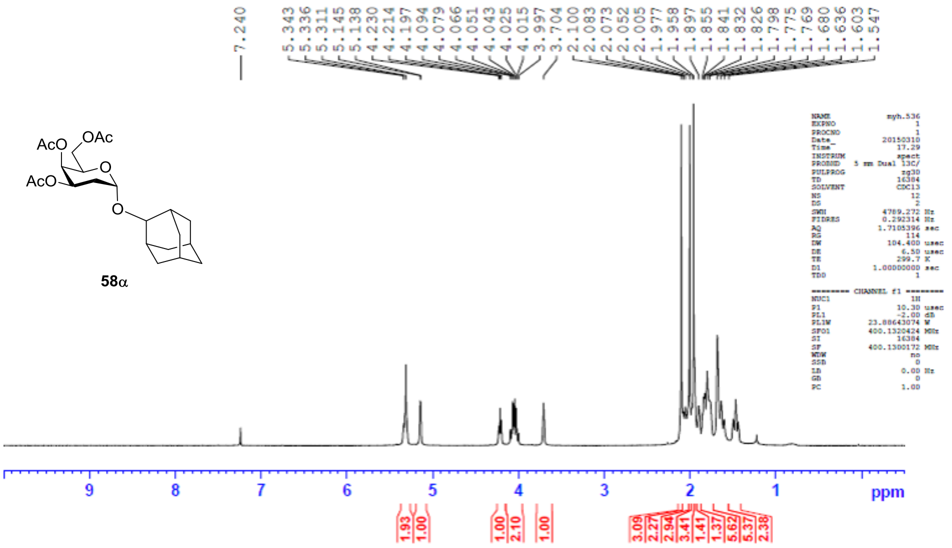
7.240
5.343 5.336 5.311 5.145 5.138 4.230 4.214 4.197 4.094 4.079 4.066 4.051 4.043 4.025 4.015 3.997 3.704 2.100 2.083 2.073 2.052 2.005 1.977 1.958 1.897 1.855 1.841 1.832 1.826 1.798 1.775 1.769 1.680 1.636 1.603 1.547
AcO
OAc
AcO
O
O
58α
ppm
1.93 1.00 1.00 2.10 1.00 3.09 2.27 2.94 3.41 1.41 1.37 5.62 5.37 2.38

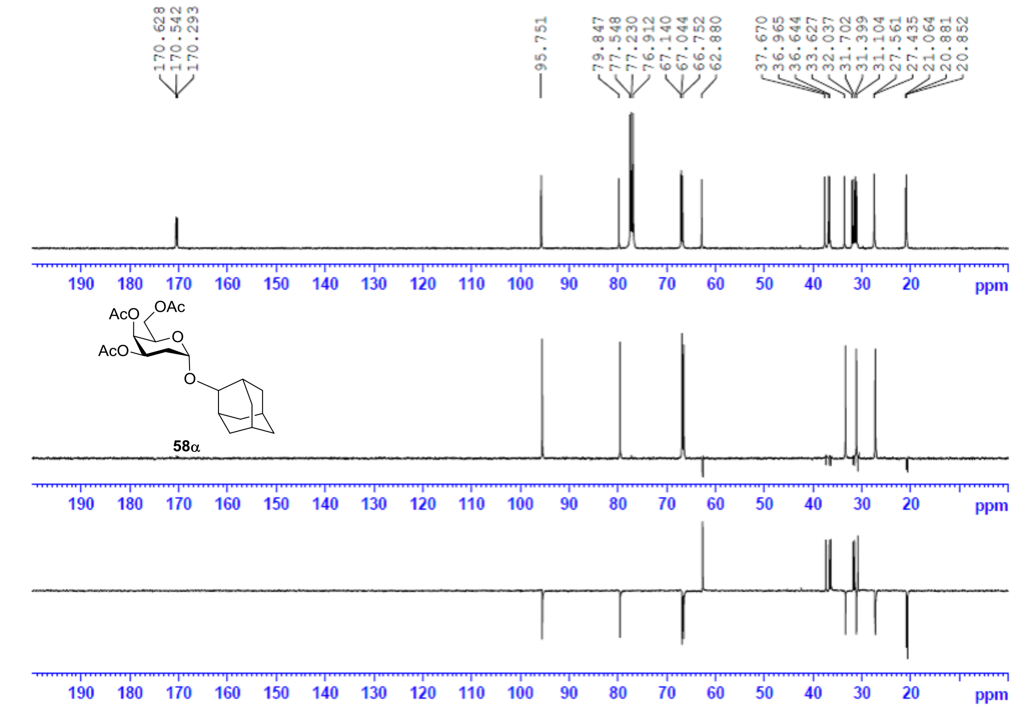
170.628
170.542
170.293
95.751
79.847
77.548
77.230
76.912
67.140
67.044
66.752
62.880
37.670
36.965
36.644
33.627
32.037
31.702
31.399
31.104
27.561
27.435
21.064
20.881
20.852
AcO
OAc
AcO
O
O
58α
ppm

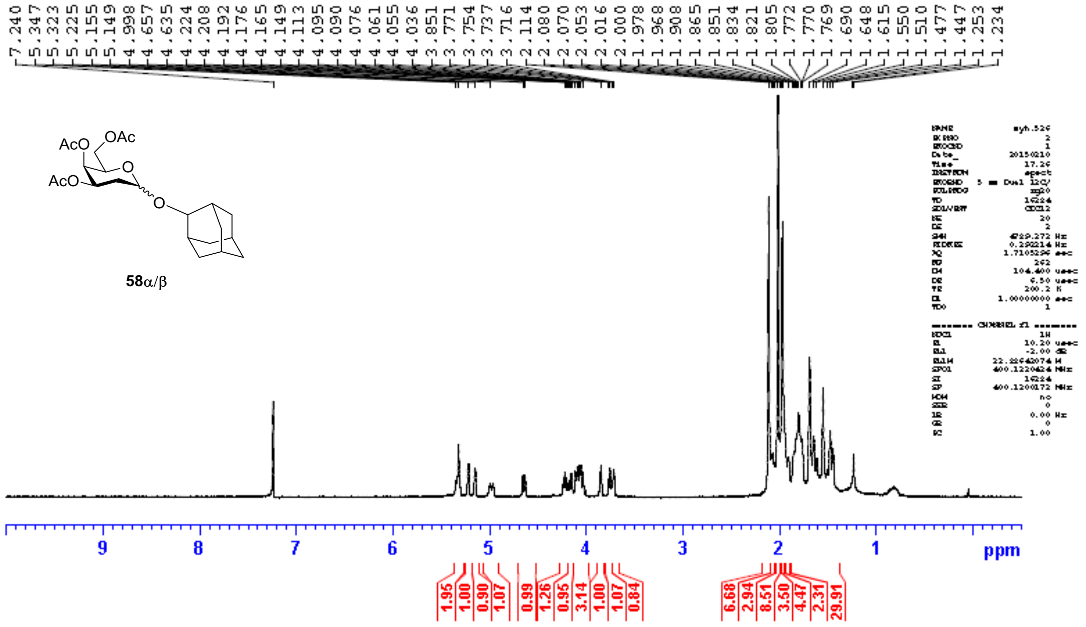
7.240
5.347
5.323
5.225
5.155
5.149
4.998
4.657
4.635
4.224
4.208
4.192
4.176
4.165
4.149
4.113
4.095
4.090
4.076
4.061
4.055
4.036
3.851
3.771
3.754
3.737
3.716
2.114
2.080
2.070
2.053
2.016
2.000
1.978
1.968
1.908
1.865
1.851
1.834
1.821
1.805
1.772
1.770
1.769
1.690
1.648
1.615
1.550
1.510
1.477
1.447
1.253
1.234
AcO
OAc
AcO
O
O
58α/β
ppm
1.95
1.00
0.90
1.07
0.99
1.26
0.95
3.14
1.00
1.07
0.84
6.68
2.94
8.51
3.50
4.47
2.31
29.91

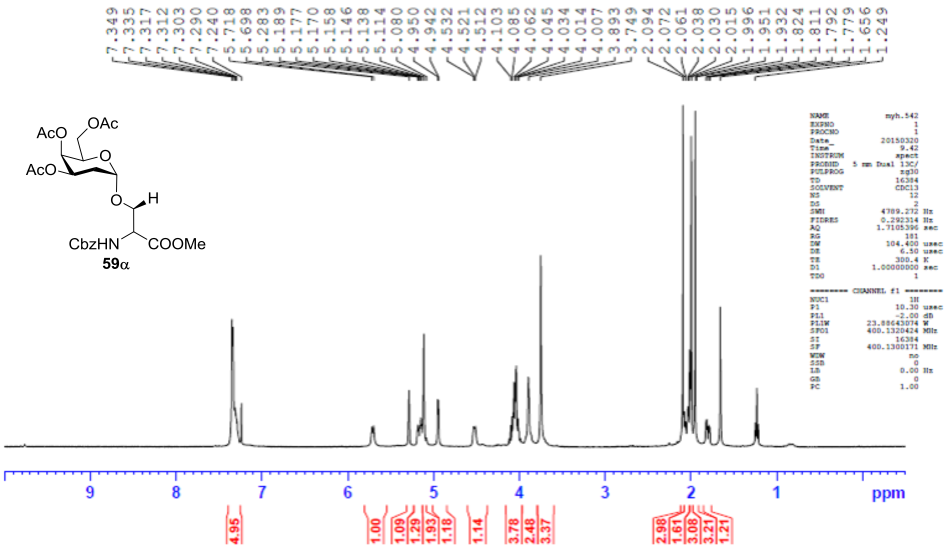
AcO
OAc
AcO
O
O
H
CbzHN
COOMe
59α
ppm

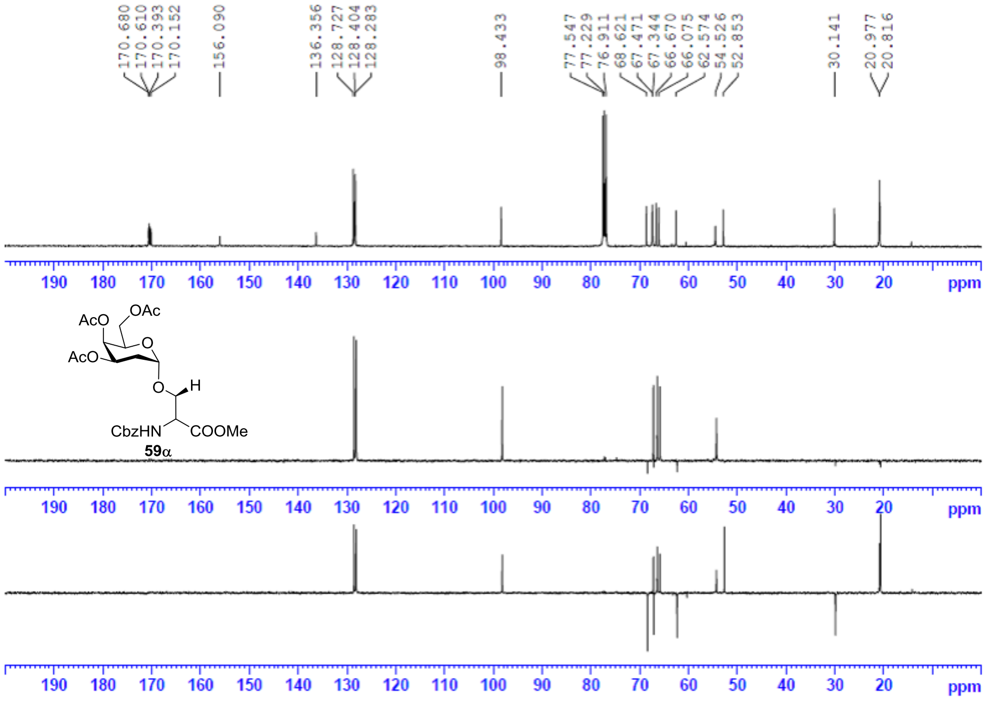
AcO
OAc
AcO
O
O
H
CbzHN
COOMe
59α
ppm

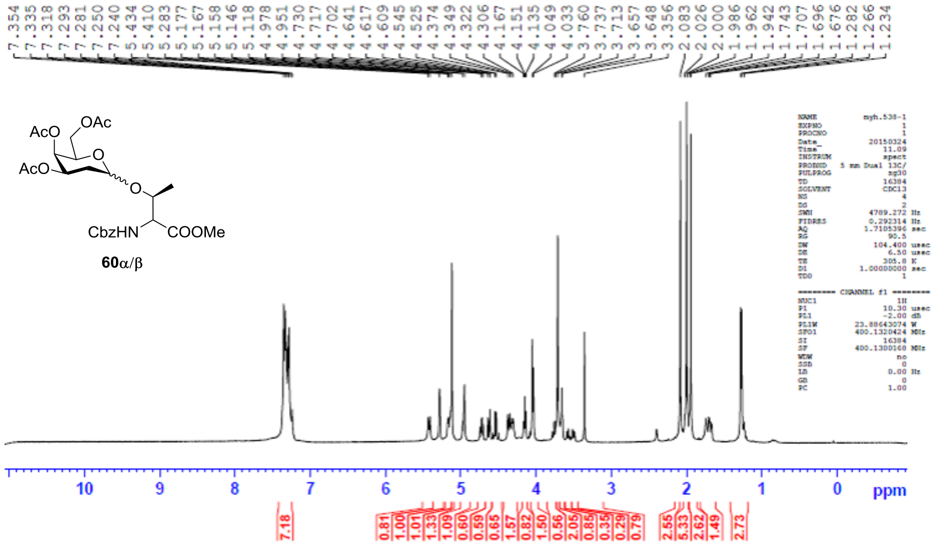
AcO
OAc
AcO
O
O
CbzHN
COOMe
60α/β
7.354 7.335 7.318 7.293 7.281 7.250 7.240 5.434 5.410 5.283 5.177 5.167 5.158 5.146 5.118 4.978 4.951 4.730 4.717 4.702 4.641 4.617 4.609 4.545 4.525 4.374 4.349 4.322 4.306 4.167 4.151 4.135 4.049 4.033 3.760 3.737 3.713 3.657 3.648 3.356 2.083 2.026 2.000 1.986 1.962 1.942 1.743 1.707 1.696 1.676 1.282 1.266 1.234
10 9 8 7 6 5 4 3 2 1 0 ppm

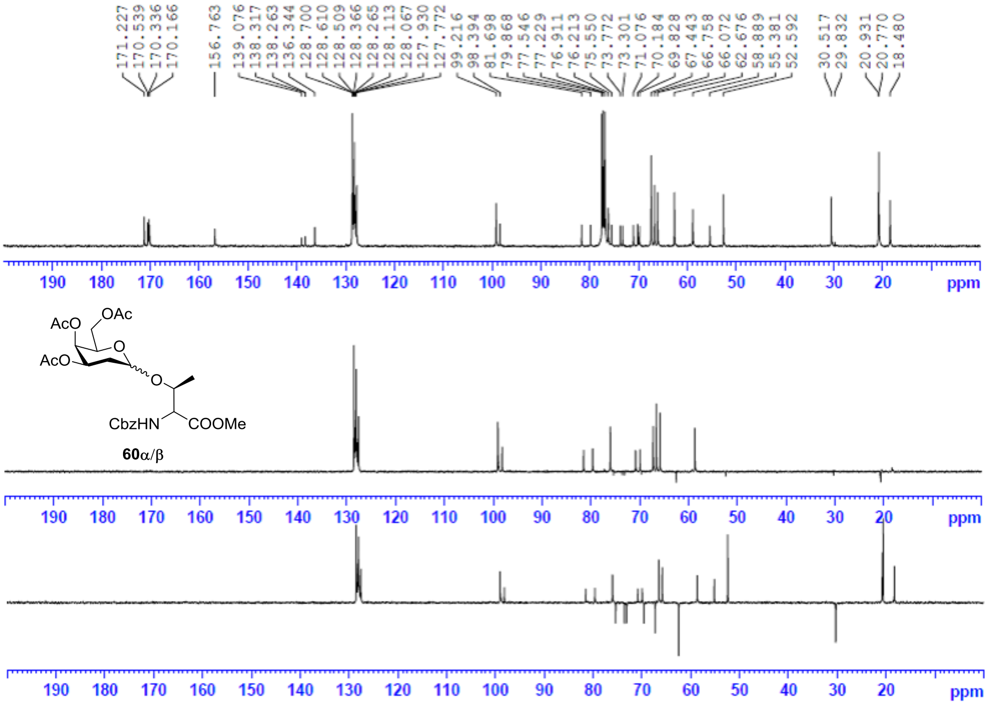
171.227 170.539 170.336 170.166 156.763 139.076 138.317 138.263 136.344 128.700 128.610 128.509 128.366 128.265 128.113 128.067 127.930 127.772 99.216 98.394 81.698 79.868 77.546 77.229 76.911 76.213 75.550 73.772 73.301 71.076 70.184 69.828 67.443 66.758 66.072 62.676 58.889 55.381 52.592 30.517 29.832 20.931 20.770 18.480
190 180 170 160 150 140 130 120 110 100 90 80 70 60 50 40 30 20 ppm
AcO
OAc
AcO
O
O
CbzHN
COOMe
60α/β

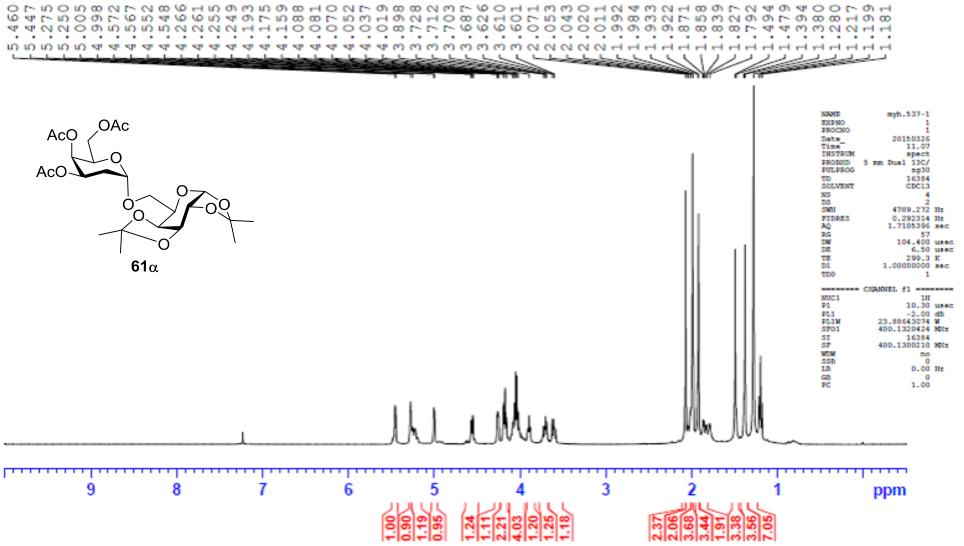
5.460 5.447 5.275 5.250 5.005 4.998 4.572 4.567 4.552 4.548 4.266 4.261 4.255 4.249 4.193 4.175 4.159 4.088 4.081 4.070 4.052 4.037 4.019 3.898 3.728 3.712 3.703 3.687 3.626 3.610 3.601 2.071 2.053 2.043 2.020 2.011 1.992 1.984 1.933 1.922 1.871 1.858 1.839 1.827 1.792 1.494 1.479 1.394 1.380 1.280 1.217 1.199 1.181
AcO
OAc
AcO
O
O
O
O
O
O
61α
9 8 7 6 5 4 3 2 1 ppm
1.00 0.90 1.19 0.95 1.24 1.11 2.21 4.03 1.20 1.25 1.18 2.37 2.06 3.68 3.44 1.91 3.38 3.56 7.05

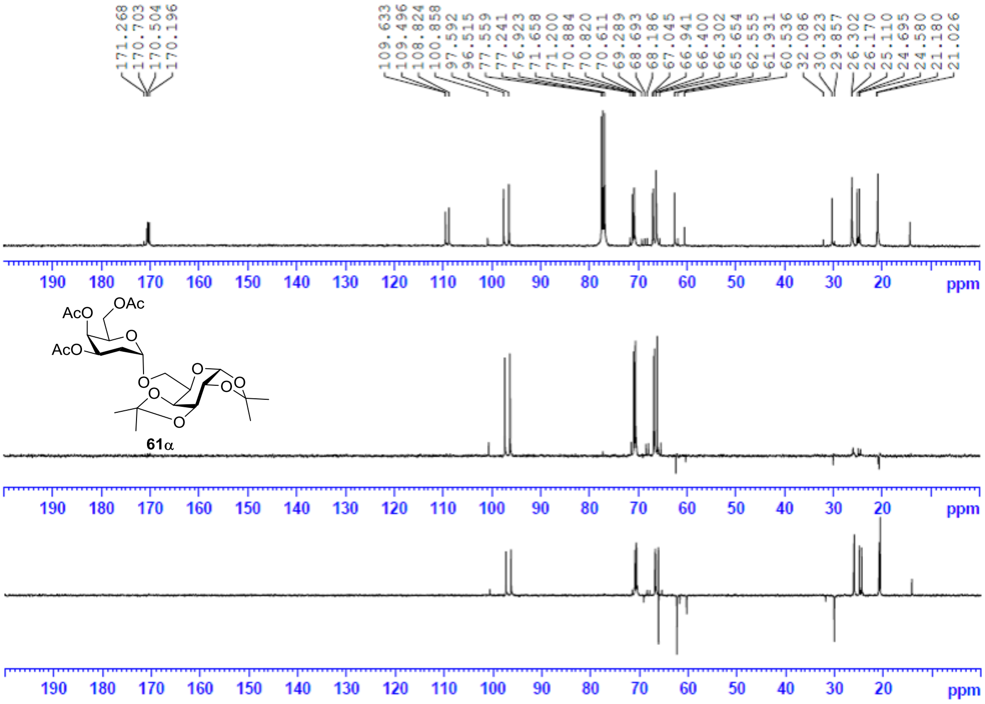
171.268 170.703 170.504 170.196
109.633 109.496 108.824 100.858 97.592 96.515 77.559 77.241 76.923 71.658 71.200 70.884 70.820 70.611 69.289 68.693 68.186 67.045 66.941 66.400 66.302 65.654 62.555 61.931 60.536 32.086 30.323 29.857 26.302 26.170 25.110 24.695 24.580 21.180 21.026
190 180 170 160 150 140 130 120 110 100 90 80 70 60 50 40 30 20 ppm
AcO
OAc
AcO
O
O
O
O
O
O
61α
190 180 170 160 150 140 130 120 110 100 90 80 70 60 50 40 30 20 ppm
190 180 170 160 150 140 130 120 110 100 90 80 70 60 50 40 30 20 ppm

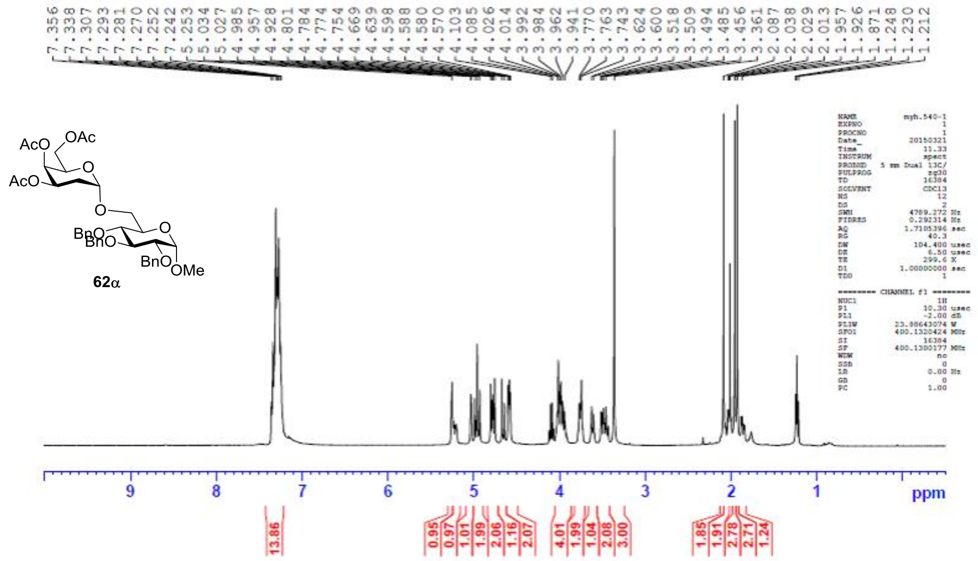
7.356
7.338
7.307
7.293
7.281
7.270
7.252
7.242
5.253
5.034
5.027
4.985
4.957
4.928
4.801
4.784
4.774
4.754
4.669
4.639
4.598
4.588
4.580
4.570
4.103
4.085
4.026
4.014
3.992
3.984
3.962
3.941
3.770
3.763
3.743
3.624
3.600
3.518
3.509
3.494
3.485
3.456
3.361
2.087
2.038
2.029
2.013
1.957
1.926
1.871
1.248
1.230
1.212
AcO
OAc
AcO
O
O
BnO
BnO
O
BnO
OMe
62α
9
8
7
6
5
4
3
2
1
ppm
13.86
0.95
0.97
1.01
1.99
2.06
1.16
2.07
4.01
1.99
1.04
2.08
3.00
1.85
1.91
2.78
2.71
1.24

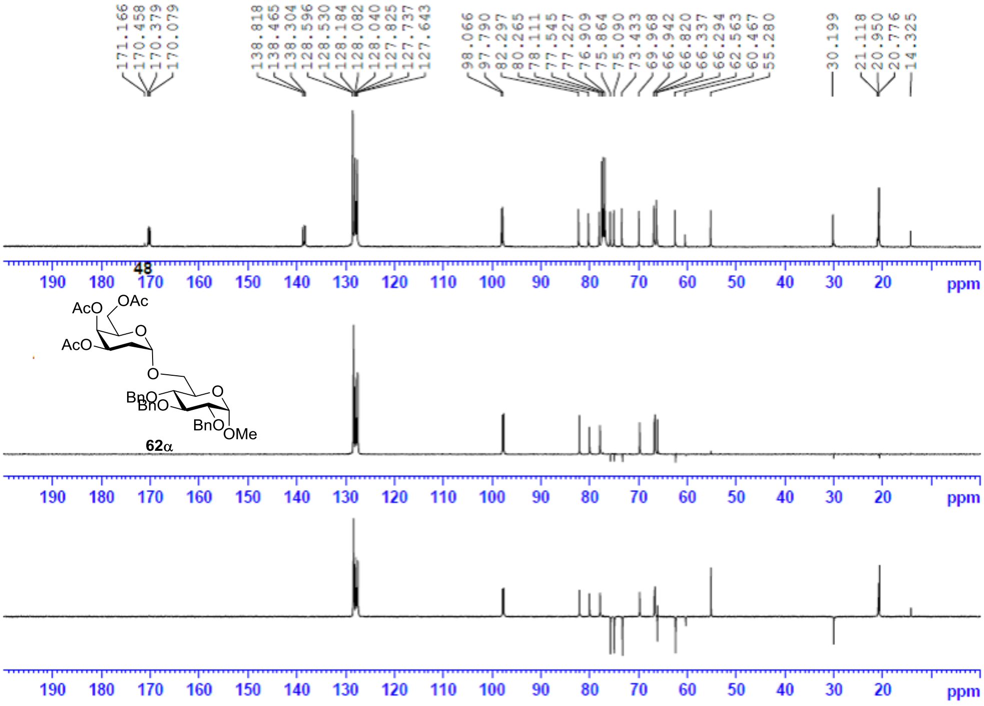
171.166
170.458
170.379
170.079
138.818
138.465
138.304
128.596
128.530
128.184
128.082
128.040
127.825
127.737
127.643
98.066
97.790
82.297
80.265
78.111
77.545
77.227
76.909
75.864
75.090
73.433
69.968
66.942
66.820
66.337
66.294
62.563
60.467
55.280
30.199
21.118
20.950
20.776
14.325
48
190
180
170
160
150
140
130
120
110
100
90
80
70
60
50
40
30
20
ppm
AcO
OAc
AcO
O
O
BnO
BnO
O
BnO
OMe
62α

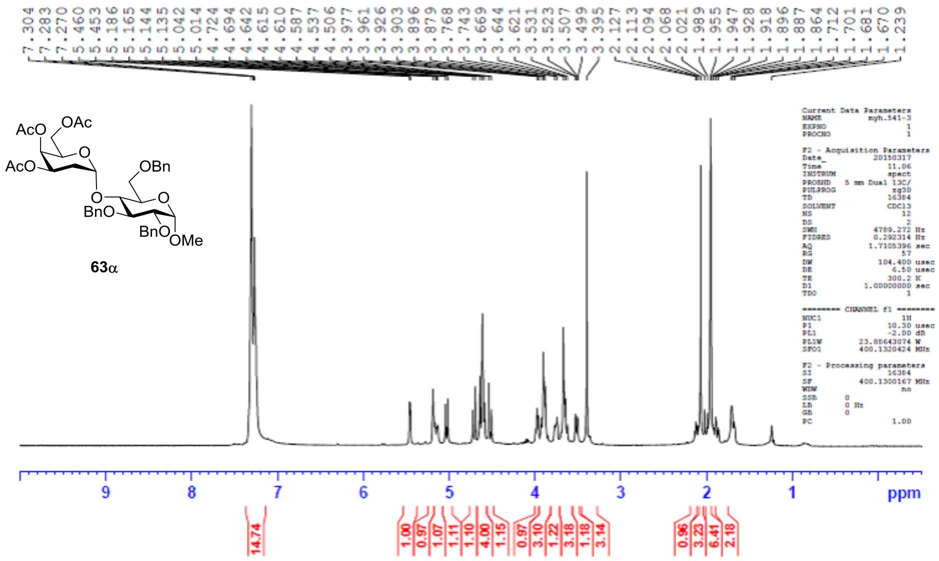
7.304
7.283
7.270
5.460
5.453
5.186
5.165
5.144
5.135
5.042
5.014
4.724
4.694
4.642
4.615
4.610
4.587
4.537
4.506
3.977
3.961
3.926
3.903
3.896
3.879
3.768
3.743
3.669
3.644
3.621
3.531
3.523
3.507
3.499
3.395
2.127
2.113
2.094
2.068
2.021
1.989
1.955
1.947
1.928
1.918
1.896
1.887
1.864
1.712
1.701
1.681
1.670
1.239
AcO
OAc
AcO
OBn
BnO
BnO
OMe
63α
9 8 7 6 5 4 3 2 1 ppm
14.74
1.00
0.97
1.07
1.11
1.10
4.00
1.15
0.97
3.10
1.22
3.18
1.18
3.14
0.96
3.23
6.41
2.18

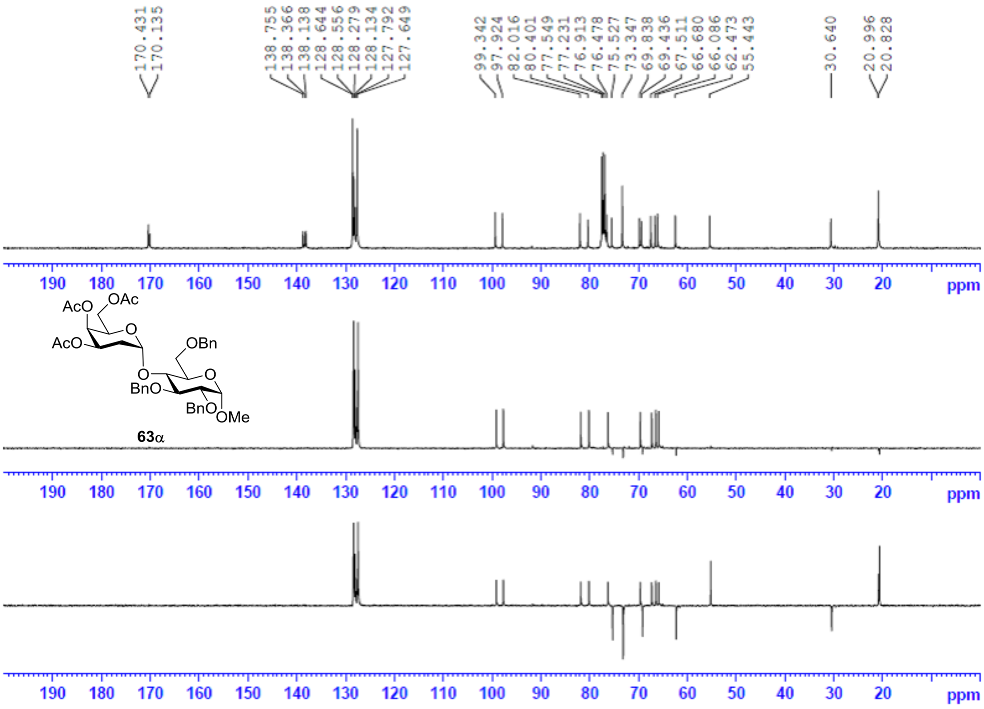
170.431
170.135
138.755
138.366
138.138
128.644
128.556
128.279
128.134
127.792
127.649
99.342
97.924
82.016
80.401
77.549
77.231
76.913
76.478
75.527
73.347
69.838
69.436
67.511
66.680
66.086
62.473
55.443
30.640
20.996
20.828
190 180 170 160 150 140 130 120 110 100 90 80 70 60 50 40 30 20 ppm
AcO
OAc
AcO
OBn
BnO
BnO
OMe
63α

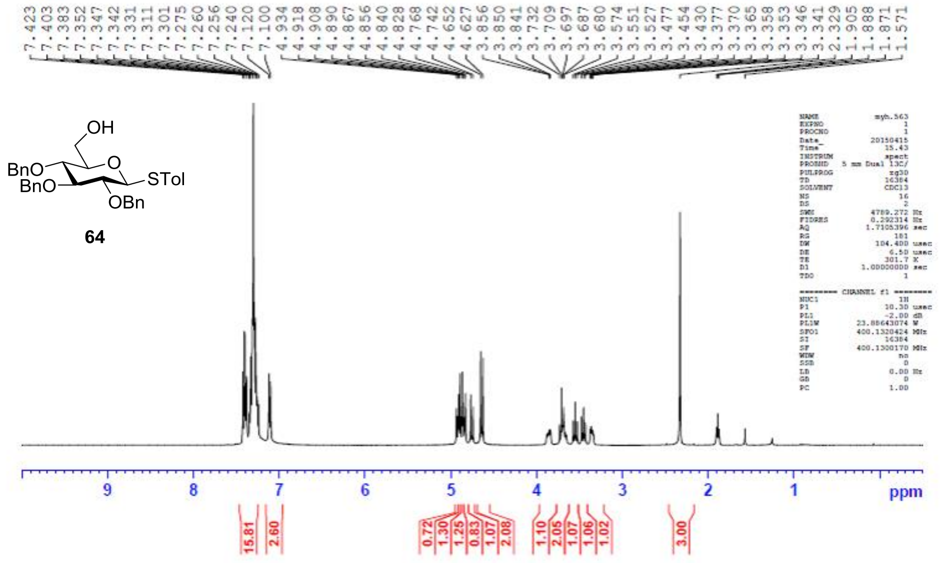
7.423
7.403
7.383
7.352
7.347
7.342
7.331
7.311
7.301
7.275
7.260
7.256
7.240
7.120
7.100
4.934
4.918
4.908
4.890
4.867
4.856
4.840
4.828
4.768
4.742
4.652
4.627
3.856
3.850
3.841
3.732
3.709
3.697
3.687
3.680
3.574
3.551
3.527
3.477
3.454
3.430
3.377
3.370
3.365
3.358
3.353
3.346
3.341
2.329
1.905
1.888
1.871
1.571
OH
BnO
BnO
O
STol
OBn
64
9
8
7
6
5
4
3
2
1
ppm
15.81
2.60
0.72
1.30
1.25
0.83
1.07
2.08
1.10
2.05
1.07
1.06
1.02
3.00

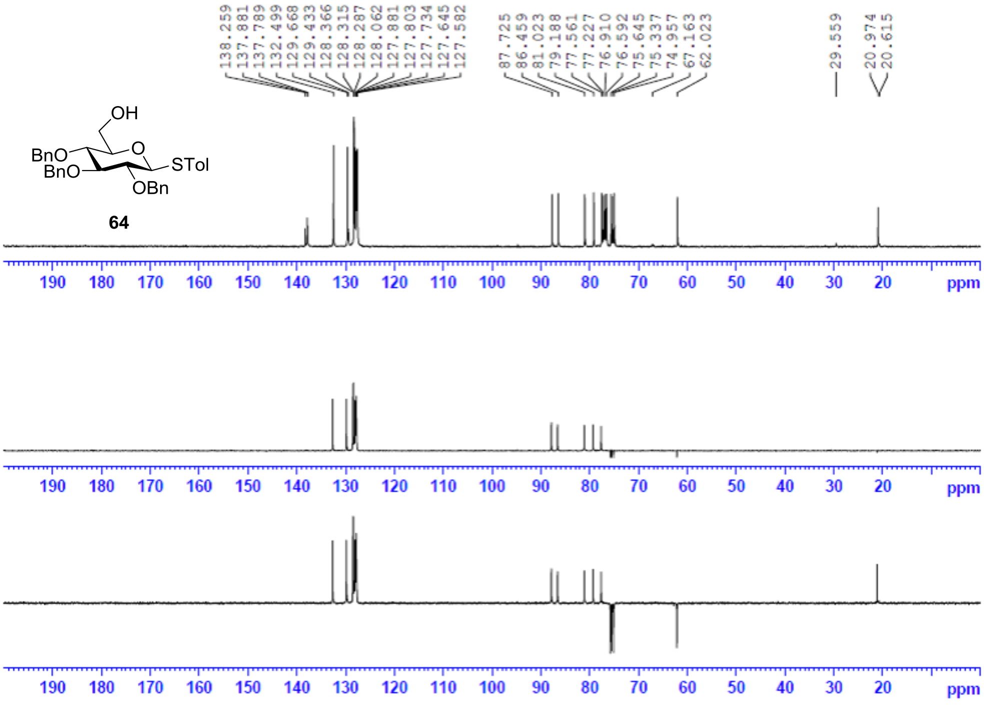
138.259
137.881
137.789
132.499
129.668
129.433
128.366
128.315
128.287
128.062
127.881
127.803
127.734
127.645
127.582
87.725
86.459
81.023
79.188
77.561
77.227
76.910
76.592
75.645
75.337
74.957
67.163
62.023
29.559
20.974
20.615
OH
BnO
BnO
O
STol
OBn
64
190
180
170
160
150
140
130
120
110
100
90
80
70
60
50
40
30
20
ppm

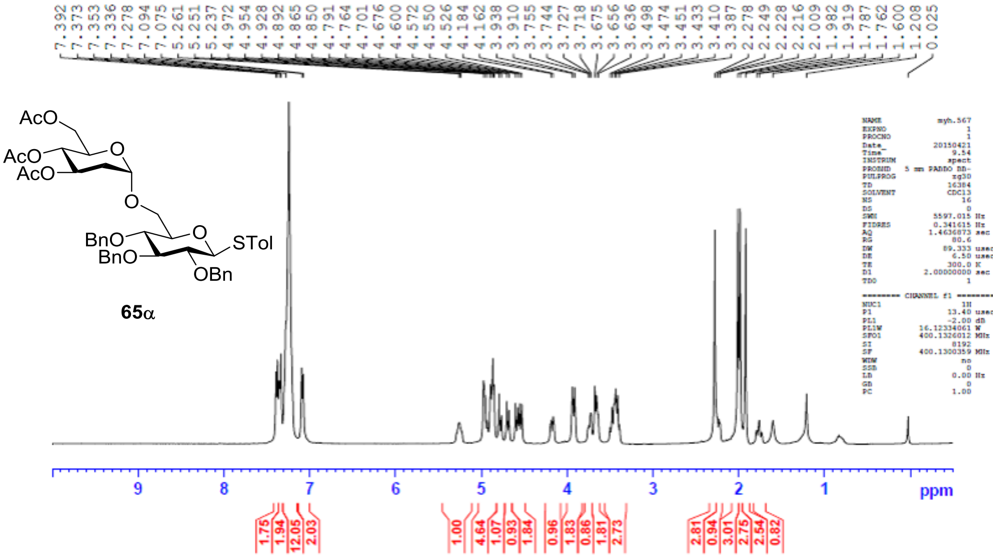
AcO
AcO
AcO
O
O
BnO
BnO
O
STol
OBn
7.392 7.373 7.353 7.336 7.278 7.094 7.075 5.261 5.251 5.237 4.972 4.954 4.928 4.892 4.865 4.850 4.791 4.764 4.701 4.676 4.600 4.572 4.550 4.526 4.184 4.162 3.938 3.910 3.755 3.744 3.727 3.718 3.675 3.656 3.636 3.498 3.474 3.451 3.433 3.410 3.387 2.278 2.249 2.228 2.216 2.009 1.982 1.919 1.787 1.762 1.600 1.208 0.025
9
8
7
6
5
4
3
2
1
ppm
1.75 1.94 12.05 2.03 1.00 4.64 1.07 0.93 1.84 0.96 1.83 0.86 1.81 2.73 2.81 0.94 3.01 2.75 2.54 0.82

65α

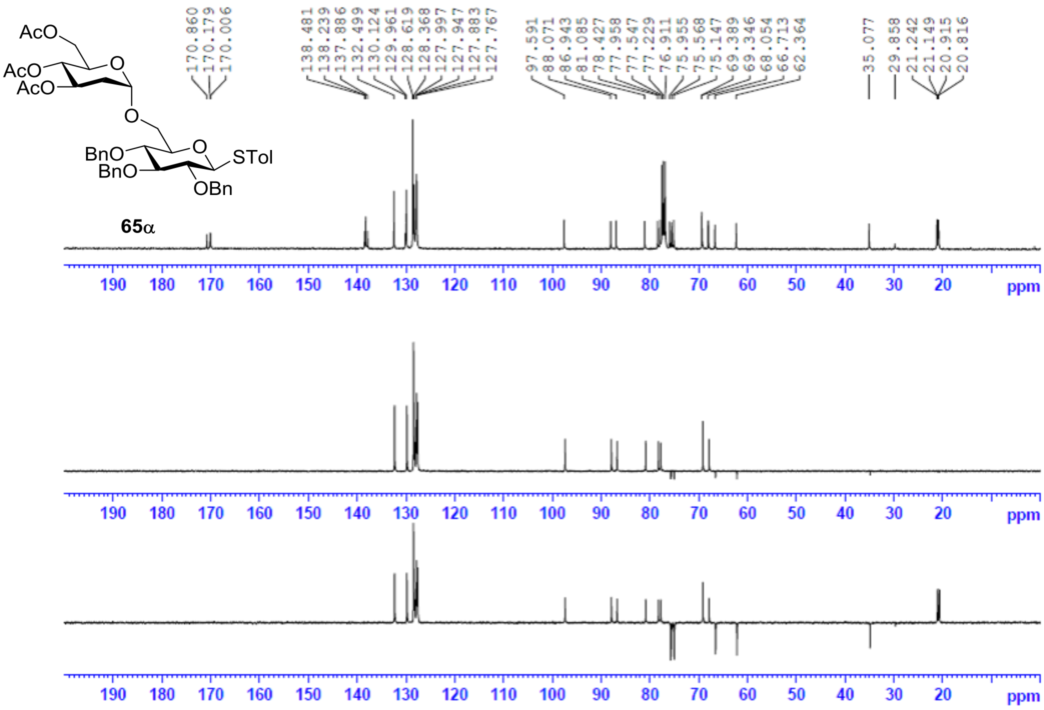
AcO
AcO
AcO
O
O
BnO
BnO
O
STol
OBn
170.860 170.179 170.006
138.481 138.239 137.886 132.499 130.124 129.961 128.619 128.368 127.997 127.947 127.883 127.767
97.591 88.071 86.943 81.085 78.427 77.958 77.547 77.229 76.911 75.955 75.568 75.147 69.389 69.346 68.054 66.713 62.364
35.077 29.858 21.242 21.149 20.915 20.816
190 180 170 160 150 140 130 120 110 100 90 80 70 60 50 40 30 20 ppm

65α

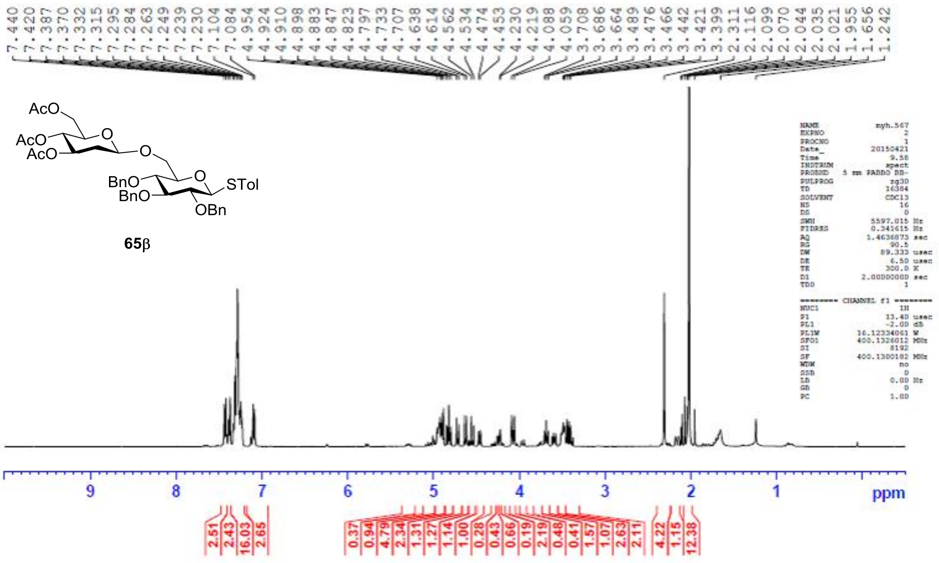
AcO
AcO
AcO
BnO
BnO
STol
OBn
65β
ppm

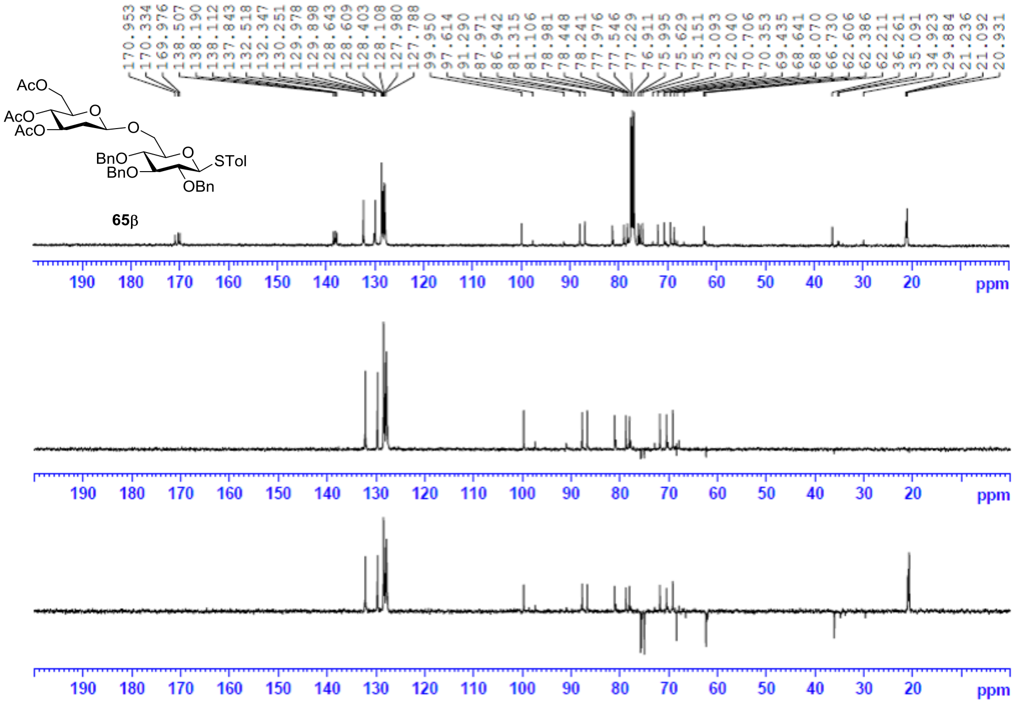
AcO
AcO
AcO
BnO
BnO
STol
OBn
65β
ppm

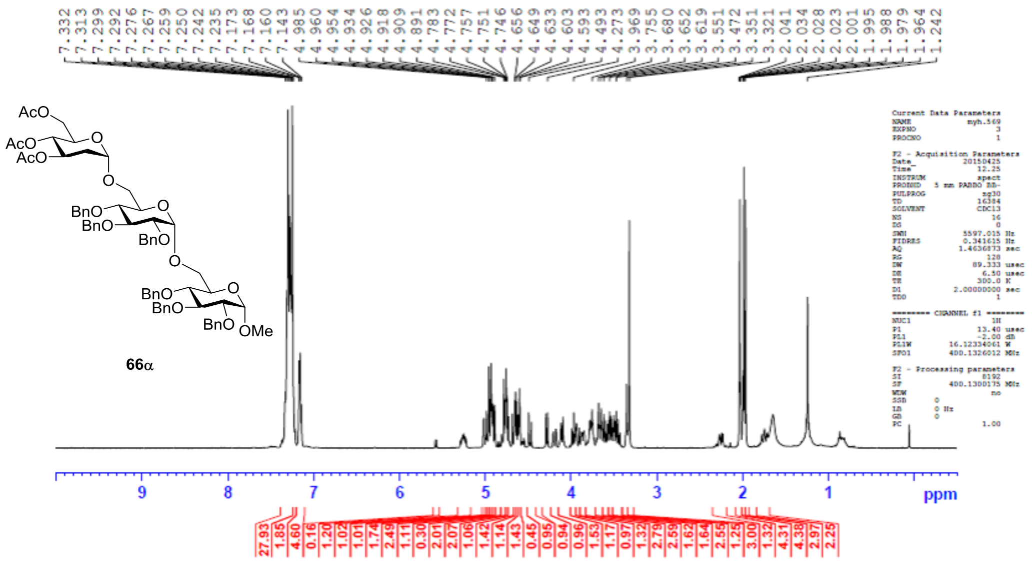
7.332 7.313 7.299 7.292 7.276 7.267 7.259 7.250 7.242 7.235 7.173 7.168 7.160 7.143 4.985 4.960 4.954 4.934 4.926 4.918 4.909 4.891 4.783 4.772 4.757 4.751 4.746 4.656 4.649 4.633 4.603 4.593 4.493 4.273 3.969 3.755 3.680 3.652 3.619 3.551 3.472 3.351 3.321 2.041 2.034 2.028 2.023 2.001 1.995 1.988 1.979 1.964 1.242
AcO
AcO
AcO
O
O
BnO
BnO
BnO
O
O
BnO
BnO
BnO
O
OMe
66α
9 8 7 6 5 4 3 2 1 ppm
27.93 1.85 4.60 0.16 1.20 1.02 1.01 1.74 2.49 1.11 0.30 2.01 2.07 1.06 1.42 1.14 0.45 0.95 0.94 0.96 1.53 1.17 0.97 1.32 2.78 2.59 1.62 1.64 2.55 1.23 3.00 1.32 4.31 4.38 2.97 2.25

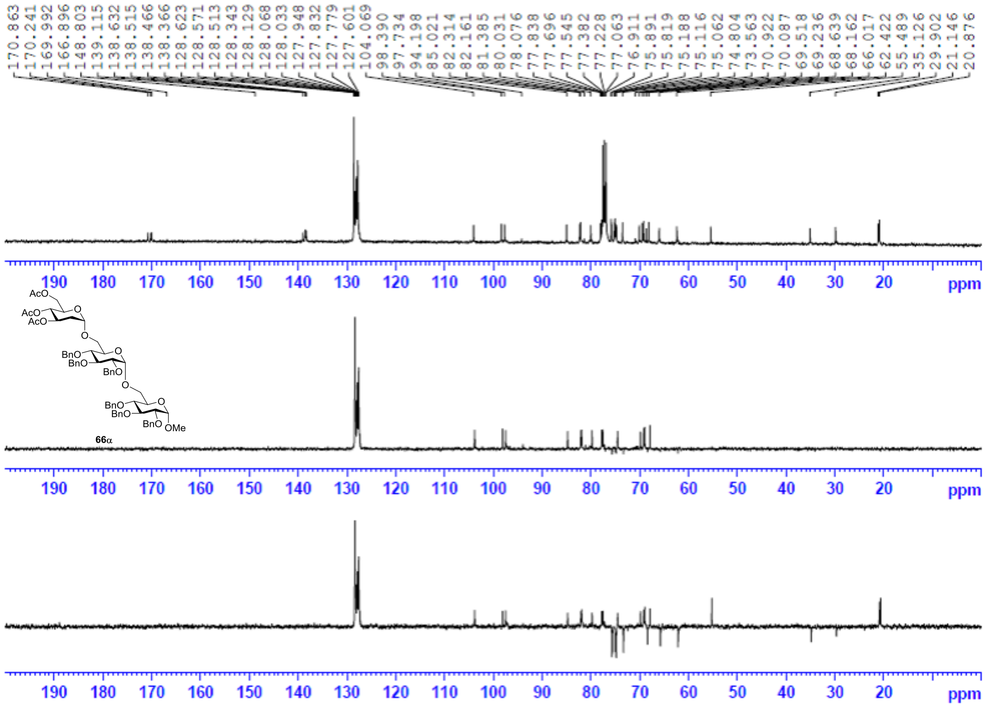
170.863 170.241 169.992 166.896 148.803 139.115 138.632 138.515 138.466 138.366 128.623 128.571 128.513 128.343 128.129 128.068 128.033 127.948 127.832 127.779 127.601 104.069 98.390 97.734 94.198 85.021 82.314 82.161 81.385 80.031 78.076 77.838 77.696 77.545 77.382 77.228 77.063 76.911 75.891 75.819 75.188 75.116 75.062 74.804 73.563 70.922 70.087 69.518 69.236 68.639 68.162 66.017 62.422 55.489 35.126 29.902 21.146 20.876
190 180 170 160 150 140 130 120 110 100 90 80 70 60 50 40 30 20 ppm
AcO
AcO
AcO
BnO
BnO
BnO
BnO
BnO
BnO
OMe
66α
190 180 170 160 150 140 130 120 110 100 90 80 70 60 50 40 30 20 ppm
190 180 170 160 150 140 130 120 110 100 90 80 70 60 50 40 30 20 ppm

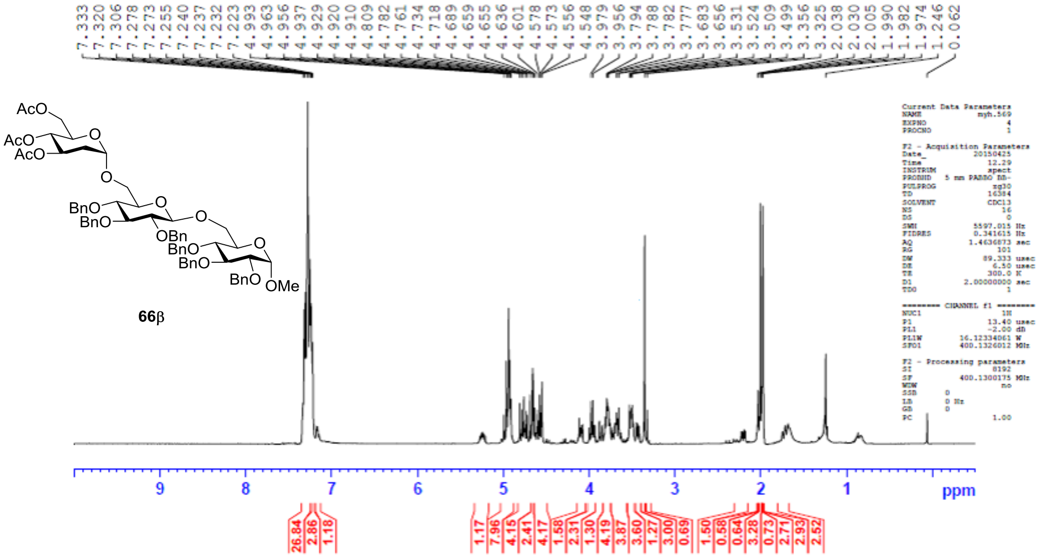

66β

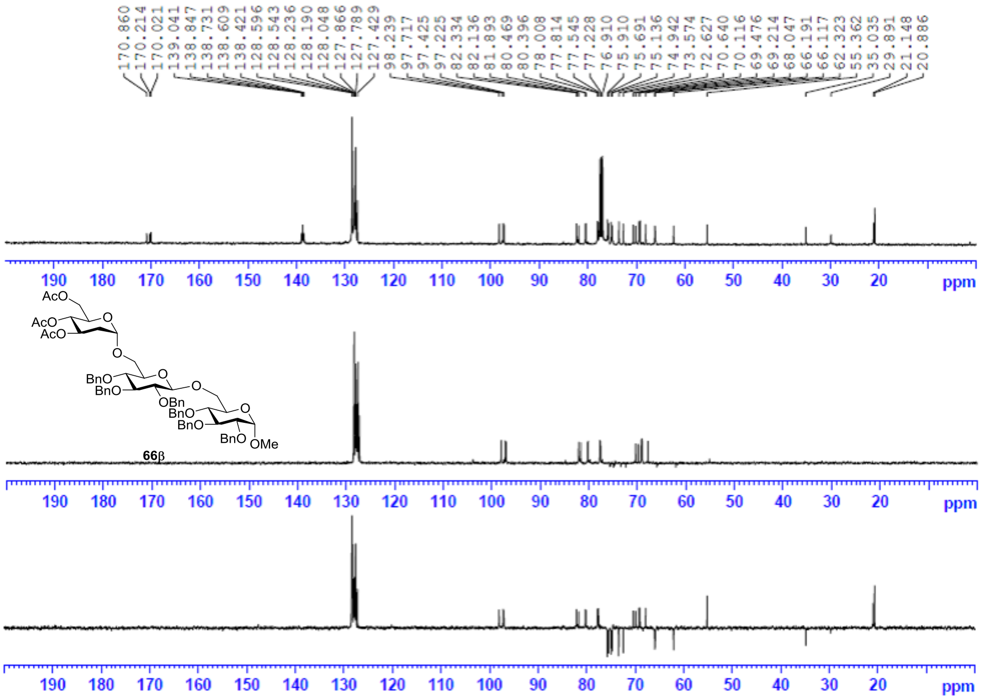

66β
